# Applications of Single-Atom Alloys in Thermal, Electro, and Photo Catalysis

**DOI:** 10.1021/acs.chemrev.6c00369

**Published:** 2026-10-12

**Authors:** Nima Rajabi, Dongbeom Kim, Yicheng Wang, Emma Hollis, Dennis Meier, Toghrul Azizli, Matthew M. Montemore, Adam Holewinski, Dayne F. Swearer, Phillip Christopher, E. Charles H. Sykes

**Affiliations:** † Department of Chemistry, 1810 Tufts University, Medford, Massachusetts 02155, United States; ‡ Department of Chemical Engineering, 8786 University of California, Santa Barbara, California 93106, United States; § Department of Chemistry, 3270 Northwestern University, Evanston, Illinois 60208, United States; ∥ Department of Chemical and Biological Engineering, 1877 University of Colorado, Boulder, Colorado 80303, United States; ⊥ Department of Chemical and Biomolecular Engineering, 5783 Tulane University, New Orleans, Louisiana 70118, United States; # Renewable and Sustainable Energy Institute, 1877 University of Colorado, Boulder, Colorado 80303, United States; ⊗ Department of Chemical and Biological Engineering, 3270 Northwestern University, Evanston, Illinois 60208, United States; ¶ Department of Chemical and Biological Engineering, 1810 Tufts University, Medford, Massachusetts 02155, United States

## Abstract

Single-atom alloys (SAAs) are an emerging class of heterogeneous catalysts that maximize atom efficiency by embedding isolated reactive metal atoms within a host metal surface, creating well-defined active sites. Atomic isolation imparts unique catalytic properties, including high selectivity, resistance to poisoning, coking, and sintering, and in some cases the ability to circumvent conventional scaling relationships that limit traditional catalysts. This article focuses on progress in the SAA field since our 2020 review ( Chem. Rev. 2020, 120, 12044−12088 10.1021/acs.chemrev.0c0007832588624), while incorporating earlier foundational studies where relevant. We first examine the fundamental principles governing SAA formation, stability, electronic structure, reactivity, and selectivity, drawing on insights from experimental single-crystal model systems and theoretical studies. We then review the application of SAAs in a wide range of industrially important reactions, including hydrogenation, dehydrogenation, selective oxidation, water–gas shift, Fischer–Tropsch synthesis, and dry reforming, among others. Beyond thermal catalysis, SAAs demonstrate great promise in electrocatalysis and plasmon-driven chemistry. Their structurally simple active sites have also enabled the rational design of novel catalysts and promoters that challenge conventional catalytic intuition. Looking ahead, recent advances in computing, including machine-learning potentials, offer the opportunity to explore vast compositional spaces and discover new alloy chemistries for challenging reactions using the SAA concept.

## Graphical abstract

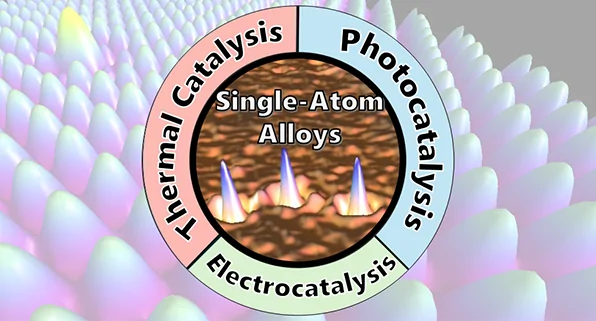

## 1 Introduction

Catalysis is a crucial process for modern life in terms of agriculture, chemical and materials production, and the environment. Homogeneous catalysts typically offer high selectivity and rates for a wide variety of chemistries and due to their relatively simple and uniform structures, are mechanistically well understood. However, challenges in catalyst recovery, reuse and operability in high temperature gas phase environments limit the commercial adoption of many homogeneous systems in the largest scale chemical processes.^1,2^ Similarly, enzymes can mediate rapid and highly selective transformations, but they are generally less tolerant of harsh processing conditions and additives because catalytic activity depends on preserving protein structure.^3^ In contrast, heterogeneous catalysts are often preferred industrially because they are generally more tolerant of high temperature and pressure operating conditions and are easier to recover and regenerate than homogeneous or biological counterparts. Heterogeneous catalysts are foundational to modern chemical industries, enabling essential transformations in fields ranging from energy to pharmaceuticals.^4^ For example, catalytic hydrogenation plays a central role in refining fuels, producing chemicals and fertilizers, and powering devices such as fuel cells and hydrogen storage systems. Traditionally, these transformations rely on supported, precious metal nanoparticles, such as Pt, Pd and Rh. However, these catalysts use precious metals in an atom inefficient way and can have limited selectivity.^5,6^ Furthermore, they are often susceptible to deactivation through active-site blockage or poisoning, loss of surface area via sintering and related processes, and leaching of catalytic species.^7^ In response, there has been growing interest in developing atom-efficient alternatives that decrease precious metal consumption while improving heterogeneous catalyst performance (activity, selectivity and stability).

One of the most significant advances in this direction was the advent of single-atom catalysts (SACs), where isolated metal atoms (often in ionic form) are anchored to high surface area supports such as metal oxides,^8–10^ carbon-based materials,^11,12^ MXenes,^13,14^ zeolites,^15–17^ and transition metal dichalcogenides.^18,19^ SACs can integrate some of the merits of homogeneous and heterogeneous systems by providing more uniform active metal centers alongside improved stability and metal utilization efficiency.^20^ Although SACs have been studied for decades—for example, in 1979 transmission-infrared (IR) studies on alumina-supported Rh demonstrated that isolated Rh atoms facilitate H_2_CO decomposition—the field rapidly expanded in the early 2000s.^21^ For instance, the group of Flytzani-Stephanopoulos demonstrated that trace amounts of cationic precious metals (such as Au and Pt) on reducible oxide supports could catalyze reactions such as the water–gas shift with exceptional activity and stability, outperforming nanoparticle-based systems.^22^ In another landmark study, Zhang and co-workers synthesized a Pt/FeO_*x*_ SAC consisting exclusively of isolated Pt atoms anchored to iron oxide nanocrystals.^23^ This catalyst exhibited outstanding stability and high activity for both CO oxidation and preferential CO oxidation in H_2_. The remarkable performance is attributed to the partially vacant 5d orbitals of the positively charged Pt centers, which lower the CO adsorption energy and reduce activation barriers, as supported by density functional theory (DFT) calculations. Despite these advantages, many SACs are limited in high-temperature applications due to sintering or changes in the active site oxidation state.^24^ For example, DeRita et al. studied the influence of high temperature H_2_ environments on the stability and mobility of single Pt atoms deposited on anatase TiO_2_ nanoparticles.^25^ Above ∼ 573 K in H_2_, a combination of X-ray absorption and CO probe molecule IR spectroscopy suggested that Pt atoms reduced to a near neutral oxidation state, resulting in the formation of mobile Pt atoms that likely serve as precursors for Pt cluster formation. While conditions that induce mobility of isolated metal sites on porous supports depend on both the support and metal composition,^26–28^ the stability of SACs under reducing environments remains a pervasive problem, particularly for precious metals.^29^

Unlike the other SAC examples, which were chemically synthesized on high surface area supports, single-atom alloys (SAAs) emerged from fundamental surface science and scanning probe experiments on single crystals that correlated the isolated atom geometry with, e.g., enhanced H_2_ activation, spillover and selective hydrogenation reactions.^30^ As opposed to a majority of SAC reports, SAAs can uniquely stabilize precious metals as isolated sites in close to neutral oxidation states, imparting distinct reactivity.^31,32^ The most common form of SAAs consists of catalytically active metal atoms embedded within the surface layer of a more inert but more selective host metal, typically a coinage metal such as Cu, Ag, or Au. Initially conceived as a strategy to minimize precious metal usage, SAAs have since demonstrated benefits far beyond cost reduction.^33,34^ A compelling early example of how SAAs emerged from fundamental surface science research comes from a study by the Sykes group using scanning tunneling microscopy (STM) and temperature-programmed desorption (TPD) under ultrahigh vacuum (UHV) conditions. They directly visualized isolated Pt atoms embedded in a Cu(111) surface and demonstrated that these PtCu SAAs enable selective partial hydrogenation of butadiene to butenes, an important reaction in the polymer industry. Specifically, the isolated Pt atoms enabled low-temperature H_2_ activation and H atom spillover to sites on the Cu host where the weak binding of H enabled selective hydrogenation. These results informed the synthesis of supported PtCu SAA catalysts by Flytzani-Stephanopoulos and co-workers who demonstrated the selective partial hydrogenation of butadiene under industrially relevant conditions.^35^

Shortly after the concept of SAAs was demonstrated in model systems, supported SAA nanoparticle catalysts were shown to offer superior selectivity, stability, coke and CO poisoning resilience as compared to conventional nanoparticle-based systems (Table 1).^36–38^ In a broad sense, the emergence and development of the SAA catalysis field can be divided into several overlapping stages (Table 1). Early studies of dilute bimetallic catalysts established that ligand and ensemble effects are important factors that dictate the chemistry of alloy surfaces. The development of chemically sensitive, atomically resolved scanning tunneling microscopy subsequently enabled the constituent elements and local order of alloy surfaces to be visualized directly. Building on these foundations, model-surface studies demonstrated that isolated guest atoms could activate reactants, suppress pathways requiring larger ensembles, and cooperate with the surrounding host metal to produce unexpected catalytic properties that were not a simple interpolation between the constituent metals. These insights informed the synthesis of supported SAA nanoparticles and the extension of the concept to hydrogenation, dehydrogenation, oxidation, and many other reactions as well as SAA incorporation in electrocatalysis and plasmon-assisted chemistry. More recently, operando characterization has revealed that the geometric and electronic structures of SAA active sites can evolve under reaction conditions, while first-principles calculations, data-driven screening, and general electronic-structure rules have enabled increasingly predictive design across wide regions of SAA composition space.

**Table 1:** Broad Chronological Eras in the Development of the SAA Catalysis Field with Representative References

**Period**	**Developmental stages leading to SAAs**	**Key milestones and references**
**1970s–1998**	**Ensemble and ligand effects describe alloy surface chemistry**	Alloy catalysis studies established that dilution could eliminate large ensembles that can lead to undesired reactions. Geometric (ensemble) and electronic (ligand) effects were used to describe molecular adsorption and reaction. STM subsequently enabled the constituent elements of metal-alloy surfaces to be distinguished with atomic resolution.^39–42^
**1998**	**Dilute surface alloys**	Dilute Au incorporated into Ni surfaces disrupted the contiguous Ni ensembles associated with carbon nucleation, thereby improving resistance to coking during steam reforming. In another study suitably spaced noncontiguous Pd monomer pairs were hypothesized as the active motif for vinyl-acetate synthesis but not directly characterized.^43,44^
**2009**	**Single atoms perform a catalytic elementary step**	Isolated Pd atoms in a Cu surface were shown to dissociate H_2_ and enable H atom spillover to the Cu host.^45^
**2009–2016**	**Establishment of the general SAA concept and translation from single crystals to supported nanoparticles**	A 2012 study of atomically isolated Pd in Cu surfaces demonstrated highly selective hydrogenation of e.g. acetylene to ethylene and the SAA term was coined. The concept was then demonstrated in PdCu nanoparticles in 2013 and later PtCu nanoparticles including butadiene hydrogenation under relevant reaction conditions, followed by the demonstration of CO-resistant PtCu SAA catalysts.^35,37,46,47^
**2016–2018**	**Expansion to alkane and alcohol chemistry and fundamental electronic principles**	PtCu SAAs extended isolated-site catalysis to alcohol and alkane chemistry; C–H activation and selective dehydrogenation while suppressing coke formation. Parallel computational studies showed how the energetic landscapes of some SAAs can circumvent conventional scaling relationships, while experimentally observed free-atom-like dopant d-states provided a picture of their unique electronic structures.^38,48–50^
**2018–2021**	**Broadening application, scale-up and first predictive design**	SAAs were demonstrated to catalyze many different chemical reactions and play a role in electrochemical and plasmon-assisted catalysis. Scalable SAA synthesis and data-driven discovery were demonstrated. First-principles and artificial-intelligence screening surveyed large SAA composition spaces and theory-led prediction followed by surface-science and nanoparticle validation produced a selective, coke-resistant RhCu propane-dehydrogenation catalyst.^51–56^
**2021–present**	**Dynamic active sites and intuitive understanding**	In situ and operando studies showed that SAAs are not necessarily static: adsorbates can reversibly move isolated atoms to and from the surface and lead to the formation or break up of clusters, while the partial charge of an active dopant atom can change as a function of the reaction environment. More recently, the ten-electron-count rule provided a simple metric for predicting the strength of adsorbate–dopant binding to SAAs. Most recently theory identified, and experiments demonstrated, that isolated Ni atoms increase the ethylene epoxidation selectivity of Ag by 25%.^57–60^

The distinct reactivity of SAA catalysts, often exhibiting high reactivity and selectivity, is in many cases due to the breaking of linear scaling relationships between activation and reaction energies, unlocking catalytic performance unattainable by traditional metallic systems.^49^ Further, their uniform, atomically dispersed active sites allow reaction mechanisms to be understood and enable predictive modeling via theory.^61^ Theory-led screening and first-principles modeling have recently predicted new SAA compositions and active-site motifs for difficult chemistries and subsequent synthesis and catalytic testing have validated these predictions, confirming the anticipated activity, selectivity and stability under reaction conditions.^56^ Over the past decade, SAAs have been applied to a wide range of reactions, delivering outstanding performance in areas such as CH_4_ activation,^62^ CO_2_ conversion,^63^ selective hydrogenation,^35,46,47,64–67^ dehydrogenation,^57,68–70^ oxidation,^60,71^ and coupling reactions^72,73^ among many others. Since the 2020 comprehensive review of SAA catalysis,^33^ the field has grown rapidly and expanded beyond classical thermal catalysis into electrocatalysis^74^ and plasmonic photocatalysis,^75^ as will be discussed in this review. All three areas are discussed here, with thermal catalysis covered in the greatest depth simply because it remains the most extensively studied.

This review focuses on the emerging chemistry, physics, and catalytic behavior of SAAs, with particular emphasis on how dopant site isolation in a metallic host enables reactivity patterns that differ fundamentally from those of conventional nanoparticles, bulk alloys, and single-atom catalysts on supports. We highlight advances in the synthesis, structural and spectroscopic characterization, and theoretical description of these systems, then examine how these insights inform their performance across thermal, electro-, and photocatalysis. Particular attention is given to mechanistic concepts such as adsorbate binding, spillover, bond activation, selectivity control, and dynamic restructuring under reaction conditions. We also discuss emerging extensions of the field. Rather than providing an exhaustive catalog of all reported systems, this review emphasizes the fundamental design principles that connect atomic-scale structure to function and identifies key opportunities and challenges for the future development of SAAs. When discussing SAA composition (as well as other dilute alloys) in this review, we will use the format XY, where X is the dopant and Y is the host metal; e.g., PdCu refers to Pd atoms doped in Cu.

To improve the accessibility of the information presented throughout this review, each major section concludes with a summary table that compiles all of the SAA systems discussed in that section. For each SAA, the tables provide a concise overview of the key information covered in the text, including the catalyst composition, reaction, principal findings, and other relevant metrics where applicable. These section-specific summaries are intended to serve as convenient references, allowing readers to quickly compare different SAA systems and identify important trends without having to revisit the full discussion.

## 2 Fundamental Properties

### 2.1 Structure and Stability of SAAs

Understanding the thermodynamic stability of alloyed structures is essential for rational catalyst design, as it determines whether the desired atomic configurations will be stable and will be maintained under reaction conditions.^76,77^ No̷rskov and co-workers began systematically investigating the stability of surface alloys using DFT in the late 1990s and related these to experimental data.^78,79^ Two factors considered were the aggregation and segregation energies (Figure 1a). These energies determine whether single atoms remain isolated and in the surface layer and, hence, whether an SAA is formed with an assumption that enthalpy predominantly controls thermodynamic stability. Calculations of the aggregation and segregation energies most often do not include the effect of adsorbates for simplicity and generality; however, as discussed further below (Section 2.3.4) adsorbates can affect these energies.

**Figure 1:**
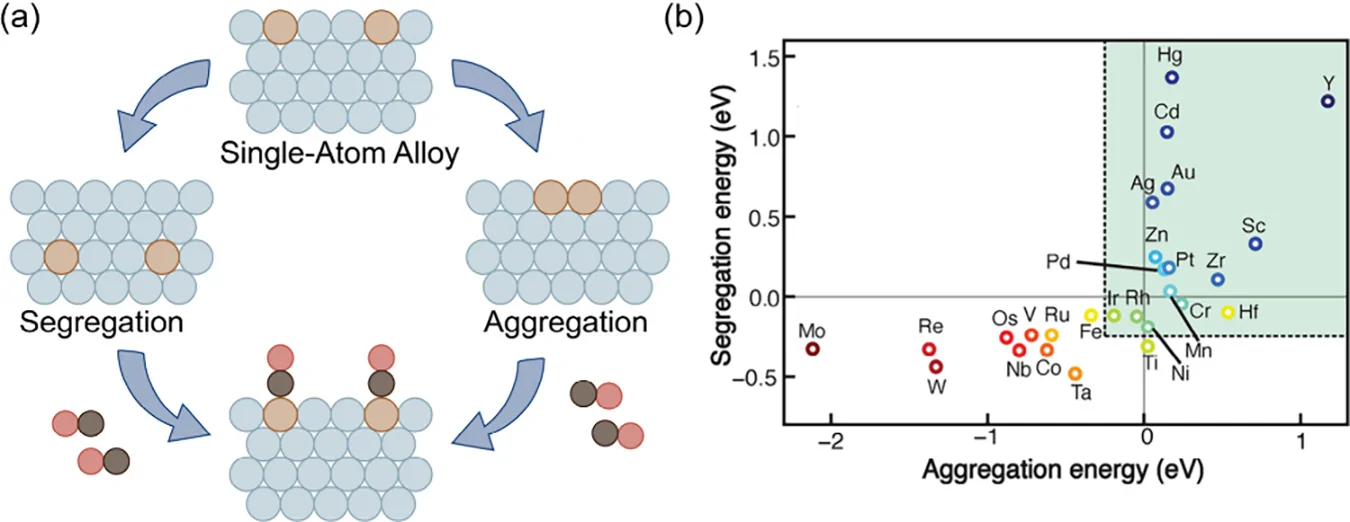
(a) Influence of segregation and aggregation on SAAs. (b) Segregation vs aggregation energy for 27 dopants in Cu(111). The green region indicates potentially stable SAAs. An entropic contribution of 0.25 eV is assumed. The stability of an SAA is represented by a gradient from blue to red, where dark blue circles denote the most stable SAAs, and dark red circles indicate no SAA formation. Adapted from ref.^85^ Copyright 2025 The Authors. Licensed under CC BY 4.0.

Aggregation energy (Δ*E*_agg_) provides a measure of the thermodynamic driving force for dopant atoms in an alloy surface to either remain dispersed as isolated single atoms or form larger clusters.^80–84^ There are different methods to calculate the aggregation energy at the dilute limit. However, it is often determined by comparing the formation energy of dimers or trimers to that of an isolated single atom:

$$\Delta E_{\mathrm{agg}}=E_{\mathrm{tot}}\left(n\right)+\left(n-1\right)E_{\mathrm{tot}}\left(\mathrm{host}\right)-nE_{\mathrm{tot}}\left(\mathrm{SAA}\right)$$ 1

where *E*_tot_, *E*_tot_(host) and *E*_tot_(SAA) are the total energies of an alloy with a cluster of *n* dopant atoms (often 2 or 3 are used in the calculation), the host metal without any dopant atom, and the SAA, respectively. Negative aggregation energies indicate the formation of, e.g., dimers or trimers and thus predict a tendency to cluster, whereas positive aggregation energies indicate that the isolated dopant atom is more stable than clusters.

The other commonly considered factor is the segregation energy (ΔE_seg_), which quantifies the relative stability of a dopant atom in the surface layer of the host vs in the bulk and is most often defined as

$$\Delta E_{\mathrm{seg}}=E_{\mathrm{tot},\mathrm{bulk}}-E_{\mathrm{tot},\mathrm{SAA}}$$ 2

where *E*_tot,bulk_ and *E*_tot,SAA_ are the total energies of the dopant atom in the bulk (e.g., third layer) and with the dopant in the surface, respectively. A negative segregation energy indicates a thermodynamic preference for the dopant to migrate into the bulk (in the absence of adsorbates), decreasing the number of active surface sites and shifting the catalytic properties toward those of the host material. Conversely, positive values favor dopant stabilization at the surface, preserving the SAA configuration and its associated catalytic performance.^55,86–88^ An excellent representative example is the DFT study of Cu(111)-based SAAs in Figure 1b where segregation and aggregation energies were calculated for 27 different dopants, and 16 were found to form stable SAAs.^85^ Several of the SAAs that were predicted to be stable were already synthesized by experimentalists.^35,45,89–91^ Hence, the combination of segregation and aggregation energies can provide a good first approximation of SAA stability. While enthalpy is the primary thermodynamic driving force that dictates SAA stability, configurational entropy can also contribute and was shown to influence host nanoparticle (NP) size dependent SAA formation.^92^

Recent advances in computational catalysis have combined DFT and machine learning (ML) to investigate how segregation and aggregation energies influence the stability of many SAAs. DFT calculations provide atomistic insight into electronic structure and bonding interactions that govern dopant behavior on alloy surfaces. However, because these simulations are computationally intensive, DFT has limits with respect to large-scale screening. By incorporating descriptors such as electronic configuration, atomic size, electronegativity, and cohesive energy, ML methods can reproduce and predict trends in dopant stability across a wide range of alloy systems and surface facets with reasonable accuracy. For example, Rao et al. used DFT to study the stability of SAAs by comparing them with subsurface, dimer, and adatom configurations. Using a data set including 28 host metals (26 d-block metals plus Al and Pb), they identified 250 stable SAA combinations (Figure 2).^82^ In a second step, they compared different ML approaches to reproduce their DFT data. A hybrid model using bonding configurations and tabulated atomic properties with a kernel ridge regression algorithm was able to correctly identify 190 SAAs with 85 false positives. Furthermore, the model was able to generalize to different geometries not included in the training data.

**Figure 2:**
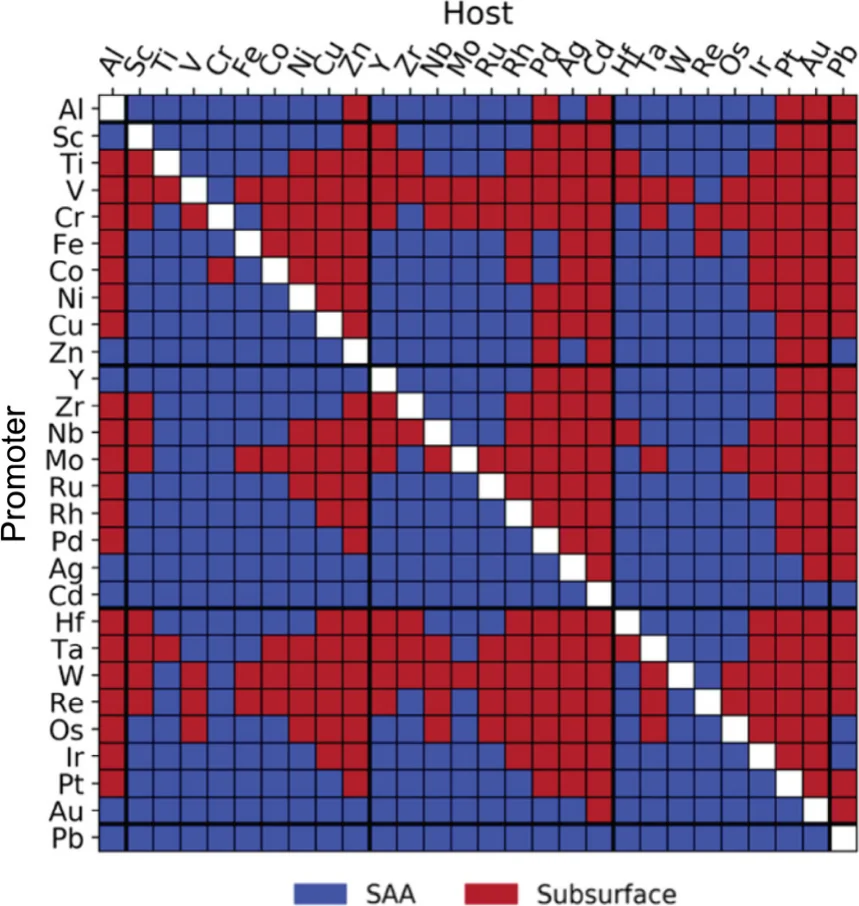
DFT data for the SAA stability vs the dopant atom in the subsurface. Adapted with permission from ref.^82^ Copyright 2020 Springer.

In a different study, Mpourmpakis and co-workers used a five-feature model employing second-order polynomial kernel ridge regression to investigate FCC hosts (Ir, Pd, Pt, Rh) with FCC, BCC, and HCP dopant atoms.^88^ They applied their model to Ir- and Pd-based SAA NPs and were able to predict segregation energies with a mean absolute error of 0.24 eV (Figure 3). A third example used ML to investigate the stability of SAAs in terms of segregation and dimer and trimer aggregation energies for 3d, 4d, and 5d transition metals (756 different combinations). The authors trained multiple machine learning models (including linear, tree-based, and neural network approaches) to predict segregation and aggregation energies, finding that gradient boosted trees yielded the best performance. They showed that segregation energy is primarily governed by the d-band filling of both solute and host, the host nearest-neighbor distance, and the solute d-bandwidth, whereas aggregation energy depends mainly on the Pauling electronegativities of the host and solute, the host nearest-neighbor distance, and the host cohesive energy. Using these models, 266 out of 756 combinations were predicted to form stable SAAs, with gradient boosted trees achieving an R^2^ of 0.91 and MSE of 0.05 eV^2^ for segregation energies, and an R^2^ of 0.44 and MSE of 0.49 eV^2^ for trimer aggregation energies.^83^ These examples demonstrate that the synergy between DFT and ML not only accelerates the screening of promising metal combinations, but also the use of physically interpretable ML models deepens understanding of the fundamental parameters controlling SAA formation and stability. The broader capabilities and limitations of such data-driven models are discussed critically in Section 2.6.1.

**Figure 3:**
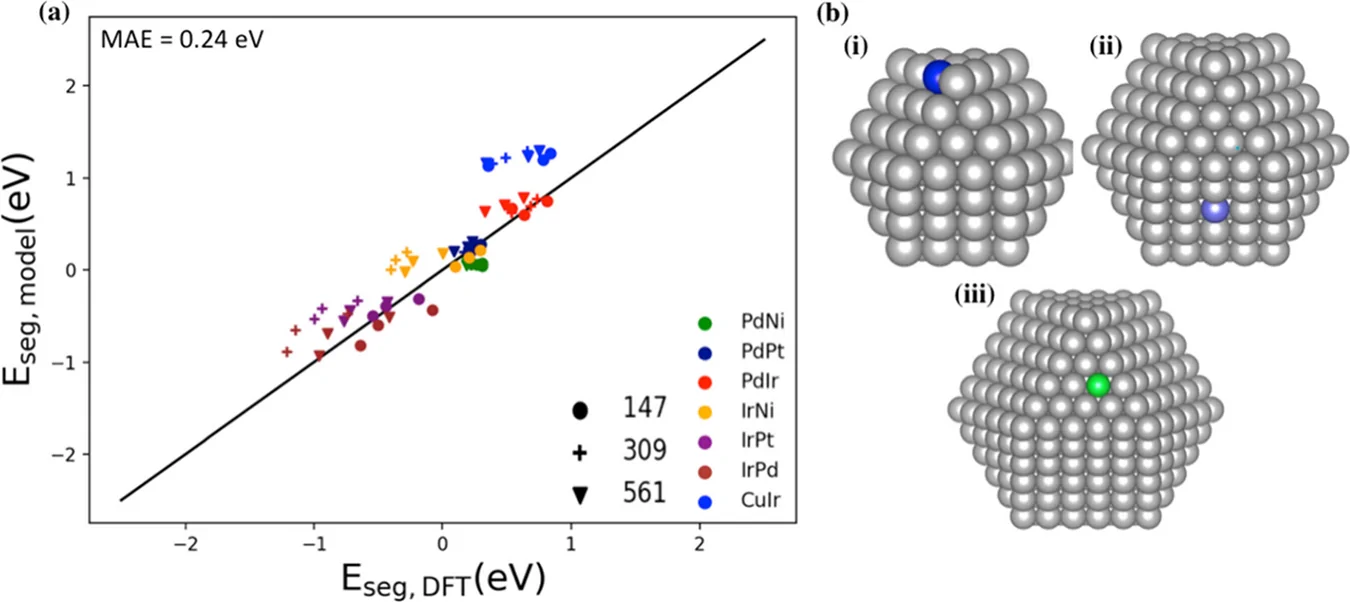
(a) Parity plot comparing model- and DFT-predicted segregation energies for Pd- and Ir-based SAA nanoparticles. The shape of the data points represents nanoparticle size (147-, 309-, or 561-atom), while their color denotes the host–dopant combination. (b) Representative nanoparticles of different sizes and coordination numbers (CNs): (i) 147-atom NP with a dopant with a CN = 7, (ii) 309-atom NP with a CN = 8 dopant, and (iii) 561-atom NP with a CN = 9 dopant. Reproduced from ref.^88^ Copyright 2022 The Authors. Published under CC BY-NC-ND 4.0.

While many studies have examined SAA stability from a thermodynamic perspective, there is still a question of whether kinetics associated with alloy formation enable the system to reach thermodynamically preferred states. Comparative studies of Pd and Ag deposition into Cu(111) surfaces reveal that subtle differences in diffusion kinetics and step-edge interactions can lead to markedly distinct alloying pathways, which can dictate the local structure of formed SAAs.^93^ STM shows that Pd atoms deposited near room temperature are efficiently trapped at ascending Cu step edges, resulting in localized incorporation near terrace boundaries. Alternatively, Ag atoms remain highly mobile, readily crossing step edges and alloying across terraces far from their initial landing sites. DFT calculations clarify this behavior by demonstrating that, despite Pd having a lower intrinsic barrier for step-edge crossing than Ag, its much stronger binding to ascending step edges effectively immobilizes it once captured. Consequently, Pd atoms have only limited opportunities to escape a given terrace before becoming incorporated, while Ag atoms experience repeated attempts to overcome the Ehrlich–Schwoebel barrier and thus sample a broader range of surface sites prior to alloying. These findings underscore that surface alloy formation is governed not only by thermodynamic driving forces but also by kinetic constraints associated with diffusion and step-edge binding. This consideration is important for both surface science and NP SAA studies, as the specific synthetic protocols may dictate alloying mechanisms and result in structures not at the thermodynamic energy minimum.

The stability of dopant atoms in the surface is further influenced by the surrounding environment. Dopants typically have higher surface free energies than the host metal (particularly in the original SAA embodiment where more reactive dopants are alloyed with coinage metal hosts), making segregation into the bulk energetically favorable under vacuum conditions.^94^ However, the presence of adsorbates, such as CO, can reverse this tendency (Figure 1a), because strong dopant–adsorbate interactions can stabilize the dopant at the surface.^86^ Furthermore, adsorbates can also have an effect on aggregation.^57^ This will be discussed in detail in Section 2.3.4. To capture these competing effects in a systematic way, adsorption, segregation, and aggregation energies are used as key descriptors for predicting which SAAs are both stable and catalytically active.

The fundamental thermodynamic principles underpinning SAA formation, particularly the factors preventing clustering (high Δ*E*_agg_), are closely related to the material’s resistance to thermal degradation processes like sintering. There are several studies that have reported enhanced sintering resistance of SAAs vs monometallic NPs. In a study by Shan et al., silica supported NiCu SAAs and Cu NPs were compared for the selective nonoxidative dehydrogenation of ethanol.^95^ The Cu NPs exhibited 25% deactivation after 8 h at a reaction temperature of 250 °C and 50% deactivation after 12 h at 300 °C (Figure 4), comparable to previous studies related to NP sintering.^96,97^ In contrast, the NiCu SAA did not deactivate under the same conditions (Figure 4), suggesting sintering resistance, although differences in sintering properties were not directly assessed. Another study showed that adding 1% of Rh to Cu NPs on SiO_2_ enhanced the sintering resistance.^56^ High-angle annular dark-field (HAADF) scanning transmission electron microscopy (STEM) images showed that the Cu NPs were 20× larger than the RhCu particles after being subjected to identical dehydrogenation reaction conditions at 450 °C.

**Figure 4:**
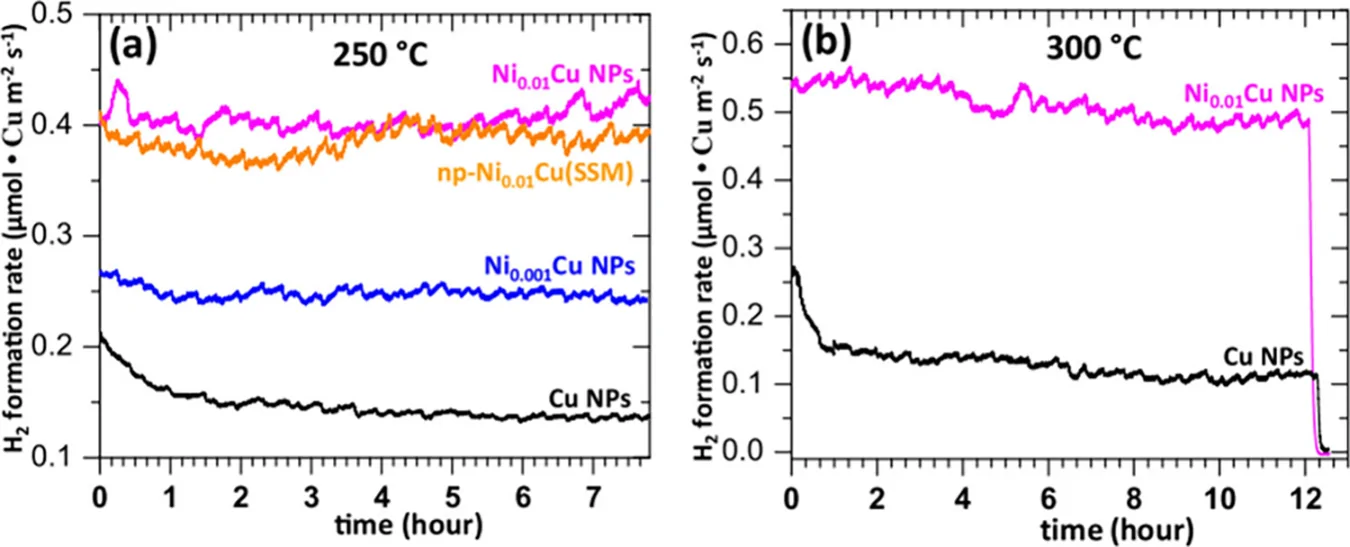
Stability tests of (a) silica-supported monometallic Cu NPs, silica-supported Ni_0.001_Cu NPs, silica-supported Ni_0.01_Cu NPs, and np-Ni_0.01_Cu during ethanol dehydrogenation at 250 °C and (b) of silica-supported monometallic Cu NPs and Ni_0.01_Cu NPs at 300 °C. Reaction rates are normalized to the Cu surface area of each catalyst. Reproduced with permission from ref.^95^ Copyright 2017 Elsevier.

These sparse reports of enhanced sintering resistance of SAAs motivated rigorous analysis of this apparent effect and mechanistic interrogation. Finzel et al. recently prepared 1–2 nm diameter Cu and PtCu_100_ SAA nanoparticles on SiO_2_ and examined their sintering at elevated temperature in H_2_, where the primary sintering mechanism is proposed to be Ostwald ripening.^98^ A combination of methanol synthesis reactivity, transmission electron microscopy (TEM) and *in situ* extended X-ray absorption fine structure (EXAFS) measurements demonstrated substantially suppressed sintering of PtCu_100_ SAAs with substantial particle growth only at ∼ 700 °C compared to 500 °C for Cu. STM measurements and DFT calculations were used to propose that reactive dopants localize near undercoordinated surface Cu atoms and slow the rate of atom emission that is the first step in Ostwald ripening. Further, a regression model was developed that demonstrated a correlation between d-state width of the dopant (once alloyed) and surface stabilization, providing a predictive mechanistic picture for the design of sinter-resistant SAAs. However, the role of reactive environments and the influence of adsorbates binding to the dopant and host were largely unexplored in this work.

In addition to SAA promotion of catalyst stability through sintering resistance, SAAs also provide a clear demonstration of how atomic-scale dilution can decouple activity from deactivation, enabling coke-resistant reactivity. A representative example is PtCu SAAs, which combine the high C–H activation capability of Pt with the weak binding of carbon-containing species to Cu.^38^ Across surface science experiments, simulations, and catalytic tests, PtCu SAAs were shown to activate C–H bonds in small hydrocarbons at substantially lower temperatures than Cu, while avoiding the rapid coking that deactivates Pt-based catalysts. DFT and kinetic modeling revealed that these materials exhibit intermediate activation barriers and endothermic formation of deeply dehydrogenated species, rendering the surface active yet self-limiting against carbon buildup. As a result, PtCu SAAs sustain stable C–H activation under realistic conditions, illustrating how SAAs can deliver both reactivity and intrinsic resistance to coke formation through atomic-scale design. Another example investigated by Kruppe et al. is the selective hydrogenation of acetylene to ethylene on a PdCu(111) SAA model catalyst under ambient pressure (C_2_H_2_:H_2_ ratio of 1:100).^99^ They observed ethylene formation on the PdCu(111) SAA, whereas Cu(111) showed no reactivity under the same conditions. They also found that carbon deposition on the surface decreased as the Pd coverage increased from 0.28% to 8.5% of a monolayer. Thus, Pd appears to suppress carbon deposition on the surface.

To summarize, the formation and stabilization of SAAs must be considered from both thermodynamic and kinetic perspectives. Extensive analyses have considered the stability of SAA formation compared to dopant aggregation or bulk segregation to identify initial targets. However, the unique alloying mechanisms of different dopants, as well as the influence of synthetic conditions raise questions on whether kinetics or thermodynamics dictate initially formed catalyst structures. As catalysts are brought under reaction conditions, adsorbates can substantially influence the stability of the dopant site (discussed further below in Section 2.3.4), but also SAA formation can suppress overall deactivation rates through sintering and coking resistance. These studies highlight that SAAs can be quite stable (both locally at the active site structure level and from an overall catalytic activity perspective) under harsh hydrogenation conditions (which is not common for SACs), although further work is needed to quantitatively assess the distribution of dopant locations under reaction conditions.

### 2.2 Electronic Structure

The d-band model offers a foundational framework for understanding reactivity trends in metals and alloys.^100–102^ Within this model, the chemical nobility or reactivity of a metal surface is largely governed by the position and filling of the *d-*band with respect to the Fermi level, as well as the strength of molecular orbital coupling with adsorbates. Transition metals on the right side of the series have fuller d-bands that lie farther below the Fermi level. As a result, they fill more antibonding states, which weakens adsorption and increases bond activation energies. In contrast, metals on the left of the transition series have partially filled *d-*bands closer to the Fermi level and generally exhibit stronger interactions with adsorbates due to greater overlap between metal *d-*orbitals and adsorbate frontier orbitals. This trend explains the characteristic inertness of late transition and noble metals such as Cu, Ag, and Au, whose *d-*bands are nearly full. Additionally, the extent of orbital overlap between the metal and the adsorbate influences both the chemisorption strength and the activation barriers for bond dissociation. Moving down the periodic table, orthogonalization energy cost often increases resulting in weaker bonding (depending on the adsorbate), further enhancing their apparent nobility.^103^

The electronic coupling between the dopant and the host matrix, which determines how the dopant’s d-states hybridize, or remain isolated, within the host’s electronic environment can lead to SAAs with unique electronic structure. For instance, the Schlögl group reported a CuAg SAA containing 0.3 at% Cu, in which the host and the dopant exhibit weak wave function mixing due to the energy mismatch between Cu 3d states and the Ag 4d band.^50^ Valence photoemission spectroscopy (Figure 5a) revealed that the d-band of the Cu dopants in Ag is very narrow, with a width of 0.5 eV, significantly narrower than that of typical metallic surfaces, which have d-band widths ranging from 2.5 to 11 eV.^104^ The electronic structure of these Cu atoms isolated in Ag is more similar to that of a single Cu atom in a vacuum, particularly in terms of the bandwidth, than to bulk Cu. Thus, these states were described as free-atom-like states. A pressed powder CuAg SAA sample demonstrated the persistence of these narrow Cu d-states even under methanol reforming conditions (0.5 mbar of a 1:1 CH_3_OH/H_2_O mixture at 300 °C), as shown by *in situ* X-ray photoelectron spectroscopy (XPS). The authors theoretically investigated 24 possible Ag-based SAAs and predicted free-atom-like states for Ni, Mn, Pd, and Cr. These predictions were later experimentally confirmed, for example, in a PdAg SAA.^105^

**Figure 5:**
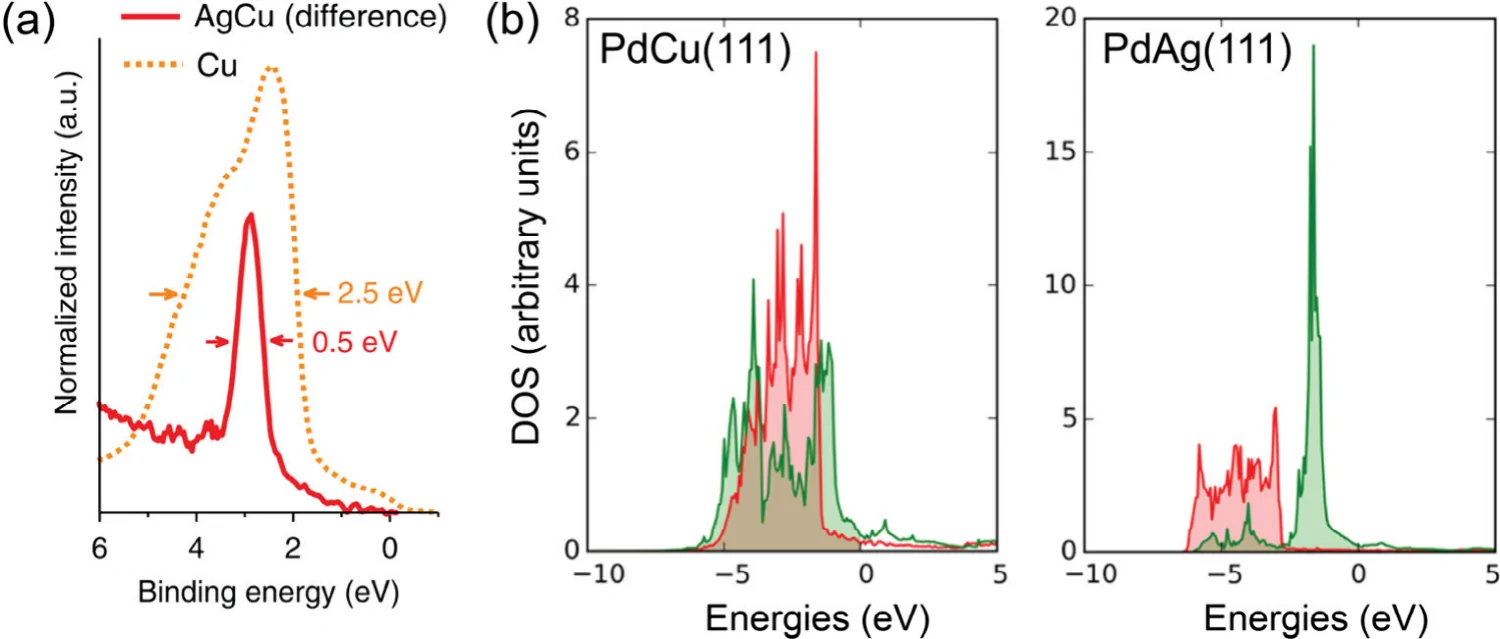
(a) Valence photoemission difference spectrum of AgCu (red) compared to pure Cu (orange). The Cu dopant in Ag exhibits narrow 3d-states relative to the broader peak of metallic Cu, which is ascribed to the free-atom-like electronic state of the dopant in the SAA. Adapted with permission from ref.^50^ Copyright 2018 Springer Nature. (b) d-projected DOS of PdCu(111) and PdAg(111), showing the spectrum of the host metal (red) and the dopant (green). Adapted with permission from ref.^106^ Copyright 2018 Springer Nature.

Thirumalai et al. investigated 15 SAAs with Pt, Pd, Ir, Rh, and Ni as dopant atoms in Cu, Ag, and Au host metals using DFT.^106^ They found that Ag- and Au-based SAAs exhibit narrower dopant d-bands compared to their Cu-based counterparts (Figure 5b), and many feature free-atom-like electronic structures resembling the d-projected density of states (DOS) of single metal atoms in the gas phase. This was attributed to weak host–dopant metal orbital hybridization, resulting in less effective wave function mixing. The authors proposed a competition between two opposing effects: compressive strain, which tends to broaden the d-band, and electronic isolation, which narrows it. In a second phase of the study, they evaluated the catalytic performance using DFT of Pd-based SAAs in various reactions: water dissociation, nitric oxide dissociation, and hydrogenation of acetylene to ethylene. Among these systems, PdAu and PdAg exhibited higher reactivity than PdCu, and both PdAu and PdAg exhibited free-atom-like states near the Fermi level. In contrast, the lower reactivity of PdCu was attributed to the absence of this state, due to stronger wave function mixing between Pd and Cu, which forms a band that lowers the density near the Fermi level. This transition from a narrow electronic state to a broader band was demonstrated for the PdAu system, where DFT calculations showed that increasing the number of next-neighbor Pd atoms, from single atoms to dimers and trimers, leads to a broadening of the Pd *d-*band.^57^ The reactivity of these different ensembles was also markedly different than Pd in the SAA configuration, experimentally exhibiting 100% selectivity for ethanol dehydrogenation, whereas Pd clusters are less selective and also promote C–C bond scission and CO formation. Thus, both electronic and ensemble-size effects can potentially play a role in the unique reactivity of SAAs.

Monasterial et al. examined the correlation between the d-band center and H adsorption on Ag-based SAAs with 21 dopants from across the d-block.^107^ The d-band center correlated poorly with H adsorption energies (R^2^ = 0.34) for SAAs, with improved correlations for dopant dimers (R^2^ = 0.63) and trimers (R^2^ = 0.72). This study, along with a study of Ag(211) alloys,^108^ also showed that free-atom-like states near the Fermi energy can lead to counterintuitive, nonmonotonic adsorption trends with increasing ensemble size, due to dopant–dopant hybridization.

Berger and Michaelides conducted a DFT study on the full series of 4d transition metal atom dopants embedded in Cu and Ag in a range of coordination environments: in the bulk, in the (111) and (100) terminated surfaces, and at step edges (using a (211) slab).^109^ These environments range from strong confinement (Cu(111)) to weak confinement (Ag(211)), allowing the authors to examine how confinement influences the free-atom-like d-states. The differences in confinement in this study are determined by the number of nearest neighbors, ranging from 12 in the bulk (highest confinement) to 7 for dopants at step edges (lowest confinement), as well as by the dopant–next neighbor host atom distances of 2.55 Å for Cu (higher confinement) and 2.89 Å for Ag (lower confinement). They found that free-atom-like d-states are characteristic of the late transition metals (TMs), whereas early TMs exhibit broad d-states often comparable in width to those of the host. Furthermore, the difference in the d-state energies of host and dopant atoms was determined not to be the primary factor driving the formation of sharp free-atom-like d-states, because the d-states of late TMs are actually closer in energy to the host d-band than those of the early TMs which did not exhibit free-atom-like states. Their calculations indicated that the spatial orbital overlap of the dopant and host atoms was a more important factor, and that late transition metals with their smaller atomic radii hybridized poorly, leading to free-atom-like states (Figure 6). In addition to size differences, hybridization between dopant and host states becomes increasingly unfavorable from early to late transition metals because of higher d-orbital population leading to filling of antibonding states. Finally, weaker confinement was shown to produce narrower dopant d-bands, particularly for early transition metals. For example, Zr has a d-bandwidth of 2.41 eV in Cu vs 1.02 eV when it is located at a step edge on Ag. Overall, the study indicated that confinement strength and host–dopant hybridization are key descriptors for predicting and tuning free-atom-like d-states in transition-metal-doped systems.

**Figure 6:**
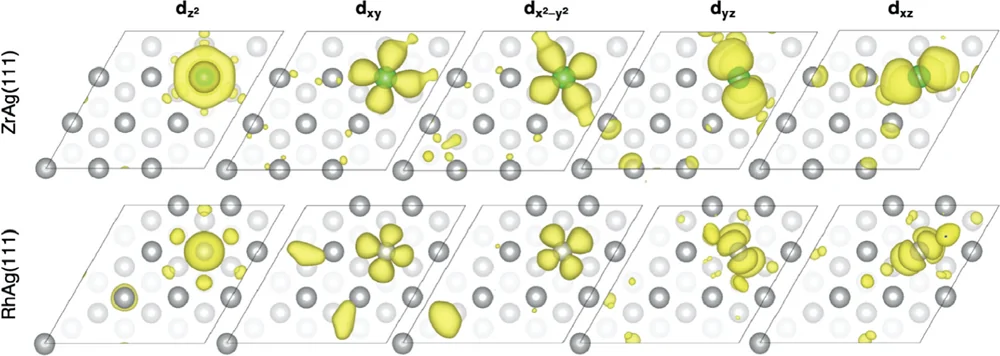
Electron density isosurfaces (yellow) of selected Kohn–Sham states reveal the orbital character and spatial extent of the dopant d-states. The early TM dopant Zr (green atom), embedded in a Ag(111) surface (gray atoms), displays more spatially extended, atomic orbital–like states compared to the more localized d-states of the late TM dopant Rh (green atom). Surface atoms are shaded according to depth: top layer (dark gray), second layer (medium gray), and third layer (light gray). Adapted from ref.^109^ Copyright 2025 The Authors. Licensed under CC-BY 4.0.

In a study conducted by Huang and co-workers, a deep learning framework was developed that combined tight-binding moment theory with graph neural networks to reveal the underlying electronic structure of SAAs and the influence of creating vicinal heteroatom dopants.^110^ By quantitatively describing how interatomic orbital coupling and on-site orbital resonance dictate the d-band features of active sites, the approach provided a mechanistic understanding of the emergence of free-atom-like d-states. The framework was applied to analyze the evolution of d-band characteristics in SAAs under two catalytically relevant scenarios: varying coordination environments and dopant-induced modifications. For Pt_1_Ag SAAs on different FCC facets, a systematic narrowing and upward shift of the d-band were observed with decreasing CN, consistent with weakened orbital coupling. In doped Pt_1_M_1_Ag SAAs, where a M is a next-neighbor to Pt, variation of the dopant metal across the transition series (from left to right) revealed that decreasing d-orbital radius and energy led to weaker orbital overlaps, resulting in a narrower, higher-lying d-band. Exceptions observed for d^10^ dopants highlight the additional role of delocalized *sp*-states beyond the tight-binding description. Overall, this theory-guided data-driven approach refines traditional d-band theory and establishes a new framework for predicting and designing SAA catalysts with predictable electronic and catalytic properties.

Finally, Zhang et al. investigated the electronic structure of isolated dopant atoms in SAAs compared to dimers and trimers by DFT modeling of Pd_1_Ag­(111), Pd_2_Ag­(111), and Pd_3_Ag­(111) with a focus on the formate oxidation reaction.^111^ The Pd atom in the PdAg(111) SAA exhibits a pronounced, narrow peak in its partial DOS between −0.75 and −2.0 eV, originating from the free-atom-like nature of the Pd atom *d-*states. The d-states of Pd_2_ and Pd_3_Ag­(111) exhibited broader d-states, underscoring the distinct electronic character of SAAs compared to small clusters. However, it is worth mentioning that a theoretical screening based on the open-access Materials Project database, which contains more than 150,000 materials, revealed that free-atom-like d-states can be observed beyond the dilute limit of SAAs.^112^ In particular, localized d-states were identified in a wide range of intermetallic compounds (IMCs), demonstrating that strict atomic dilution is not a prerequisite, although some degree of site isolation remains important to prevent d-band hybridization. Moreover, these localized states are often associated with partial anionic character due to charge transfer from surrounding metals, and their reduced d-bandwidth can significantly influence adsorption properties by increasing orthogonalization energy penalties, thereby weakening adsorbate binding such as CO. Trimetallic systems were even found to exhibit two distinct free-atom-like d-states originating from different dopants as long as site isolation existed.

The concept of free-atom-like d-states has deep roots in the solid-state physics of dilute alloys. Localized d-states on a transition-metal impurity that hybridize only weakly with the surrounding electrons were described decades ago as virtual bound states in the Friedel-Anderson picture, and their interaction with adsorbate orbitals has long been modeled by the Newns-Anderson model of chemisorption, in which a sufficiently narrow metal d-band splits into discrete bonding and antibonding states upon coupling to a molecular orbital.^113–116^ Viewed in this light, the free-atom-like states of SAAs are a surface-catalytic manifestation of a well-established phenomenon, and the same framework clarifies why they are not a universal feature. Whether a dopant retains a sharp, atom-like d-state depends on the degree of host–dopant orbital overlap, which varies continuously with dopant identity, host, and coordination environment.^106,109,112^ The distinction between “free-atom-like” and “band-like” is therefore better regarded as a spectrum than a sharp dichotomy. The literature generally suggests that late transition-metal dopants in coinage-metal hosts often lie near the free-atom-like limit, while early transition-metal dopants often lie closer to the band-like limit, and intermediate compositions and dopant ensembles populate the continuum between them.^107,109^

The extent to which these states directly govern catalytic performance is less clear. Their presence has been correlated with enhanced reactivity in several systems, such as the higher activity of PdAu and PdAg relative to PdCu,^106^ and reactivity gains have been attributed to partial d-band contraction upon alloying, such as low-temperature methane C–H activation on alloyed early transition-metal solutes.^117^ However, the free-atom-like state is difficult to isolate from covarying factors, because tuning the d-state width by changing the dopant simultaneously alters composition, charge transfer, orbital energy, orbital occupation, and adsorption-site geometry. Importantly, these states are not a prerequisite for the defining advantage of SAAs. The ability to break linear scaling relations arises largely from the differences in coordination of transition states and intermediates across chemically and geometrically distinct sites, a site-specific effect that does not require a free-atom-like d-state,^32,49,118^ so that even SAAs without sharp free-atom-like states can remain catalytically interesting. In summary, free-atom-like d-states are a real and now well-characterized feature of many, but not all, SAAs; they appear to modulate adsorption, though frequently to a modest degree, and they are neither necessary nor sufficient for the chemically distinctive behavior of SAAs. Disentangling their specific contribution from other electronic and geometric effects, and quantitatively reconciling their effect with various models of adsorption, remains an open challenge and a worthwhile target for future work. In this context, advanced multireference electronic structure methods have been shown to substantially improve the description of adsorption energetics in SAAs by accurately treating the strongly correlated electronic states of transition-metal dopants.^119^ Specifically, embedded DMRG-NEVPT2 calculations demonstrated that reliable predictions of CO adsorption on transition-metal-doped Ag(100) require large active spaces including all dopant d orbitals, underscoring the importance of beyond-DFT approaches for quantitatively modeling adsorption in these systems.

The preceding discussion focused primarily on studies that examined the shape of the d-states in isolated dopant atoms on host metal surfaces. From a different perspective, the charge state of the dopant atoms is another important electronic property in the design of new SAAs. Réocreux et al. used Bader charge analysis to examine the charge state of late transition-metal dopants (Ni, Rh, Pd, and Pt) in the (111) facet of coinage-metal hosts (Cu, Ag, Au) and correlated the dopant charges with the electronegativity difference between the host and dopant metals (Figure 7a).^32^ For Cu, which has the lowest electronegativity of the coinage metals (χ = 1.90), the investigated dopants are anionic, whereas for Au, which has the highest electronegativity (χ = 2.54), the dopants are nearly neutral or cationic. For example, Ni in Au(111) carries a total charge of 0.32e, whereas Pt in Au(111) has – 0.12e. In another study, Yin et al. used DFT to investigate Ni, Pd, and Pt atoms in Cu(111) and Cu, Ag, and Au in Ni(111), respectively.^120^ They observed that CuNi(111) forms a phase-separated alloy (PSA), an alloy with distinct Cu and Ni phases; AgNi(111) and NiCu(111) form either SAAs or a PSA depending on the dopant coverage; and Pd, Pt, and Au always form SAAs. Yin et al. therefore explored the charge states of dopants in these six systems either in close proximity (1 to 4 dopant atoms) without being nearest neighbors (SAA regime), or as small clusters formed by 1 to 4 dopants (PSA regime). In the SAA regime, they found that dopants are predominantly negatively charged with the exception of Cu in Ni(111) (Figure 7b). With increasing negative charge on the dopants, the Fermi level of the alloy decreases, as the host correspondingly becomes more positively charged. Furthermore, the charge per dopant in the SAA regime is always more negative compared to their corresponding PSAs. Overall, these studies demonstrate that the dopant charge state in SAAs is strongly governed by the electronegativity of both the host metal and dopant. This charge state plays a key role in dictating adsorption behavior and related processes such as spillover, which will be discussed later in Section 2.3.

**Figure 7:**
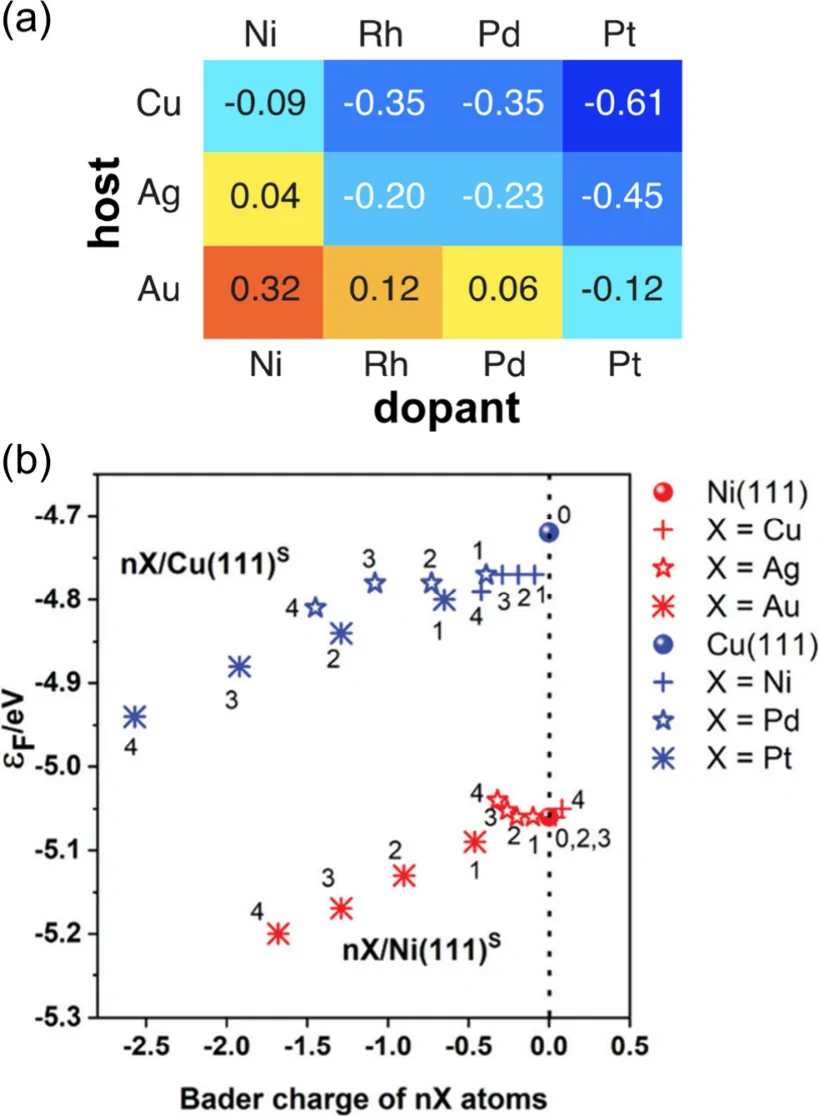
(a) Atomic charge by Bader analysis of different dopants in Cu, Ag and Au host metals. Adapted from reference.^32^ Copyright 2022 The Authors. Licensed under CC BY 4.0. (b) Fermi level vs Bader charge (in e) of nX isolated dopants in Ni(111) (red) or Cu(111) (blue). Adapted with permission from ref.^120^ Copyright 2022 Royal Society of Chemistry.

In addition to enhancing direct adsorbate binding strength, the dopant atoms in SAAs also affect the electronic structure of the surrounding surface atoms, which in turn modulates the binding strength of adsorbates. Recent DFT studies revealed that molecular adsorption near dopant sites can be destabilized by up to ∼ 300 meV, creating a “zone of exclusion” around the reactive site.^121^ This effect originates from a local decrease in the d-projected DOS at nearby host atoms, leaving fewer d-states near the Fermi level available for bonding and thus weakening adsorption (Figure 8). The strength of this electronic perturbation follows periodic trends and depends on the covalent contribution of the dopant’s d orbitals to the surrounding host lattice. Surface science experiments on the RhCu(111) SAA confirmed this predicted effect, revealing that CO diffusion from Cu host sites to the Rh atoms was thermally activated. Similarly, calculations for stepped Ag(211) SAAs showed that dopant substitution nearly always destabilizes adsorption on nearby Ag sites, with subsurface substitution following the same trend.^108^ The primary exceptions were Cd, Zn, and Cu, which could stabilize adsorption on neighboring sites. Notably, the dopants that stabilized or had minimal effect on nearby-site adsorption tended to be those that weakened or minimally affected direct H binding to the dopant itself. Together, these findings demonstrate that dopant-induced bond compensation can reshape the local adsorption landscape in SAAs, potentially providing a new handle for tuning catalytic behavior.

**Figure 8:**
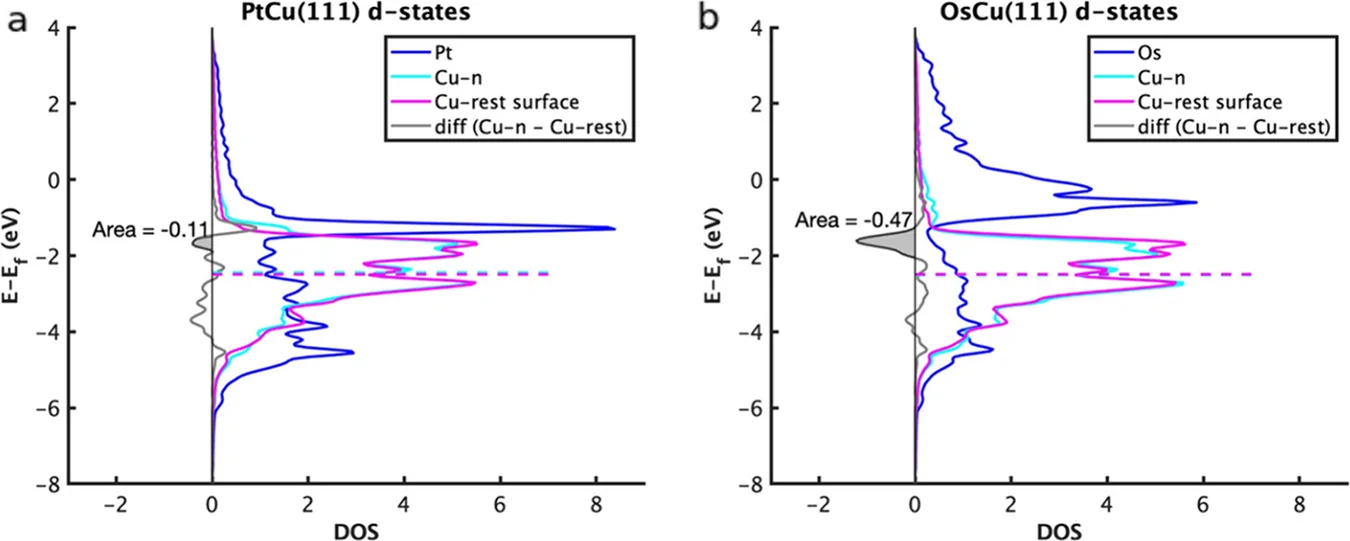
DOS analysis of clean SAAs. The d-state DOS of surface atoms for (a) PtCu(111) and (b) OsCu(111) is shown. The d-states of the single atom dopant, nearest neighbor Cu sites (Cu-n) and the remaining Cu surface atoms (second and third coordination shells around the dopant) are shown in blue, cyan and magenta, respectively. The difference between the n-site Cu atoms and the other Cu surface atoms is highlighted in gray. The shaded gray region represents the difference in d-state DOS at the upper band edge of the Cu atoms. Dotted lines mark the position of the d-band center of the Cu surface atoms. Adapted with permission from ref.^121^ Copyright 2021 American Chemical Society.

In summary, the electronic structure of SAAs is governed by a complex interplay between the dopant d-orbital size and energy, host-dopant hybridization, coordination environment, and charge transfer effects. While the d-band model provides a useful foundation for understanding reactivity trends, deviations in SAAs, particularly the emergence of narrow, free-atom-like d-states, mean that their catalytic behavior can deviate from that expected of the pure metal. The poor performance of the d-band center alone as a descriptor for SAAs motivates models that additionally capture the covalent and ionic contributions to metal–adsorbate bonding. Descriptor frameworks that supplement the d-band center with some combination of the adsorbate-metal coupling matrix element, band filling, and a reduction-potential (ionic) term have been shown to predict adsorption trends across transition and main-group metals, including for data sets that contain SAAs.^103,122–124^ The value of such multiterm descriptors is evident for cases where the d-band center fails outright, such as CO_2_ activation on SAAs, where additional electronic descriptors are required to reproduce the trends.^125^ These band-focused models provide descriptions of how adsorption energies arise from more fundamental electronic properties that are complementary to the ten-electron count rule and spillover-energy descriptor discussed below. Additionally, dopant-induced perturbations to the surrounding host surface and the resulting “zone of exclusion” demonstrate that the dopant’s influence extends a few atoms beyond the isolated active site. Together, these insights underscore the need for a more nuanced, multiparameter framework to accurately predict and design SAA catalysts with tailored reactivity and selectivity.

### 2.3 Adsorption and Chemical Properties

In supported heterogeneous catalysis, molecular adsorption typically marks the beginning of the catalytic process, as reactants must first interact with and bind to the surface before undergoing transformation. The previously discussed free-atom-like d-states of the dopant atoms make SAAs unique compared to extended metal domains in how they interact with adsorbates. In the following section, we discuss adsorption and associated surface processes, like spillover, aggregation and reverse segregation, that occur on SAAs, highlighting the distinctive ways in which SAAs interact with adsorbates compared to conventional metal surfaces.

#### 2.3.1 CO Adsorption on SAAs

Carbon monoxide is often used as a probe molecule to investigate the adsorption behavior of SAAs because CO is a simple molecule that typically adsorbs and desorbs from SAAs reversibly thereby yielding binding energies to active sites which can be compared to DFT predictions. Further, CO is involved in many important industrial chemistries (CO oxidation and preferential oxidation, hydroformylation, Fischer–Tropsch, methane reforming). Moreover, CO also plays a crucial role in catalyst poisoning and is therefore critical to understand along the path of improving heterogeneous processes. For example, the platinum-group metals (PGMs) are highly active catalysts for many heterogeneous processes but are prone to CO poisoning due to their strong CO affinity. Alloying PGMs with coinage metals that bind CO more weakly can mitigate this limitation. Using RPBE DFT-parametrized kinetic Monte Carlo (KMC) simulations, highly dilute alloys of PGM (Pd, Pt, Rh, Ir) and Ni atoms dispersed in the (111) facet of coinage metal hosts (Cu, Ag, and Au) were examined, forming SAAs.^86^ Figure 9a shows the KMC-simulated CO desorption peak temperatures for the 15 SAAs, and where experimental data were available, these values are compared to CO desorption temperatures measured with TPD. The experimental and calculated data show very good agreement, differing by less than 20 K. Ni, Pd and Pt SAAs show reduced CO binding energies and lower CO desorption temperatures compared to the respective pure metals, and therefore enhanced CO tolerance. In contrast, Rh- and Ir-doped SAAs bind CO more strongly, suggesting greater reactivity than their pure metals but with reduced resistance to CO poisoning.

**Figure 9:**
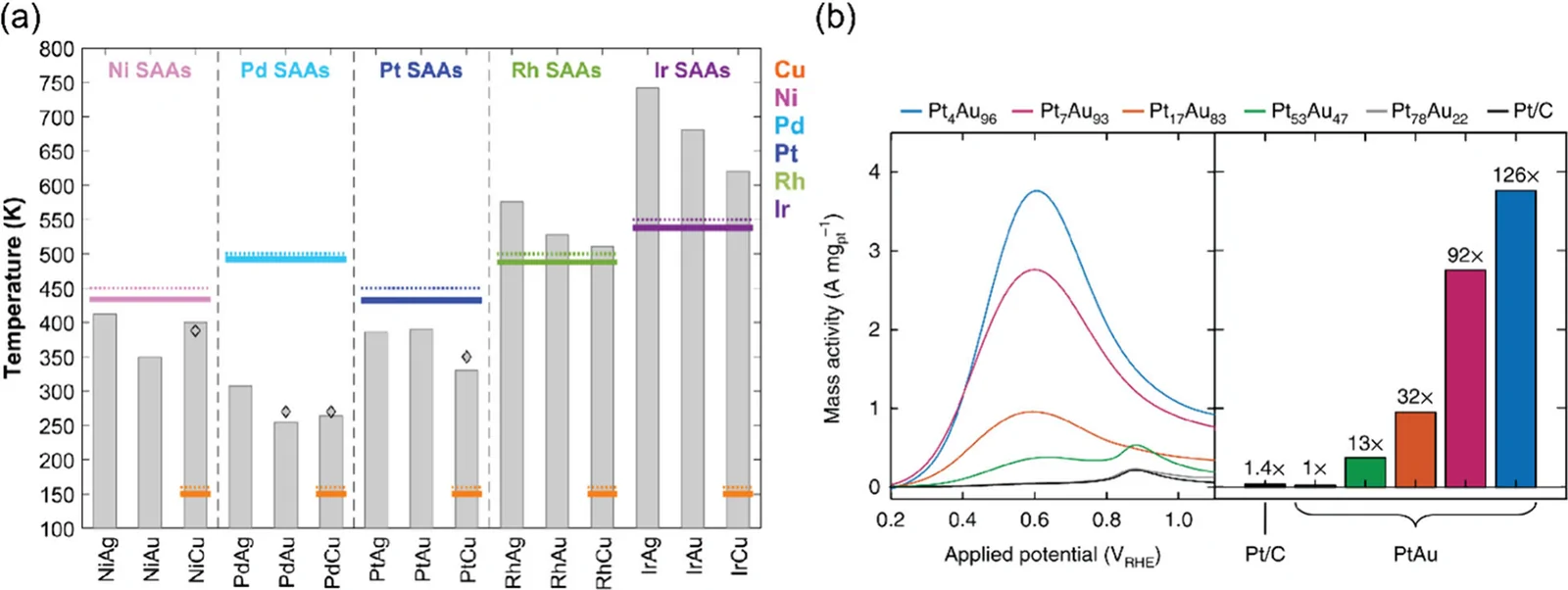
(a) Comparison of KMC (gray bars) and experimental (diamonds) desorption peak temperature of CO on different SAAs. The desorption temperature from the respective dopant metal surface is given in colored horizontal lines (theory) and dotted horizontal lines (experiment). Adapted from ref.^86^ Copyright 2018 The Authors. Licensed under CC BY 4.0. (b) Pt mass-normalized anodic sweeps obtained from PtAu nanoparticle catalysts in an electrolyte containing 0.1 M HClO_4_ and HCOOH. Adapted with permission from ref.^126^ Copyright 2018 Springer Nature.

The CO tolerance of SAA systems has also been demonstrated experimentally by Liu et al., who investigated the effect of CO on the rate of a hydrogenation reaction over PtCu SAA nanoparticles.^37^ Specifically, they studied the selective hydrogenation of acetylene to ethene with and without 200 ppm of CO cofed over a Pt_0.008_Cu SAA and compared the results to Pt nanoparticles supported on γ-Al_2_O_3_. They observed that the reaction rate on the SAA was reduced by half in the presence of CO, whereas the reaction rate on the Pt nanoparticles decreased by a factor of 15 when CO was introduced. Furthermore, using TPD experiments on PtCu(111) single crystals with increasing Pt coverages (from SAA to the multilayer regime) under UHV conditions, Liu et al. showed that CO binds more strongly to Pt ensembles than to isolated Pt atoms, providing further support for the enhanced CO poisoning resistance of SAAs. In a related study on electrocatalytic formic acid oxidation reaction (FAOR), the activity of Pt and PtAu bimetallics ranging from Au-core/Pt-shell to the SAA regime were compared.^126^ Orders-of-magnitude increases of the Pt mass-normalized FAOR activity in the single Pt atom regime (Pt_4_Au_96_) compared to core–shell Pt_78_Au_22_ nanoparticles were observed (Figure 9b). The binding of CO to dopant sites in PtAu(111) SAAs, small clusters in Au(111) and the Pt(111) surface were investigated by DFT. For the atop adsorption site, a decrease in CO binding energy was found from – 1.03 eV on Pt atoms in Au(111) to – 1.27 eV on Pt(111). This trend is consistent with the higher FAOR activity on the SAA by virtue of the weaker binding of CO. Together, these studies demonstrate the weaker binding of CO and thus superior CO resistance of many, but not all, SAAs as compared to the corresponding extended metal surfaces.

Muir et al. used CO adsorption on PdAg(111) to investigate the transition from the SAA regime to the cluster regime by reflection–absorption infrared spectroscopy (RAIRS) and TPD.^127^ They observed a CO stretch band at 2047 cm^–1^ with a desorption peak at 272 K for Pd coverage less than 0.2% of a monolayer, assigned to CO bound to an isolated Pd atom. Increasing the Pd coverage led to a new feature in the IR spectra at 1950 cm^–1^, accompanied by a corresponding CO desorption peak at 390 K, consistent with CO bound at Pd–Pd bridge sites. This demonstrates that Pd ensembles form once the Pd coverage exceeds ∼ 0.2% of a monolayer. For NiCu(111) SAAs, a single CO band (2004 cm^–1^) was observed at low Ni coverages (1.3% of a monolayer) by RAIRS and was assigned to adsorption on single Ni atoms.^128^ A bridge-bound species (1874 cm^–1^) was observed at coverages above 3% of a monolayer Ni, indicating that beyond this point, the system no longer remained in the pure SAA regime. Increasing the Ni dopant concentration from 1.3% to 55% of a monolayer was also accompanied by an increase in CO desorption temperature in TPD, which was attributed to stronger CO binding on Ni ensembles compared to isolated dopants in the SAA, as the SAA limit is strongly dependent on the nature of the dopant and host as previously described.

Hannagan et al. analyzed CO adsorption on RhCu(111) SAA with RAIRS and TPD and confirmed assignments with atomic scale imaging of the alloy surface by STM.^129^ Even at a Rh coverage as low as 0.2% of a monolayer, they observed a broad C–O stretch band at 2007 cm^–1^ (full width at half-maximum (FWHM) 24 cm^–1^). Isotopic substitution experiments confirmed that the band broadening originated from CO dipole–dipole coupling. This indicates that the CO molecules are already in close proximity even at very low Rh coverages, and therefore the Rh atoms themselves must also be close to one another. STM images revealed that Rh atoms were predominantly located in dense brims above the step edges, explaining why these systems exhibited peak shifts consistent with dipole–dipole coupling. However, the absence of a bridge-bonded CO IR feature indicates that the 0.2% system remains in the SAA regime. To further probe this Rh “bunching” effect, Hannagan et al. then performed experiments on a more stepped Cu surface with 0.2% of a monolayer of Rh and observed a narrower C–O stretch peak (FWHM 13 cm^–1^) shifted to lower frequency (1997 cm^–1^). This frequency shift indicates a reduction of dipole–dipole coupling, signifying that Rh atoms are farther apart on the stepped surface rather than crowded above the fewer step edges on the flat Cu(111) surface. The narrow FWHM indicates more uniform Rh site environments. Together, these results highlight the importance of combining real-space imaging with STM and spectroscopic methods.

In addition to roughening the surface, raising the alloying temperature can achieve better single-atom dispersion, although an excessively high temperature can drive dopants to the subsurface. For example, Lucci et al. used STM to show that at 315 K, Pt atoms alloy mostly in regions above steps on Cu(111) and raising the alloying temperature to 550 K significantly increases Pt dispersion in the terraces. Wang et al. observed with STM that the dispersion of the dopant is strongly affected by the facet of the substrate. Rh shows accumulation of dopants above the step edges on Cu(111), whereas in Cu(110) and Cu(100), a more homogeneous distribution is achieved with similar Rh deposition parameters.^130^ This observation was ascribed to differences in energetic barriers for atomic hopping, exchange, swapping, and vacancy filling calculated by DFT.

To examine the influence of host metal structure on the behavior of reactive dopants in SAAs, the adsorption of CO on a dopant in a (211) step and a (100) terrace was examined using DFT and compared to the corresponding (111) surface.^80^ The results revealed strong correlations among CO adsorption energies across facets, resulting in robust scaling relations. Specifically, calculated CO adsorption energies (*E*_ads_) on the isolated metal atoms follow the trend (211) > (100) ≈ (111), with similar adsorption behavior on the (111) and (100) surfaces despite differences in atomic coordination. This indicates that reactivity trends of dopants in one facet can inform the prediction of other facets, streamlining the discovery of new SAA catalysts. On the other hand, switching the dopant substantially influences the *E*_ads_ of the SAAs. For instance, Pt- and Pd-doped SAAs exhibit weaker CO binding and hence higher CO tolerance, whereas Ir- and Rh-doped systems bind CO more strongly compared to the pure dopant metal surface, suggesting enhanced reactivity but greater susceptibility to poisoning. Consequently, the strong cross-facet correlations and consistent adsorption behavior also highlight the structural insensitivity of SAAs, where reactions occurring directly at the dopant site are largely decoupled from variations in the host surface structure. However, if spillover of the adsorbate onto the host metal occurs, the reaction can be structure sensitive because it proceeds on the host facet. In the absence of spillover, SAAs thus can circumvent the structure sensitivity that often complicates catalyst design, further underscoring the predictability of their reactivity.

When using CO RAIRS to probe the structure of surface alloys, caution is needed when working with elements that bind CO strongly. For example, Wang et al. investigated CO adsorption on the RhCu(100) SAA, where they observed an IR peak at 1926 cm^–1^ when CO was adsorbed at 180 K (Figure 10a).^131^ Upon increasing the temperature, a new IR feature appeared at 1978 cm^–1^, accompanied by a decrease in the intensity of the 1926 cm^–1^ peak (Figure 10a). By 260 K, only the higher wavenumber peak remained (Figure 10a). The CO TPD profile showed two desorption features, at 265 and 440 K. STM imaging at 5 K revealed elliptical features on the Rh atom sites (Figure 10 **b,c**), which were assigned to a dicarbonyl species, consistent with the IR and TPD results that showed that once half of the CO desorbed, the IR peak shifted from a frequency consistent with a dicarbonyl to one expected for a single CO on Rh. Similar dicarbonyl features have been reported for Rh single atoms supported, e.g., on oxides, albeit the selection rules for dicarbonyls for dopants on metal surfaces dictate that only the symmetric vibration can be observed.^132,133^ Wang et al. also investigated the RhCu(100) SAA with *in situ* RAIRS under 10^–6^ mbar CO at RT.^131^ An IR peak consistent with a dicarbonyl species (1926 cm^–1^) was observed, demonstrating that such species persist even at moderate CO pressures and temperatures much higher than those at which CO desorbs in UHV TPD experiments. This suggests that dicarbonyl intermediates could potentially play a larger role in reaction mechanisms than the UHV TPD studies alone might suggest. Another DFT study by Christiansen et al. predicts an increase in the differential binding strength for adsorption of up to the third CO molecule on V, Cr, and Mn dopants in Cu(100).^134^ In contrast, a decrease in differential binding strength with increasing CO coverage on the dopant is predicted for Fe, Co, and Ni, indicating a weakening of each CO bond. However, experimental data of these effects on SAAs are scarce, which points to new research directions. Beyond multicarbonyl adsorption at single-atom sites, coadsorption of different species on a single-atom site can influence the reactivity of SAA systems.

**Figure 10:**
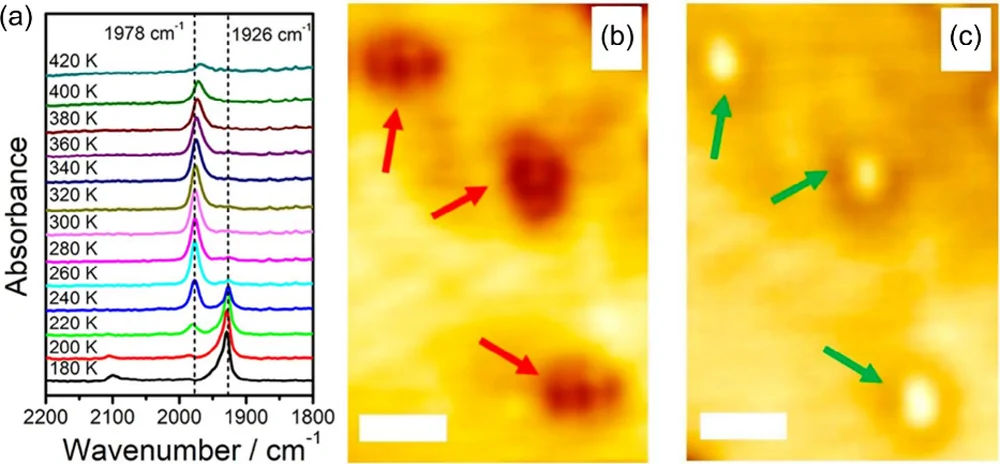
(a) Temperature-dependent RAIRS measurements on CO-saturated RhCu(100). (b) STM image acquired after CO adsorption followed by annealing to approximately 220 K, which populates Rh sites but not Cu sites. Red arrows indicate three dicarbonyl features, two oriented horizontally and one vertically. (c) STM image of the same area after applying – 3 V STM tip pulses to remove CO from the Rh sites. Green arrows mark the CO-free Rh atom sites that previously hosted the dicarbonyl species (both images 12 mV, 500 pA and 5 K). The scale bar corresponds to 1 nm in both panels. Reproduced with permission from ref.^131^ Copyright 2022 American Chemical Society.

#### 2.3.2 General Trends of Adsorption Behavior

Due to the well-defined electronic structure of the dopant, generalizing adsorption trends is key to increasing the predictability of SAA behavior. Most SAA work has focused on late transition metal dopants, but DFT studies that include dopants across the periodic table have shown that trends are qualitatively different between SAAs and larger ensembles or pure metals. Specifically, on pure metals adsorption tends to strengthen roughly monotonically moving toward the left on the periodic table.^122^ However, when varying the dopant in an SAA, a U-shape or W-shape is often observed.^59,107^ Dopant dimers and trimers show intermediate behavior between SAAs and pure metals.^107^

Using thousands of DFT calculations of the binding strength of adsorbates to SAAs, Réocreux and co-workers developed a simple 10-electron count rule for predicting the binding strength of adsorbates to dopant atoms.^59^ This rule is intuitive from a molecular orbital perspective in that optimal binding occurs when the adsorbate’s electrons saturate the dopant’s d orbitals. The resulting trends show that species covalently bound to SAAs with *4d* and *5d* dopants follow the 10-electron rule with a single extremum as seen in Figure 11a. As an example, N (with three bonding electrons) binds to Tc (with seven d electrons) as seen in Figure 11. Some care is needed to properly identify the number of bonding electrons for p-block elements, rather than simply counting the total number of electrons. The n_σ_ orbital, representing a lone pair on the adsorbate, remains populated across the period and is not involved in the d-orbital saturation or bonding (Figure 11b). When this lone pair is excluded, a universal 10-electron count rule applies. Extending this principle to a molecular adsorbate, N_2_ binds most strongly to *4d* and *5d* dopants with a d^*8*^ configuration, such as Ru and Os, through interaction with its lone pair (Figure 11c). However, not all adsorbates follow this rule perfectly. Molecules with highly polarized, closed-shell bonds, such as H_2_O, NH_3_, halogens, or hydroxyl groups, were posited to interact primarily through electrostatics rather than covalent electron sharing. In these cases, the adsorbate’s electrons cannot readily reshuffle upon binding. For such species, the 10-electron count rule becomes less predictive, and the dopant’s atomic charge may provide a more reliable descriptor of binding strength. Additionally, recent work has shown that deviations from these simple electron-counting trends in SAAs can arise from spin-dependent electronic effects.^135^ In particular, the dopant spin moment and its change upon adsorption can weaken bonding by promoting early occupation of antibonding states and by altering dopant–host interactions, leading to the characteristic W-shaped adsorption trends observed for 3d dopants.

**Figure 11:**
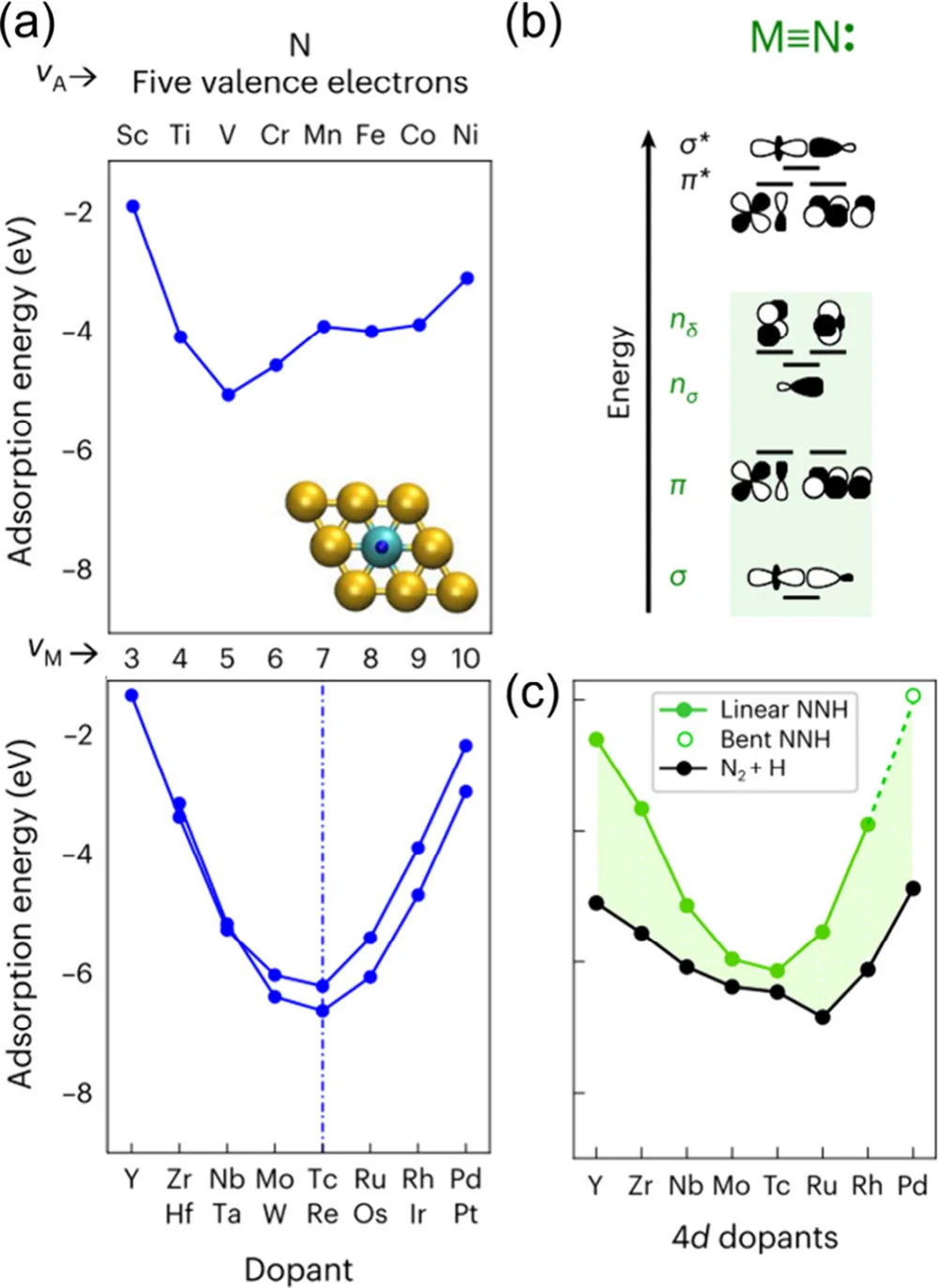
(a) Atomic N (blue) binding energies on 3d (top) and 4d/5d (bottom) TM dopants in Au(111). (b) Molecular orbital diagrams for MN, where M is the dopant. (c) Formation energy of N_2_ (black) and NNH (green) on Au based SAAs for 4d dopants. Adapted from reference.^59^ Copyright 2024 The Authors. Licensed under CC BY 4.0.

Building on these electron-counting concepts, a related single-atom-saturation framework predicts intermediate binding on SAAs by combining electronic saturation, represented by the d-electron occupancy of the guest atom, with geometric saturation of its coordination environment, while also accounting for the identity of the host and the adsorption configuration of the intermediate.^136^ By unifying electronic and geometric contributions within a single descriptor, this approach extends purely electron-count-based rules toward a more general basis for predicting binding across diverse host-dopant pairs and adsorbates. In a similar spirit, the degree of active-site isolation can itself be used as a descriptor, with a volcano-type relationship between isolation and selectivity providing a route to identify optimal single-site alloy compositions.^70^

Berger et al. performed a detailed theoretical study using DFT and KMC to investigate possible synergistic effects of coadsorbates in SAAs.^137^ They explored Ni, Cu, all the 4d transition metals and Ir and Pt doped atomically in Ag(111), Cu(111), and Cu(211) surfaces, with the dopant located at the step edge in Cu(211). CO, NO, and H atoms were modeled for both homo- and heteroadsorbate coadsorption cases. The results indicated that when the adsorbate coverages surpass the dopant site coverages, coadsorption becomes energetically favorable in nearly all cases because for example, the dicarbonyl is energetically preferred vs a monocarbonyl with the second CO on the host. Together these results suggest that coadsorption is a general characteristic of SAA catalysis.^137^ Furthermore, they investigated a model reaction between two different species, finding that in systems which favor coadsorption, only the reaction barrier needs to be overcome. In contrast, in systems without preferential coadsorption, an additional energy barrier associated with forming the reaction-ready state is observed. As a result, the catalytic activity in the coadsorption case may be higher, which has implications for the design of SAAs that perform e.g. heterocoupling.

Christiansen and co-workers systematically investigated multiple CO adsorption on 3d and 4d transition metal substitutional impurities in Cu(111) and Cu(100) surfaces using the BEEF-vdW functional, which explicitly includes dispersion interactions.^138^ Their calculations show that while the first CO binds strongly to most impurity atoms, the differential binding energy typically decreases with additional CO molecules, although Cr and Mn exhibit the opposite trend due to changes in unpaired d-electron count. Importantly, the dispersion interaction contributes significantly to the binding of the weakest-bound CO molecule, which is the species most likely to participate in the CO_2_ reduction reaction (CO_2_RR), and several 4d impurities (e.g., Nb, Mo, Rh, Pd) bind multiple CO molecules with energies close to that of pure Cu. For Mn, Fe, and Co, adsorption of four CO molecules pulls the impurity atom out of the surface lattice, suggesting a potential degradation pathway for single-atom alloy catalysts, while calculated CO stretching frequencies provide clear spectroscopic fingerprints for experimental detection of these multicarbonyl species.

This principle of localized electronic interactions extends to more complex adsorbates. DFT studies demonstrate that in the SAA regime, where electronic mixing between metals is minimal, the dilute component exhibits sharp, atom-like electronic states localized on individual dopant atoms. When these states align appropriately with specific molecular orbitals, selective hybridization occurs, enabling control over adsorption and reactivity according to molecular orbital theory. Spivey and Holewinski analyzed the adsorption energies of crotonaldehyde on TM dopants in Cu, Ag and Au(111) hosts.^139^ They identified two stable adsorption modes: one via the HOMO, which is primarily localized on the aldehyde group, and another via the HOMO–1, which is mainly localized on the alkene group. High-lying, nearly empty d-states of the dopant favor binding through the HOMO due to better energy alignment and stronger coupling. In contrast, low-lying, nearly filled *d*-states show a preference for interaction via the HOMO–1. This preference arises primarily from the occupation of antibonding states and stronger coupling to the HOMO, where Pauli repulsion is particularly unfavorable. Therefore, the early TM dopants favor the binding via the aldehyde group, whereas the later TMs prefer the binding via alkene group. Control over such interactions could be broadly applied to control chemo- or regioselectivity, or even selectivity within complex mixtures, provided the key molecular orbitals are spatially localized. However, adsorption represents only one step in a catalytic cycle, and full reaction pathways must be considered to understand overall selectivity. Reaction free-energy analyses for crotyl alcohol and butanal formation on Au, FeAu, and PtAu surfaces show that the preferred product pathway on the dopant site aligns with its initial adsorption geometry. While selectivity has been extensively studied for monometallic and traditional alloy systems, experimental validation in SAAs remains limited, one notable example being acrolein hydrogenation on PdAg, where dopant and host exhibit competing reactivity.^140^ A general design insight is that dopant sites should either complement or greatly outperform the host in intrinsic reactivity. Although surface segregation of the host may occur depending on the environment, SAAs still offer a powerful framework for achieving molecular-level selectivity control in heterogeneous catalysis.

#### 2.3.3 Spillover

A key principle underlying the high selectivity of SAA catalysts is the pronounced difference in reactivity between the dopant atom and the host metal. As described throughout this review, isolated dopant sites can exhibit chemical properties that are distinct from both their bulk-metal counterparts and the extended surface of the host metal. Moreover, the spillover of reactive intermediates from dopant to host and *vice versa* plays an important role in reaction mechanisms on SAAs.^141^

##### 2.3.3.1 Hydrogen Spillover

Hydrogenation reactions are a major class of industrially relevant reactions. Typically, the activation of dihydrogen is the first step and can be reaction rate limiting. Cu is a widely used hydrogenation catalyst as it is highly selective; however, its intrinsic ability to activate H_2_ is limited. An early study of SAAs investigated the influence of Pd dopants on hydrogen activation on Cu(111) and Au(111) in UHV.^45^ STM images of PdCu(111) containing 1% of a monolayer of Pd revealed that approximately 50% of the Pd atoms are located in surface sites, while the remaining 50% occupy subsurface positions. A similar distribution of surface and subsurface Pd species was observed for 1% PdAu(111). Both SAA surfaces were exposed to 10 L of H_2_. On the PdCu(111) surface, depressions corresponding to hydrogen atoms were observed in STM upon cooling to 7 K. These hydrogen atoms formed islands on the Cu host surface, indicating spillover from the isolated Pd atoms to the Cu host (Figure 12a). In contrast, no hydrogen islands were observed on Au(111). DFT calculations suggest that hydrogen activation is feasible on Pd atoms in both substrates. However, hydrogen spillover is thermodynamically hindered on PdAu(111) but possible on PdCu(111), explaining the experimentally observed differences in STM.

**Figure 12:**
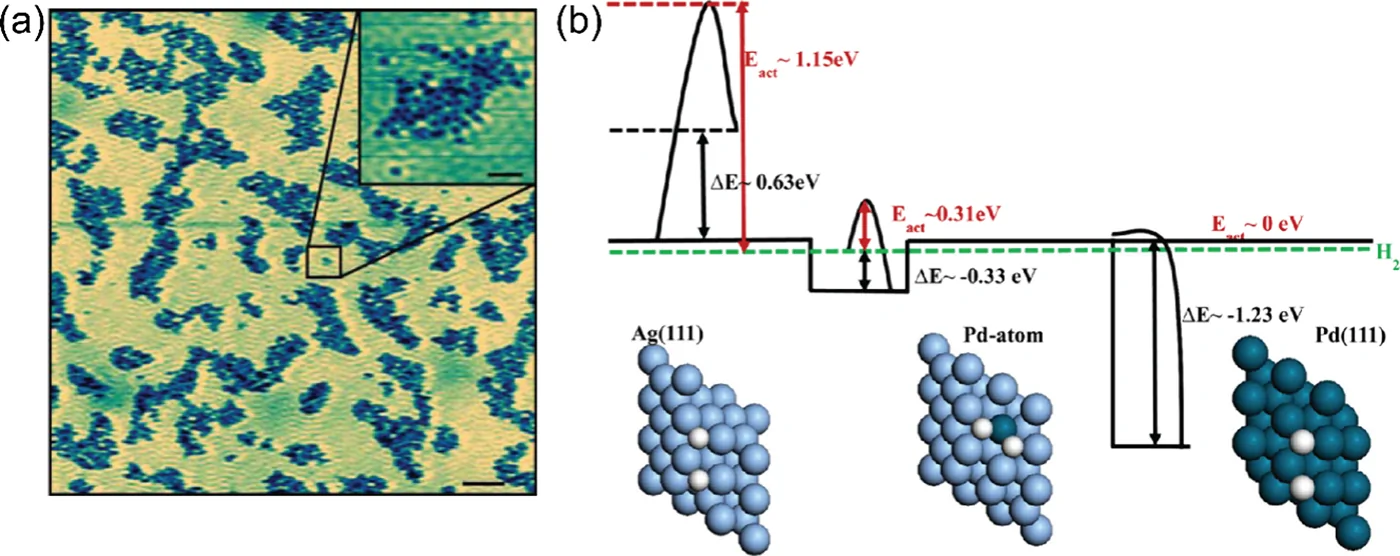
(a) STM image of islands of H atoms on PdCu(111) at 7 K (scale bar 15 nm, I = 10 pA, V_tip_ = −8 mV; Inset: scale bar 5 nm, I = 50 pA, Vtip = 20 mV). Reproduced with permission from ref.^45^ Copyright 2006 American Physical Society. (b) Potential energy diagram of dissociation of H_2_ on Ag(111), PdAg(111) and Pd(111) with activation energy *E*_act_ (red) and binding energy ΔE (black). H atoms are stable on PdAg SAA and Pd(111) surfaces but unstable on Ag(111) with respect to gas-phase H_2_. Reproduced with permission from ref.^36^ Copyright 2015 American Chemical Society.

Further experimental investigations have expanded this picture. Osada et al. examined H_2_ activation and H spillover on PdCu(111) at 80 K,^142–144^ while Lucci et al. studied H_2_ adsorption on PdAu(111) at 85 K.^145^ In the latter case, H_2_ desorption from the Pd sites was observed at 175 K, which is approximately 135 K lower than on pure Pd. Notably, when a H_2_-preexposed PdAu(111) surface was subsequently dosed with CO, a common catalyst poison, a new H_2_ desorption feature emerged at 110 K, accompanied by the disappearance of the 175 K feature. This behavior was attributed to CO-induced spillover of H from Pd dopant sites to the Au host surface, with the low-temperature feature corresponding to H_2_ desorption from Au.

For 0.08% of a monolayer PdAg(111), Muir et al. reported no evidence of hydrogen atoms spilling over onto the host after exposure to 50 L of H_2_ at 85 K using TPD.^127^ However, hydrogen desorption from Pd atoms alloyed at RT was likewise undetected, which the authors attributed to the extremely low Pd concentration and the corresponding sensitivity limits of the TPD experiment. Such low Pd coverages were necessary because, at higher Pd coverages, TPD and IR measurements revealed a high-temperature CO desorption feature and an IR band at 1958 cm^–1^, respectively, indicative of bridge-bound CO and thus Pd dimers. In contrast, DFT calculations by Aich et al. suggested that H spillover is feasible on PdAg(111).^36^ Specifically, they reported a decrease in the activation energy for H_2_ dissociation from 1.15 eV on pure Ag(111) to 0.31 eV on the PdAg(111) SAA, accompanied by a substantial weakening of atomic hydrogen binding from – 1.23 eV on Pd(111) to – 0.33 eV on PdAg(111). This combination of a lowered dissociation barrier and reduced hydrogen binding strength suggests that unlike the previous experiment, at the correct pressure, temperature and dopant coverage, hydrogen spillover may be energetically accessible on PdAg(111) (Figure 12b). The difference in spillover behavior for the three Pd SAAs (Cu(111), Ag(111), Au(111)) shows the importance of the choice of host metal for spillover.

Yoshinobu and co-workers used surface science analysis approaches to study H_2_ activation and spillover on PdCu(111) SAA model catalysts.^142^ Using a combination of IRRAS, TPD, XPS and DFT calculations, the number of H_2_ molecules that can adsorb at a Pd site prior to spillover was interrogated. They proposed that the first dissociated H_2_ molecule will be retained by the Pd site, while subsequent H_2_ dissociation events result in H spillover to Cu. The results provide a mechanistic picture of how SAA surfaces develop during initial exposure to H_2_ environments, and further suggest that, under hydrogenation reaction conditions, reactive dopants may retain some dissociated H, although direct connection to supported metal catalysts will be influenced by reactant chemical potentials and dopant location on the host (terraces, steps, etc.).

Hydrogen spillover has also been demonstrated for SAAs with different dopants. For example, single Pt atoms in Cu(111) show hydrogen activation and spillover onto the Cu surface at 85 K.^30^ The hydrogen recombination barrier from an isolated Pt site is lower than on pure Pt or Cu (>300 K), as evidenced by an H_2_ desorption temperature of 230 K in TPD. Gu and Lin investigated the mechanism of hydrogen spillover on the PtCu(111) system with MD calculations using a ML interatomic potential fit to DFT.^146^ They showed that Pt undergoes deactivation after the dissociative adsorption of H_2_ by the two H atoms adsorbed in its vicinity. Collisions of gas-phase H_2_ with these 3-fold adsorbed H atoms at Pt sites promote H spillover to the Cu host, which in turn frees up the Pt site and enables continuous H spillover on PtCu(111).

A broad theoretical study on hydrogen spillover was performed on nine Cu(111)-based SAAs doped with Fe, Co, Ni, Ru, Rh, Pd, Os, Ir, and Pt.^76^ The energy profiles for H_2_ dissociative adsorption and subsequent H atom spillover were calculated for all of these. Only the group 10 TMs (Ni, Pd, and Pt) exhibit lower activation barriers for H-atom diffusion than H_2_ dissociation, facilitating spillover (Figure 13). Therefore, H spillover is kinetically feasible on these systems. Fu and Luo explain the observed trend using the *d-*band model. Specifically, the interaction between the dopant atom and H_2_ must be strong enough to ensure a low activation barrier for H_2_ dissociation. However, a sufficiently weak H binding energy to the dopant site is required to facilitate spillover. The d-band center shifts downward in energy from group 8 to group 10, leading to a decrease in adsorbate binding energy and hence reducing the spillover energy, making the process favorable.

**Figure 13:**
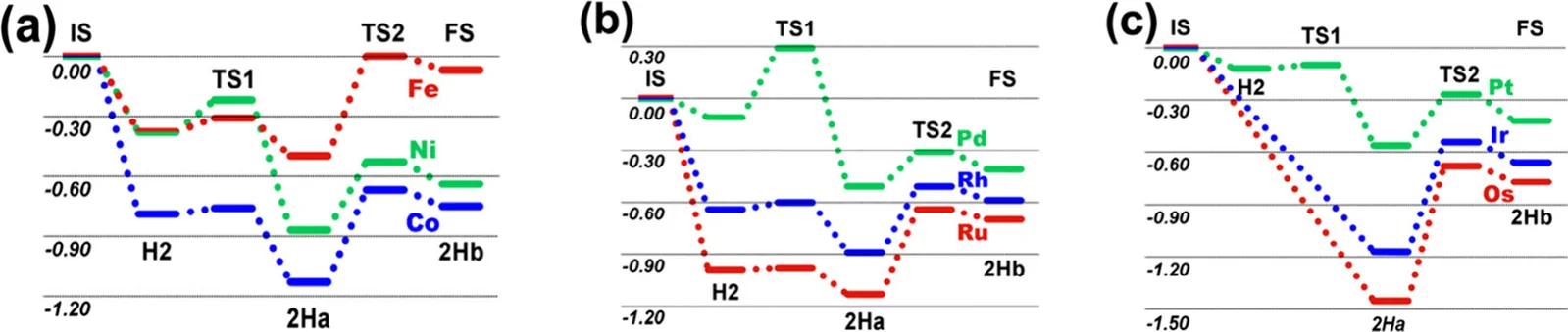
H_2_ dissociation and H-atom spillover energy profiles of Cu(111)-based SAA systems with dopants from the (a) fourth, (b) fifth and (c) sixth period of the periodic table. “IS” and “FS” are the initial and final state, and TS1 and TS2 denote the transition states of H_2_ dissociation and H-atom spillover. Adapted with permission from ref.^76^ Copyright 2013 American Chemical Society.

Interestingly, DFT studies suggest that SAAs can form dihydrogen and dihydride complexes, similar to some SACs, which do not form on pure metal surfaces. The potential importance or utility of these complexes has not been thoroughly investigated.^147^

##### 2.3.3.2 H Spillover and Hydrogenation Selectivity in SAAs

Hydrogen atom spillover can be exploited for selective hydrogenation, where H_2_ activation occurs on the dopant atoms and the activated hydrogen atoms spill over to the surrounding host surface, on which the hydrogenation reaction takes place. For example, the hydrogenation of 1,3-butadiene on PtCu(111) was explored using STM and TPD.^35^ For the 2% of a monolayer PtCu(111) SAA, the only observed hydrogenation product after exposure to 50 L of H_2_ and 0.1 L of butadiene was butene (Figure 14a). No butane was detected, which was attributed to the weak binding of butene on Cu, resulting in highly selective hydrogenation to butene. Experiments in which this reaction was performed with and without CO adsorbed at the Pt atom sites illustrated that Pt served as the H_2_ activation site, whereas the selective hydrogenation step occurred on Cu. When the Pt coverage was increased to 30% of a monolayer, there was a crossover from single Pt atoms to ensembles, and a lower selectivity toward butene was observed, mainly due to decomposition of 1,3-butadiene into hydrocarbon fragments, as evidenced by high-temperature H_2_ desorption at 420 K (Figure 14b). STM images showed only isolated Pt atoms at low (∼2% of a monolayer) Pt concentrations, whereas at higher concentrations linear chains of 2–10 Pt atoms were present. These Pt ensembles are capable of C–C and C–H bond cleavage, thereby forming hydrocarbon fragments and hence drastically reducing the selectivity to the preferred product butene.

**Figure 14:**
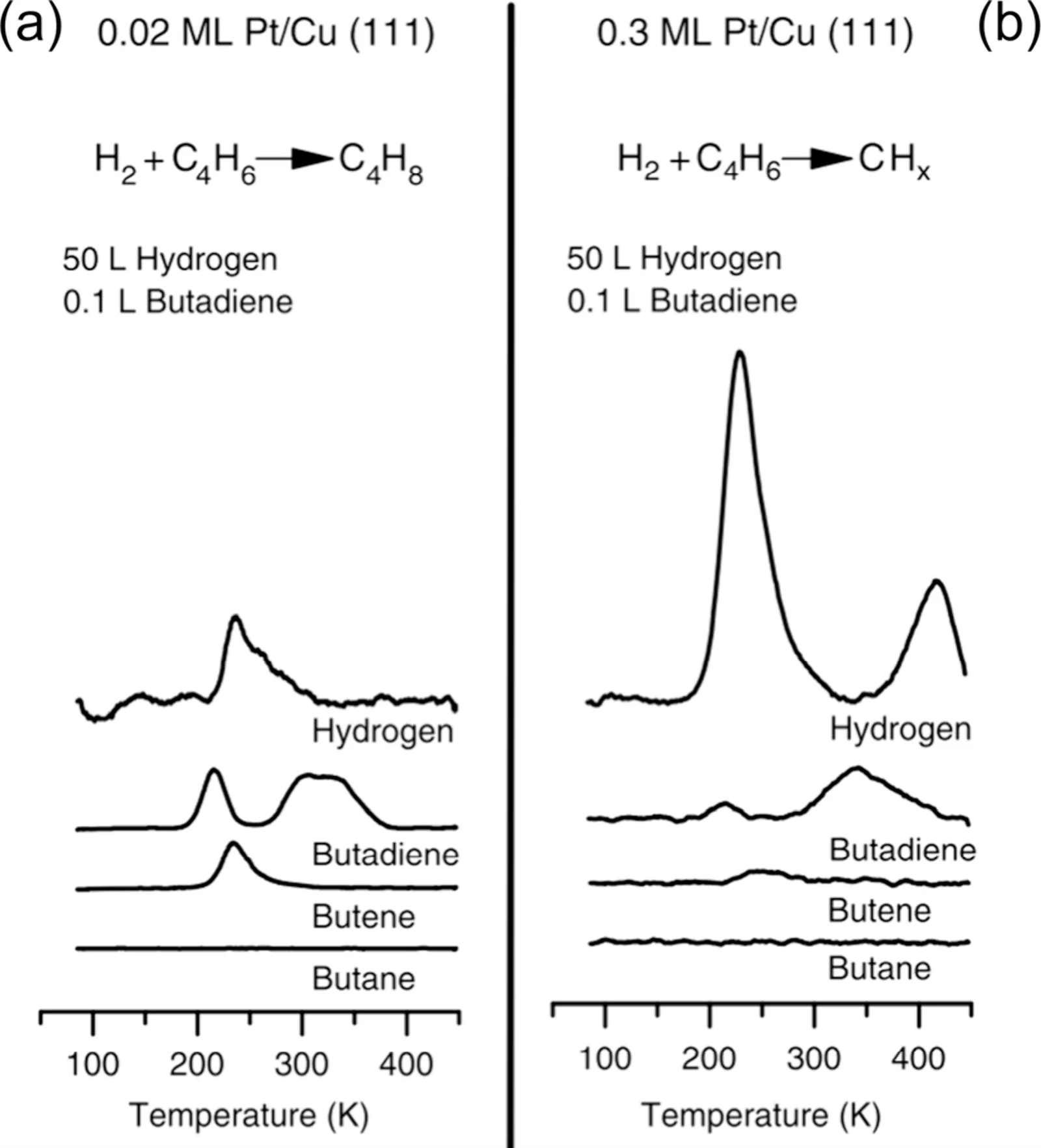
TPD from (a) 2% of a monolayer PtCu(111) and (b) 30% of a monolayer PtCu(111) after exposure to 50 L H_2_ and 0.1 L butadiene at 85 K. Adapted from ref.^35^ Copyright 2015 The Authors. Licensed under CC BY 4.0.

Lv et al. performed DFT studies of this system, comparing single Pt atoms and linear chains of 4 Pt atoms in Cu(111).^148^ They showed that the H_2_ activation and H spillover steps are similar in both systems and hence the kinetics of selective hydrogenation are similar for both systems. However, the activation energy for the dehydrogenation of 1,3-butadiene (CH_2_CHCHCH_2_ → CHCHCHCH_2_ + H), the first step in decomposition, is 0.36 eV higher for single Pt atoms compared to Pt chains, which reduces side products (hydrocarbon fragments) and hence increases selectivity for the SAA.^148^ Hossain et al. studied 1,3-butadiene hydrogenation on a 2.7% of a monolayer PdCu(111) surface under 0.1 Torr 1,3-butadiene and 10 Torr H_2_ at 280 K with RAIRS.^149^ 1-butene formation was 84% selective, with 2-butene and carbonaceous deposit as unwanted side products. Together, these studies suggest the increased hydrogenation selectivity of SAAs like PtCu and PdCu arises from hydrogen spillover and highlight this bifunctionality advantage of SAAs. Comparisons of SAAs with pure metal catalysts and conventional alloy systems with closer to a 1:1 ratio of the metals illustrate that isolated single atoms exhibit distinct reactivity, for example suppressing C–H and C–C bond cleavage, which enhances selectivity further. A study by Flytzani-Stephanopoulos and co-workers highlighted a different aspect of the high selectivity of SAAs.^150^ For the selective hydrogenation of 1-hexyne to 1-hexene on PdAu, surface science experiments and DFT showed that the reactant and product have similar adsorption strengths. The high selectivity arises from the reactant binding to the dopant site, where hydrogenation of the C atom bound to the dopant is strongly favored over hydrogenation at C atoms not bound to the dopant.

##### 2.3.3.3 Spillover of Other Surface Species

To exploit the bifunctionality of SAAs and the resulting high selectivity in reactions beyond hydrogenation, spillover of reactants and intermediates other than hydrogen must be investigated. Jalil et al. studied ethylene epoxidation on Ag, for which dioxygen activation can be rate-limiting.^60^ A theoretical screening of 22 dopants in Ag(111) identified Ni as a promising dopant, with the DFT calculations indicating that O_2_ dissociation on Ni atoms in Ag(111) has a low activation energy, while atomic oxygen binds relatively weakly to the Ni sites, making spillover likely. TPD experiments revealed that, after 500 L O_2_ exposure at 350 K, O_2_ desorption is observed from 1% of a monolayer Ni in Ag(111) at 520 K. The amount of desorbed O_2_ was 4× higher than the amount of Ni sites, indicating spillover of atomic oxygen onto the Ag substrate. The O_2_ desorption temperature from NiAg(111) is about 70 K lower than that from pure Ag, demonstrating that oxygen recombination occurs at the Ni sites after reverse spillover of oxygen atoms from the Ag host to the Ni dopant occurs. This low O_2_ desorption temperature also confirmed the predicted low energy of the transition state for dissociation/association. This is analogous to the case for H_2_ dissociation, spillover and recombination on PtCu and PdCu SAAs.

Another group of important reactants in heterogeneous catalysis comprises small organic molecules such as alkanes or alcohols. Wang et al. studied ethanol dehydrogenation on the 1% PtCu(111) SAA and compared it with Cu(111).^151^ They observed a 6-fold increase in activity on the SAA relative to the clean Cu surface and showed that the O–H bond of ethanol is activated at Pt sites at 160 K, followed by ethoxy spillover. The C–H bond is subsequently activated on the Cu(111) substrate at 310 K, leading to 100% selective acetaldehyde formation. Five K STM tip voltage pulse experiments were used to move ethoxy species and demonstrate they were bound to the Cu host rather than to the Pt single atoms, further supporting ethoxy spillover (Figure 15). In contrast, Molina et al. were unable to detect ethoxy species concentrations higher than the Pt coverage using RAIRS, which would exclude ethoxy spillover.^152^ They demonstrated that a sputtered Cu(111) surface was also capable of forming ethoxy species, thus attributing ethoxy formation to Cu defects rather than to Pt sites. These different results for similar SAA systems show that more work is needed to substantiate conclusions.

**Figure 15:**
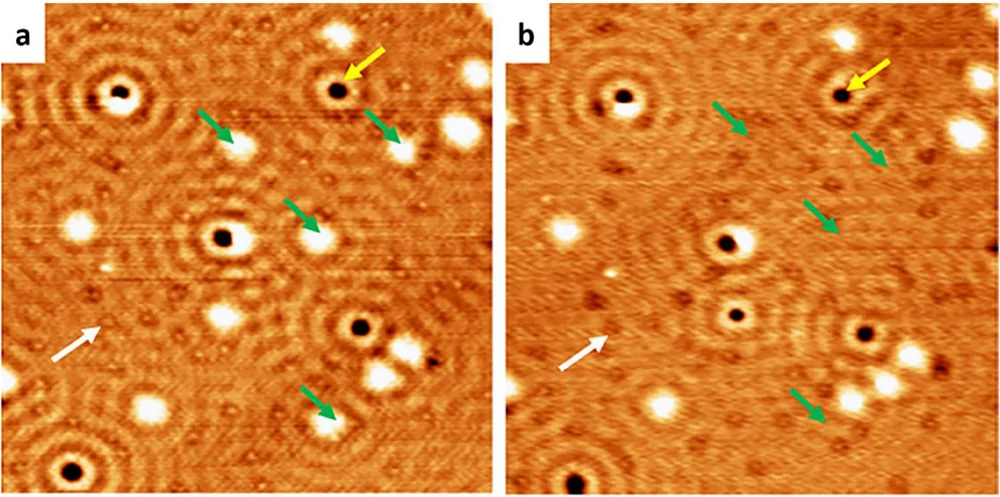
STM images of PtCu(111) after 0.5 L ethanol exposure at 5 K and annealing to 160 K (10–30 mV, 30 pA). (a) before and (b) after STM tip manipulation, in which the ethoxy species (green arrow) are desorbed. Adapted with permission from ref.^151^ Copyright 2018 Springer Nature.

Gu et al. investigated CH_3_ and H spillover from Rh single sites to Cu(111) using molecular dynamics, considering both surface phonons and electron–hole pairs,^153^ with a high-dimensional potential energy surface machine-learned from DFT data. Their calculations indicated that spillover of *H and *CH_3_ surface species is possible at ambient pressure. They also demonstrated that collisions of gas-phase molecules with these surface species facilitate spillover, with the heavier *CH_3_ species acquiring energy more efficiently and therefore exhibiting a higher spillover probability. Increasing the temperature (500 K) makes *H and *CH_3_ recombination and subsequent methane desorption more likely.

To increase predictability in SAA systems without high computational costs, generalization of the observed spillover behavior is essential. Réocreux et al. used DFT to identify a simple descriptor to predict the spillover energy.^32^ They investigated adsorbates (H, CO, OH_*x*_, NH_*y*_, and CH_*z*_) on Ni, Rh, Pd and Pt in Cu, Ag and Au(111), giving 12 different SAAs and corresponding spillover energies. The spillover energy is defined as the energy difference of either the formation energy or adsorption energy on host and dopant sites. Three terms constituting the interaction between the host/dopants and the adsorbate were considered: electrostatic, dispersion and covalent interactions. It was shown that dispersion interactions do not make a significant contribution by comparing different exchange–correlation functionals with strong and weak treatments of dispersion and demonstrating that the resulting differences are negligible. They found that for saturated species the charge of the dopant is a good descriptor. For unsaturated species, the spillover energy of C was found to best describe their behavior. Combining these two in a linear surrogate model led to prediction of DFT calculated spillover energies with an error of 0.04 eV. Hence, the dopant charge and the spillover energy of C are well suited to capture the contribution of electrostatic and covalent interactions, respectively.

Reverse spillover refers to the process in which reactants migrate from the host surface to the dopant. Hannagan et al. investigated whether the spillover energy could serve as a descriptor for improving selective formic acid decomposition to CO_2_ and H_2_ while inhibiting CO.^154^ On PtCu(111), no increase in activation was found relative to Cu, whereas NiCu(111) shows increased reactivity. Formate is more stable on Ni than on the Cu host, making reverse spillover energetically favorable, but this is not the case for Pt. Since C–H activation is the rate limiting step, the propensity for reverse spillover may explain why the NiCu(111) SAA shows a higher reactivity vs PtCu.^154^ A flow reactor study showed, however, that NiCu NPs on SiO_2_ are not significantly more reactive than Cu NPs on the same support under steady-state conditions, which contrasted with the model study results. The authors ascribed this observation to the high surface coverage in a flow reactor, where adsorbate-stabilized C–H activation may weaken the influence of Ni.

##### 2.3.3.4 The Molecular Cork Effect

A useful strategy to further investigate spillover phenomena is the so-called *molecular cork effect*. As shown previously, both the activation and recombination (*via* diffusion from the host back to the dopant) of, for example, diatomic reactants often take place on dopant sites. Blocking the dopant sites with strongly bound and stable molecules (e.g. CO) after exposure to the spillover species (e.g., H_2_), suppresses the low barrier pathway and increases the overall recombination energy. This effect was demonstrated for PdCu(111).^155^ Hydrogen was exposed to the substrate, where spillover occurred. After the spillover of atomic H, the surface was exposed to CO, which binds strongly to Pd sites and thus blocks them. TPD measurements showed an increase in the H_2_ desorption temperature by approximately 50 K after CO exposure compared to the unexposed surface (Figure 16), with desorption initiating upon CO desorption from the Pd sites. This indicates that Pd sites are no longer accessible for H recombination when blocked by CO.^155^ Such blocking can impact on-surface reactions by trapping less-stable H atoms. Simpson investigated another possible application of this concept for hydrogen storage on surfaces using blocking groups with higher desorption temperatures than CO. The functionalization of N-heterocyclic carbenes as molecular corks on Pd- and Pt-doped Cu(111) surfaces was studied using DFT.^156^ Electron-withdrawing groups were found to weaken the N-heterocyclic carbene-surface bond, whereas electron-donating groups strengthened it, demonstrating that carbenes enable tunable desorption temperatures.

**Figure 16:**
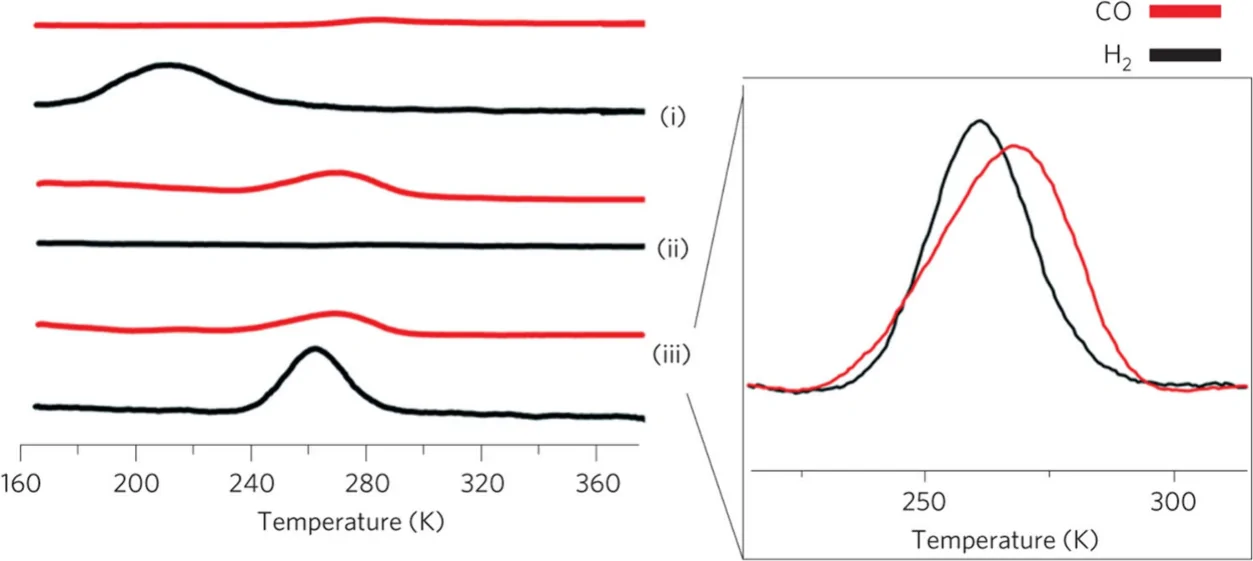
CO (red) and H_2_ (black) TPD traces after (i) hydrogen exposure, (ii) CO followed by hydrogen exposure and (iii) hydrogen followed by CO exposure of 1% of a monolayer PdCu(111). For (iii) the H_2_ desorption temperature shifts from 210 to 260 K due to CO blocking the Pd single-atom sites. Adapted from ref.^155^ Copyright 2013 Springer Nature.

#### 2.3.4 Adsorbate-Induced Structural Changes in SAAs

Despite metal surfaces being traditionally thought of as rigid, over the last ∼ 30 years many observations of adsorbate-induced surface restructuring have been reported. In bimetallic systems, the surface composition can dynamically respond to changes in the surrounding chemical environment, with adsorbates sometimes altering segregation tendencies between the constituent metals. For example, adsorbates have the capability of stabilizing one metal at the surface, which would otherwise preferentially reside in the bulk. A recent study on Ni–Ag SAAs provides an example of such oxygen-induced dopant segregation and stabilization.^60^ In the context of ethylene epoxidation, the incorporation of trace amounts of Ni into Ag was found to increase selectivity by ∼ 25%. Surface science experiments revealed that surface Ni atoms facilitate O_2_ activation while avoiding overbinding of atomic oxygen. However, TPD studies showed that after heating Ag(111) to ∼ 700 K, the Ni atoms moved to the subsurface. Upon further O_2_ exposure, Ni was found to segregate back to the surface, consistent with its high oxophilicity as compared to Ag (Figure 17a). This segregation behavior demonstrates how adsorbate interactions can dynamically and reversibly control dopant location, with oxygen specifically driving surface enrichment of Ni in Ag catalysts. *Ab initio* studies by Dhouib and Guesmi on group 10 metals (Ni, Pd, Pt) in Au(111) predicted that this behavior is general.^157^ O_2_ or atomic O reverses the segregation tendencies of Ni, Pd and Pt, driving dopants that are buried in the bulk to emerge at the surface due to their strong affinity for oxygen.

**Figure 17:**
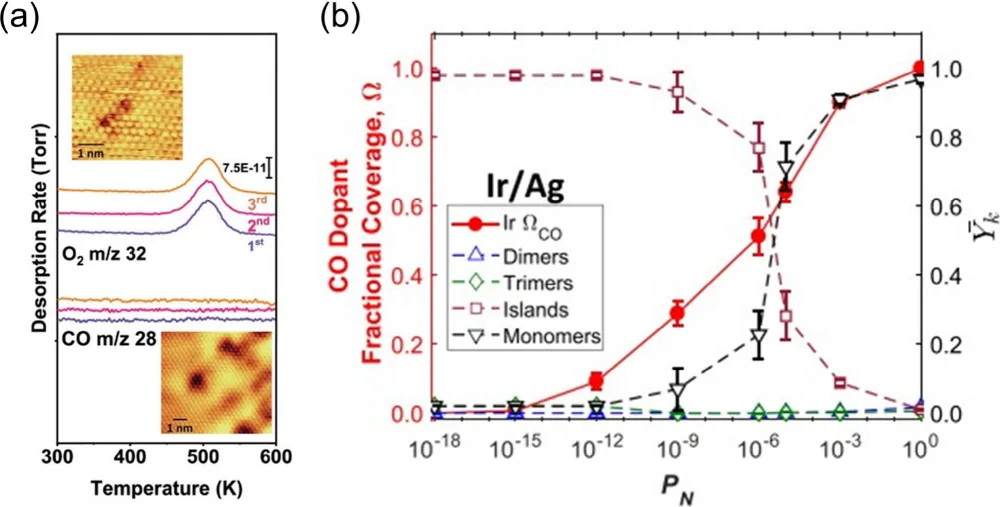
Adsorbate effect on aggregation and segregation. (a) Shown are sequential TPD experiments consisting of an initial 500 L O_2_ dose at 350 K followed by TPD, and subsequently a 0.5 L CO dose at 90 K followed by TPD. The insets present 13 K STM images of Ni incorporated beneath the Ag(111) surface immediately after Ni deposition (lower inset; imaging conditions: 0.2 nA, 100 mV) and of Ni present at the Ag(111) surface after cooling from 473 K in O_2_ (upper inset; 0.3 nA, 50 mV). Adapted with permission from ref.^60^ Copyright 2025 AAAS. (b) Fractions of surface species on IrAg(111) at 350 K as a function of normalized pressure. The left *y*-axis (red) corresponds to the fractional coverage of the CO on the dopant, while the right *y*-axis (black) shows the fractions of the various surface species. Adapted with permission from ref.^158^ Copyright 2019 American Chemical Society.

Carbon monoxide is another adsorbate that can drive reversible dopant redistribution in bimetallic systems. For example, in PdAu(111) alloys, CO exposure and mild annealing induce dynamic restructuring that directly impacts catalytic behavior.^159^ Time-resolved temperature-programmed infrared reflection–absorption spectroscopy (IRRAS), combined with *ab initio* thermodynamics and machine-learning molecular dynamics, revealed that upon vacuum annealing, surface Pd islands become encapsulated by Au and partially dissolve into the subsurface, leaving isolated Pd monomers at the surface. Subsequent exposure to 0.1 mbar CO drives these Pd atoms back to the surface, while further heating to 473 K causes complete redissolution into the subsurface. These results demonstrate how CO can both probe and control atomic rearrangements at alloy surfaces. This CO-induced reversible segregation of Pd exemplifies how adsorbates can dynamically tune dopant distribution under reaction conditions, stabilizing active sites or regenerating surface composition in fluxional bimetallic catalysts. Further theoretical analyses show that such restructuring trends are not unique to this system. CO adsorption often promotes dopant segregation to the surface due to stronger binding. At high coverage (>0.5 monolayer), CO can enhance dopant dispersion because strong repulsive interactions between CO molecules at high surface coverages drive dopant atoms to disperse. At lower coverages, CO can favor aggregation if it prefers to coordinate to multiple dopant atoms. These results demonstrate that CO-induced structural dynamics can be exploited to tune ensemble effects and catalytic performance in PGM–coinage metal SAAs.^86^

In a related study by Finzel and Christopher, the influence of environmental conditions on the coordination environment of Pt in ∼ 5 nm diameter Ag host particles was examined using DFT calculations, statistical mechanics, Fourier-transform infrared spectroscopy (FTIR) and X-ray absorption spectroscopy (XAS).^92^ For dilute Pt concentrations (Ag:Pt molar ratio >50), isolated Pt atoms preferred to reside in the Ag bulk, while annealing at 523 K in CO caused Pt to move to the surface. Thermodynamic phase diagrams along with XAS and FTIR measurements suggested that in addition to CO being bound to SAA structures, structures can also form where surface Pt atoms are bound to one or more subsurface Pt atoms. While this conclusion was only indirectly supported *via* spectroscopy, the result is consistent with the increasing body of literature demonstrating that adsorbates can be used to manipulate the structure and function of SAAs.

Another example of when the apparent reactivity of an SAA can depend sensitively on reaction conditions, particularly pressure is illustrated by CO adsorption and activation on NiCu(100) SAAs, where markedly different behavior is observed between UHV and millibar regimes.^160^ Under UHV conditions, isolated Ni atoms embedded in Cu(100) significantly strengthen CO adsorption without inducing dissociation. In contrast, exposure to CO at elevated pressures leads to substantial surface accumulation of carbon and oxygen, indicating spontaneous CO dissociation and disproportionation. DFT calculations rationalize this pressure dependence by showing that these reaction pathways are thermodynamically inaccessible at low CO coverages but become viable at higher coverages due to increased adsorbate–adsorbate repulsion, which raises the energy of the molecularly adsorbed state.

Designing alloy catalysts that remain stable and active under reaction conditions requires understanding how adsorbates and the local chemical environment influence surface segregation. Recent developments have combined *ab initio* theory with machine learning to accelerate this process. A data-efficient model using an element-centered fingerprint (ECFP) was developed to predict surface energies across diverse alloy compositions with high accuracy, enabling rapid estimation of segregation trends in hundreds of SAAs under both clean and CO-covered conditions.^161^ Extending a similar type of screening to more realistic colloidal systems, DFT and neural-network analyses revealed that common ligands such as amines and thiols can significantly alter dopant segregation energies, often reducing the thermodynamic driving force for segregation compared to bare surfaces and exhibiting a strong dependence on ligand binding configuration and strength.^162^ Building on this, recent studies have highlighted the critical role of these surface-bound ligands in dictating the aggregation behavior of SAAs.^84^ Systematic investigations of group 10 (Ni, Pd, Pt) and group 11 (Ag, Au, Cu) metal combinations across (111) and (100) facets reveal that ligand coordination can either stabilize atomic dispersion or promote dopant clustering, depending on the host–dopant chemistry. In the absence of ligands, isolated single-atom configurations are generally favored over dimer or trimer formation, although facet-dependent exceptions exist. Upon ligand adsorption, however, clear trends emerge: Ni-, Pd-, and Pt-based hosts tend to maintain stable SAAs, whereas Ag-, Au-, and Cu-based hosts doped with Ni, Pd, or Pt exhibit a pronounced tendency toward aggregation. Together, these findings illustrate that alloys are not static but dynamically respond to changes in their surrounding chemical environment. Given the large number of potential SAA combinations and adsorbates, machine learning is useful in efficiently predicting these trends, with DFT providing more accurate and detailed mechanistic insight into adsorbate- and ligand-induced segregation that define catalyst structure and function under operating conditions.

DFT studies have broadly shown that CO can strongly influence dopant dispersion and aggregation in SAAs,^80,158^ in agreement with the discussion above. Under vacuum conditions, most PGM-dopant SAA systems feature negative mixing enthalpies and thus thermodynamically favor isolated dopant atoms, with the notable exception of IrAg(111), where dopant clustering is preferred. DFT has shown that the introduction of CO fundamentally alters these trends in a system-dependent manner: on PdAu(111), PdAg(111), IrAg(111), and NiCu(111), the size and morphology of dopant ensembles can be reversibly tuned by adjusting the CO partial pressure, whereas PtCu(111) and RhCu(111) remain largely insensitive to CO and retain atomically dispersed dopants across a wide range of CO coverages. In the case of IrAg(111), low CO coverage stabilizes Ir islands, while increasing CO progressively destabilizes these clusters and drives a sharp transition to an SAA regime at high coverage (Figure 17b). This behavior can be rationalized by the stronger energetic preference of CO for atop adsorption on Ir clusters relative to hollow-site binding. This adsorbate-controlled restructuring has direct catalytic consequences: in PdAu bimetallic catalysts, combined theory and experiment demonstrate that a set CO surface coverage and low temperature stabilize isolated Pd atoms, promoting a highly selective SAA phase for selective ethanol dehydrogenation that favors C–H activation while suppressing C–C scission.^57^ At elevated temperatures and lower CO coverage, isolated Pd atoms aggregate into clusters, leading to nonselective decomposition pathways and byproduct formation.

These studies establish CO as a powerful probe and control molecule for dopant restructuring. Such adsorbate-induced aggregation phenomena are not limited to CO and can also emerge under reactive hydrocarbon environments. For example, a separate DFT study examined how dopant identity influences aggregation and selectivity of Cu-based SAAs during acetylene hydrogenation,^163^ focusing on Rh, Ni, Pt, and Pd dopants. Although PdCu and PtCu SAAs are stable in vacuum and remain structurally intact under reaction conditions, RhCu and NiCu SAAs become thermodynamically unstable upon acetylene adsorption, leading to dopant aggregation in the surface. This adsorbate-induced restructuring strongly impacts catalytic behavior: PdCu and PtCu favor ethylene desorption, enabling the desired selective semihydrogenation of acetylene, whereas RhCu and NiCu promote further hydrogenation due to stronger ethylene binding and lower barriers for full hydrogenation. These calculations illustrate how different reaction intermediates preferentially bind to different surface ensembles, providing a mechanistic explanation for dopant aggregation under reaction conditions. Overall, this study demonstrates that while Pt and Pd remain highly selective by preserving isolated active sites, Rh and Ni tend to aggregate during catalysis, leading to loss of selectivity and altered reaction pathways. Furthermore, a theoretical investigation combining DFT and microkinetic modeling examined the stability and reactivity of Pt-doped Cu(111) during butadiene hydrogenation,^164^ with particular attention to dopant aggregation under reaction conditions. Adsorption of butadiene is predicted to thermodynamically drive the formation of Pt dimers on the surface. These aggregated Pt_2_ sites are less active for C_4_H_6_ hydrogenation than isolated Pt atoms, indicating that dopant aggregation diminishes catalytic performance.

Frenkel and co-workers showed that PdAu dilute alloy nanoparticles restructure reversibly under different H_2_ or O_2_ pretreatments and continue to evolve under H_2_/D_2_ reaction conditions, as revealed by HD exchange kinetics.^165^ O_2_ pretreatment biased the surface toward larger Pd–Pd ensembles because Pd is more oxophilic than Au, so oxidation stabilized Pd rich surface domains and pulled Pd toward the surface, increasing Pd–Pd proximity. In contrast, H_2_ pretreatment favored more dilute Pd distributions within the Au host because reduction removed Pd–O stabilization and promoted Pd dissolution and mixing into Au, which stabilized isolated Pd through dilution and suppressed contiguous Pd ensembles. These pretreatment-dependent ensembles produced distinct Arrhenius parameters and, for the O_2_-treated state, a pronounced heating–cooling hysteresis, implying that the active ensemble population dynamically restructures during temperature ramps in H_2_/D_2_.

In studies of bimetallic catalysts, a variety of synthetic routes have been developed to obtain model catalyst systems tailored to specific target reactions, and SAA synthesis in particular has typically relied on optimizing the preparation conditions based on the properties of the two constituent metals. In contrast, Deshlahra and co-workers showed that, without any alloy synthesis step, the reaction conditions for vinyl acetate (VA) synthesis themselves restructure a Pd–Cu physical mixture into a PdCu SAA, exemplifying reaction-driven SAA formation. Specifically, they showed that when 1 wt % Pd/SiO_2_ and 5 wt % Cu/SiO_2_ were physically mixed so as to be in contact (0.03Pd + Cu), exposure to VA synthesis conditions (40 kPa C_2_H_4_, 10 kPa CH_3_COOH, 5 kPa O_2_, 160 °C) caused Pd to migrate onto the Cu particles and restructure into a PdCu SAA (Figure 18). At the beginning of the VA reaction, the 0.03Pd + Cu sample exhibited VA formation rate and selectivity close to 0, but within 1 h it reached a VA formation rate comparable to that of the prealloyed PdCu/SiO_2_ catalyst and showed an almost identical dependence on O_2_ pressure, indicating that the two catalysts converged to the same active structure under reaction conditions. This restructuring is explained by oxidation of metallic Pd and Cu under VA conditions to form Pd and Cu diacetate species, particularly mixed PdCu diacetates, which act as mobile organometallic intermediates that deliver Pd to Cu particles along the SiO_2_ surface. In the subsequent H_2_ reduction step, these Pd species were reduced and incorporated into the Cu lattice, giving rise to the PdCu SAA structure.^166^

**Figure 18:**
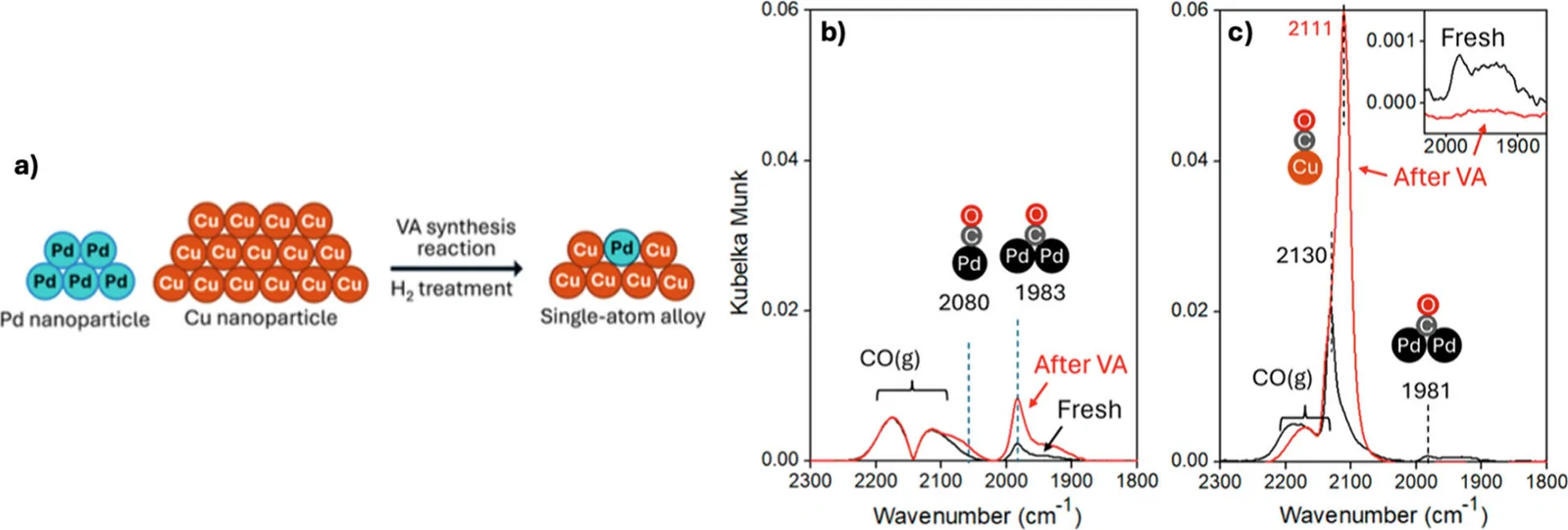
a) Scheme of restructuring of physical mixture sample *via* VA treatment. CO-DRIFTS spectra in 1% CO at 150 °C before and after VA treatment for b) 1 wt % Pd/SiO_2_ and c) 4.2 wt % 0.03Pd+Cu (physical mixture) before and after VA treatment (40, 10, 5 kPa C_2_H_4_, CH_3_COOH, O_2_, 160 °C, 2 h). Samples were treated in H_2_ at 300 °C prior to measurements. Adapted with permission from ref.^166^ Copyright 2024 American Chemical Society.

### 2.4 Breaking Scaling Relationships for Active and Selective Chemical Reactions

Scaling relations provide a unifying framework for linking adsorption energetics of chemical intermediates across different catalyst surfaces. Early DFT studies demonstrated that the adsorption energies of partially hydrogenated species (e.g., CH_*x*_, NH_*x*_, OH_*x*_, SH_*x*_) scale linearly with the adsorption energy of the corresponding central atom (C, N, O, or S), with proportionality constants determined primarily by the degree of hydrogenation rather than the specific metal surface.^167^ This insight enables reaction energies for many hydrogenation and dehydrogenation steps to be estimated from a small set of reference adsorption energies. Building on these relationships, scaling relations were incorporated into methods for constructing full reaction energy diagrams from minimal data for a single late transition metal.^168^ By utilizing the scaling relations between adsorption energies, reaction free energies for complex catalytic processes, such as methanation and ammonia synthesis, could be estimated across multiple transition metals based on calculations for a single reference surface.

The conceptual foundation of scaling relations in catalysis is closely linked to the Sabatier principle, which states that optimal catalytic activity arises when key reaction intermediates bind neither too strongly nor too weakly to the surface.^169^ This balance naturally leads to volcano-type activity trends, which can be derived for simple reactions by combining the Bro̷nsted–Evans–Polanyi (BEP) relationship, linking activation energies to reaction energies, with elementary kinetic models. A concrete manifestation of this framework is found in ammonia synthesis,^170^ where the BEP relationship provides a quantitative bridge between nitrogen adsorption strength and the barrier for N_2_ dissociation. When coupled with microkinetic modeling, this approach predicts a volcano-shaped dependence of catalytic activity on the N binding energy, with Fe and Ru located near the maximum. The volcano behavior reflects an intrinsic binding energy and activity trade-off governed by scaling relations: sufficiently strong N–metal bonding is required to lower the dissociation barrier, while weaker bonding is needed to avoid excessive surface poisoning by adsorbed N (Figure 19a).^171^ Under industrially relevant conditions, Ru and Os lie closest to the maximum, although economic constraints favor Fe as the most practical catalyst despite its slightly reduced intrinsic activity.^171^ Together, these examples illustrate how scaling relations and BEP-based models can enable efficient catalyst screening.

**Figure 19:**
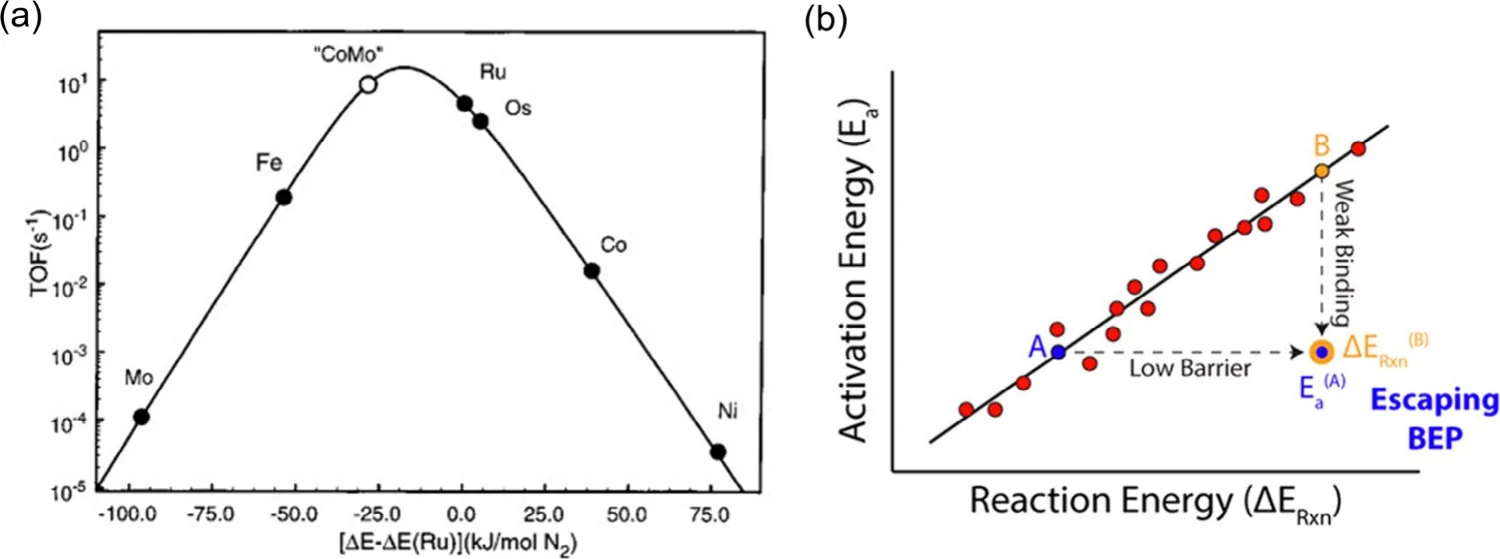
(a) Dependence of calculated ammonia synthesis turnover frequencies on N adsorption energy. Reproduced with permission from reference.^171^ Copyright 2001 American Chemical Society. (b) Diagram illustrating the BEP relationship between activation energy and reaction energy for chemical conversion, highlighting a favorable deviation from linearity that lowers the activation barrier at more positive reaction energies. Adapted with permission from ref.^49^ Copyright 2018 American Chemical Society

As described, scaling relations on transition-metal surfaces often impose an intrinsic trade-off between activity and selectivity, such that improvements in conversion are frequently accompanied by enhanced side reactions that lower selectivity.^68^ Excitingly, SAAs provide a route to circumvent these scaling relationships by spatially decoupling elementary reaction steps across chemically distinct active sites. Indeed, it has been explicitly shown with DFT calculations that predicted rates can exceed the volcano plot maximum on SAAs.^172^ In propane dehydrogenation, PtCu SAAs demonstrate this decoupling by combining high propane activation efficiency with suppressed further dehydrogenation that leads to coking, offering markedly improved selectivity at higher conversion.^68^ DFT reveals that this behavior arises from a breakdown of conventional Pt–metal alloy scaling relationships: while propane activation *via* C–H bond cleavage remains energetically accessible, propylene desorption is favored over further dehydrogenation due to weak binding, yielding an energetic separation between adsorption barriers and reaction energies.^68^ More generally, scaling relations can break when reactant dissociation occurs at a highly reactive atom while after spillover reaction intermediates bind preferentially to surrounding, less reactive host atoms, invalidating BEP correlations that assume a single dominant adsorption and reaction site (Figure 19b).^49^ This principle is exemplified by H_2_ dissociation on PtCu SAAs, where the transition state is localized on the isolated Pt atom, but the dissociated H atoms bind in mixed Pt–Cu 3-fold hollow sites, resulting in weaker H atom adsorption than pure Pt surfaces that enables spillover to Cu sites.^173^ As a result, BEP relations derived for pure metals often no longer apply to SAAs, which can escape linear scaling constraints through spatial separation of transition-state and final-state energetics. Scaling relations between intermediates can also be broken on SAAs when those intermediates occupy different sites. For example, CH_3_ often prefers top-site adsorption, and thus is calculated to be quite stable on a PtAu SAA where it binds to the Pt atom, while C prefers hollow-site adsorption, and thus is destabilized due to its binding to two Au atoms.^118^ Early TM dopants can cause particularly large deviations in scaling.^117,118,172^

The possibility of breaking scaling relationships has motivated significant work in developing and applying new computational design strategies in these cases, as the computationally convenient volcano plot becomes less accurate.^169^ Some of these strategies can be complex, but others are relatively simple. For example, two related and complementary simple design strategies for SAAs are breaking a specific BEP relationship for a single elementary step, or promoting early reaction steps, like bond activation in reactants, while destabilizing downstream intermediates. Collectively, these effects have enabled the demonstration of active and selective hydrogenation,^46^ dehydrogenation,^56^ and epoxidation catalysts.^60^

### 2.5 Trimetallic Extensions of SAAs

Beyond SAAs, dispersed single-atom alloy trimetallic systems and paired dual-atom alloys (DAAs) expand the design space of well-defined catalytic interfaces by introducing more complex active sites.^34,174–177^ Computational studies have shown that embedding heterometallic dimers into coinage-metal surfaces can generate stable dual-atom sites that are often thermodynamically preferred over isolated single atoms or homometallic dimers.^174^ In contrast to conventional alloys, many DAAs inherit the weak dopant–host electronic interactions characteristic of SAAs while exhibiting new electronic and chemical properties arising from direct dopant–dopant hybridization. When both constituent SAAs display free-atom-like d states, formation of a DAA can lead to molecular-orbital-like bonding and antibonding states localized at the heterodimer, enabling tunable adsorption energetics, such as dopant- and site-specific modification of CO binding.^174^ More broadly, two classes of trimetallic SAA-derived architectures can be envisioned: dimer dopant pairs that form DAAs with potential for emergent electronic states, and spatially separated dopant atoms that provide chemically distinct properties and enable multistep or multifunctional reaction pathways *via* spillover between the two different active sites.^34^

The DAA concept is exemplified by trimetallic Pt–Cr sites embedded in a Ag(111) surface, which were identified through computational screening and confirmed experimentally by STM and TPD.^175^ Specifically, DFT predicted that the formation energy of PtCr heterometallic dimer pairs is negative and hence favored in the Ag(111) surface as illustrated in Figure 20a. In this system, the hydrogenphilic Pt atom and oxophilic Cr atom act synergistically to enable ethanol dehydrogenation, a reaction that is not possible on either of the corresponding PtAg or CrAg bimetallics. DFT revealed that, unlike the corresponding SAAs, these dual Pt–Cr sites stabilize the O–H bond activation transition state and final state to initiate the reaction (see Section 3.3.1 for more discussion). Collectively, these proof of principle studies establish trimetallic DAAs as a new class of catalytic active sites that preserve the localized, well-defined nature of SAAs while greatly expanding the design space through dopant–dopant interactions, enabling rational tuning of electronic structure and reactivity beyond what is achievable with single atom sites alone. It was recently demonstrated that PtCrAg films with dilute Pt and Cr content also exhibited selective ethanol dehydrogenation under near-ambient conditions.^178^ Additionally, a PtRuCo DAA has been shown to be highly active for hydrogen evolution,^179^ and a MoFePd metallene DAA has been shown to be active for electrochemical nitrate conversion to ammonia,^180^ suggesting this architecture may be useful for both electrocatalysis and thermal catalysis.

**Figure 20:**
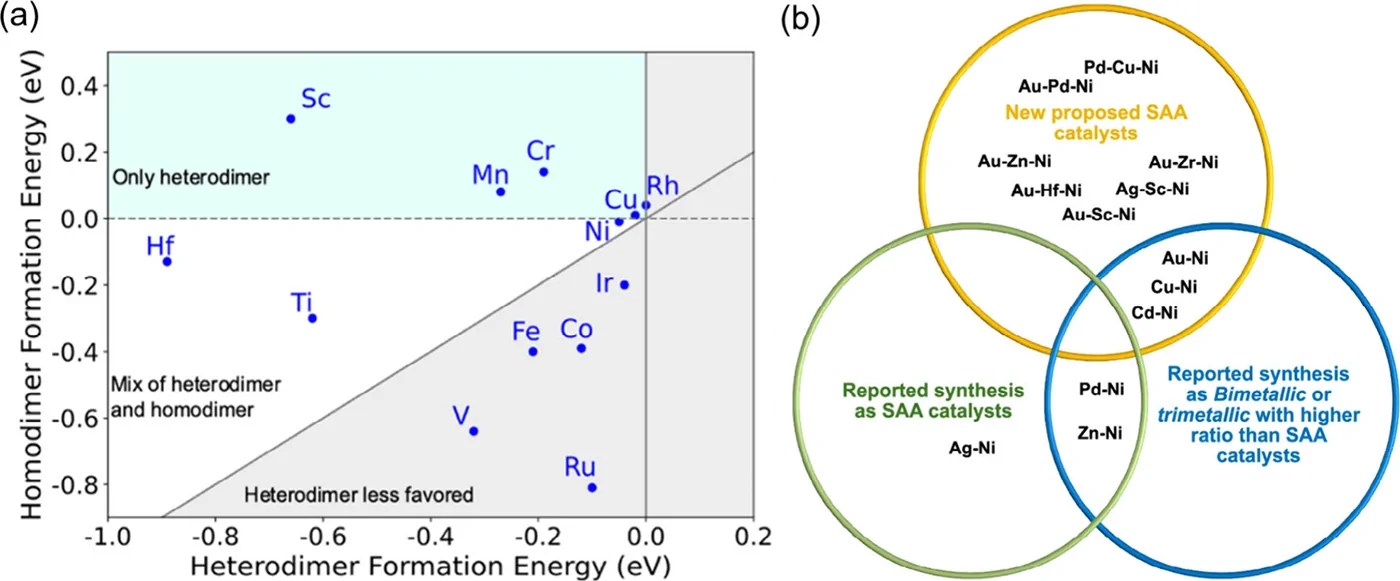
(a) Theoretical evaluation of heteroatomic dimer stability obtained by introducing a third metal into the PtAg SAA. The calculated formation energies for both homo- and heterodimer pairs are compared, with the shaded upper-left region highlighting the most promising systems characterized by energetically preferred heterodimer formation and unfavorable homodimer formation. Adapted from ref.^175^ Copyright 2023 The Authors. Licensed under CC BY 4.0. (b) Practical feasibility of synthesizing promising SAA candidates for the bio-oil-to-hydrogen pathway. Reproduced from ref.^181^ Copyright 2025 The Authors. Licensed under CC BY-NC-ND 4.0.

Recent theoretical and computational studies further demonstrate that the extension from SAAs to trimetallic SAAs and DAAs enables new approaches to catalyst design across diverse reaction classes. Descriptor-based DFT screening of Ni-based bimetallic and trimetallic SAAs for acetic acid dehydrogenation revealed that introducing a second dopant adjacent to the active site allows independent tuning of stability, hydrogen adsorption, and coke-resistance properties that are strongly correlated on monometallic and bimetallic surfaces.^181^ PdNi SAA sites were predicted to maximize H_2_ activity, while CuNi SAA sites promote hydrogen desorption specifically. Importantly, introducing a second dopant to the Ni host further modulates local electronic structure and site stability, yielding trimetallic SAA configurations that maintain balanced H* adsorption energy, enhanced C* gasification, and resilience under hydrogen-rich environments. Multiple promising trimetallic Ni-based SAA catalysts were identified by evaluating M1–M2 first-nearest-neighbor stability under vacuum and H* conditions, H_2_ activity, affordability, and regeneration capability. As summarized in Figure 20b, although numerous bimetallic and trimetallic Ni-based alloys have been reported, ratio-controlled synthesis of Ni-based SAAs remains limited, with most viable trimetallic systems incorporating Au or Pd together with an additional M2 dopant. Several additional computational studies have screened DAAs for stability or for various reactions including nitrate reduction and formic acid decomposition.^182–187^

In Pt–X–Cu systems, the introduction of Sn with a larger atomic radius and strong affinity for Pt induces surface segregation known as atom abstraction, drawing Pt from subsurface or bulk environments to form fully exposed atomic or dimeric motifs at the catalyst surface.^188^ The formation of Pt_1_Sn_1_ surface dimers is driven by geometric mismatch between Pt and Sn combined with favorable Pt–Sn interactions. This configuration stabilizes isolated metallic Pt sites under propane dehydrogenation conditions while significantly reducing noble-metal loading by maximizing the fraction of catalytically active atoms. This trimetallic configuration achieves near-total Pt surface exposure, enhances catalytic stability, and improves cost efficiency relative to conventional Pt-based catalysts, illustrating how cooperative dopant–dopant interactions can be leveraged to control active-site accessibility. Qualitatively similar results were found for the PdSnAg system, where Sn prefers to occupy the surface and there are attractive interactions between Sn and Pd.^189^ This allowed Sn to modify the reactivity of Pd single sites in the surface.

Parallel advances in Cu-based DAAs for CO_2_ activation reveal how dinuclear sites composed of early–late transition metal pairs (e.g., Y and Fe) can achieve strong covalent CO_2_ binding while retaining Cu-like CO adsorption energies, thereby mitigating surface poisoning and preserving downstream C–C coupling chemistry.^190^ In these systems, machine-learning analysis identified electropositive dopants as key contributors to CO_2_ activation, highlighting how asymmetric dopant–dopant interactions generate cooperative binding motifs reminiscent of enzymatic active sites. Collectively, these studies illustrate ways by which trimetallic SAAs and DAAs offer extended elemental structure space, as well as tunable stability, selectivity, and multifunctionality through rational control of atomic-scale site composition, geometry and stability.

Instead of tuning the dopant ensemble, it is possible to tune active site properties by using an alloy host. For example, Pt single sites in an Al_13_Fe_4_ intermetallic have been studied in the context of hydrogenation.^191^ Ru single sites in PtCu alloys have shown high performance for oxygen evolution, outperforming commercial RuO_2_ in terms of overpotential and lifetime.^192^ Surface science studies have shown that a SnAg host can tune the chemical and electronic properties of single-atom Pd sites, and relatively uniform sites can be created through annealing.^189^ Several other studies have examined alloy-host SAAs or similar architectures for various reactions.^193–200^ In principle, this architecture could allow a highly tunable and well-defined active site, and the design space is very large. However, synthesis and characterization could prove challenging. Table 2 summarizes the operational definitions and scope of various catalyst classes discussed throughout this review. It provides a concise framework for distinguishing these catalyst classes based on their structural characteristics, atomic distribution, and operational definition, thereby ensuring consistent terminology and facilitating comparison across the literature.

**Table 2:** Operational Definitions and Scope of Atomically Dispersed Alloy-Related Catalyst Classes Discussed in This Review

**Name**	**Description/operational definition**	**Key structural criterion**	**Examples from this review**	**Representative citation**
**Single-atom alloy (SAA)**	Composed of isolated catalytically active metal atoms typically dispersed within a more noble metal host surface	Isolated dopant atoms embedded in a metallic host lattice with no dopant–dopant nearest neighbors.	PtCu, PdCu, PdAg, PdAu, NiCu, NiAg, RhCu, PtAu + many more	^50^
**Dilute alloy**	Dilute alloys generally contain a low concentration of a catalytically active metal dopant distributed within a less reactive host metal. Depending on the arrangement of the dopant atoms at the surface, these systems typically contain isolated single atoms with coexisting dimers, trimers, or other small ensembles.	Low dopant concentration, but atomic isolation of dopant is not guaranteed.	Dilute PdAu, PtCu, NiCu, RhCu systems	^83^
**Nanoparticle single-atom alloy**	Nanoparticle SAAs consist of atomically dispersed active metal atoms in a second host metal in nanoparticle form.	Dopant atom in metallic nanoparticle hosts with atomic dispersion	PtCu/SiO_2_, NiCu/SiO_2_, PdCu nanoparticles, PtAu nanoparticles	^47^
**Supported single-atom catalyst (SAC)**	Unlike SAAs which have entirely metal–metal bonding, supported SACs consist of isolated single metal atoms anchored on the surface of a nonmetal support such as an oxide or carbonaceous material.	Single atoms coordinated to a support rather than embedded in a metal lattice.	Pt_1_/FeO_*x*_, Pt/CeO_2_, Rh/TiO_2_, single atoms in zeolites or carbon supports	^23^
**Dual-atom alloy (DAA)**	DAAs contain isolated heterometallic dopant atom pairs (AA or AB) embedded within the surface layer of a metallic host (C).	Isolated dopant–dopant pairs, often heterometallic, embedded in a metallic host.	PtCrAg, PtRuCo, MoFePd, Cu-based DAAs	^174^
**Trimetallic SAA**	Trimetallic systems consist of two different dopant atoms isolated from themselves and each other (A+B) in a third metal host (C).	Two different types of dopant atoms incorporated into a third host metal, yielding two different atomic active-sites.	Ni-based trimetallic SAAs, Pt–Sn–Cu, PdSnAg	^181^
**Intermetallic compound (IMC)**	Intermetallic compounds are ordered alloy phases with well-defined stoichiometry and uniform local coordination environments.	Long-range ordered alloy structure; atomic isolation can occur by crystallographic ordering rather than dilution.	Pt_3_Sn, Pt_3_Co, Al_13_Fe_4_, InPd_3_, InPd_2_Cu	^112^
**Dilute plasmonic alloy**	A dilute alloy in which a small number of reactive atoms are incorporated into or associated with a plasmonic metal host. The plasmonic metal can act as a light-harvesting antenna, while isolated dopants may serve as reaction sites.	Dilute reactive sites associated with a plasmonic host; atomic isolation must be demonstrated to classify as SAA-like.	light-driven methane dry reforming system	^75^
**Supported Catalytically Active Liquid Metal Solutions**	SCALMS materials consist of small amounts of catalytically active metal atoms dissolved in a low-melting liquid-metal host.	Isolated atoms in a liquid metal environment rather than a crystalline solid alloy surface.	PtGa	^201^
**Phase-separated alloy (PSA)**	An alloy in which the two metals form distinct domains or phases rather than an atomically dispersed or intermixed alloy.	Dopants cluster into separate domains from host metal.	CuNi, AgNi, or NiCu systems when phase separation occurs under certain coverages or conditions	^120^

### 2.6 Role of Theory in Predicting New Catalysts

A number of computational studies have performed screening to identify promising new candidates for different reactions.^202^ Various design principles have been used, often moving beyond a single descriptor due to the possibility of breaking scaling relations between reaction barriers and adsorption energies on SAAs. For example, in some cases a barrier (kinetics) and an adsorption and/or reaction step energy (thermodynamics) are both calculated, generally with the goal of identifying SAA candidates with a low activation barrier along with weak binding of adsorbates and intermediates.^56,60^ This approach serves to promote early reaction steps on the dopant site while destabilizing downstream intermediates on host sites or host-dopant mixed sites.^172^ Such promotion of the early steps can accelerate reactant activation, while destabilizing the downstream species helps prevent site poisoning, suppress nonselective pathways, and facilitate desorption of the desired products. Alternatively, developing specific scaling relations for a subset of systems can allow screening, understanding and prediction within those systems.^203^ Furthermore, given that e.g. hydrogen spillover to the host metal after H_2_ activation can enable selective hydrogenation reactions by virtue of weak binding of hydrogen to Cu, screening for favorable H spillover energetics can also be useful for identifying promising candidates for hydrogenation.^204^ Finally, more comprehensive screening that includes many possible reaction intermediates can bring more detailed insights into the design of SAA catalysts.^205^

These opportunities underscore the need for accelerated strategies to explore the vast compositional and structural space of SAAs and more complex surface motifs under relevant conditions. As an example, recent studies have demonstrated that ML can significantly accelerate the discovery and evaluation of SAA catalysts for ethanol dehydrogenation by efficiently capturing complex adsorbate–metal interactions.^206^ In one such approach, a gradient boosting regressor was trained to predict oxygen and carbon adsorption energies on Cu-, Ag-, and Au-based SAAs using readily accessible periodic properties of transition metals as descriptors. This data-efficient model achieved reasonable accuracy, enabling reliable estimation of key binding energetics with limited training data. Importantly, the ML framework was coupled with a microkinetic model for nonoxidative ethanol dehydrogenation, revealing that NiAu, NiAg, and PtAg SAAs exhibit higher turnover frequencies than their monometallic coinage-metal counterparts. This illustrates the nontrivial nature of adsorption on SAA surfaces and demonstrates that ML models can uncover physically meaningful descriptors beyond traditional electronic structure metrics. More broadly, the integration of ML with microkinetic modeling establishes a rapid, predictive platform for screening ethanol dehydrogenation catalysts, offering a rational pathway for identifying high-performance SAAs while reducing reliance on expensive quantum chemical calculations. Other ML methods, such as Bayesian optimization and an AI agent, have also been used to efficiently screen SAAs for multiple reactions, including CO_2_ electroreduction.^207,208^

#### 2.6.1 Machine Learning for SAAs: Capabilities, Limitations, and Opportunities

Because SAAs present well-defined, localized active sites, they are in several respects more amenable to both DFT modeling and ML than many other catalyst architectures, whose heterogeneous and often ill-defined active sites can complicate data generation and active site construction for screening. This has made SAAs a fertile testbed for data-driven catalyst design, as the examples throughout this review illustrate. Nonetheless, ML for SAA design inherits the broader capabilities and challenges of ML for heterogeneous catalysis as a whole.^209–211^ Two classes of model dominate the SAA literature: the first maps a composition or local structure directly onto a target property, such as a segregation, aggregation, activation or adsorption energy;^82,88,110^ the second consists of machine-learning interatomic potentials (MLIPs) that predict energies and forces as a function of atomic positions, which are particularly valuable for molecular dynamics and the study of processes such as spillover of intermediates and catalyst restructuring.^146,212^

ML is best suited to tasks such as rapid compositional screening across the large host-dopant space, identification of broad periodic and electronic trends, and prioritization of candidates for subsequent DFT calculations or experimental testing. The studies discussed above that screen hundreds to tens of thousands of host-dopant combinations for stability or activity demonstrate the power of ML in rapidly identifying candidates for further study.^82,161,206–208^ Problems that remain challenging are those that depend on phenomena poorly represented in the training data, that are challenging to validate with DFT, or that require long time scales and/or realistic conditions. This includes dynamic alloy restructuring, reaction-induced aggregation or segregation, adsorbate coverage effects, and the kinetics of elementary steps under realistic reaction environments including solvent, electrical potential, and temperature. Observing the details of these processes, such as large-scale restructuring, can require long time scales and large numbers of atoms, making them expensive to study even when accurate MLIPs are available. Thus, while ML can sometimes greatly accelerate such studies, these phenomena remain difficult to capture routinely, particularly in a design context.

Some of these limitations stem from the data on which the models are trained. Many models are trained primarily on DFT calculations of idealized, near-equilibrium structures at low adsorbate coverage, so systematic DFT errors, the neglect of coverage and finite-temperature effects, and sparse data density for specific systems of interest can all propagate into predictions. Predictive accuracy also varies substantially with the target property: in one representative study, gradient-boosted models predicted SAA segregation energies with R2 near 0.9 but trimer aggregation energies with R2 near 0.4.^83^ Extrapolation beyond the training domain is a particular concern for SAAs, whose distinctive electronic structure, such as free-atom-like d-states, early transition-metal dopants, or unusual partial charge states, can place systems of interest in sparsely sampled regions of feature space where model confidence is low and errors are larger. More recently, quantum dynamics simulations revealed that H_2_ dissociation on M_1_/Au­(111) (M = Cu, Pd, Ag) SAA surfaces proceeds through distinct mechanisms that depend on the dopant element.^213^ In particular, Ag was found to promote H_2_ activation through an unconventional intersite synergistic mechanism by electronically activating neighboring Au atoms, highlighting the diverse reactivity that can arise from different SAA active-site architectures.

Several strategies can mitigate these issues and represent promising directions for the field. Uncertainty quantification, via model ensembles, Gaussian-process variance, or related methods, allows predictions in extrapolative regimes to be flagged rather than trusted blindly, and has been applied within the SAA space to guide data acquisition.^187,214,215^ Active learning uses such uncertainty estimates to build compact, information-rich training sets, reducing the DFT cost of screening.^81^ Transfer learning and pretrained models, increasingly including large general-purpose “foundation” models, offer a route to data efficiency by providing useful levels of accuracy without additional training, along with the potential to be fine-tuned to SAA chemistries using limited additional data.^216–219^ Generative and inverse-design approaches invert the traditional ML workflow: instead of direct screening of the entire design space, they learn a representation of the space and optimize within it toward a target property, e.g. with generative diffusion models or reinforcement learning, and propose promising candidates directly. These approaches have begun to be applied to bimetallic and alloy catalysts.^220^ Finally, grounding models in experimental data collected under operating conditions, ideally within closed-loop or autonomous discovery workflows, should help bridge the gap between idealized training data and working catalysts.^221^ Importantly, these tools can augment physical understanding; as noted in our outlook (Section 6.1), simpler descriptor-based screening combined with strong mechanistic insight often remains effective, and increased model complexity yields diminishing returns once it moves beyond the fidelity of the underlying data.

We discuss specific computational design studies in more detail throughout this review in concert with our description of the corresponding applications. Table 3 highlights the tasks in SAA research for which ML is particularly well-suited, as well as those that remain challenging to address using current ML approaches.

**Table 3:** Tasks Where ML Is Well-Suited versus Challenging to Apply to the Design of SAAs

**Task or question**	**ML suitability**	**Basis**
**Compositional screening across the large host–dopant space**	Well-suited	A large, well-defined design space; ML accelerates initial screening
**Identifying broad periodic and electronic trends**	Well-suited	Trends are learnable from existing DFT data sets
**Predicting near-equilibrium properties (segregation, adsorption energies)**	Well-suited	Accuracy varies with the target property
**Prioritizing candidates for subsequent DFT or experiment**	Well-suited	ML acts as an accelerator ahead of higher-fidelity methods
**Dynamic restructuring; reaction-induced aggregation or segregation**	Challenging	Long time scales and large systems, poorly represented in training data; costly even with accurate MLIPs
**Adsorbate coverage and realistic reaction environments (solvent, potential, temperature)**	Challenging	Training data are typically idealized, near-equilibrium, and low-coverage
**Kinetics of elementary steps under operating conditions**	Challenging	Difficult to validate with DFT and usually absent from training data
**Extrapolation to distinctive SAA electronic structures from other data sets**	Challenging	Such systems can lie in sparsely sampled regions of feature space where model confidence is low
**Mitigation strategies: uncertainty quantification; active learning; transfer and foundation models; generative/inverse design; grounding in operando data.**		

### 2.7 Overall Design Strategies

There are a few broad strategies for identifying a potentially promising SAA for a given specific reaction (see Figure 21). For SAAs, a particularly promising design strategy is to identify a dopant that lowers the activation energy of the most rate limiting step, while choosing the host metal to tune adsorption strength, enable selectivity via weak binding of intermediates and suppress undesired reaction pathways and deactivation by e.g. coke. In several cases this leads to breaking of scaling relations, usually either due to differences in surface coordination between different intermediates and transition states, or due to spillover of reaction intermediates (see Section 2.4). For hydrogenation reactions, isolated Pd or Pt atoms in Cu, Ag, or Au hosts can facilitate H_2_ dissociation while maintaining weak adsorption of reaction intermediates (see Section 3.2.1). For oxidation reactions, more oxophilic dopant atoms may be chosen to activate molecular oxygen and/or stabilize oxygen-containing species (see Section 3.4.1).

**Figure 21:**
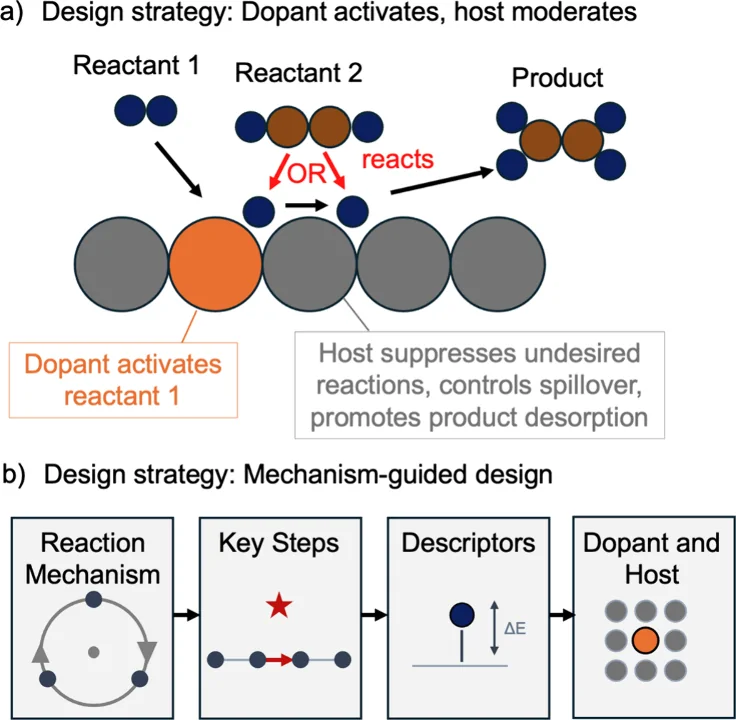
Two possible, complementary design strategies for SAAs for a given reaction.

In a related strategy, the rational design of SAA catalysts begins with comprehensive studies of the elementary reaction steps, and ideally the adsorbate–surface interactions that govern activity and selectivity. This is a common design strategy for catalysts of any type, particularly for reactions of moderate complexity. For SAAs, the localized and well-defined active site can facilitate effective modeling and intuitive reasoning, while the possibility of cooperativity between dopant and host sites can potentially be leveraged to improve performance (see Section 2.3.3). Therefore, in this picture the optimal SAA structure depends on matching the binding properties of the host–guest pair to the adsorption requirements of the rate-determining elementary steps. Since binding properties are governed by the electronic structure, electronic descriptors can be incorporated into the catalyst design workflow alongside structural descriptors, such as the degree of active-site isolation (see Section 2.3.2). However, the possibilities of surface segregation, dopant aggregation and adsorbate-induced restructuring (discussed in Sections 2.1 and 2.3.4) must also be considered in the overall design.

### 2.8 Conclusions and Outlook on Fundamental Properties

In this section, we discussed the physical concepts underlying SAAs and how their unique geometric and electronic properties govern their chemical behavior. The stability of SAAs can often be predicted from thermodynamic considerations, particularly aggregation and segregation energies. The structures of SAAs are known to be highly sensitive to their chemical environment, with some adsorbates capable of inverting segregation and aggregation behavior and both stabilizing and destabilizing the SAA regime. Poor hybridization of the dopant atom orbitals with those of the host metal often gives rise to free-atom-like d-states and hence different electronic structures than the pure metals. This, combined with the well-defined sites, often leads to higher stability and better predictability compared to their SAC counterparts which often have less well-defined active sites. DFT and surface experiments have helped in predicting and understanding supported SAA nanoparticle catalyst behavior. However, there are examples in which these approaches were not applicable.^154,222^

A new frontier in surface science research involves narrowing the pressure and material gaps between extended-surface UHV and ambient pressure NP studies. Ambient-pressure XPS, STM and IR studies of extended surfaces represent one approach to close the pressure gap.^31,223–225^ Another approach for the material gap is to prepare systems in UHV that have a greater structural similarity to NPs. In a recent example of this approach, Schauermann and co-workers prepared PdCu SAA clusters on a thin layer of Al_2_O_3_ on NiAl(110) in UHV.^226,227^ They then compared their reactivity for the selective nonoxidative butanol dehydrogenation to that of the extended PdCu(111) SAA surface. They observed even higher selectivity for the SAA clusters compared to the extended SAA surface, which they ascribed to the nanoscopic nature of the Pd/Cu particles. These aforementioned approaches are critical for further enhancing the predictivity and reliability of theory and surface science experiments on the pathway to realize new catalytic materials. In summary, Table 4 gives an overview of the SAA systems reported in this section and their relevant properties.

**Table 4:** Summary of Representative SAA Catalytic Systems, The Techniques Used to Verify Atomic Isolation, The Associated Reaction Type or Elementary Step, Key Catalytic Performance Metrics, Proposed Reaction Mechanisms, and Corresponding References

	**SAA system**	**Technique used to show evidence for atomic isolation**	**Reaction type/step**	**Key catalytic metric(s)**	**Proposed mechanism**	**Ref. #**
**1**	PtCu(111)	STM, TPD, and RAIRS	Hydrogenation	Highly selective hydrogenation	H_2_ activation on Pt, hydrogenation on Cu host	^30^,^31^,^35^,^37^,^45^,^223^
**2**	PdCu(111)	STM, TPD, and RAIRS	Hydrogenation	Selective hydrogenation	Spillover of the dissociatively adsorbed hydrogen from Pd atoms to the Cu surface enables selective hydrogenation	^46^,^47^,^99^,^142^,^143^,^149^,^155^,^225^
**3**	PdAg(111)	TPD, and RAIRS	Hydrogenation	Higher Pd coverage increases conversion of acrolein but lowers 2-propenol selectivity	Hydrogenation proceeds through stable surface intermediates with reaction rate-limited desorption. Pd single atoms increase conversion but shift selectivity away from 2-propenol toward propanal.	^140^
**4**	PdAu(111)	STM and TPD	Hydrogenation	Pd–Au monomers activate H_2_ at 85 K; H desorbs at 175 K, much lower than from pure Pd (310 K), indicating weak H binding.	Isolated Pd atoms dissociate H_2_, H atoms initially reside at Pd sites, and CO adsorption at Pd sites forces H spillover to Au sites.	^145^,^150^
**5**	PtCu(111)	STM, TPD, DFT (previous studies)	Hydrogenation	PtCu(111) activates CO_2_ to CO, with CO adsorbed on Pt sites up to 400 K; under CO_2_/H_2_, Pt promotes RWGS and stabilizes surface Pt at 450 K	CO formation and adsorption stabilizes Pt atoms at the surface. Under CO oxidation, O_2_ forms Cu_2_O and can bury/remove detectable Pt, but preadsorbed CO protects Pt and leaves exposed Pt sites in Cu_2_O “holes”	^224^
**6**	PtCu(111)	RAIRS, TPD, and STM	Dehydrogenation	C–H activation with simultaneous weak C binding	Pt enables C–H activation, PtCu maintains C–C coupling	^38^,^68^,^151^,^152^,^222^
**7**	RhCu(111)	STM and TPD	Dehydrogenation	Low-temperature nonoxidative dehydrogenation	Low carbon–hydrogen bond activation barriers on Rh atom dopants	^56^
**8**	NiCu(111)	TPD and STM	Dehydrogenation	NiCu(111) SAAs are more reactive than Cu(111)	Formic acid reaction via formate pathway: O–H activation forms formate intermediates, which are more stable at Ni sites, where rate-determining C–H activation occurs	^154^
**9**	PtCrAg(111)	TPD and STM	Dehydrogenation	Pt–Cr dual-atom sites in Ag(111) convert ethanol, while PtAg and CrAg single-atom alloys are unreactive	Oxophilic Cr binds the O-containing fragment, while hydrogenphilic Pt assists O–H bond cleavage	^175^
**10**	PdCu(111)	STM, TPD, and RAIRS	Dehydrogenation	High selectivity of Isolated Pd atoms on Cu(111) terraces	Terrace Pd atoms favor H abstraction/dehydrogenation, while Pd ensembles and step-edge Pd enable C–C/C–H bond scission	^226^,^227^
**11**	RhCu(111)/NiCu(111)	STM, TPD, RAIRS	Alcohol decomposition/synthesis	On NiCu(111), ethanol forms acetaldehyde, whereas on RhCu(111), it forms methane and CO	Rh single-atom sites lower the activation barrier for C–C bond cleavage, promoting methane formation.	^91^
**12**	PtCu(110)	STM	Alcohol decomposition/synthesis	Strongly suppressed sintering in PtCu relative to Cu	Pt dopants localize near undercoordinated Cu atoms and slow Cu atom emission during Ostwald ripening	^98^
**13**	NiAg(111)	STM, and TPD	Epoxidation	Increase in the selectivity of ethylene epoxidation	Ni boosts selectivity by stabilizing unselective nucleophilic oxygen	^60^
**14**	PdCu(111)	STM (previous work)	Formic acid decomposition	HCOOH to bidentate formate conversion was 12%, similar to Cu(111)	HCOOH adsorbs preferentially at Pd, dissociates to monodentate formate + H at 150–160 K, converts to bidentate formate on Cu at 300 K, then decomposes at ∼ 450 K.	^144^
**15**	NiAu(111)	STM, and RAIRS	Carbon–carbon coupling	selective sp^3^-sp^3^ carbon–carbon coupling	Iodine atoms strongly bind to and block the Ni active sites, forcing the methyl groups onto the surrounding Au surface, Ni then mediates coupling	^72^
**16**	PdAu(111)	STM, and RAIRS	Carbon–carbon coupling	selective sp^3^-sp^3^ carbon–carbon coupling	Balanced methyl and iodine binding at Pd sites	^72^
**17**	PdAg(111)	TPD, and RAIRS	CO adsorption	Pd aggregation begins above 0.002 ML, giving bridge-bound CO	H_2_ can dissociate at Pd sites, but the resulting H atoms do not spill over onto Ag	^127^
**18**	NiCu(111)	RAIRS, STM, and TPD	CO adsorption	CO binds more weakly to isolated Ni atoms in Cu than to Ni(111), suggesting reduced CO poisoning	Isolated Ni atoms alloy into Cu(111), especially in Ni-rich brims at ascending step edges	^128^
**19**	RhCu(111)	RAIRS, STM, and TPD	CO adsorption	CO vibrational signatures on isolated Rh sites	Rh alloys into Cu(111) near step edges, forming isolated Rh sites whose CO vibrational behavior depends on step density and hence local Rh–Rh spacing.	^129^
**20**	RhCu(100)	RAIRS, STM, and TPD	CO adsorption	Direct evidence of mono- and dicarbonyl CO species on isolated Rh sites in RhCu(100)	The surface switches between mono- and dicarbonyl configurations depending on temperature and CO pressure	^131^
**21**	NiCu(100)	STM, RAIRS, and TPD	CO adsorption and activation	NiCu(100) strengthens CO adsorption under UHV	At low CO coverage, CO activation is thermodynamically forbidden; at high CO coverage, adsorbate–adsorbate repulsion raises the CO energy state and opens dissociation pathways	^160^

## 3 Thermal Catalysis

### 3.1 Introduction

Following the initial report of the SAA paradigm in 2012,^46^ we reviewed SAA-related studies on a reaction-by-reaction basis in 2020, with a primary focus on thermal catalysis.^33^ Building on that foundation, this section of the review serves as an updated and expanded overview of SAA applications reported over the past six years since 2020. We highlight recent work in the area of thermal catalysis by SAAs on a reaction-by-reaction basis, organized into classes of reactions: hydrogenation, dehydrogenation, selective oxidation, coupling chemistries, and other reactions. For each reaction class, we describe whether SAAs were used to exploit the original concept of molecular activation on reactive single atom sites followed by spillover to host coinage metal sites, or if SAA reactivity occurred through other mechanisms. Further, in each example we summarize how reactivity and selectivity were influenced by SAA structure and composition. In addition, the studies reviewed provide varying levels of evidence for three related aspects of SAA catalysis: (1) whether dopant atoms are (primarily) atomically isolated within the host metal, (2) whether the isolated dopant sites or dilute ensembles are responsible for the observed catalytic performance or stability, and (3) whether the proposed SAA structure is preserved or dynamically evolves under pretreatment or reaction conditions. This latter point is particularly important because adsorbate- and reaction-induced restructuring, reversible segregation, aggregation, and dynamic ensemble evolution can alter the identity and abundance of active sites during catalysis. Accordingly, throughout this section, we discuss how each study supports these claims, what characterization methods were used to establish the SAA structure and its structural evolution, and how these structural features correlate with the reported catalytic behavior. We point to previous reviews and original contributions that highlight the use of various characterization tools for identifying single atom active site structures.^228–231^ We note that no characterization tool is able to provide quantitative evidence of the sole existence of isolated atoms in a sample. Each characterization tool has limits of detection and operational conditions. The optimal approach to substantiate the importance of single atom active sites in SAAs is through systematic studies of the influence of dopant to host molar ratio on reactivity (along with associated characterization) with the convergence of catalytic behavior at the limit of very low <1:100 molar ratios.^28^ However, this approach can also be challenging if the host metal has inherent reactivity.

### 3.2 Hydrogenation Reactions

Hydrogenation reactions on catalytic surfaces require activation of H_2_ and selective addition of hydrogen to unsaturated bonds. The ideal catalyst dissociates H–H bonds with relatively low activation barriers while adsorbing reactants and intermediates with optimal binding energies. This enables hydrogenation of only the desired moieties without molecular decomposition that occurs on strongly binding surfaces. The original idea for the first PdCu and PtCu SAAs was that isolated reactive atoms provide sites for H_2_ dissociation and hydrogen spillover, while adjacent coinage host metals serve as the hydrogenation sites that enable high product selectivity due to the moderate hydrocarbon or oxygenate binding energy. As a result, SAAs can combine low barriers for H_2_ activation with weak binding of H atoms, thereby breaking the linear scaling relationship that links H_2_ dissociation to H atom adsorption strength (sometimes described as the selectivity-activity trade-off), and these ideas have motivated extensive recent work.^61^ In this section, we start by reviewing reports that either demonstrate or further interrogate the originally proposed hydrogenation mechanism for SAAs, highlighting why SAAs are effective for hydrogenation reactions. We then follow with a review of publications describing SAAs that exploit new mechanisms for hydrogenation reactions, such as combining two reactive elements with surface structures that enable desired reactivity. Throughout, we focus on examples where the compositional space of bimetallic catalysts is varied sufficiently to demonstrate the advantage of the SAA structure over larger ensembles.

#### 3.2.1 Catalysis by Hydrogen Spillover from Reactive Site (Dopant) to Coinage Host

In SAA-catalyzed hydrogenation reactions, the commonly proposed mechanism involves reactive dopants such as Pd or Pt serving as the active sites that facilitate H_2_ dissociation, whereas hydrogenation occurs on coinage metal hosts (Cu, Ag, Au) enabled by H spillover, and thus the coinage metal surface chemistry governs selectivity by enforcing preferred reactant and intermediate adsorption geometries and energies, as discussed in detail in the previous section. For example, when H atoms spill over from a Pt or Ni dopant atom to Cu, Cu surface sites are selective for hydrogenation of specific unsaturated bonds due to reactant adsorption specificity.^232,233^ A simple design principle for the selective hydrogenation of α,β-unsaturated aldehydes is to promote the preferential binding of the reactant through the terminal C = O group rather than the C = C bond, leading to selective hydrogenation of only the C = O group.^234,235^ As discussed below, this picture does not perfectly predict selectivity, but can serve as a guide for initial screening.

While previous studies demonstrated selective propyne hydrogenation on PdCu SAAs and optimized reaction conditions to maximize propene selectivity, kinetic parameters were not reported.^236^ Trenary and co-workers studied selective propyne hydrogenation to propene on 2% of a monolayer PdCu(111) SAAs and employed RAIRS to quantify propene and propyne in a batch reactor, enabling extraction of apparent kinetic parameters.^225^ On Cu(111), propyne conversion reached 4.8% after 12 h with 28% selectivity to propene, whereas the 2% PdCu(111) SAA reached 100% conversion with 50% selectivity. For 2% PdCu(111), an apparent activation energy of 39.4 ± 0.9 kJ/mol and reaction orders of – 0.36 in propyne and 1.05 in H_2_ were measured, which are comparable to those reported for a pure Pd catalyst (39.7 ± 0.8 kJ/mol). The authors used this comparison to conclude that Pd in 2% PdCu(111) serves as the hydrogenation site,^225^ but did not explain the mechanistic picture that allows the SAA to combine the activity of Pd with the selectivity of Cu surfaces. The same group reported a similar analysis of the selective hydrogenation of 1,3-butadiene with a similar conclusion that the hydrogenation kinetic parameters are similar for PdCu SAAs compared to pure Pd.^149^

Interestingly, the authors also reported that H spillover does not occur on PdAg(111) SAA surfaces. Instead, Pd is proposed to retain dissociated H species, which may limit the use of this SAA for semihydrogenation of alkynes and demonstrates the importance of the nature of the SAA host metal.^237^ This is in apparent contradiction with work from Friend and co-workers, which proposed that H spillover can occur from Pd islands to neighboring Ag surfaces.^238^ Perhaps the local structure of the Pd, specifically the larger ensembles present in the system studied by Friend, is key to enabling H spillover to Ag, although this requires further analysis.

A recent study identifies lattice expansion in the Cu host of a Zn/Cu SAA as a key factor governing enhanced CO_2_ hydrogenation to methanol.^239^ Combined in situ experiments and DFT calculations showed that the resulting geometric and electronic modifications facilitate H_2_ and CO_2_ activation, promoting the formate (*HCOO) pathway and improving methanol production at low temperatures.

A theoretical analysis of alkyne hydrogenation on PdAu SAAs provided insights that may help explain the similar apparent activation energies for Pd-based SAAs and pure Pd catalysts, albeit with different selectivity, for this class of reactions. Sautet and co-workers examined selective hydrogenation of 1-hexyne to 1-hexene, with overhydrogenation to *n*-hexane as the unselective pathway, using a combination of microkinetic simulations and isotopic exchange experiments during hydrogenation on Pd_0.04_Au_0.96_ SAAs.^240^ Using degree of rate control analysis (Figure 22a), they found that the H_2_ dissociation transition state, occurring on Pd, was the primary rate controlling step for 1-hexene formation. This explains the similar apparent barrier for semihydrogenation on pure Pd and Pd-based SAAs. However, analysis of degree of selectivity control in Figure 22b showed that 1-hexene selectivity was governed by hydrogenation of the alkyne on the Au surface. Thus, the selectivity-controlling step occurs after the rate-controlling step on PdAu SAAs, explaining why Pd-based SAAs can display similar apparent barriers to Pd with drastically different selectivity. Interestingly, the authors also argue that surface H coverage on the Au host, rather than the different adsorption energies of the alkene and alkyne, dictates the selectivity.

**Figure 22:**
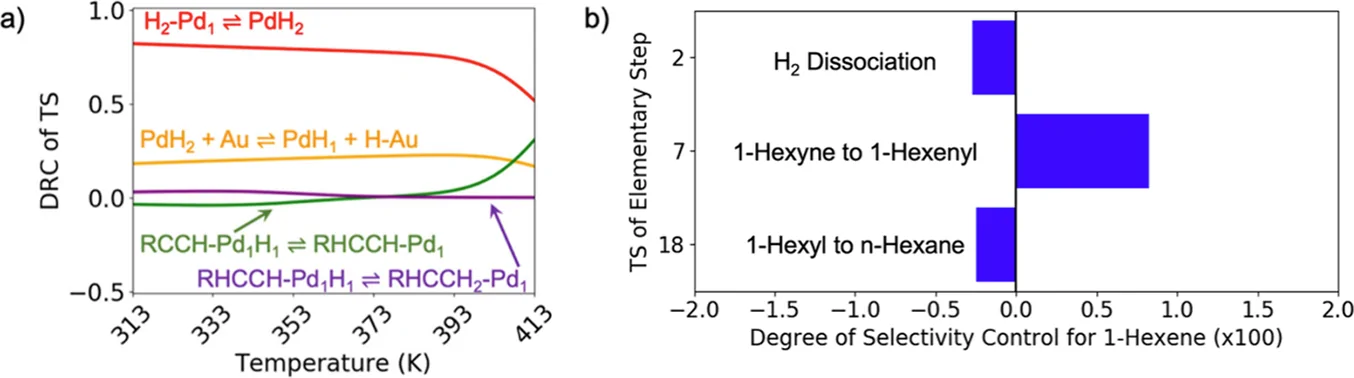
Analysis of the kinetics of 1-hexyne hydrogenation shows H_2_ dissociation is the rate-controlling step. (a) Degree of rate control shows that the H_2_ dissociation transition state (red) is the main rate-controlling step, followed by H migration from Pd to Au (orange), whereas C*–*H bond formation steps (green and purple lines) are not rate-controlling. (b) Degree of selectivity control, evaluated at *T* = 373 K. Adapted with permission from ref.^240^ Copyright 2022 American Chemical Society.

While the selective hydrogenation of alkynes to alkenes is a crucial first step, the subsequent transformation of the resulting alkene intermediate determines the final product distribution. Notably, isomerization was found to be a significant pathway competing with overhydrogenation in these systems. Friend and co-workers used 1-hexene hydrogenation and isomerization as a model reaction over Pd_4_Au_96_ SAAs and benchmarked them against monometallic Pd.^241^ At the same 1-hexene conversion of 92%, Pd_4_Au_96_ suppressed overhydrogenation, yielding only ∼ 11% *n*-hexane compared to about 33% on Pd, and the combined selectivity to the 2 and 3-hexene isomers increased from about 67% on Pd to about 89% on Pd_4_Au_96_. The authors proposed that the reaction followed a Horiuti-Polanyi mechanism, in which hydrogen was added stepwise through a half-hydrogenated hexyl surface intermediate. By fitting a DFT-parametrized microkinetic model to the measured deuterium distributions in the C_6_ products, together with the overall selectivity, they concluded that alloying Pd into Au decreases the barriers for H_2_ dissociation and H spillover but maintains low surface coverage of H, thereby favoring reversible hexyl rearrangement to 2 and 3-hexene over further hydrogenation to *n*-hexane. This study quantitatively identified why isomerization is favored relative to full hydrogenation by combining isotope exchange experiments with DFT. However, because the catalyst composition was fixed at Pd_4_Au_96_ without performing experiments on a range of host:dopant ratios, it lacks direct assessment of the importance of the SAA structure.

Based on a comparison of reports on hydrogenation reactions on SAAs containing Pd as the active metal, an important issue to address is how the host metal, reaction conditions, and Pd structure influence H spillover. While H spillover from Pd to Cu has been demonstrated many times and H spillover from Pd to Au was proposed by Sautet and co-workers,^240^ there are conflicting reports regarding H spillover from Pd to Ag. The PdAu studies above used Pd-monomer models based on prior evidence that monomers predominate at the surface. However, because H_2_ activation and spillover may depend on Pd ensemble size, minority ensembles and pretreatment- or reaction-induced changes in surface Pd distribution may also contribute to the reported behavior.^242^ Thus, a comprehensive analysis of the requirements (host composition, conditions, Pd structure) for H spillover for this class of SAAs, combining structural characterization with kinetic measurements under reaction conditions, would be valuable.

Beyond alkyne semihydrogenation, SAAs have been designed to mitigate activity-selectivity trade-offs and to maximize precious metal utilization in oxygenate hydrogenation.^68,243^ Kyriakou and co-workers examined furfural hydrogenation on PdCu SAAs with the goal of optimizing Pd utilization by varying catalyst compositions including Cu, Pd, Pd_1_Cu_53_, Pd_1_Cu_216_, and Pd_1_Cu_234_.^244^ The Pd_1_Cu_216_ catalyst exhibited the optimal Pd utilization, with a turnover frequency (TOF) ∼ 11× greater than that of pure Pd, while retaining almost quantitative furfuryl alcohol selectivity. The decreased TOF for Pd_1_Cu_53_ compared to Pd_1_Cu_216_ was attributed to Pd migration to the Cu bulk based on EXAFS measurements, which was proposed to decrease the rate of H_2_ activation. This work concluded that the catalytic benefit of PdCu SAAs depends on both dopant fraction and dopant location, because isolated Pd must remain surface accessible to serve as a H_2_ dissociation site.

Meyer and co-workers showed that PdAg SAA catalysts are active and selective for the hydrogenation of acrolein.^36^ Their EXAFS analysis suggested that at Pd loadings as low as 0.01 wt %, Pd atoms are fully dispersed within Ag nanoparticles as isolated sites. They proposed the isolated Pd centers enhanced catalytic activity in acrolein hydrogenation by providing sites that facilitate H_2_ dissociation. For nanoparticles of comparable size, the alloy containing 0.01 wt % Pd and 8 wt % Ag alloy also showed greater selectivity than monometallic catalysts. This behavior suggests that individual Pd atoms also create favorable adsorption environments for acrolein, promoting selective hydrogenation of the aldehyde group. This work further raises the question for PdAg SAAs as to whether all hydrogenation steps occur at Pd sites or if the reaction is mediated through H spillover to Ag.

Recent work on the location of the dopant atoms in SAAs by Zaera and co-workers analyzes PtCu SAAs in the context of the hydrogenation of unsaturated aldehydes, attempting to interrogate the location of Pt atoms under reaction conditions. Using EXAFS and DFT, Pt is proposed to reside under the Cu surface and at the particle-SiO_2_ support interface under reaction conditions and induce a remote promotion of H_2_ activation at the Cu surface, rather than serving directly as the H_2_ dissociation site.^245,246^ However, XAS analysis is an inverse problem – a structure is proposed and the resulting spectra is compared to the experiment. Thus, while a proposed structure can give a good fit to experimental data, the robustness of the structural assignment requires comparison to other possible structures; even then, the ensemble-averaged nature of XAS can make it difficult to distinguish isolated dopant atoms from minority populations of dimers or small clusters. Further, the proposed subsurface location of Pt in Cu particles is at odds with direct visualization using *in situ* TEM by Zhou and co-workers, who observe preferential Pt localization at stepped Cu surfaces under H_2_ environments.^247^ The different observations obtained for supported nanoparticles and well-defined Cu­(Pt) single-crystal thin films under distinct H_2_ pressures and temperatures suggest that further efforts are needed to more fully understand the distributions of dopant locations that exist in complex supported catalysts under realistic conditions.

Alongside the mechanistic interrogations of previously reported SAA compositions for selective hydrogenation reactions, DFT calculations coupled with microkinetic modeling have been used to screen reactive dopants and guide SAA design toward desired properties. Using DFT and microkinetic modeling, Sautet and co-workers predicted how different transition metal dopants in Cu-based SAAs can tune selectivity of acrolein hydrogenation toward propenol.^248^ Dopants were first screened by calculating their segregation and aggregation energies in Cu(111), followed by the calculation of the adsorption energies of acrolein in both the C = O and C = C binding modes. Based on this screening, Cr and Mn were identified as promising dopants because they favor the C = O bond adsorption mode with moderate binding strength. Free energy profiles and associated microkinetic modeling for acrolein hydrogenation on CrCu revealed the barrier for migration from the C = O to the C = C binding mode as the selectivity controlling step and the source of the high selectivity predicted for this catalyst. This work continues to raise questions on whether reactive dopant sites solely serve as active sites for H_2_ activation in hydrogenation reactions or whether they also serve as the hydrogenation site. Trenary and co-workers provided further evidence of this in the context of acrolein hydrogenation on PdAg(111) SAAs, where Pd loadings below 1% in the SAA regime decreased propenol selectivity, demonstrating that dopants in certain SAAs may serve as unwanted hydrogenation sites.^140^

The first report of catalytic transfer hydrogenation of furfural to 2-methylfuran by Cu-based SAAs comes from Wang and co-workers, using isopropanol as the source of hydrogen.^249^ A volcano-type relationship was observed between the catalytic performance and the Cu:Pd molar ratio across catalysts synthesized at Cu:Pd = 20:1, 8:1, 4:1, and 1:1 ratios, where the Pd_1_Cu_8_/Al_2_O_3_ sample delivered the highest selectivity of 81%. Higher Pd loading led to Pd clustering and increased unselective 2-methyltetrahydrofuran products. Mechanistically, isotopic labeling with isopropanol OD and isopropanol D8 supported a mechanism in which furfural was first converted to furfuryl alcohol through a metal-site-mediated transfer hydrogenation route, followed by furfuryl alcohol conversion to 2-methylfuran *via* a Lewis-acid-mediated route. Oxidized Cu (CuO) was implicated in the ring activation pathway, with the highest selectivity obtained at intermediate Pd loadings where Pd remained atomically dispersed. This suggests that transfer hydrogenation may be mechanistically similar to SAAs in hydrogenation reactions by H_2_, although future work should further interrogate these systems. A Pt_1_Ni SAA catalyst was shown to enable efficient hydrogen-free lignin depolymerization,^250^ producing high yields of phenolic monomers while preserving cellulose under mild conditions. Mechanistic studies and DFT calculations revealed that the synergistic interaction between isolated Pt atoms and Ni sites promotes selective dehydroxylation and formation of key unsaturated intermediates, thereby enhancing the production of valuable propenyl-substituted phenols.

This summary of recent selective hydrogenation work continues to substantiate the utility of SAA structures with separated H_2_ activation and reaction sites. Interesting questions have been raised both about the location of reactive dopant elements under reaction conditions and more broadly about their function. Do dopants always serve as just reactive spillover sites for hydrogenation equivalents? Or do they also serve as hydrogenation sites? These questions will undoubtedly draw further theoretical and experimental interrogation and are likely highly dependent on material composition, catalyst structure, and reaction conditions. Interestingly, the field has also begun to consider the function of SAA structures where noncoinage metal reactive elements are both the host and dopant.

#### 3.2.2 Hydrogenation through Engineered Host-Dopant Electronic Interactions

In this section, we discuss reports where the proposed mechanism of hydrogenation reactions on SAAs extends beyond hydrogen activation on an isolated dopant atom followed by spillover to the host. Instead, these reports propose that SAA reactivity is shaped by electronic interactions between the host and dopant metals, as well as by the local interfacial sites formed at the host-dopant boundary. In many cases, both metals are reactive metals (not coinage metals) and contribute to the overall catalytic behavior, either by tuning adsorption energetics or by modifying reactivity at the host-dopant interface. These examples therefore highlight a distinct function of SAA catalysts in hydrogenation reactions in which performance emerges from coupled host-dopant effects rather than from spillover to a less reactive but more selective host.

The hydrogenation of levulinic acid to gamma valerolactone over RuCo bimetallic catalysts on N–C supports was reported by Ke and co-workers.^251^ Catalysts were labeled Ru_1_Co@N–C-x where x denotes the RuCl_3_ concentration used during synthesis while the Co precursor amount was kept constant, giving Ru loadings of 0.03, 0.07, 0.30, and 0.74 wt % for x = 0.1, 0.4, 1.1, and 3.3, respectively, with Ru nanoparticles appearing at the Ru_1_Co@N–C-3.3. These catalysts were prepared by pyrolysis of Ru-containing ZIF-67, which provided the Co matrix and generated an N-doped carbon scaffold that helped stabilize Ru atoms within the Co lattice and suppress aggregation. In looking at Figure 23, the reaction rate increased linearly with Ru at% at lower loadings and more slowly at higher Ru content where Ru nanoparticles form (Ru_1_Co@N–C-3.3), consistent with atomically dispersed Ru serving as active sites. Ru_1_Co@N–C-1.1 had 3-fold higher reactivity and similar selectivity compared to the pure Ru catalyst (Ru_1_@N–C) leading the authors to conclude that charge transfer from Co to Ru enhanced the reactivity and that the reaction primarily occurred at Ru sites.

**Figure 23:**
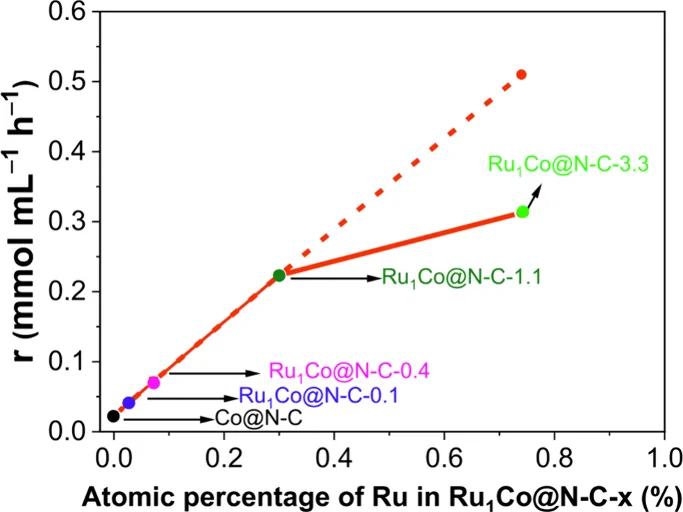
Comparison of reaction rates of levulinic acid to γ-valerolactone for Ru_1_Co@N–C-x with increasing Ru content, where x denotes the RuCl_3_ feed concentration used during synthesis and the corresponding Ru loadings are 0.03, 0.07, 0.30, and 0.74 wt % for Ru_1_Co@N–C-0.1, Ru_1_Co@N–C-0.4, Ru_1_Co@N–C-1.1, and Ru_1_Co@N–C-3.3, respectively. Reaction conditions: 5 mmol of levulinic acid, 30 mL H_2_O, 80 mg of catalyst, 150 °C, and 4.5 MPa of H_2_ pressure. Adapted with permission from ref.^251^ Copyright 2021 American Chemical Society.

Wei and co-workers reported two studies on the selective hydrogenation of 4-nitrostyrene (4-NS) to 4-aminostyrene (4-AS) over SAAs with Ni serving as the host metal.^252,253^ A series of bimetallic catalysts was prepared by varying Ru loading from 0.1 to 2 wt % while keeping the Ni content constant at 23 wt %. The optimal 0.4 wt % RuNi (1:99 atomic ratio) SAA delivered greater than 99% 4-AS yield with a TOF of 4290 per hour per Ru, outperforming monometallic Ni (7.1% conversion with 11.3% 4-AS selectivity) and monometallic Ru, which reached 100% conversion but was unselective, giving only 15.7% 4-AS selectivity due to preferential C = C hydrogenation. The high selectivity required the SAA structure because, according to DFT, this active site enforced a parallel nitro adsorption motif and decreased the N–O cleavage barrier below that for C = C hydrogenation. Future spectroscopic analysis may provide more direct evidence of this mechanism controlling selectivity.

Further, Wei and co-workers used DFT to screen 15 Ni-based SAAs to target NO_2_ over C = C hydrogenation, identifying IrNi SAAs as promising. Hydrogenation experiments using a 1:1 mixture of nitrobenzene and styrene yielded pseudo-first-order hydrogenation rate constants of 1.8 h^–1^ and 0.05 h^–1^, respectively. Based on these results, they concluded that the NO_2_ activation pathway is favored on IrNi SAAs in which the kinetically relevant nitro reduction step on IrNi is predicted to be more facile than the competing C = C hydrogenation step.^253^

Xiong and co-workers synthesized PdNi SAAs *via* galvanic replacement using 2 wt % Ni as the dominant component, while varying Pd from 0.01 to 0.2 wt %, which corresponds to a Pd:Ni molar ratio range of approximately 1:36 to 1:18, and studied the semihydrogenation of acetylene to ethylene.^254^ Pd_0.1_Ni_2_, corresponding to a Pd:Ni molar ratio of 1:36, achieved 91.4% ethylene selectivity at 95% conversion at 140 °C and maintained above 85% selectivity at 180 °C, whereas monometallic Ni/SiO_2_ remained inactive up to 150 °C. The authors further note that Pd_0.1_Cu_2_ and Pd_0.2_Cu_2_ prepared by the same GR method exhibited lower activity than Pd_0.1_Ni_2_, motivating the use of Ni as the host. DFT calculations attributed the high selectivity to electron-rich Pd sites formed by electron transfer from Ni, which weakened ethylene adsorption and suppressed overhydrogenation, consistent with the C_2_H_4_ TPD results. Further studies could use microkinetic modeling to clarify the mechanistic basis of the high selectivity and may also consider long-term catalyst stability when using Ni as the host for dispersed Pd sites.

Using toluene hydrogenation as a probe reaction, Satsuma and co-workers constructed PtM (M = Co, Ni or Cu) SAA catalysts to examine how the host metal tunes the reactivity of dispersed Pt sites in SAA structures.^255^ Extensive analysis of particle size and composition-dependent reactivity demonstrates optimal Pt mass specific activity for Pt_0.034_Co_5.3_ SAA catalysts, 23-fold higher than that of 2 wt % Pt nanoparticles (Figure 24). DFT and AIMD calculations suggested that toluene selectively adsorbs on Co ensembles around Pt sites and that H_2_ activation occurs on the Pt site. Additional, direct experimental evidence that toluene resides on Co ensembles neighboring Pt and that H_2_ activation does not occur on Co would help strengthen these claims.

**Figure 24:**
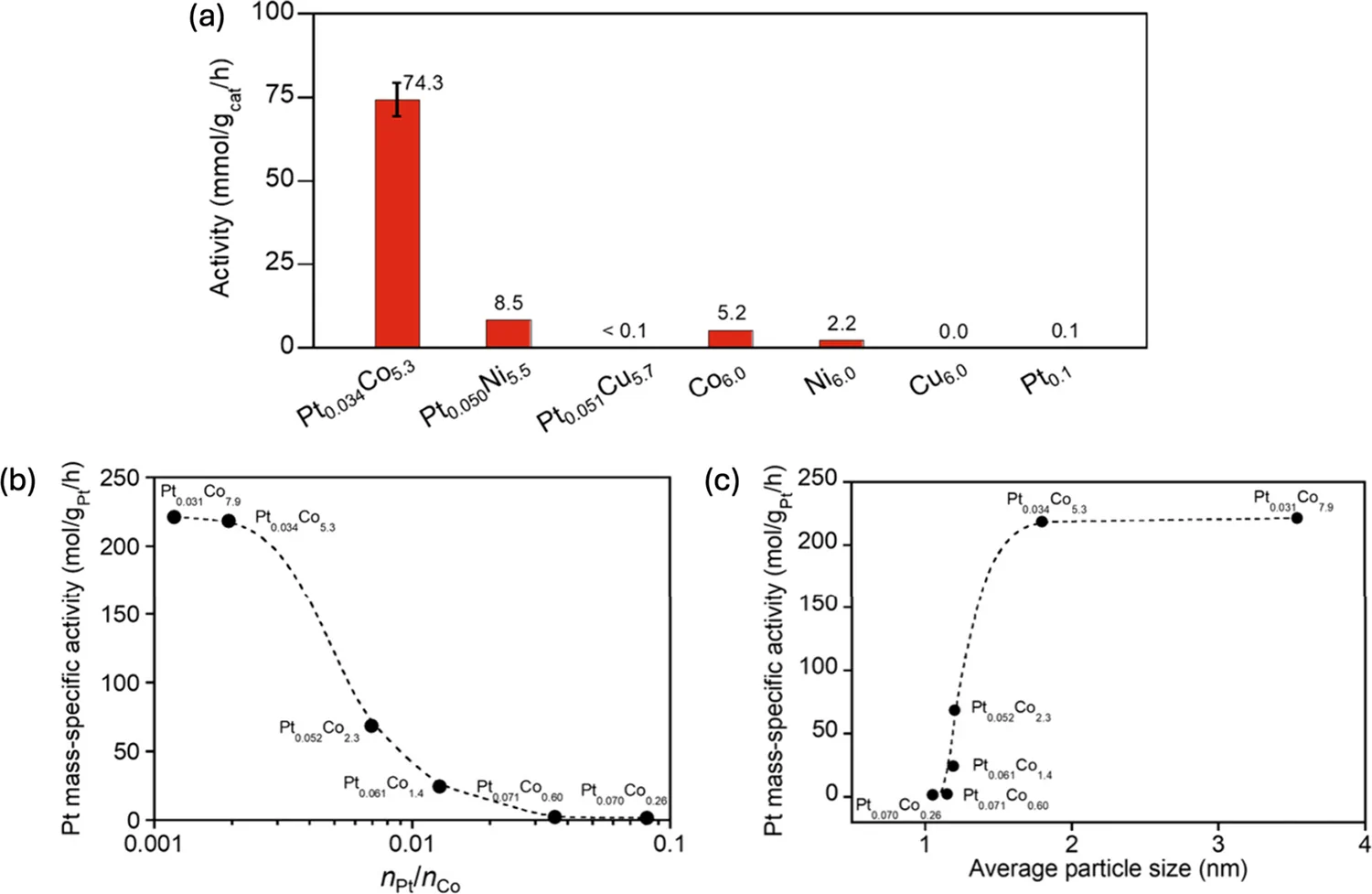
(a) TOL hydrogenation activity of PtM SAAs (Pt_0.034_Co_5.3_, Pt_0.050_Ni_5.5_, and Pt_0.051_Cu_5.7_), base-metal nanoparticle supported catalysts (Co_6.0_, Ni_6.0_, and Cu_6.0_), and Pt supported catalysts (Pt_0.1_ and Pt_2.0_), (b) Pt mass-specific activity on n_Pt_/n_CO_, and (c) Particle size dependency of Pt mass-specific activity. Adapted with permission from ref.^255^ Copyright 2023 American Chemical Society.

Wei and co-workers investigated the aqueous-phase hydrogenation and rearrangement of furfural to cyclopentanol on PtCo SAAs.^256^ The optimized PtCo SAA, containing 0.89 wt % Pt and 36.4 wt % Co (Pt:Co molar ratio ≈ 1:135), achieved an excellent cyclopentanol selectivity of 99.1%. The catalyst exhibited a TOF of 2257 h^–1^ based on Pt sites and 564 h^–1^ based on Pt–Co interfacial sites at low furfural conversion (∼15%), placing it among the most active systems reported. In contrast, monometallic Co, Pt, and physical mixtures of Pt and Co catalysts showed much lower activity or cyclopentanol selectivity than the PtCo SAA. The exceptional performance of the bimetallic catalyst was attributed to electron transfer from Co to Pt that tuned the electronic structure of Pt–Co sites. However, it is worth noting that PtCo bimetallic catalysts with larger Pt contents provided similar activity and selectivity, albeit with a slight increase in over hydrogenation. It is not clear from this result if the Pt single atom structure is needed for high activity or if the overall Pt content dictates high selectivity.^256^

Two representative studies on CO_2_ hydrogenation over RuNi SAAs report different products depending on particle size and composition.^257,258^ Su and co-workers synthesized RuNi/SiO_2_ *via* galvanic replacement using 24.4 wt % Ni as the dominant component with 0.3 wt % Ru, corresponding to a Ru:Ni molar ratio of approximately 1:140, and obtained ∼ 5 nm diameter particles. The catalysts showed a 9× increase in conversion as compared to the pure Ni catalysts at 200 °C and >99% CH_4_ selectivity across a broad temperature range.^257^ Alternatively, Masri and co-workers prepared RuNi/TiO_2_ catalysts with varying particle sizes and Ru:Ni ratios. With decreasing molar ratios, where SAA structures are implicated, almost complete selectivity to CO through the reverse water–gas shift (RWGS) reaction over a broad temperature range was achieved.^258^ Both studies propose mechanisms to explain the observations, with Su et al. suggesting that CO* dissociation is facile at Ni–Ru interfaces, while Masri et al. suggest CO* desorption is facile at the same site. Interestingly, Masri and co-workers argue that CH_4_ formation is coincident with extended Ru domains forming. This apparent distinction in behavior highlights the challenge of defining structure–function relationships for SAAs where local metal structures are likely dependent on synthesis, pretreatment, and reaction conditions.

The one-step conversion of furfural to tetrahydrofuran (THF) was investigated by Li and co-workers. Specifically, Pt was alloyed with Pd to minimize decarbonylation, which generates poisoning CO species.^259^ PtPd SAAs were encapsulated in a zeolite, in which electron deficient Pd sites adjacent to isolated Pt atoms were proposed as the active sites. By systematically varying the Pt to Pd molar ratio, Pt_10_Pd_90_@S-1 was identified as optimum, achieving greater than 93% THF selectivity at full furfural conversion, outperforming monometallic Pd@S-1, which reached 100% conversion but only 25.7% THF selectivity due to decarbonylation (Figure 25). They attributed the high performance to improved CO poisoning resistance of Pt_10_Pd_90_@S-1, which was demonstrated through H_2_–D_2_ exchange and furan TPD in the presence of CO, which together indicated furan hydrogenation prior to desorption.

**Figure 25:**
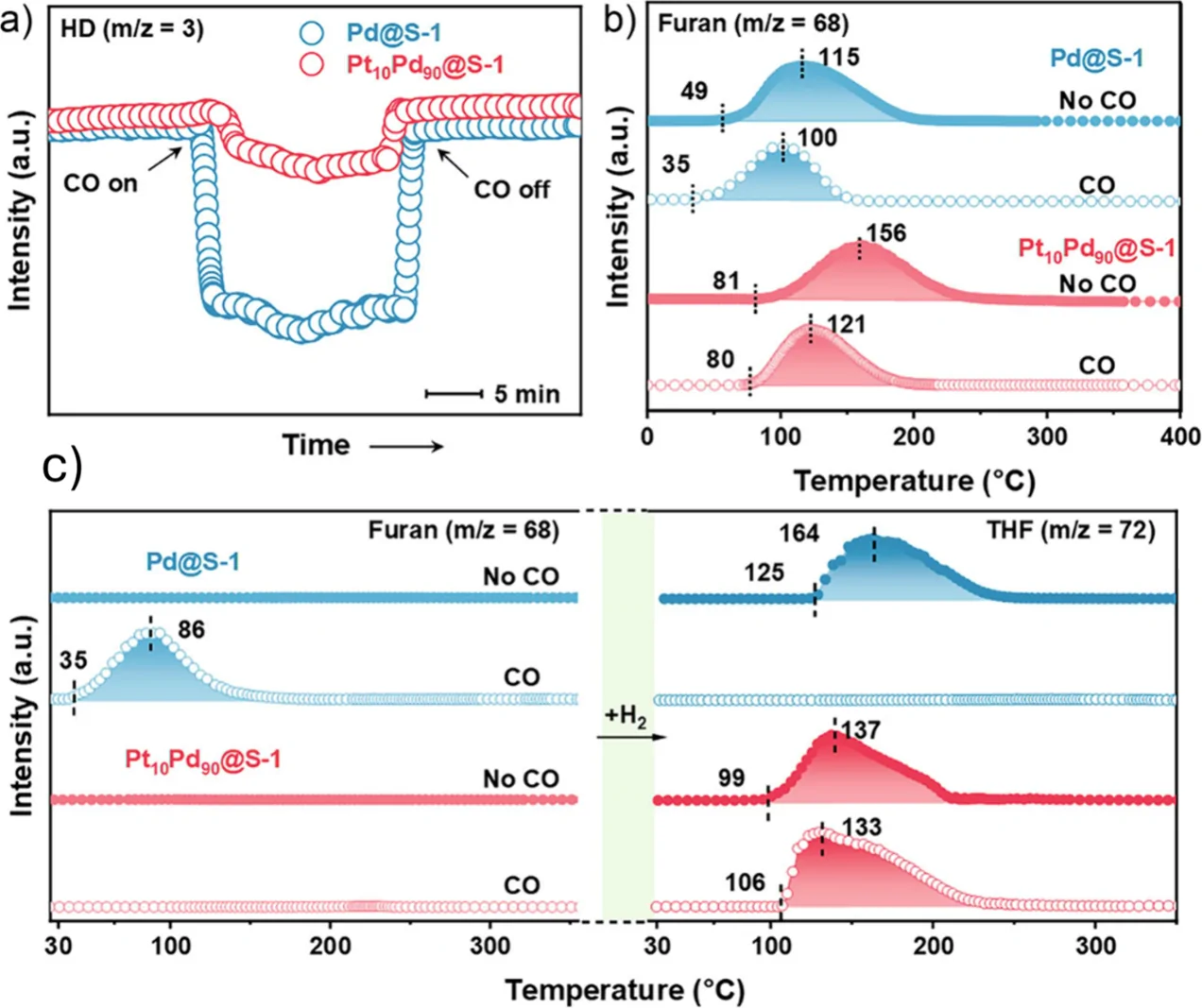
a) H_2_–D_2_ exchange experiments in the presence of CO over Pd@S-1 and Pt_10_Pd_90_@S-1. b) Furan-TPD experiments in the absence and presence of CO over different catalysts. c) Furan- temperature programmed surface reaction spectroscopy (TPSR) experiments in the absence and presence of CO over different catalysts. Adapted with permission from ref.^259^ Copyright 2025 Wiley-VCH GmbH.

This section highlights a substantial focus in the literature on SAAs comprising two reactive metals, with most reports claiming that both the single atom geometry and charge transfer between the elements are important for distinct reactivity. For this class of materials, most authors propose that the catalytic cycles occur at or just around the single atom site, rather than through spillover mechanisms commonly proposed for coinage metal hosts. Studies where catalyst composition and particle size were varied particularly stand out as instructive on how the composition and structure of active sites can substantially influence their reactivity.^255^

#### 3.2.3 Small Ensemble Effects: Beyond the Single-Atom Limit

Although the focus of this review is on the reactivity of SAA catalysts, oftentimes in the study of SAAs authors have concluded that very small ensembles of dopants are more advantageous (e.g., for C–C bond cleavage).^260^ This section will highlight comparisons between ensembles or clusters of reactive metals in the alloy with SAAs, with particular focus on Pd as the reactive metal.

Eagan and co-workers examined CO_2_ hydrogenation to methanol over PdIn alloys with Pd in various structures synthesized *via* galvanic replacement (Pd_1_In_100_: isolated atoms, Pd_1_In_50_: small clusters, and Pd_1_In_5_: extended clusters).^261^ Catalytic performance was compared at a constant 0.5% conversion for all catalysts, and Pd_1_In_50_ showed the highest activity with MeOH selectivity reaching 95%, compared with 68% for Pd_1_In_100_ and 93% for Pd_1_In_5_. Pd_1_In_50_ also exhibited the lowest activation energy of 23 kJ/mol, compared to 32 kJ/mol on Pd_1_In_5_ and 50 kJ/mol on Pd_1_In_100_. On the basis of prior studies, they attributed the performance of Pd_1_In_50_ to 1) Pd clusters providing bridge/hollow sites on which H* was stabilized,^45,145^ 2) short Pd–Pd distances enabling facile H* delivery to neighboring Pd atoms,^105,242^ and 3) smaller clusters exposing a higher density of Pd–In interfaces.^262–264^ While these arguments are reasonable, they were not substantiated by H_2_–D_2_ exchange experiments that elucidate H* behavior (dissociation, diffusion, and recombination) or complementary theoretical studies.

Madix and co-workers varied the Pd:Au molar ratio from 1:25 to 1:8 in dilute PdAu nanoparticles and tested their reactivity for liquid phase benzaldehyde hydrogenation to benzyl alcohol (BOH).^265^ Pd_10_Au_90_ exhibited optimum performance, balancing high selectivity and activity (95% BOH selectivity and a site-time yield of 434 mmol_(BOH)_s^–1^ mol_(Pd)_^–1^), corresponding to the molar ratio where some Pd atom ensembles are present at the Au surface. DFT calculations and kinetic parameter measurements demonstrate that small Pd ensembles provide the optimal binding strength for the reactant and intermediate, without driving hydrogenolysis that occurs on larger Pd ensembles. Similarly, in the one-pot hydrogenation of nitroarenes and aldehydes, a separate study showed that the size of Pd ensembles on Au nanoparticles dictates selectivity toward condensation products. Using a series of well-defined Pd-in-Au catalysts ranging from single atoms to trimers, the highest selectivity was achieved with Pd single atoms (Pd_2_Au_98_/SiO_2_), which selectively hydrogenated nitrobenzene with little reactivity toward benzaldehyde. Theoretical calculations confirmed that larger Pd ensembles increase benzaldehyde adsorption energies, competing with nitroarene reduction and reducing selectivity. Together, these studies highlight that controlling the nanoscale Pd ensemble size on Au surfaces is crucial for optimizing both activity and selectivity in multicomponent hydrogenation reactions.^266^

Finally, Duan and co-workers examined the influence of Pd ensemble size for propyne semihydrogenation on PdCu bimetallic catalysts.^267^ When Pd was present as ensembles (Pd_0.1_Cu), a combination of high selectivity (even in a propene-rich feed) to propene and high activity was observed, while either decreasing Pd content to the single atom regime or much higher Pd content caused decreased selectivity (Figure 26). DFT indicated that Pd ensembles lower the H_2_ dissociation barrier, and calculated Gibbs free energies showed that Pd ensemble surfaces favored propylene desorption, whereas larger Pd domains promoted overhydrogenation.

**Figure 26:**
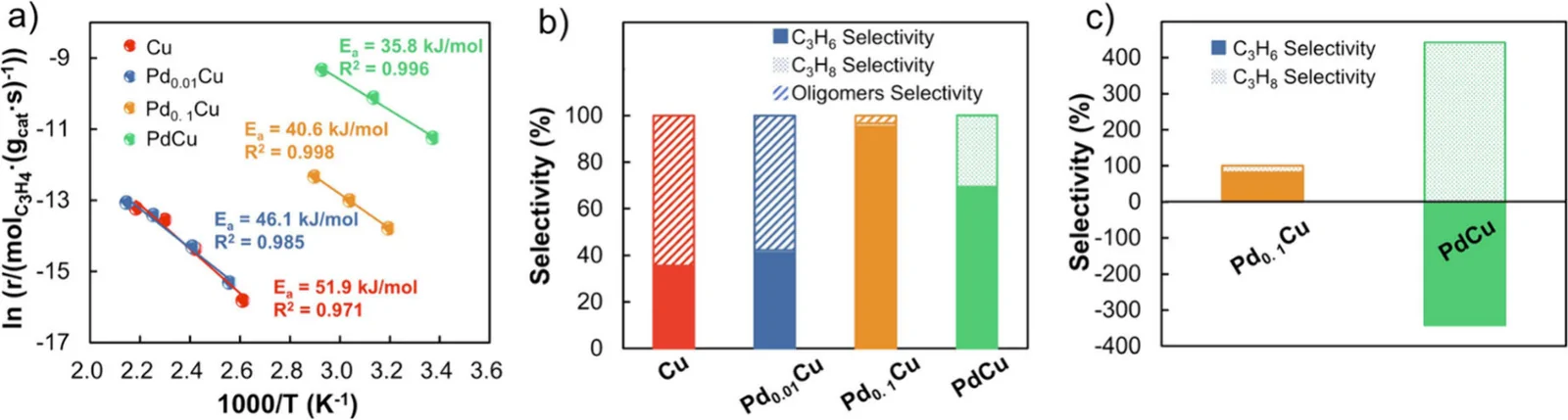
a) Arrhenius plots for propyne conversion, b) comparison of the product selectivities over the Cu and Pd_*x*_Cu catalysts, and c) comparison for the product selectivity over the Pd_0.1_Cu and PdCu catalysts in the case of a propylene-rich feed. Adapted with permission from ref.^267^ Copyright 2025 Wiley-VCH GmbH.

These studies show that SAA structures are not always optimal for every reaction, and in some cases dilute alloys with small dopant (e.g., Pd) ensembles are preferred. However, in order to fully utilize these effects, additional work is needed to clarify the synthetic approaches and reaction conditions that stabilize dopant ensembles with targeted sizes.

### 3.3 Dehydrogenation

Selective dehydrogenation reactions involving alkanes and oxygenates are industrially important and rely on catalysts that selectively cleave C–H or O–H bonds while suppressing unwanted side reactions such as hydrogenolysis, C–C scission and coking.^268,269^ SAAs offer a strategy to utilize isolated reactive metal atoms to selectively activate targeted bonds while enabling facile product desorption from surrounding host coinage metal surfaces. By contrast, ensembles of reactive metals often facilitate undesired C–C hydrogenolysis reactions and coking.^57^ Consequently, reactive single atom composition, local environment (e.g., degree of site isolation and site oxidation) and restructuring under reaction conditions influence the reactivity of SAAs for dehydrogenation reactions. This section reviews reports of dehydrogenation reactions on SAAs, mostly those published since 2020. We first outline why SAAs comprising an active metal dopant on a coinage metal host are effective for dehydrogenation and then discuss recent reports of SAAs composed of two active metals. Finally, we will discuss IMCs featuring single atom-like active sites, similar to SAAs.

#### 3.3.1 Dehydrogenation Controlled by Isolated Metal Site and Local Active Site Structure

As a representative industrially important selective dehydrogenation reaction, propane dehydrogenation involves selective activation of C–H bonds in propane to produce propene and H_2_.^270^ In contrast, overdehydrogenation accelerates coke formation, lowers propene selectivity and causes catalyst deactivation.^271^ Experimental studies on alkane dehydrogenation over SAA catalysts date back to 2018, when both the Sykes/Flytzani-Stephanopoulos and Gong groups published on selective alkane dehydrogenation on Pt–Cu SAAs.^38,68^ These studies demonstrated the importance of the SAA structure for optimal reactivity but most importantly stability with respect to coking and highlighted that the Pt site provides C–H bond activation energetics that enable the initial dehydrogenation reaction without subsequent C–C or C–H activation. These two studies motivated continued SAA research that has since explored a wide range of compositions and addressed mechanistic questions.

Sykes and co-workers used DFT-based catalyst composition screening to identify that isolated Rh sites in RhCu(111) SAAs have an almost identical C–H activation energy to pure Pt(111), while also exhibiting much higher coke resistance than Pt(111).^56^ Consistent with this prediction, experiments showed that RhCu/SiO_2_ SAAs (Rh:Cu molar ratio of 1:100) exhibit ∼ 100% propene selectivity, higher activity on a per mole precious metal basis than Pt/Al_2_O_3_, and better stability than Pt/Al_2_O_3_ (Figure 27). This was a surprising result that illustrates the novel reactivity offered by the SAA structures because Rh is traditionally used for hydrogenolysis, not dehydrogenation.

**Figure 27:**
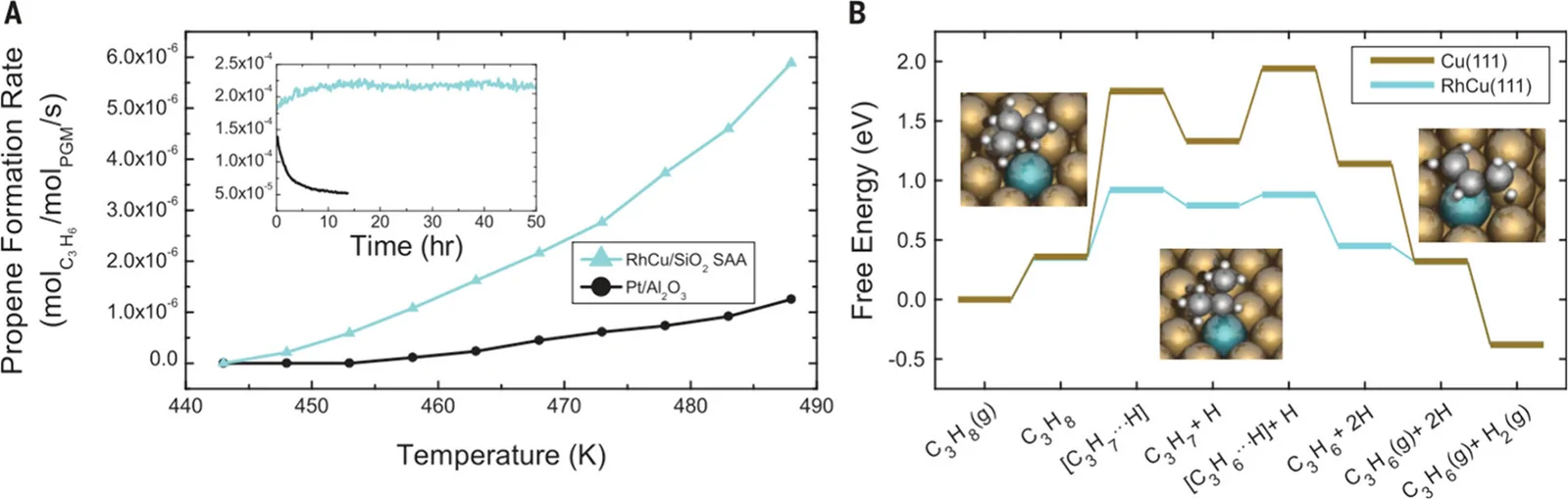
Propane dehydrogenation on RhCu SAAs. (A) Propene formation rate vs temperature for the nonoxidative propane dehydrogenation reaction on RhCu/SiO_2_ SAA (Rh:Cu 1:100) and Pt/Al_2_O_3_ catalysts. (B) Free-energy diagram of RhCu(111) SAA and Cu(111) for the nonoxidative dehydrogenation of propane. Free energies were calculated at 500 K for a C_3_H_8_ and H_2_ partial pressure of 1 kPa and a C_3_H_6_ partial pressure of 0.01 Pa. Adapted with permission from ref.^56^ Copyright 2021 AAAS.

Various theoretical studies have since examined alkane dehydrogenation mechanisms on Cu-based SAAs and used computational screening to identify additional promising compositions. Gong and co-workers previously reported that PtCu SAAs offer high propene selectivity and low coke formation rates in propane dehydrogenation, yet the origin of this performance remained unresolved.^68^ To further analyze the alkane dehydrogenation mechanism on PtCu SAAs, DFT calculations were combined with KMC simulations to interrogate a 54-step reversible reaction network that includes adsorption, desorption, C–H scission, and C–C cracking.^272^ The detailed reaction network confirmed the original mechanistic hypothesis that high selectivity derives from facile initial C–H activation and propene desorption and high barriers for subsequent C–H and C–C activation prevent coking. A H_2_ cofeed was suggested to further mitigate coking.

Cheng and co-workers used DFT to compare RhCu and PdCu SAAs for propane dehydrogenation with a focus on where reactive dopants localize in the nanoparticle surface without adsorbed species.^273^ Rh was predicted to localize at Cu(111) surface sites, while Pd preferred to reside at steps and edges. Dopant atoms at these undercoordinated sites were predicted to be more reactive for propane dehydrogenation than dopants at Cu(111) sites. While this was not experimentally demonstrated, this work highlights an important question for SAA catalysts: what is the distribution of dopant coordination environments under reaction conditions, and how is it influenced by the presence of adsorbates?

Zhang and co-workers used DFT calculations to compute 92 C–H bond cleavage energy barriers for propane dehydrogenation and after training a regression model on 12 descriptors, predicted the C–H activation barriers of 53 SAAs.^274^ RuCu(111) was identified as the most promising propane dehydrogenation catalyst, with a lower first C–H barrier compared to Pt(111) (0.36 vs 0.55 eV) and a similar second C–H barrier (0.64 vs 0.68 eV), while also increasing the barrier for propylene over dehydrogenation (1.00 vs 0.79 eV) and suppressing C–C scission. Feature analysis suggested that this barrier reduction is driven mainly by the surface energy of the dopant, with comparatively smaller influences of the host metal. Zhang and co-workers performed a dual-descriptor (C_2_H_4_ desorption energy and C_2_H_5_ adsorption energy) DFT based screening across 15 SAAs for ethane dehydrogenation, identifying that NiCu decreased the C–H activation barrier compared to other SAAs while maintaining balanced adsorption–desorption strengths, thereby likely inhibiting the coke-forming pathway.^275^ Although both studies identify promising SAAs, no direct experimental comparisons have been made for SAAs of different composition (e.g., PtCu, RhCu, RuCu and NiCu). These theoretical papers collectively show that SAAs can enable high alkene selectivity, whereby the isolated dopant sites promote C–H activation while the Cu host binds intermediates and products moderately, thereby suppressing C–C cracking and coking.

Under propane dehydrogenation conditions at relevant temperatures (>500 °C), Cu-based catalysts are prone to sintering.^56^ Gong and co-workers followed up on their previous study and interrogated the effect of replacing the SiO_2_ supports with copper phyllosilicate (CuSiO_3_) for PtCu SAA propane dehydrogenation catalysts containing 0.1 wt % Pt and 7 wt % Cu, corresponding to a Pt:Cu molar ratio of 1:215.^69^ Upon reduction at 680 °C on the CuSiO_3_ support, Cu^+^–O–Si interface sites formed at the surface-metal interface, strengthening the interaction between the Cu nanoparticle and the SiO_2_ matrix and thereby reducing sintering. This thermally activated SMSI-like interaction partially embedded the PtCu nanoparticles within the support and helped preserve the Cu host particles that isolate Pt atoms. Interestingly, when reduced at 580 °C, formation of the Cu^+^–O–Si interface was insufficient and Cu sintering proceeded. However, upon reduction at 680 °C, 0.1Pt7CuSiO_3_ delivered 42% conversion with 93% propene selectivity for at least 30 h at 580 °C. This work demonstrates the importance of the support composition and catalyst pretreatment to optimize SAA performance, especially for reactions performed at higher temperatures.

Using a similar pretreatment focused approach, Chen and co-workers used sequential heat treatment in air and H_2_ to convert carbon derived from the Cu and Pt catalyst precursors into a thin carbon layer that coated the PtCu SAA.^276^ The Pt to Cu atomic ratio was optimized and the carbon-layer-encapsulated Pt_1_Cu_30_ SAA exhibited 43.5% conversion and 98.2% propene selectivity after 32 h of testing at 600 °C. The authors proposed that the excellent catalytic performance primarily stemmed from electron transfer from the carbon layer to the single Pt atom, which weakened propylene binding, lowered the barrier for the desired second C–H step and raised the barrier for further dehydrogenation, thereby suppressing coking. However, the nature of the C-covered catalytic surfaces remains in question: specifically, how the carbon layer could influence Pt sites without substantially blocking them.

Other researchers have explored how supports and overlayers on SAA nanoparticles can be used to influence their reactivity for alternative alkane dehydrogenation reactions. Methylcyclohexane dehydrogenation to toluene and H_2_ is interesting for the development of liquid organic hydrogen carriers (LOHCs). For example, Satsuma and co-workers designed a complex catalytic structure starting with a ∼ 1 nm Fe overlayer on a Cu core and then deposited Pt on the surface *via* galvanic replacement.^277^ The Pt loading was kept low (0.1 wt %) and the composition was optimized (Cu/(Cu+Fe) = 0.6) to maximize H_2_ evolution to higher rates than previously reported for Pt based intermetallics. The authors propose that excellent performance was due to effective isolation of Pt sites by the thin Fe overlayer on the Cu core.

Nonoxidative alcohol dehydrogenation has been another major application for SAA catalysts. In ethanol dehydrogenation, the desired pathway is selective H removal to form acetaldehyde and H_2_, whereas undesired routes include C–O activation, leading to dehydration to ethylene or ether, as well as deeper decomposition pathways that generate CO or CH_4_ through C–C scission. Early studies established NiCu and NiAu SAAs as excellent ethanol dehydrogenation catalysts that enable acetaldehyde formation at substantially lower temperatures than the corresponding pure coinage metal catalysts while suppressing C–C cleavage relative to Ni rich surfaces.^90,95,260^ Building on these foundations, subsequent work has expanded the range of host-dopant SAA compositions and clarified how reactive site isolation governs activity, selectivity and stability.

Friend and co-workers resolved the coking problem in ethanol dehydrogenation over NiCu SAAs by changing the pretreatment from H_2_ to O_2_, highlighting again the importance of pretreatment in dictating SAA structure and activity.^278^ While dispersed Ni species served as active sites for C–H bond cleavage, H_2_ pretreatment at 350 °C induced the formation of Ni clusters, which led to C–C bond scission and promoted coking. In contrast, O_2_ pretreatment at 250 °C generated a CuO overlayer that kinetically trapped Ni^2+^ in the subsurface, suppressed Ni aggregation and confined the chemistry to selective C–H activation and conferred long-term stability. However, the analysis did not include a systematic variation in Ni:Cu molar ratio, thus raising questions about the role of the SAA structure vs the oxidation state of the surface.

The mechanism of nonoxidative ethanol dehydrogenation to acetaldehyde on Cu-based SAAs was interrogated by Sykes and co-workers using Ni_0.006_Cu/SiO_2_ and a model NiCu(111) surface prepared by dosing 1% of a monolayer Ni into Cu(111).^279^ Kinetic isotope effect (KIE) measurements using Ni_0.006_Cu/SiO_2_ demonstrated a pronounced k_H_/k_D_ value for C_α_–H (C_α_–D) substitution and almost no effect for O–H (O–D) substitution, suggesting C_α_–H activation at isolated Ni atoms was the primary rate-determining step. Ethanol TPD measurements demonstrated lower temperature acetaldehyde desorption (30 K) and higher yields (12x) on NiCu(111) than on Cu(111), consistent with a decreased barrier for the first C_α_-H scission at Ni sites. In repeated TPD measurements on NiCu(111), acetaldehyde yield increased cumulatively with TPD cycles, indicating that the ethoxy intermediates formed on Ni subsequently spilled over to adjacent Cu, thereby regenerating Ni sites for reuse. At higher Ni surface coverages where Ni ensembles form, ethanol decomposition and coke-related site blocking were observed, reinforcing that selectivity requires the SAA structure.

In a follow-up study, Sykes and co-workers compared NiCu(111) with RhCu(111) SAAs for ethanol dehydrogenation and showed that changing only the dopant on the same Cu host surface redirected the reaction pathways.^91^ Specifically, the RhCu(111) SAA lowered the C–C cleavage barrier, producing CH_4_ and CO alongside acetaldehyde and thereby decreasing acetaldehyde selectivity. In contrast, on the NiCu(111) SAA, the comparatively higher C–C scission barrier leads the C–H pathway to dominate and thus preserves high acetaldehyde selectivity, consistent with the previous report. This comparison further emphasizes that dehydrogenation reactions on SAAs require careful dopant element choice that dictates which bond cleavage events occur and hence how selectivity changes.

Fredersdorff et al. reported the synthesis of Pd–Cu single-atom alloys, consisting of isolated Pd atoms dispersed within Cu nanoparticles supported on Al_2_O_3_/NiAl­(110). Density functional theory calculations indicated that these atomically dispersed Pd species acquire a negative partial charge through interaction with the Cu host. Across a broad range of Pd loadings, the catalysts exhibit near-unity selectivity for butanol dehydrogenation to butanal, a behavior that is not reproduced on single-crystalline PdCu(111), underscoring the distinct reactivity of isolated Pd sites in the SAA regime.^226^

To explore promising SAA combinations for ethanol dehydrogenation beyond Cu-based systems, Haider and co-workers established a machine learning framework to predict intermediate binding energies, effectively identifying candidates such as NiAu and NiAg.^206^ While this approach accelerated catalyst screening, their subsequent study revealed that single atom sites often deviate from traditional scaling relations, which can lead to inaccuracies in standard catalytic models. To address this, the group developed a more precise, nonscaling model based on full DFT parameters that successfully reproduced experimental trends. This refined modeling further clarified the mechanistic competition between initial O–H and α-C–H activation pathways, highlighting the critical role of site-specific energetics. Collectively, this work demonstrates how machine learning can drive catalyst discovery while emphasizing the necessity of high-fidelity modeling to capture the unique electronic behavior of isolated atom active sites.^280^ In related work on alcohol dehydrogenation, in a study of the partial oxidation of 1-phenylethanol to acetophenone over Pd-in-Au SAA catalysts,^281^ isolated Pd atoms were found to exhibit turnover frequencies up to 2 orders of magnitude higher than those of pure Pd or Au catalysts, demonstrating the significant enhancement in catalytic activity achieved through SAA formation and supported by theory.

Identifying the oxidation state of reactive sites under reaction conditions is essential for understanding the working mechanism of SAA catalysts. Sykes and co-workers used *operando* high energy resolution fluorescence detection (HERFD) X-ray absorption near edge structure spectroscopy (XANES) to show that, upon ethanol adsorption from room temperature to 150 °C, isolated Ni atoms in a NiAu SAA acquired a positive partial charge.^58^ As ethanol desorbed at 200 °C, the Ni K-edge shifted back toward the reduced state, indicating a reversible decrease in this partial positive character. These results demonstrate that the oxidation state of isolated Ni active sites is reversibly modulated by the reactive environment, in a manner reminiscent of single site homogeneous catalysts when ligands (de)­attach, an important consideration for reactions involving oxygenates or oxidants.

The small size of SAA active sites limits their ability to stabilize multiple intermediates or transition states in larger molecules, which may limit their utility (see also Section 2.5).^44^ To overcome this limitation, Montemore and co-workers proposed DAAs in which, for example, Pt–Cr dimers are embedded in Ag(111). They identified these elements using DFT calculations and performed TPD experiments of ethanol dehydrogenation and dehydration.^175^ Pt–Cr dimers were chosen as they were predicted to form stable heterodimers by DFT calculations. By varying dopant coverage in the experimental measurements, PtAg and CrAg were demonstrated to be essentially inactive toward ethanol, even up to 10% of a monolayer of each dopant. Alternatively, PtCrAg became active and highly selective to acetaldehyde at about 3% of a monolayer Pt and 3% of a monolayer Cr. Interestingly, higher dopant coverages shifted selectivity toward ethylene, reaching about 80% at 10% of a monolayer. Figure 28a summarizes the key mechanistic picture. A single dopant atom cannot stabilize both fragments during O–H scission of ethanol; PtAg only favors adsorption of H while CrAg only favors ethoxy adsorption, whereas a PtCr dimer stabilizes both the H and ethoxy species thereby lowering the barrier toward acetaldehyde formation. Figure 28b further shows that larger PtCr ensembles facilitate C–O cleavage, promoting dehydration and shifting selectivity toward ethylene at higher dopant coverages beyond the dimer limit. These effects have recently been demonstrated to translate to high surface area materials at catalytically relevant pressure and temperature. Specifically, trimetallic Pt_*x*_Cr_*x*_Ag_1–2x_ alloy films (x≤0.1) were designed to stabilize isolated Pt–Cr pair ensembles within an Ag matrix for nonoxidative ethanol dehydrogenation. *Operando* XAS confirmed the formation and stability of Pt–Cr pairs, while ambient-pressure XPS showed that these sites are highly active for ethanol dehydrogenation.^178^

**Figure 28:**
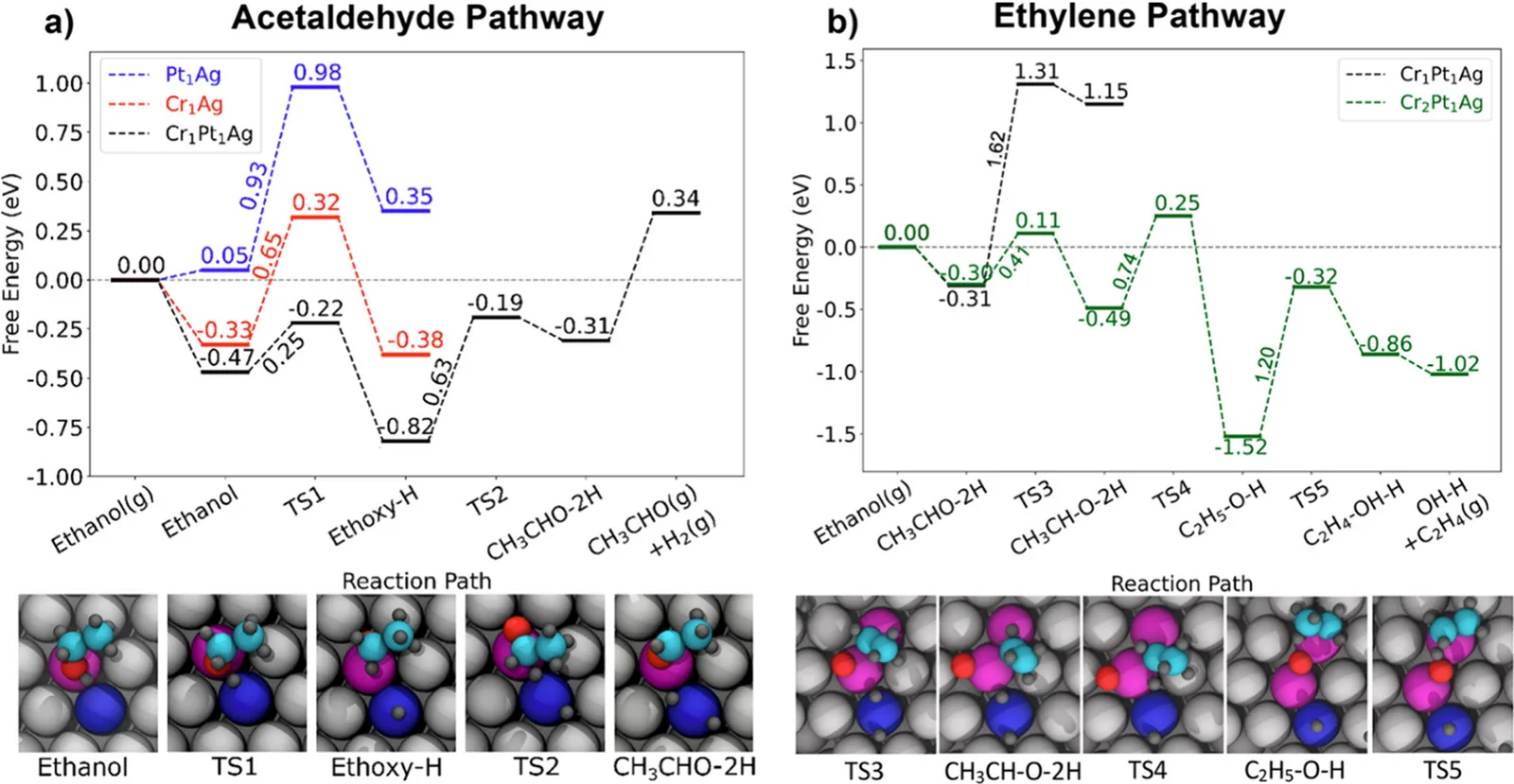
DFT-calculated energetics of (a) ethanol dehydrogenation to acetaldehyde on Pt_1_Ag, Cr_1_Ag, and the Pt_1_Cr_1_Ag DAA and (b) further reaction to ethylene on Pt_1_Cr_1_Ag and Pt_1_Cr_2_Ag. The Pt_1_Cr_1_ dimer site kinetically and thermodynamically facilitates initial O–H bond breaking significantly more than the respective SAAs, and the Pt_1_Cr_2_ trimer site facilitates C–O bond cleavage. Images of intermediates and transition states along the pathways are shown. Reproduced from ref.^175^ Copyright 2023 The Authors. Licensed under CC-BY 4.0.

In a study of the hydrodechlorination of 1,2-dichloroethane over Pd active sites on Ag,^282^ isolated Pd atoms were found to favor selective ethylene formation, whereas contiguous Pd ensembles promoted ethane production, highlighting the importance of active-site architecture in bimetallic catalysis.

Related to alcohol dehydrogenation, reactions of adsorbed methanol or methanol fragments (e.g., methoxy, hydroxyls species) were modeled by Stamatakis and co-workers to correlate SAA composition with specificity of oxygenate bond activation and formation.^283^ For example, Ag based SAAs were identified as promising for selective conversion of methanol to formaldehyde whereas PdAu SAAs are promising for dimethyl ether formation from methanol. Broad trends across all SAAs and functional groups demonstrated deviation from the scaling behavior of pure metals with SAAs, enabling low activation energies and weak binding across various transformations of oxygenates. This offers useful predictions for the synthesis and testing of new SAA catalysts for a range of important chemical conversions.

Another example of using metal–support interactions to influence SAA performance was reported by Liu and co-workers in the context of RuNi SAAs on Ti_3_C_2_T_*x*_ MXene supports (T_*x*_ is OH, O, or F surface termination) for ammonia borane hydrolysis (NH_3_BH_3_ + 2H_2_O → NH_4_^+^ + BO_2_^–^ + 3H_2_).^284^ The Ru structure was varied from nanoparticles, single atoms (i.e., SACs), and SAAs (alloyed with Ni) on the Ti_3_C_2_T_*x*_ support, and the RuNi SAA with 1:20 Ru:Ni molar ratio was found to exhibit the highest reactivity on a per Ru mass basis (Figure 29 **a,b**). This was attributed to the combined influence of charge transfer from the support and alloying that creates Ru atom sites with optimal electronic structure (Figure 29) and binding energies of kinetically relevant reaction intermediates.

**Figure 29:**
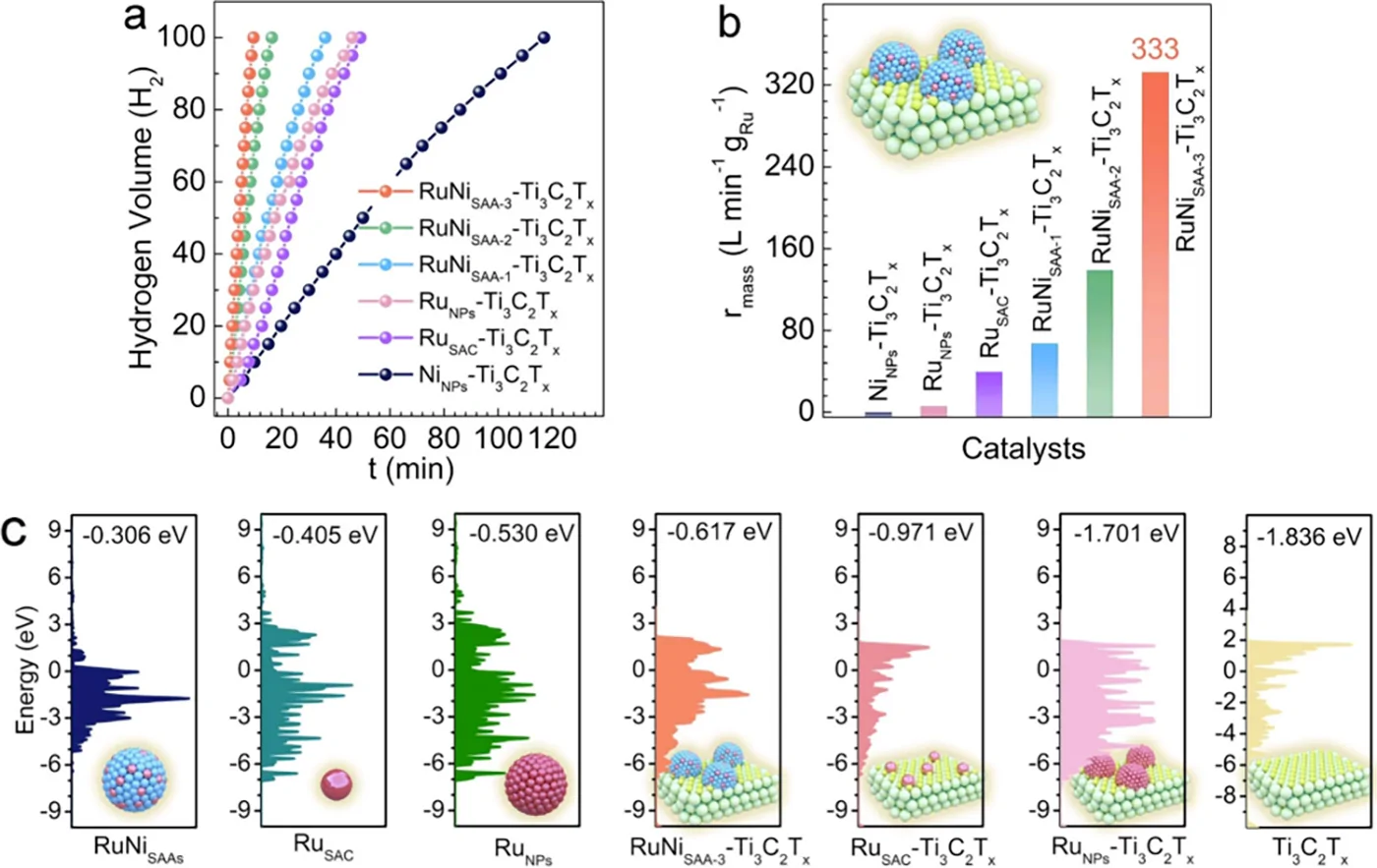
Catalytic performance test for ammonia borane hydrolysis. a) Hydrogen generation over time, b) corresponding r_mass_ value for RuNi_SAA‑x_–Ti_3_C_2_T_*x*_, Ru_NPs_–Ti_3_C_2_T_*x*_, Ru_SAC_–Ti_3_C_2_T_*x*_, Ni_NPs_–Ti_3_C_2_T_*x*_, and c) PDOS plots with d-band center position of Ru in RuNi_SAA_, Ru_SAC_, Ru_NPs_, RuNi_SAA‑3_–Ti_3_C_2_T_*x*_. Adapted with permission from ref.^284^ Copyright 2023 Wiley-VCH GmbH.

To summarize, alkane and alcohol dehydrogenation reactions continue to be intensely studied on SAAs (along with other dehydrogenation reactions), with reports detailing the influence of SAA composition, how reactive metal ensemble formation affects selectivity, and broader catalyst optimization including the use of metal–support interactions and dual atom dopant formation to enhance performance. In general, these reactions proceed from initial C–H activation at the reactive dopant sites, with the dopant and host composition being controlled to minimize unwanted C–C, C–O (for alcohols) or deeper dehydrogenation reactions. Many of the studies were computational, providing various interesting hypotheses and predictions for future experimental interrogation.

#### 3.3.2 Extending the SAA Concept: Atomically Dispersed Sites in Supported Catalytically Active Liquid Metal Solutions and IMCs

This section covers emerging SAA motivated catalyst designs based on supported catalytically active liquid metal solutions (SCALMS) and IMCs. Interestingly, these seemingly disparate classes of materials are analogous to SAAs and have been extensively reported for alkane dehydrogenation with high activity and low coking rates by virtue of site isolation and the mobility of the dopants between the surface and bulk.^285,286^ There is a rich degree of structural complexity in these systems; for example, as the Pt:Ga molar composition varies from very low (e.g., 1:100) to stoichiometric (e.g., 1:2), the thermodynamically preferred state of the catalytic materials under dehydrogenation conditions varies from liquid metal (SCALMS) to well-defined IMCs. At both ends of this spectrum Pt can be site isolated with surrounding Ga, albeit in quite different structures.^287^ In another study, Ga–Ni SCALMS were investigated for ethylene oligomerization, revealing that isolated Ni atoms on the liquid metal surface serve as the active catalytic sites.^288^ Combined experimental and theoretical analyses further demonstrated that hydrogen pretreatment enriches surface Ni atoms, enhancing catalytic performance.

Wasserscheid and co-workers studied PtGa based SCALMS at ∼ 50:1 Ga:Pt molar ratios, where Pt is thought to primarily exist as isolated species, for propane dehydrogenation. Experimental analysis of support effects demonstrated that SiC supported PtGa liquid-metal catalysts exhibited higher reactivity than SiO_2_ or Al_2_O_3_ supports during propane dehydrogenation at 500–600 °C, with both high selectivity (>95%) and high reactivity on a per g_Pt_ basis.^289^ Coke formation was attributed primarily due to active sites on the support, which bolstered the argument of Pt isolation which mitigates coke formation that occurs on extended Pt domains. Irreversible reactivity changes were observed, even in the limit of lower temperature reactions where coke formation was essentially nonexistent. Detailed analysis of this system *via* varying Pt:Ga molar ratio, manipulating pretreatment conditions, performing X-ray diffraction (XRD) pair distribution function (PDF) measurements, DFT calculations, and microscopy demonstrate that in addition to the expected single Pt atoms dissolved in Ga, other structures such as oxidized Ga, Ga-rich Pt IMCs and Pt-rich rafts can form.^201,290^ Careful control of pretreatment conditions (generally harsh reduction in H_2_) allowed for optimization of catalytic performance with improved reactivity and stability. Again, this study points to the importance of pretreatment and support optimization to maximize SAA catalyst performance.

Li and co-workers extended these findings and compared various Pt alloys with low melting point metals (In, Sn, Ga, etc.) and found that PtIn catalysts with 1:25 molar ratio exhibited optimum propane dehydrogenation performance (rate and selectivity) at 600 °C.^291^ The optimal Pt performance was proposed to be due to charge transfer between In and Pt as well as suppressed Pt–Pt ensemble formation. Papp and co-workers have further optimized SAA SCALMS for propane dehydrogenation with variations in Ga, In, and Sn based components that form the low melting point liquid metals.^292^ Further, Zhu and co-workers have examined GaPt-based SAA SCALMS for selective hydrogenation chemistry, analogous to the initial demonstration of SAA hydrogenation catalysts.^293^

These examples highlight an interesting translation of the SAA concept to supported liquid metals that enable high reactivity, stability and selectivity for propane dehydrogenation around or below 600 °C. Future examination of how the dynamic structure of liquid metal catalysts influences the reactivity of the dopant atoms and the effect of the reaction environment will be interesting extensions to this area.

IMCs provide another route to site isolation through an ordered lattice, which makes them conceptually adjacent to SAAs and SCALMS. They have been widely explored for propane dehydrogenation, where site isolation in more inert hosts (e.g., the aforementioned Pt in Ga system) and local environment control are key levers for catalytic performance.^294–298^ However, because IMCs often entail intrinsic site to site coupling within an ordered framework and hence different electronic properties, their structure activity relationships can differ from the isolated dopant picture emphasized for SAAs, so we do not discuss them in detail here and instead refer the reader to a dedicated review.^299^

Overall, these studies highlight the interesting opportunities for SAAs in the form of supported liquid metals at reaction conditions. The use of liquid metal hosts (Ga, In, Bi) can overcome the usual high-temperature limitations of SAAs composed of coinage metal nanoparticles that are prone to sintering and enable retained site isolation of reactive elements under harsh alkane dehydrogenation conditions. We note similar coke resistant behavior has been seen for SCALMS with dilute amounts of reactive elements.^300^ Inherent differences in active site structure and dynamics in liquid metals compared to supported metal nanoparticles (which are themselves dynamical) and their influence on reactions will be an interesting area for future fundamental studies.

### 3.4 Selective Oxidation Catalysis

Selective oxidation reactions are responsible for the production of chemicals on the scale of tens of billions of dollars per year. Therefore, optimization of catalyst selectivity and stability is a key goal for more efficient production. Essential to the design of selective oxidation catalysts is controlling the relevant surface species (e.g., atomic oxygen, molecular oxygen, hydroxyls, sulfur oxides, etc.), their location/state (surface, specific surface sites, subsurface, surface oxide), and their reactivity under desired reaction conditions.^301^ While bimetallic catalysts have been extensively explored for selective oxidation catalysis, our previous review paper did not describe selective oxidation chemistry, as this has only recently emerged as another potential application for SAAs.^33,302,303^

Coinage metal catalysts are well-known for selective oxidation, with the ability to activate O_2_ and produce labile reactive oxygen species. The largest scale industrial example is the direct epoxidation of ethylene *via* molecular oxygen on Ag particle surfaces.^304,305^ In this section, we discuss recent work that examined the influence of SAA formation on the reactivity of coinage metal surfaces for molecular oxygen activation and the resulting influence on selective oxidation catalysis. Interestingly, the concept of site isolation that enables facile activation and weak binding in selective hydrogenation chemistry can be directly translated to selective oxidation chemistry.

#### 3.4.1 Oxygen Activation and Selective Oxidation on Cu and Ag based SAAs

The focus of this section is Cu and Ag based SAAs and Au based SAAs will be covered in sections 3.4.2 and 3.4.3. Both Ag and Cu surfaces can dissociate molecular oxygen at catalytic temperatures and pressures, although O_2_ dissociation on Ag is much slower and can be rate limiting in oxidation reactions.^306^ To understand how SAAs may be useful in selective oxidation chemistry, it is first important to consider how dilute alloy formation influences oxygen dissociation rates, oxygen coverages and oxygen speciation as compared to the host coinage metals. To date this topic has only been sparsely discussed in literature.

Beginning with Cu-based systems, Sykes and co-workers investigated how dilute Rh doping affects O_2_ dissociation and the oxidation of Cu on single crystals and supported catalysts over many orders of magnitude of O_2_ pressure.^307^ Using sequential TPD measurements together with STM imaging, they demonstrated that on clean surfaces, Rh atoms in Cu(111) serve as active sites that promote O_2_ dissociation as well as initial oxidation of Cu surfaces in the regions above step edges (Figure 30a**)**. *In-situ* XPS was used to quantify Cu(111) surface oxidation as a function of time and oxygen partial pressure. On RhCu(111) with 1% of a monolayer Rh present as isolated surface atoms, oxide coverage was initially higher than on Cu(111), yet the final degree of oxidation was lower; see Figure 30b. The study extended this analysis to RhCu nanoparticles by loading Rh at 0.01 or 0.06 wt % on Cu nanoparticles and monitoring Cu oxidation at three different O_2_ pressures by *in situ* UV–vis spectroscopy (Figure 30c**)**. Consistent with the surface science studies, initial surface oxidation rates were promoted by Rh, while surface oxidation was suppressed at longer time scales or higher oxygen pressure. DFT studies revealed that Rh stabilized a molecular O_2_ precursor and boosted oxygen uptake on clean surfaces, but ultimately Rh at step edges hindered the lattice rearrangement required for deeper oxidation of Cu. This work illustrated that the influence of SAAs on O_2_ activation on Cu is more complicated than the case of H_2_, largely due to the complexity of oxide structures.

**Figure 30:**
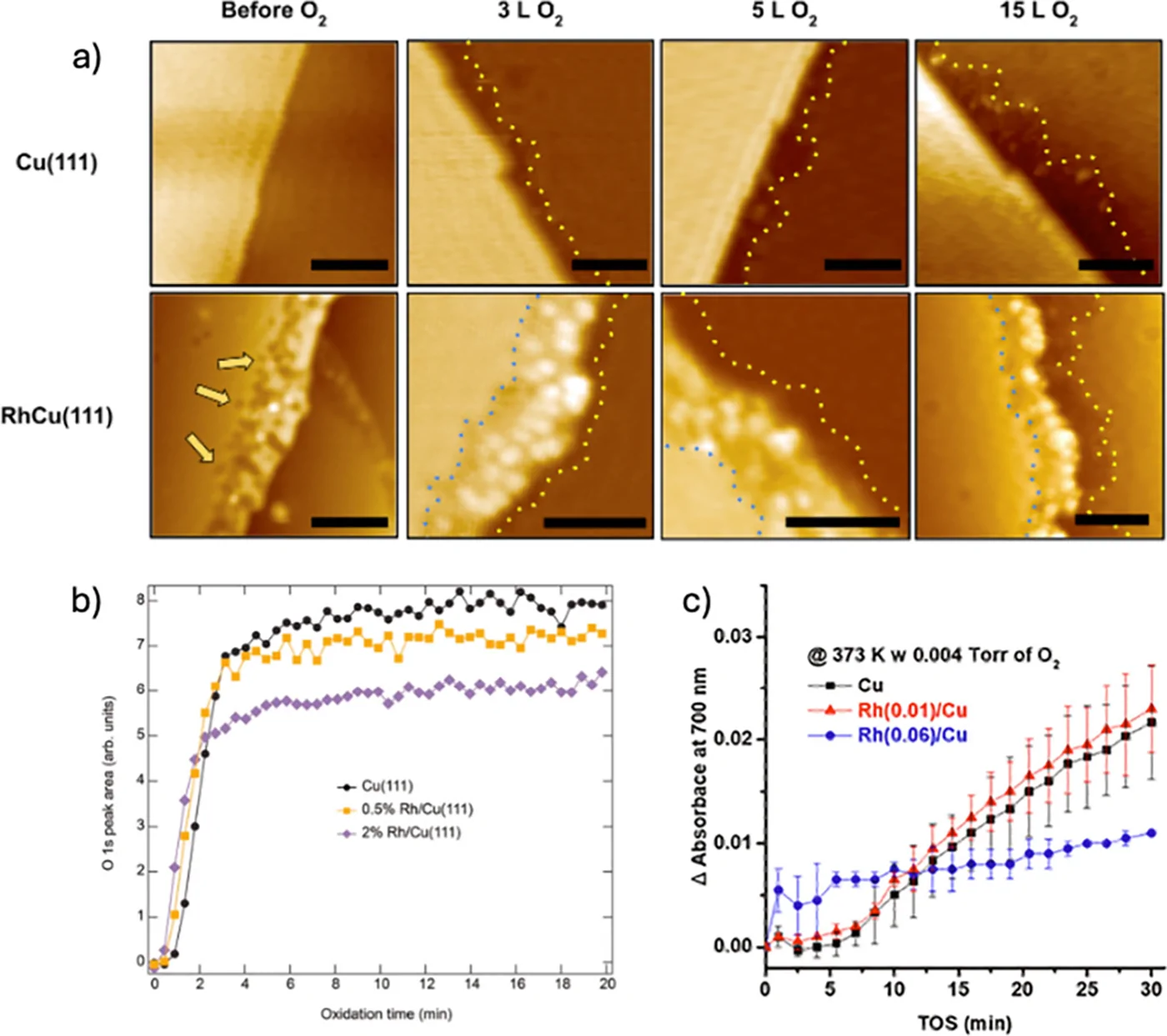
STM data showing oxidation of a) Cu(111) and 0.5% RhCu SAA after subsequent O_2_ exposures of 0, 3, 5, and 15 L (left to right). b) O 1s XPS peak area comparing the extended oxidation of Cu(111) and RhCu(111) during exposure to 5 × 10^–6^ Torr of O_2_ at 400 K. c) Oxidation of Cu and RhCu nanoparticles quantified by UV–vis. The absorbance at 700 nm corresponding to the excitonic feature associated with the band gap of Cu_*x*_O is traced during the oxidation of Cu and RhCu at 0.004 Torr of O_2_ exposures at 373 K. Adapted with permission from ref.^307^ Copyright 2025 Elsevier.

Sykes and co-workers continued these studies by comparing how single Pt and Rh atoms influence the stability and reactivity of thin CuO_*x*_ layers on Cu(111) surfaces.^308^ Sequential TPD measurements of methanol, where methanol oxidation acts to reduce the surface, were used to interrogate the influence of the reactive dopants on oxide surface reactivity. The addition of Pt atoms was found to increase the defect density of the CuO_*x*_ films and thus reactivity, while Rh stabilized ordered CuO_*x*_ layers and depressed reactivity. These two initial works show that SAAs have the ability to both promote and decrease the reactivity of coinage metal surfaces under oxidation conditions, with a careful balance of dopant composition and reaction conditions dictating the nature of the influence on reactivity.

Recent work has demonstrated that Ag-based SAAs can perform selective oxidation reactions. This work was initiated by a DFT screening of dopants in Ag(111) which predicted that single atom Ni sites offer uniquely low O_2_ dissociation energies and relatively weak atomic oxygen binding energies in NiAg SAAs (Figure 31A).^60^ Experimental measurements of O_2_ uptake (dissociation) during exposure to 500 L of O_2_ and the resulting atomic O associative desorption on 1% of a monolayer Ni doped Ag(111) confirmed this finding of facile molecular oxygen activation and spillover to Ag sites (Figure 31B). Complementary cryogenic STM studies on the NiAg(100) SAA directly identified a distinct NiO_2_ species, indicative of O_2_ dissociation at isolated Ni sites, that was absent on pure Ag(100).^309^ Near ambient-pressure XPS measurements of the O 1s region during the exposure of NiAg(111) to 1 Torr of O_2_ with increasing temperature from 325 to 700 K gave a more detailed picture of the types of oxygen formed (Figure 31D). Oxygen speciation was deconvoluted into electrophilic O (∼530.2 eV), so-called Ni-philic O (∼529.3 eV), and nucleophilic O (∼528.5 eV) species. Ni was found to stabilize the nucleophilic O species on the Ag surface, which persisted on the surface until ∼ 700 K as compared to 550 K on pure Ag, indicating that Ni stabilizes O species on the Ag surface at Torr pressures closer to catalytic conditions.

**Figure 31:**
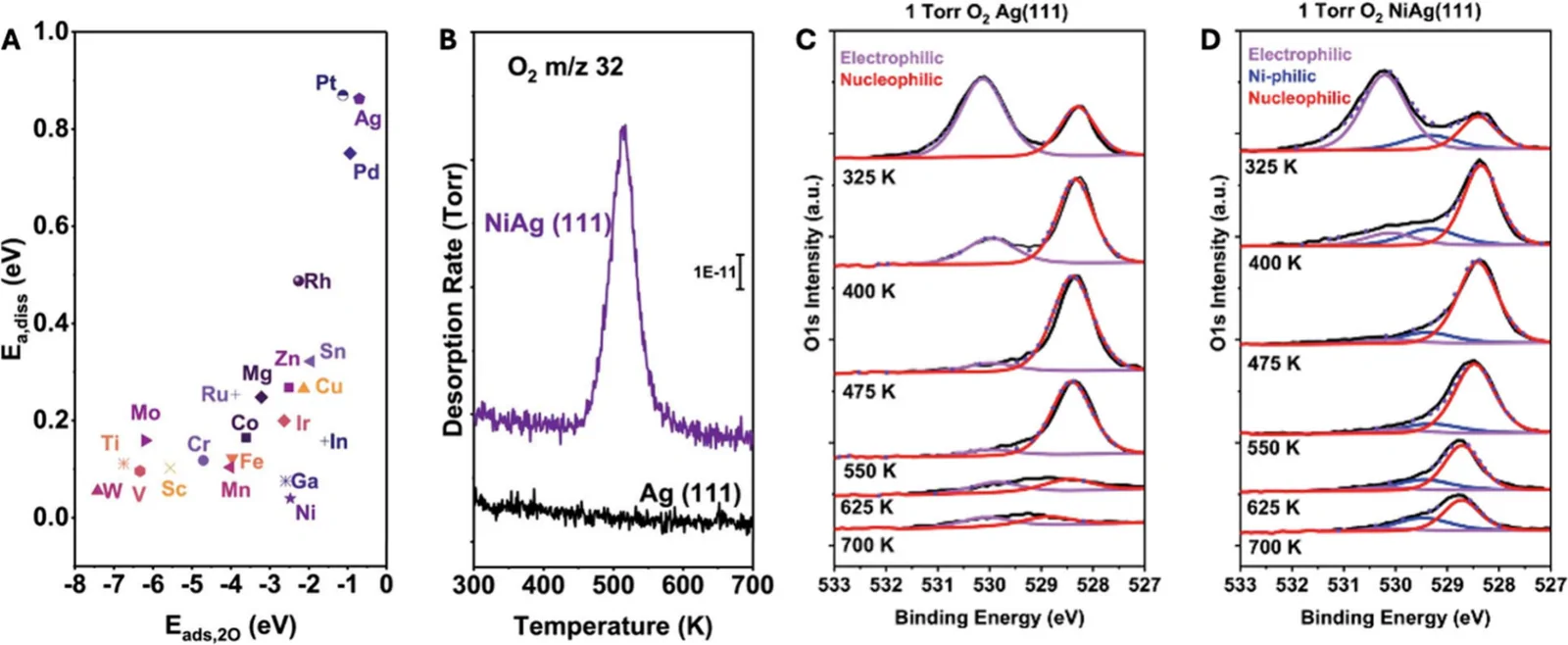
A) DFT calculations of the O_2_ dissociation barrier *E*_a,diss_ as a function of the adsorption energy of the resulting dissociated O atoms for various single atom dopants in Ag(111). B) O_2_ TPD traces for Ag(111) and ∼ 1% NiAg(111) after 500 L of O_2_ dosed at 350 K. C) and D) Electronic structure of oxygen on Ag(111) and NiAg(111) as a function of temperature in 1 Torr O_2_. AP-XPS showing a change in oxygen species from primarily electrophilic oxygen (purple, ∼ 530.2 eV) to more nucleophilic oxygen (red, ∼ 528.5 eV) as the temperature was increased on C) Ag(111) and D) ∼ 5% NiAg(111). Adapted with permission from ref.^60^ Copyright 2025 AAAS.

The findings on Ag SAAs inspired studies of ethylene epoxidation as a function of Ni doping concentration on supported Ag catalysts because O_2_ dissociation can be rate limiting in this reaction. It is well-known that the quantity and reactivity of adsorbed oxygen species on Ag dictates selectivity (between the epoxide and CO_2_) in ethylene epoxidation. Christopher and co-workers synthesized ∼ 75 nm diameter colloidal Ag nanoparticles, added Ni with molar ratios from 1:500 to 1:50 Ni:Ag and examined the influence of Ni on ethylene epoxidation rate and selectivity as seen in Figure 32. They found that NiAg_200_ SAAs with a 1:200 Ni:Ag ratio provided a ∼ 25% EO selectivity increase over unpromoted Ag. Analyzing the rates of EO and CO_2_ formation as a function of Ni concentration demonstrated that trace Ni decreased CO_2_ production rates more than EO production rates and hence increased selectivity to EO. This result can be rationalized by the aforementioned surface science studies which demonstrated that Ni acts to stabilize nucleophilic oxygen, a species widely reported to be unselective for EO synthesis, by reducing its reactivity. The influence of Ni on EO selectivity is only paralleled by the ubiquitously used industrial promoter Cl which also increases selectivity by ∼ 25%, suggesting that SAAs have the potential to substantially promote performance in selective oxidation reactions beyond promoting O_2_ dissociation and spillover.

**Figure 32:**
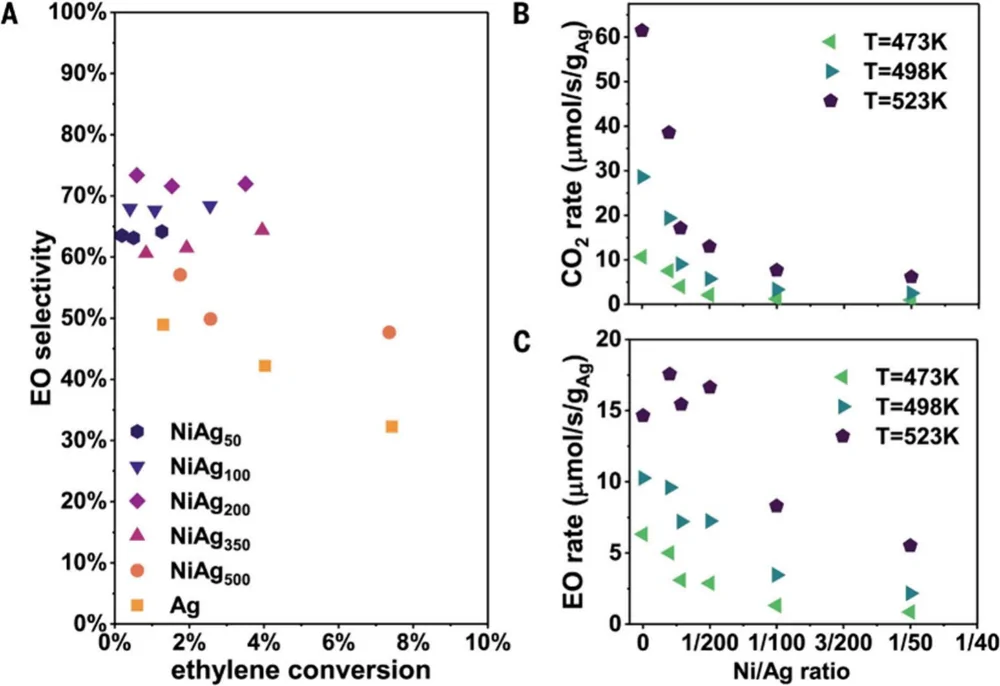
Influence of Ni loading on 5 wt % Ag/α-Al_2_O_3_ catalyst reactivity in 10% C_2_H_4_, 10% O_2_, balance He inlet with 100 to 150 mg catalyst. The Ni:Ag molar ratio was varied from 0:100 (pure Ag) to 1:50 (2% Ni). (A) EO selectivity as a function of steady-state ethylene conversion. The ethylene conversion was varied by changing the temperature between 473, 498, and 523 K, and holding for 2 h to allow the system to reach a new steady state. (B) CO_2_ and (C) EO formation rates at different temperatures as a function of Ni:Ag molar ratio. Adapted with permission from ref.^60^ Copyright 2025 AAAS.

#### 3.4.2 Direct Hydrogen Peroxide Synthesis on SAAs

The direct synthesis of H_2_O_2_ from H_2_ and O_2_ is a prototypical reaction in which control over the O–O bond activation and the reactivity of adsorbed oxidative species is crucial in dictating selectivity. Current hypotheses suggest that selective H_2_O_2_ production requires the formation of OOH* (through proton addition to adsorbed O_2_) whereas unselective H_2_O formation proceeds following O_2_ dissociation.^310,311^ On monometallic surfaces, the adsorption strength of O_2_, the stability of OOH* and the energy barrier for O–O bond cleavage are strongly coupled.^312^ As a result, active sites that adsorb O_2_ and enable OOH* formation also tend to promote O–O bond scission, which reduces H_2_O_2_ selectivity. However, the unique electronic and geometric properties of SAAs provide additional degrees of freedom that can lead to deviations from these linear scaling relationships.^313^ In this section, we focus on Pd-based SAAs for H_2_O_2_ synthesis as there have been a number of promising reports recently.

In the direct synthesis of H_2_O_2_ over PdAu SAA catalysts, Pd is thought to act as the H_2_ dissociation site (as previously discussed for SAA hydrogenation reactions), whereas Au sites bind O_2_ weakly and facilitate *OOH formation. Flaherty and co-workers prepared a composition series of SiO_2_ supported PdAu bimetallic catalysts and systematically varied the Pd to Au atomic ratio from 1:3 to 1:220 to tune Pd site isolation.^302^ Relative to monometallic Pd, Pd_1_Au_220_ increased the H_2_O_2_ selectivity from 19% to 95%, whereas the H_2_O_2_ turnover rate decreased by only about 3-fold, from 0.37 to 0.12 mol H_2_O_2_/s·mol_Pd surface_, suggesting that PdAu SAAs might offer optimal performance for this reaction, Figure 33a. To support this hypothesis, the η^1^-CO*_Pd_ (CO linearly bound atop Pd atoms) stretching frequency during ^12^CO–^13^CO exchange measurements was used to estimate the average distance between isolated Pd sites in the second coordination shell, Figure 33 **b,c**. As the Pd:Au molar ratio decreased there was a decrease in the shift of ^12^CO* during isotope exchange, demonstrating increasing site isolation and thus confirming the beneficial role of the SAA geometry in promoting H_2_O_2_ selectivity. Theoretical analysis suggested that in aqueous solution both H_2_O_2_ and H_2_O formed *via* reduction of a common intermediate OOH*. Increasing Pd isolation in the Au surface destabilized the transition state for the O–O bond cleavage pathway, while the barrier for the nondissociative O–O proton–electron pathway increased only slightly, leading to higher H_2_O_2_ selectivity.

**Figure 33:**
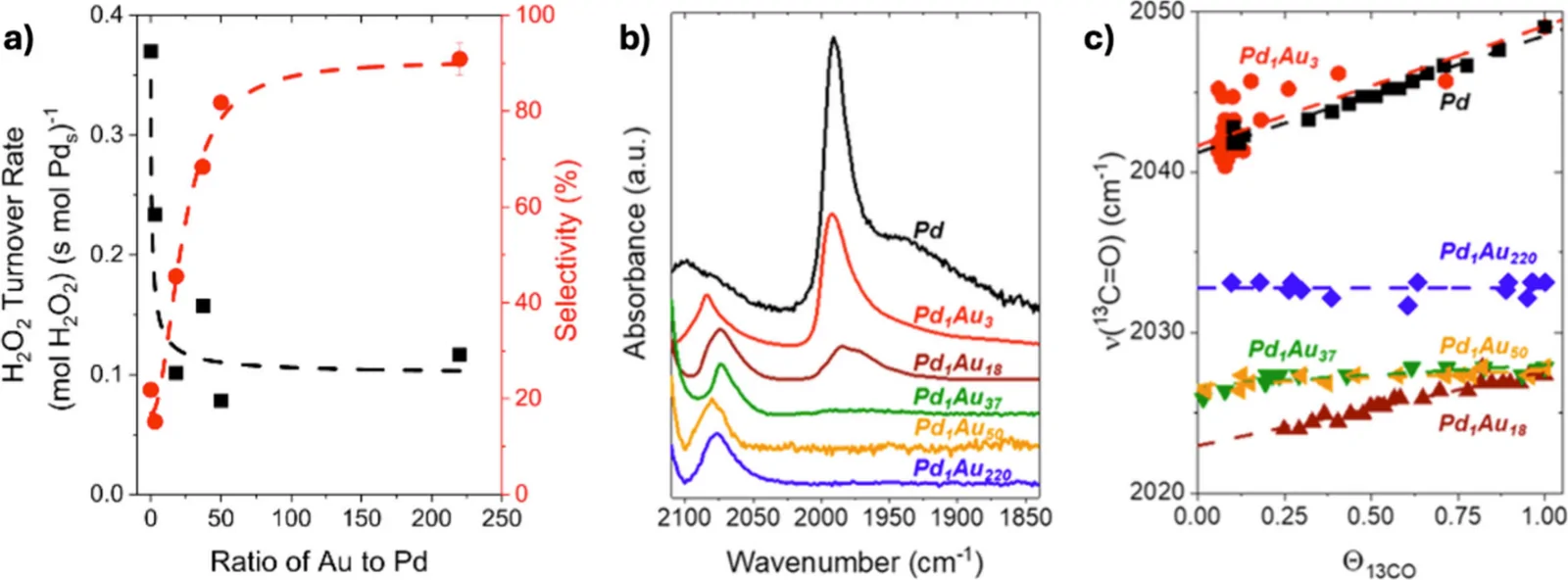
a) Steady-state H_2_O_2_ turnover rates (black) and primary H_2_O_2_ selectivities (red) for Pd and Pd_1_Au_*x*_ catalysts with a range of Au to Pd atomic ratios. b) Normalized infrared spectra of CO*-saturated surfaces of Pd and Pd_1_Au_*x*_ catalysts. c) Vibrational frequency for the ν­(C = O) mode of η^1^-^13^CO*_Pd_ as a function of the fractional coverage of ^13^CO* on monometallic Pd and PdAu_*x*_ at 303 K. Adapted with permission from ref.^302^ Copyright 2021 American Chemical Society.

To clarify the respective roles of Pd and Au sites and the sequence of elementary steps in H_2_O_2_ formation on PdAu SAAs, Svensson and Grönbeck combined DFT with KMC and proposed a solution-mediated site-communication mechanism.^314^ H_2_ dissociates on isolated Pd sites, after which H* oxidizes to H^+^ in solution and the excess e^–^ remains on the metal. Undercoordinated Au edge and corner sites then accept H^+^ and electrons to reduce O_2_ through OOH* to H_2_O_2_, relying on proton transfer in the liquid and electron conduction in the metal rather than surface spillover of H from Pd. Furthermore, by applying generalized CN scaling for Au terrace, edge, and corner sites and coupling it to KMC simulations, they quantified rates and selectivity across nanoparticle morphologies and Pd monomer distributions, predicting near 100% selectivity when Pd monomers reside at low density in (111) facets, whereas Pd near edges, in (100) facets or in excess lowered selectivity.

The role of the support in modifying H_2_O_2_ synthesis has also been examined, specifically in the context of generating reactive oxidants *in situ* for hydro-oxidation. Au nanoparticles supported on TiO_2_ are known to be active for O_2_-based selective oxidations and can generate H_2_O_2_ under H_2_/O_2_ feeds. H_2_O_2_ formation is thought to occur at the Au-metal oxide interfacial sites, and the overall rate is limited by the poor ability of Au to dissociate H_2_.^315^ Cargnello and co-workers synthesized PdAu nanoparticles containing 15 at% Pd to aid H_2_ activation and immobilized them on TiO_2_ or carbon.^316^ Under 2-propanol hydro-oxidation at 150 °C with cofed H_2_ and O_2_, all catalysts were more than 99% selective to acetone, and PdAu SAAs on TiO_2_ showed roughly twice the activity of Au on TiO_2_. Notably, PdAu on carbon remained active when the bed was physically mixed with TiO_2_, whereas Au on carbon was inactive, consistent with a bifunctional mechanism in which Pd sites dissociate H_2_ and Au activates O_2_ without O–O cleavage to generate a mobile oxidant consistent with H_2_O_2_ that diffuses to oxide grains where selective alcohol oxidation occurred. Given the high Pd concentration compared to most SAA reports, the role of Pd single atoms vs small clusters in this reaction may require further interrogation.

Related to this work, Huang and co-workers designed a dual site catalyst that coupled Pd single atoms on C_3_N_4_ and a PdSn SAA on SnO_2_ by tuning the Pd and Sn loadings.^317^ An optimum composition was identified at 3 wt % Pd with 8 wt % Sn, achieving 92% H_2_O_2_ selectivity with a H_2_O_2_ productivity of 7,020 mol kg_Pd_^–1^ h^–1^, outperforming the Pd/C_3_N_4_ SAC benchmark at 65% selectivity and 3,387 mol kg_Pd_^–1^ h^–1^, while Pd NPs on C_3_N_4_ delivered only 18% selectivity and 1,212 mol kg_Pd_^–1^ h^–1^. They proposed that Pd–N_4_ single atoms on C_3_N_4_ primarily activate H_2_, while Pd sites interacting with Sn at the SnO_2_ surface favored the OOH* route and suppressed O–O bond cleavage. *In-situ* DRIFTS supported this two-site mechanism showing that isolated Pd sites readily dissociated H_2_, and that an OOH* signal appeared on the dual-site catalyst under mixed feeds, consistent with a 2e^–^ pathway toward H_2_O_2_. However, direct evidence for which SAC or SAA sites predominantly mediate H_2_ dissociation requires further interrogation. Clear differentiation of the roles of different sites in catalysts containing SAAs in addition to other active sites is an important area of continued research as SAAs are tailored for more complicated chemistries.

#### 3.4.3 Other Oxidation Reactions

In addition to the representative oxidation reactions discussed above, SAA catalysts based on a diverse range of alloy combinations have been explored across a broad range of oxidation chemistries. Below, we briefly highlight several additional examples that illustrate the generality of the SAA approach and discuss CO preferential oxidation (PROX), CO oxidation, partial oxidation of methane (POM), and the liquid phase aerobic oxidation of 5-hydroxymethylfurfural (5-HMF).

CO-PROX is used to purify hydrogen fed to proton exchange membrane fuel cells (PEMFCs) to selectively remove CO, which acts as a catalytic poison when present in the H_2_-rich feed gas. Typically, an H_2_ rich stream (∼50% partial pressure) containing ∼ 1000 ppm of CO and stoichiometric oxygen is fed to the PROX catalyst with the aim of driving only CO oxidation. Zeng and co-workers reported that tuning the Pt to Cu weight ratio in PtCu SAAs supported on CeO_2_ enabled highly selective CO-PROX that proceeded mainly *via* a Mars van Krevelen (MvK) mechanism.^318^ In their composition series, Pt_0.1_Cu_0.19_/CeO_2_ delivered optimal CO conversion and CO_2_ selectivity close to 100% at 80 °C. They proposed that two distinct active sites participated in the mechanism. *Operando* DRIFTS suggested that the first active site, isolated Pt atoms on the Cu host, exhibited a weakened CO–Pt interaction compared to Pt/CeO_2_, and that during reaction Cu^0^ was oxidized to Cu^+^ to generate a Cu^+^–CeO_2_ interface. The second type of active site, this Cu^+^–CeO_2_ interface, was partially reduced, with concomitant formation of surface oxygen vacancies, which in turn activated gas phase O_2_ and resupplied lattice oxygen, thereby functioning as the MvK-type oxygen supply and activation site. The involvement of lattice oxygen was supported by CO/He and isotopic switching experiments, in which CO_2_ formation persisted even without gas-phase O_2_ and ^44^CO_2_ formation from ^28^CO/^36^O_2_ indicated direct participation of lattice ^16^O in the oxidation catalysis, supporting a MvK-type pathway. This work suggests that SAA sites may be able to work in tandem with other active sites and drive challenging selective oxidation reactions.

More recently, Shan and co-workers reported a first-principles microkinetic screening study across many transition metal SAAs that identified oxygen and hydroxyl migration between host and dopant sites as key descriptors for CO PROX performance. The study further suggested an optimal window of CO and O binding strength to the single atom site, and highlighted SAA candidates such as Rh_1_Cu, Rh_1_Ag, and Ni_1_Cu that should favor CO oxidation while suppressing H_2_ activation.^319^

CO oxidation to CO_2_ is one of the most intensively studied reactions in heterogeneous catalysis, both because it is important for automotive exhaust treatment and often used as a model reaction to interrogate catalyst structure and function. Before Rupprechter and co-workers reported on PdAu SAAs, research into doped Au clusters for CO oxidation had mostly centered on the multiple atoms substitution of Au with Cu or Ag.^320^ They examined CO oxidation on PdAu_24_/TiO_2_, an atomically precise Au-based cluster catalyst with a Pd:Au atomic ratio of 1:24 at a total metal loading of 2 wt %. Relative to pure Au_25_/TiO_2_ prepared and tested under the same conditions, PdAu_24_/TiO_2_ showed a much earlier activity onset near 225 °C and reached more than 90% CO conversion at 250 °C, whereas Au_25_/TiO_2_ gave only about 10% conversion at the same temperature. This enhancement was attributed to a pretreatment in 5% O_2_/Ar followed by 5% H_2_/Ar at 250 °C, which induced migration of the single Pd dopant to the Au surface and generated isolated Pd surface sites instead of extended Pd ensembles. These surface-exposed Pd sites were proposed to promote O_2_ activation. Operando DRIFTS further indicated Pd–Au rearrangement under CO oxidation and, for multiply doped clusters, features attributable to neighboring Pd sites or Pd–Au bridge sites, whereas EXAFS detected no significant Pd–Pd coordination. This contrast illustrates the difficulty of excluding minority dopant ensembles using ensemble-averaged XAS alone and highlights the need for complementary operando characterization.

Other prior reports identified Bi as an effective promoter of CO oxidation on Pd that mitigated deactivation by overoxidation of the catalyst surface.^321,322^ Li and co-workers synthesized PdBi SAAs on SiO_2_ by varying Bi from 0 to 5 wt % at a fixed Pd loading of 1 wt %.^323^ The best performing sample was 1Pd2Bi-SiO_2_ with 0.8 wt % Pd and 2.6 wt % Bi, and it reached 100% CO conversion at 130 °C, whereas 1Pd-SiO_2_ required 180 °C. At 100 °C, the Pd normalized rate on 1Pd2 Bi-SiO_2_ was reported as 413 μmol_CO2_/g_Pd_·s, roughly 10× higher than conventional Pd-SiO_2_. XAFS indicated that calcination yields Pd_*x*_Bi_*y*_O_*z*_ oxide clusters, whereas H_2_ reduction reconstructed these species into a PdBi_1_ SAA as evidenced by the loss of Bi-Bi coordination and the emergence of Pd–Bi or Pd–O–Bi scattering. The authors propose that Pd seggergates into metallic domains that contain Bi single atoms (evidenced by TEM) and that Bi also exists seperately as oxidized Bi domains. They attributed the improved activity to Pd–O–Bi interactions that suppressed PdO sintering together with electron transfer from Bi to Pd that weakened CO binding. Although CO oxidation is classified as a total oxidation reaction, the fundamental principles of electronic effects and active site modulation demonstrated by the Bi promoter provide valuable insights for the rational design of SAA-based selective oxidation catalysts.

Partial methane oxidation (POM) involves reactions between CH_4_ and O_2_ to produce syngas (CO and H_2_), and two main mechanisms have been proposed: a direct partial oxidation pathway and a combustion reforming reaction (CRR) pathway in which H_2_O generated from combustion reforms CH_4_.^324^ Gong and co-workers synthesized a MoNi SAA with a Mo to Ni molar ratio of 1 to 20 and observed that POM proceeded with over 95% CH_4_ conversion and 97% syngas selectivity at 800 °C, as shown in Figure 34a.^325^ They further identified MoNi as the true active phase, in which Mo preferentially adsorbed oxygen and thereby alleviated O_2_ poisoning of Ni. A temperature programmed reaction with CH_4_:O_2_ = 2:1 coflow shown in Figure 34b revealed that CO_2_ and H_2_O were formed at lower temperatures, whereas CO and H_2_ appeared only when the CO_2_/H_2_O signals began to decrease, indicating an initial total combustion step followed by reforming between the remaining CH_4_ and the formed CO_2_/H_2_O. Complementary TPSR experiments under dry reforming of methane (DRM), steam reforming of methane (SRM), and combined DRM+SRM conditions shown in Figure 34c demonstrated that DRM favors RWGS with high CO selectivity, while SRM favors WGS with high H_2_ selectivity, and their coexistence yields ∼ 98% selectivity to both CO and H_2_. The results support the hypothesis that POM underwent the CRR mechanism in which DRM/SRM are coupled with WGS/RWGS equilibria after the initial combustion step. Based on DFT calculations, the authors argue that the isolated Mo site exhibits the optimal O* binding energy to promote selectivity in the POM reaction.

**Figure 34:**
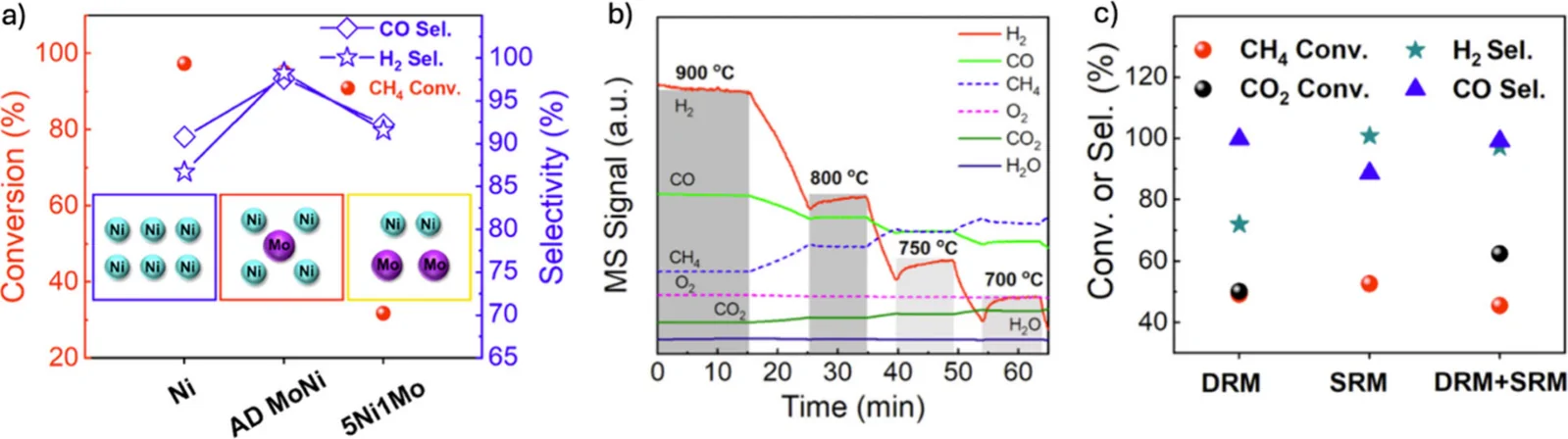
a) Catalytic performance of Ni/SBA-15, 5Ni1Mo/SBA-15 and atomically dispersed (AD) MoNi alloy/SBA-15 catalyst. b) TPSR experiment for AD MoNi alloy/SBA-15 under testing conditions with CH_4_:O_2_ = 2:1 flow for identification of POM mechanism. c) DRM and SRM control experiments. DRM represents dry reforming of methane and SRM represents steam reforming of methane. Adapted from ref.^325^ Copyright 2024 The Authors. Licensed under CC BY 4.0.

Hou and co-workers examined liquid phase aerobic oxidation of 5-HMF to 2,5-furandicarboxylic acid (FDCA) on Pt nanoparticles on C, single Pt atoms on N-doped C and the PtCo SAA with 0.06%Pt loading. The PtCo SAA exhibited a 99.6% FDCA yield, far superior to the Pt single atom N-doped C catalyst (29% yield), and exceeding commercial 5 wt % Pt/C (97%).^326^ They attributed the improvement to electron-donating Co that replaces N in the coordination shell around isolated Pt, forming Pt–Co interfacial motifs and making Pt more electron rich.^327^ O_2_ was proposed to be preferentially adsorbed and dissociated on Co sites adjacent to Pt to generate reactive O species that promote oxidation. Kinetic and theoretical analyses further suggested that Co shifts the kinetic bottleneck from O–O bond scission to C–H dehydrogenation, consistent with faster oxygen activation near the Pt–Co interfacial sites and more efficient transformation to FDCA. They compared only single compositions for Pt_1_@C–N and Pt_1_–Co@C–N and did not report a systematic Pt to Co composition series, so specific evidence supporting the necessity of an SAA structure was not provided.

#### 3.4.4 Outlook for Selective Oxidation Reactions on SAAs

Collectively, the studies show that SAAs provide a versatile platform for controlling selective oxidation catalysts by tuning the rate of oxygen dissociation and the reactivity, coverage and speciation of oxygen derived species on the surface of the catalyst.^63,328,329^ Despite such progress, work is needed to fully understand how different SAAs influence O_2_ activation, the reactivity of adsorbed oxidants, and the coverage of oxidants under the diverse conditions required for different selective oxidation reactions. Initial efforts indicate that this influence is condition-dependent, suggesting that catalytic behavior may vary substantially across different reaction environments. Nevertheless, recent studies of ethylene epoxidation and H_2_O_2_ synthesis clearly demonstrate how dopant atoms promote O_2_ activation and control the stability of specific oxygen species, providing promising insights that motivate future studies in this area.

### 3.5 Coupling Reactions

Coupling reactions represent a versatile class of transformations that enable the formation of new bonds such as C–O, C–C, and C–N from small molecule fragments. These reactions have been extensively developed using homogeneous catalysts, where the ligand structures on active metal species enable selective coupling processes.^330^ There have been substantial recent efforts to develop heterogeneous catalysts for coupling chemistry and SAAs have attracted attention due to their well-defined active sites and their intrinsic structural decoupling between activation and reaction sites.^331–333^ In this section, we discuss recently reported coupling reactions that exploit the structural advantages of SAAs to dictate pathways and examine how adsorbate coverage and structural evolution of the catalyst influence reactivity.

In oxidative coupling reactions of alcohols, reactive dopant sites in SAAs are proposed to influence the identity, concentration and reactivity of surface oxygen species, thereby strongly influencing reaction outcome. For example, as discussed in our previous review, Flytzani-Stephanopoulos and co-workers demonstrated that NiAu SAAs produced methyl methacrylate when methanol and methacrolein were cofed with O_2_, and Au–O_*x*_ species were identified as the primary active sites in the reaction, while Ni was proposed to promote the rate of formation of Au–O_*x*_.^334^ More recently, Flytzani-Stephanopoulos and co-workers examined the influence of PdCu SAA formation on the oxidative conversion of methanol to methyl formate (2 CH_3_OH + O_2_ → HCOOCH_3_ + 2H_2_O). Compared to pure Pd or Cu catalysts, the PdCu SAA provided distinct high selectivity and reactivity, demonstrating the promise of SAA for oxidative coupling reactions.^335^

For the same oxidative coupling reaction of methanol to methyl formate, Madix and co-workers showed that the active site structure depends on the identity of the host metal, which in turn influences reactivity.^336^ They prepared dilute Pd in Au nanoparticles embedded in raspberry-colloid-templated (RCT)-SiO_2_ and varied the Pd content from 1.7 at% to 3.4 at%. Increasing Pd from 1.7 to 3.4 at% caused an increase in methanol conversion from below 10% to about 55% while maintaining 95% selectivity to methyl formate, whereas pure Au exhibited below 10% conversion. Kinetic measurements showed a strong dependence of the rate on O_2_, consistent with O_2_ activation sites that were not saturated by adsorbed O or O_2_, and CO poisoning suppressed activity, indicating that Pd containing sites controlled turnover rates. Combining these kinetic measurements with prior DFT^337,338^ and surface studies showed that isolated Pd atoms in Au do not dissociate O_2_, they proposed that instead small Pd ensembles are responsible for O_2_ dissociation and methanol activation in PdAu dilute alloys. To further substantiate this proposal, the degree of Pd dopant isolation in the 1.7 and 3.4% PdAu samples prepared by tuning the Pd atomic ratio could be clarified by spectroscopic methods such as DRIFTS or XAS.

Eagan and co-workers studied the oxidative coupling of ethanol to ethyl acetate and proposed that Pd plays a distinct promotional role by generating Pd–Au interfacial sites that stabilized EtO* (ethoxy) and served as dominant coupling sites.^339^ Using kinetic measurements and DFT calculations, they argued that coupling was intrinsically fast and that the overall rate was governed mainly by how often AcAl* (adsorbed acetaldehyde) encountered EtO* on the surface, rather than by O_2_ or ethanol concentrations. By tuning the Pd to Au atomic ratio across a Pd_1_Au_*x*_ series, they found the highest promotion at Pd_1_Au_5_, but because Pd_1_Au_5_ showed ensemble character and Pd_1_Au_20_ provided a similar enhancement, composition-dependent evidence for a purely isolated Pd site requirement remains limited.

In our previous review paper, we discussed Wurtz C–C coupling of alkyl halides, specifically the formation of ethane from methyl iodide over PdAu SAAs.^340^ More recently, Sykes and co-workers demonstrated that the reaction pathway for C–C bond formation on NiAu SAAs depends on the Ni loading and that exchange of blocking iodine and methyl species at NiAu/PdAu SAA sites enables C–C coupling.^72^ On a 2% of a monolayer NiAu SAA, methyl species formed from CH_3_I predominantly undergo C–C coupling to ethane, whereas increasing the Ni loading to 50% of a monolayer led to methyl decomposition on the extended Ni sites suppressing ethane formation, as confirmed by TPD. DFT calculations combined with KMC simulations showed that at a low temperature the dopant sites were occupied by iodine (I*), while methyl (Me) species resided on Au, and that coupling proceeds *via* an adsorbate exchange step, I* + Me → I + Me*, followed by reaction between Me* on Ni and Me on Au (Figure 35). When this step was included in the KMC model, along with the subsequent C–C bond formation steps, the overall activation energies for C–C bond formation were 0.59 eV on PdAu and 0.57 eV on NiAu, and the resulting KMC-simulated TPD traces reproduced broad ethane desorption peaks around 235 and 260 K, in good agreement with experiment. This work shows the importance of understanding adsorbate coverages on SAAs during coupling reactions to identify likely experimental reaction pathways.

**Figure 35:**
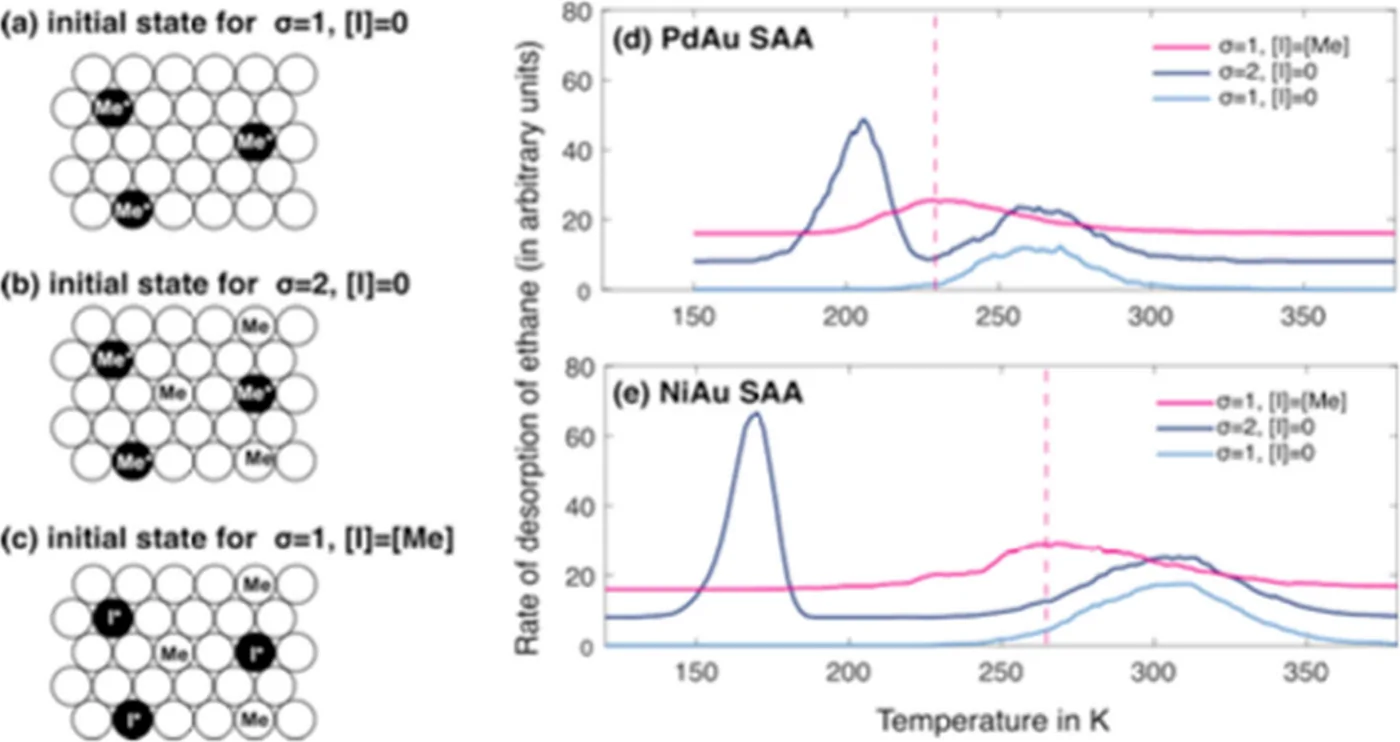
KMC-simulated TPD traces. a-c) Different initial conditions were simulated. d,e) The traces correspond to the production of ethane on the d) PdAu SAA and e) NiAu SAA. The dashed line indicates the position of the temperature peak when iodine is explicitly taken into account in the KMC simulations. Adapted with permission from ref.^72^ Copyright 2021 AIP Publishing.

The surface compositions of dilute PdAu alloys are responsive to exposure to oxidation conditions which provides important context for oxidation catalysis. Reece and co-workers showed that O_2_ pretreatment of Pd_0.08_Au_0.92_/RCT-SiO_2_ drives Pd enrichment at the nanoparticle surface, increasing the concentration of Pd sites that can interact with O_2_ by at least 3-fold compared to reductive pretreatments, as quantified by CO pulse titration.^341^ This restructuring can enhance the ability of PdAu catalysts to activate O_2_. However, the consequences of increased oxygen activation are not universally beneficial and depend also on the reacting alcohol. Friend and co-workers used gas phase oxidation of C_1_ to C_4_ linear alcohols over Pd_3_Au_97_ nanoparticles supported on RCT-SiO_2_ and showed that different reaction environments can reversibly block and regenerate active sites.^342^ Using Pd_3_Au_97_, the composition reported as optimal in their prior study,^336^ they found that at 423 K, methanol conversion increased from about 11% on Au RCT-SiO_2_ to about 32% on Pd_3_Au_97_ RCT-SiO_2_, consistent with Pd promoting O_2_ activation, while selectivity shifted from 95% methyl formate on Au to 82% methyl formate on Pd_3_Au_97_. For C_2_ to C_4_ alcohols, Pd provided little advantage, demonstrating that the promotional effect of Pd-mediated O_2_ activation is dependent on alcohol chain length, which they attributed to strongly bound carboxylates formed during over oxidation that block Pd sites. Adding 1% water during 1-propanol oxidation enhanced rates only on the RCT-supported Au and PdAu catalysts, with negligible response on bare RCT-SiO_2_ and, in their previous research, unsupported AgAu nanoparticles,^343^ supporting an OH reservoir role for the RCT support at the Au support interface. Together, these results connect oxygen activation, hydroxyl-mediated oxygen supply, and carboxylate poisoning to the observed activity–selectivity trends in dilute Au-based alloy catalysts. Because composition was fixed at Pd_3_Au_97_ in this study, direct composition dependent evidence for SAA necessity is not provided here, and the main contribution is the reversible site blocking and regeneration.

These studies show that coupling reactions on SAAs have so far been explored only in a narrow set of model systems, yet these studies highlight several unique opportunities for controlling bond formation. In particular, they underscore the importance of the rational codesign of dopant–host combinations and metal–support interfaces for controlling site blocking and regeneration, particularly under oxidative reaction environments. While the mechanistic understanding of SAA-mediated coupling has advanced considerably on the theoretical side,^85,344,345^ experimental work in this area remains comparatively limited. Addressing this gap should enable far broader control over activation barriers and selectivity in C–C and C–O coupling on SAAs than has yet been realized.

### 3.6 Other Reactions

Beyond the reactions discussed in the preceding sections, SAAs have also been applied to a broad range of additional thermal catalytic transformations. In this section, we highlight reaction classes that were not covered earlier and those that have recently gained momentum, including the water–gas shift (WGS) reaction, Fischer–Tropsch synthesis (FTS), reductive amination, isomerization, DRM, and methane cracking.

#### 3.6.1 Water–Gas Shift Reaction

The WGS converts CO and water into CO_2_ and hydrogen and serves as a key step for removing CO from syngas while simultaneously increasing the H_2_ concentration. In industrial processes, high and low temperature WGS stages are typically combined to achieve both high CO conversion and process stability. Consequently, catalysts with high activity and thermal durability have been the subject of considerable investigation.^346^ Rupprechter and co-workers designed CuPdAu trimetallic SAA catalysts on ZnO using PdAu_24_ as a fixed cluster scaffold and then substituting Cu stepwise, yielding an immobilized trimetallic SAA with an average surface composition close to Cu_3_Pd_1_Au_21_ in which Pd is retained as an isolated single atom dopant.^347^ In the WGS reaction this CuPdAu/ZnO catalyst reached about 67%/wt% loading-normalized CO conversion at 400 °C, whereas the industrial Cu/ZnO/Al_2_O_3_ benchmark reached 13%/wt% conversion under the same conditions, as seen in Figure 36. The authors proposed that pretreatment generated a strong metal–support interaction (SMSI)-derived ZnO cap that partially covered the sub nanometer SAA clusters and suppressed their sintering at high temperature. Under WGS conditions, CO partially reduced and lifted this overlayer, re-exposing the metal sites and dynamically restoring activity. Within this redox cycle, Pd was assigned as the site for CO adsorption and activation in the reduction half cycle, whereas Cu served as the redox center that activated H_2_O and supplied oxygen in the reoxidation half cycle. However, the relatively small magnitude of the promotional effect of Pd suggests that it may not serve as a unique active site. Further, the ZnO cluster interface acted as a dynamic SMSI cap that protected against sintering yet was partially removed under reaction conditions to re-expose active sites. This proposed reaction mechanism re-emphasizes a continuing theme in this review – supports can be an important additional lever in tuning the reactivity of SAA catalysts.

**Figure 36:**
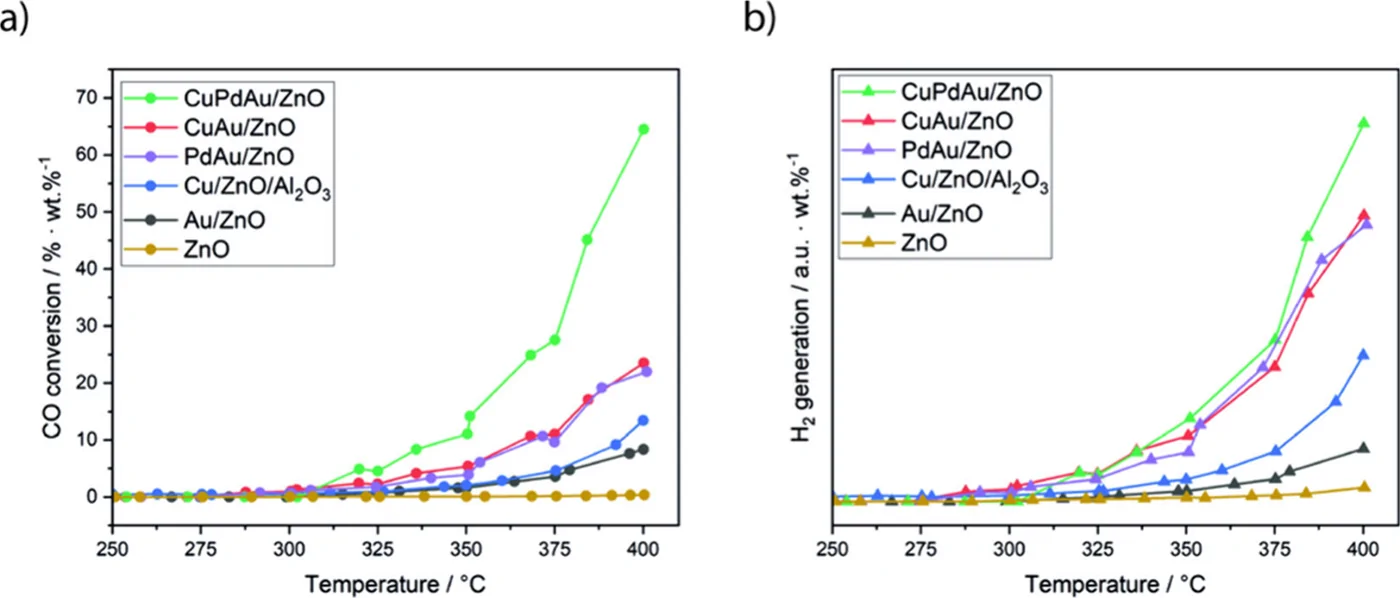
CO conversion and hydrogen production during WGS reaction normalized to the catalyst loading. a) CO conversion during the first WGS reaction cycle, b) corresponding H_2_ generation for the first cycle. Adapted from ref.^347^ Copyright 2025 The Authors. Licensed under CC BY 4.0.

#### 3.6.2 Fischer–Tropsch Synthesis

FTS is a surface-catalyzed reaction that converts CO + H_2_ (syngas) into long-chain paraffins, and Co and Ru catalysts have attracted considerable attention because they offer higher productivity, longer durability, and lower operating temperatures as compared to Fe.^348^ Li and co-workers designed RuCo SAAs that achieved higher activity and long-chain hydrocarbon selectivity than less dilute Ru/Co NPs and Co NPs. At 150 °C, Ru_1_Co_n_ (no stoichiometric composition of the SAA was provided because Co was derived from the Co–Al–O support) reached 12.5% CO conversion with 86.0% C_5_^+^ selectivity, whereas Ru/Co NPs and Co NPs delivered 5.6% conversion with 81.3% C_5_^+^ selectivity and 3.1% conversion with 77.7% C_5_^+^ selectivity, respectively.^349^ They prepared the RuCo SAA by introducing Ru species into a CoAl-LDH precursor and subjecting it to H_2_ reduction at 300–400 °C. They subsequently ran the FTS reaction while stepwise tracking the formation, operation, and regeneration of the single atom active sites by *in situ* XAFS. As shown in Figure 37, *in situ* XAFS evidenced that Ru species initially residing in a Ru–O_4_–Co_2_ oxide environment were converted upon H_2_ reduction into Ru–Co_3_ (this stoichiometric ratio was suggested only locally based on EXAFS fitting) SAA sites, and that under syngas these sites retained Ru–Co coordination while developing Ru–C and Ru–C–C­(O) contributions. DFT calculations further revealed that, at the Ru–Co_3_ active sites of the RuCo SAAs, the energy barriers for C–O bond cleavage and for C–C bond formation *via* CO insertion were greatly reduced, rendering chain-growth pathways to long-chain hydrocarbons both thermodynamically and kinetically more favorable than C_1_-terminating routes to methane and CO_2_.

**Figure 37:**
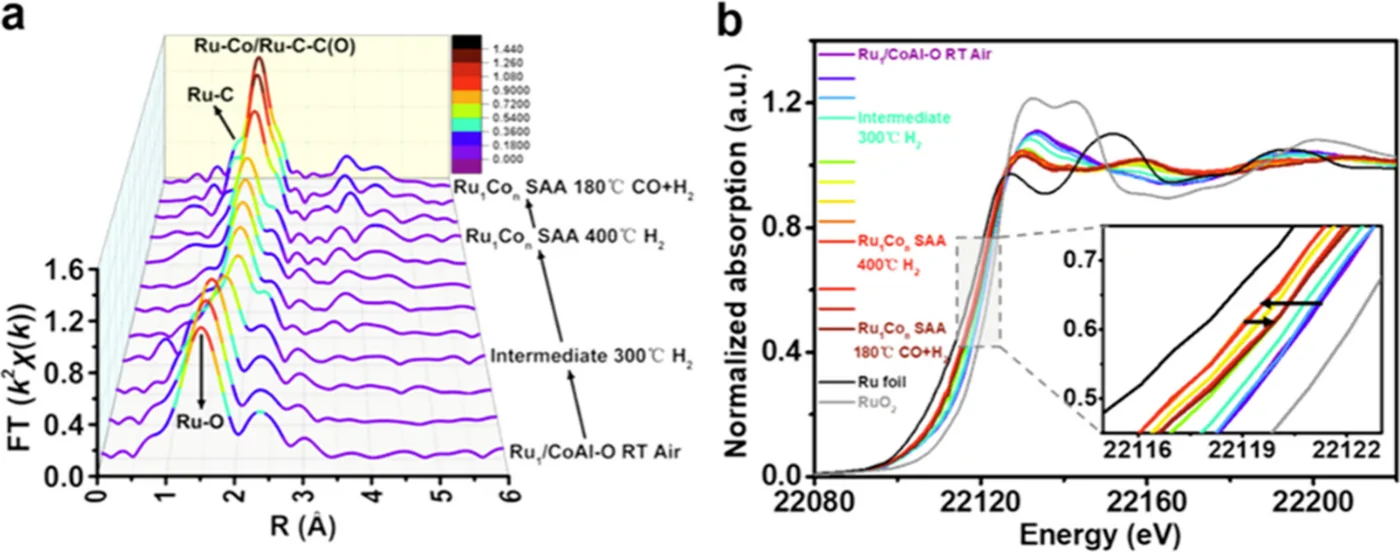
*In-situ* XAFS measurements collected under different conditions on Ru_1_Co_n_ SAA. a) Ru K-edge FT EXAFS spectra and b) Ru K-edge XANES. Adapted with permission from ref.^349^ Copyright 2021 American Chemical Society.

Porosoff and co-workers applied the same strategy, synthesizing RuCo SAAs by dilute Ru doping of a CoAl-LDH precursor, and demonstrated reactive separation of a 50/50 CO/CO_2_ feed, in which CO was selectively converted to C_5_^+^ hydrocarbons while CO_2_ conversion remained below 1%.^66^ They showed that Ru acted not as a FTS active site, but as a promoter that enhanced the reducibility and phase stability of Co, and that on the resulting Co and RuCo surfaces CO chemisorbed strongly whereas CO_2_ was weakly adsorbed, leading to the selective activation of CO. Quantitative analysis of temperature and heat-transfer effects further revealed that between 200 and 210 °C the RuCo SAA structure and the hcp Co phase were preserved and high C_5_^+^ selectivity was maintained, whereas between 220–250 °C and above, the exothermic methanation reaction generated hot spots in the catalyst, induced structural changes, and caused a sharp increase in CH_4_ selectivity. This highlighted that in addition to Ru doping, maintaining low operating temperatures is critical for preserving C_5_^+^ selectivity in CO/CO_2_ mixed feeds. Both these studies combine detailed experiments with theory to show that isolated Ru sites promote and stabilize reduced Co under CO rich conditions, leading to improved activity and C_5_^+^ selectivity in the FTS reaction. However, neither paper provides a systematic Ru to Co composition series down to the dilute limit, so composition-based evidence for the role of the SAA structure vs small Ru ensembles could be further clarified.

#### 3.6.3 Dry Reforming of Methane

DRM is a reaction that converts CO_2_ and CH_4_ gas streams from sources such as ammonia plants and biogas directly into syngas without prior separation of CO_2_ and CH_4_, and this syngas can then be further upgraded into high value-added chemicals. However, because this reaction is highly endothermic and therefore costly to operate, there is strong interest in developing catalysts that exhibit high enough activity to approach equilibrium conversion with minimal catalyst loading as well as stability even under relatively low temperature operating conditions.^350–352^ Sasmaz and co-workers employed a yolk–shell structured SAA catalyst with a Pt–NiCe core confined within hollow SiO_2_ that catalyzed DRM at a relatively low temperature of 500 °C and showed almost no loss of activity or coke deposition after over 120 h on stream.^353^ By varying the Pt loading from 0.1 to 1 wt % and comparing structures (yolk–shell vs wet impregnation), H_2_-TPR showed that introducing Pt shifted the NiO reduction β and γ peaks to lower temperatures, consistent with Pt acting as a promoter that facilitated Ni reduction and activation *via* H_2_ spillover. Analysis of the δ peak associated with NiO species strongly interacting with SiO_2_ indicated that the yolk–shell architecture induced SMSI that effectively suppressed Ni sintering in Figure 38a. TGA analysis in Figure 38b quantified coke and showed a clear optimum at Pt^0.25^–NiCe@SiO_2_. The carbon deposition decreased from 0.41 mg/g_cat_ on NiCe@SiO_2_ to 0.2 mg/g_cat_ on Pt^0.1^-NiCe@SiO_2_ and dropped to 0.03 mg/g_cat_ on Pt^0.25^-NiCe@SiO_2_, whereas a wet impregnated Pt^0.25^-NiCe@SiO_2_ showed 0.62 mg/g_cat_. These results supported the conclusion that atomically dispersed Pt, which bound CO more weakly, suppressed carbon deposition and, together with the strong SMSI of the yolk–shell catalyst, mitigated both coking and metal sintering, which explains the excellent stability.

**Figure 38:**
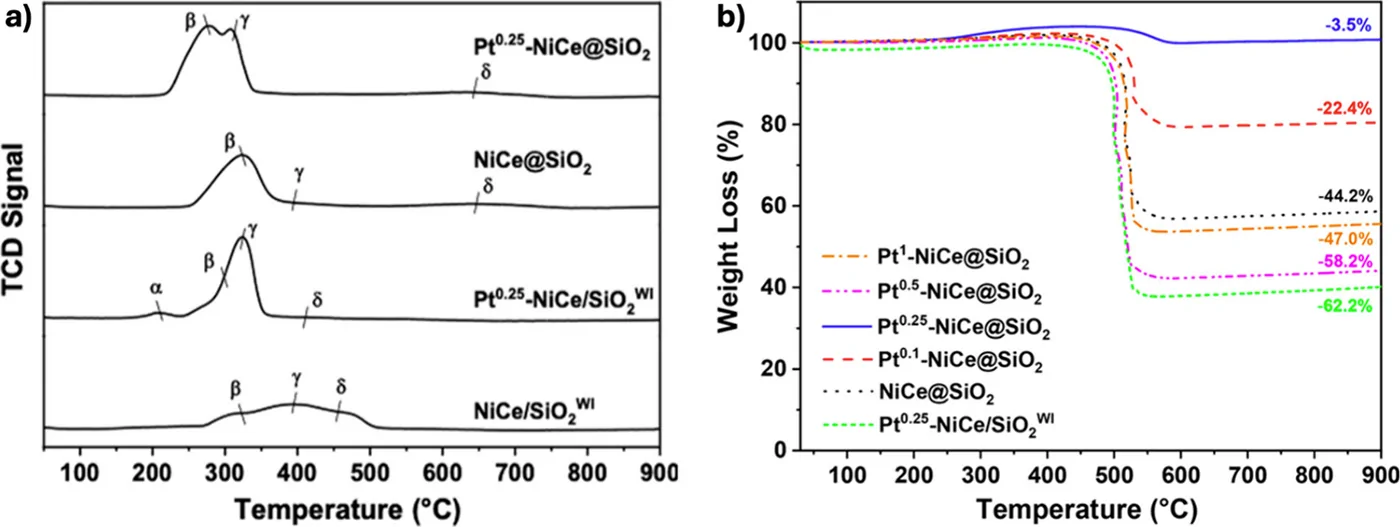
a) H_2_-TPR profiles for the yolk–shell and impregnated catalysts with and without 0.25 wt % Pt promotion. b) TGA profiles of Pt^x^-NiCe@SiO_2_. Adapted with permission from ref.^353^ Copyright 2021 American Chemical Society.

Building on the preceding experimental work, Peng and co-workers analyzed the DRM reaction on PtNi(111) surfaces with different Pt contents using DFT, thereby providing a theoretical design guideline for the optimal Pt loading range.^354^ They showed that Pt doping shifted the Ni d-band center downward, which not only weakened the overall binding of CO and various C-containing adsorbates, but also increased the activation barriers along the CH_4_ decomposition pathway, so that the rate of carbon formation became slower than that of carbon removal pathways such as CH/C oxidation and thus suppressed carbon deposition. In addition, as the surface Pt fraction increased to about 22–33 at%, CO_2_ activation shifted from the direct C–O cleavage pathway and instead proceeded *via* H-assisted CO_2_ hydrogenation and CH+OH coupling. Under these conditions, the effective activation energy for CH oxidation became lower than that for CH_4_ decomposition, which led to the discovery of Pt–Ni SAA compositions that are intrinsically resistant to coke accumulation.

In contrast, Passos and co-workers tuned the Ni to Cu molar ratio in NiCu SAAs supported on CuMgAl hydrotalcite-derived oxide and evaluated DRM at 800 °C, and revealed the limitations of the NiCu SAA concept.^355^ At the most dilute Ni levels where Ni was atomically dispersed (Ni_0.001_Cu or Ni_0.002_Cu), the initial CH_4_ conversion stayed at roughly 7%, close to the CuMgAl baseline of 11%, with TOF values of about 60 s^–1^. In contrast, at a Ni to Cu ratio around 0.1, where small Ni ensembles were inferred, the initial CH_4_ conversion increased to 39% with a TOF of 143 s^–1^, and coke deposition decreased to 0.73 mg_carbon_/mg_cat_ compared with 2.06 mg_carbon_/mg_cat_ on CuMgAl. These results indicated that isolated single Ni atoms in Cu are insufficient to ensure adequate activity and stability in DRM and that, instead, Ni ensembles above a certain size are required, highlighting that precise control over the basicity of the support and the Ni cluster size (3–4 Ni atoms) will be key design parameters for future Ni-based alloy catalysts. They ascribed the higher activity at Ni_0.1_Cu to small Ni ensembles but noted that their data could not specifically confirm 3–4 atom Ni clusters and that further structural verification is needed.

#### 3.6.4 Methane Cracking

Direct methane cracking involves splitting CH_4_ into H_2_ and solid carbon. This reaction has attracted considerable attention because it can be an energy efficient route for generating H_2_ with no stoichiometric CO_2_ production (in contrast to steam methane reforming), although separation of the C product is a challenge both for the process and catalyst lifetime.^356^ Deng and co-workers used a machine learning based screening of over 10,950 SAA surfaces to identify Re doped Ni as a top candidate for rapid C–H bond dissociation at 450 °C, then designed ReNi SAAs for methane cracking and evaluated them in a ball milling reactor that provided continuous mechanical vibration.^62^ Under 450 °C and 1 atm in a ball milling reactor operating at 500 rpm, ReNi SAAs delivered about 7.8% CH_4_ conversion together with nearly 100% H_2_ selectivity and remained stable for 240 h. Ball milling experiments combined with DFT and AIMD simulations confirmed that vibration-induced lattice deformation shifts the d-band center upward, lowering the C–H dissociation barrier by 0.15 eV. Concurrently, mechanical friction and collisions facilitate the physical detachment of carbon fragments, effectively mitigating catalyst deactivation.

### 3.7 Conclusion and Outlook in Thermal Catalysis

Across the thermal reactions surveyed in these sections, our review highlights that the conventional SAA design, in which a reactive dopant is diluted into a coinage-metal host, remains a robust and widely applicable strategy for selective hydrogenation, dehydrogenation, oxidation, coupling, and many other reactions. At the same time, the recent literature demonstrates a new direction toward expanding the paradigm, such as combining two reactive metals or exploiting SMSI to generate new interfacial active sites that deliver enhanced catalytic performance. Despite this progress, many studies still rely primarily on DFT calculations and microkinetic modeling to elucidate reaction mechanisms, and experimental validation of the role of SAA sites in catalytic reactions remains limited due to difficulties in the experimental characterization of single atom sites and dynamic restructuring that occurs in reactive environments. Future work should place greater emphasis on linking theoretical work with experimental analyses, such as *operando* spectroscopy and transient kinetics, to move from reaction- and catalyst-specific conclusions toward more general design rules.

Despite the widespread use of SAAs in research, scalable synthesis for industrial translation remains an underexplored area. A promising study by Ji and co-workers demonstrated that mechanochemical ball milling can produce PtCo SAAs and further scale the preparation to the kilogram level while preserving atomic dispersion, electronic structure, and catalytic performance for 5-HMF hydrodeoxygenation. They also generalized the same synthetic protocol to a family of MCo (M = Pd, Ru, Ir, Rh) SAAs, highlighting ball milling as a practical route that can meet large-scale production requirements.^54^

Beyond scale-up, additional validation under process-relevant conditions is still required, including durability in industrially realistic gas compositions and sustained activity under high-temperature and high-pressure operation.^357^ Nevertheless, the selective activation, stability and mixed-feed reactivity enabled by SAAs suggest that their impact extends beyond reaction-by-reaction performance, as they can enable process-level integration^66^ across coupled reaction networks by reducing or simplifying separation steps. We refer the interested reader to detailed, forward-looking reports on the industrial adoption of SAAs written by scientists at Johnson Matthey.^51,358^ These reviews further discuss practical considerations including scalable synthesis routes, realistic precious-metal loadings, tolerance to feed impurities that put extra constraint on catalysts, and the preservation of isolated active-site structures under prolonged operation, all of which remain important areas of investigation for the commercial deployment of SAA catalysts.

Table 5 summarizes representative SAA systems reported in this section, together with the experimental and characterization techniques used to verify atomic isolation, the corresponding reaction type or elementary step, key catalytic performance metrics, the proposed reaction mechanism, and the relevant references.

**Table 5:** Summary of Representative SAA Catalytic Systems, The Techniques Used to Verify Atomic Isolation, The Associated Reaction Type or Elementary Step, Key Catalytic Performance Metrics, Proposed Reaction Mechanisms, and Corresponding References

**Reaction type**	**SAA system**	**Technique used to show evidence for atomic isolation**	**Key catalytic metric(s)**	**Main catalytic findings**	**Ref. #**
**Hydrogenation**	PdCu(111)	PD-RAIRS	Isolated Pd provides H_2_ activation and Cu(111) contributes alkene selectivity	Enhanced conversion and selectivity	^225^
	PdAg(111)	PD-RAIRS, CO-TPD	Isolated Pd sites increases conversion, and Ag sites weaken adsorbate binding	Enhanced conversion but promote undesired hydrogenation pathways	^140^,^237^
	PdAu	CO-DRIFTS, DFT	Isolated Pd provides H_2_ activation and Au sites weaken hydrocarbon binding	Identified H_2_ dissociation as the rate limiting step	^240^,^241^
	PdCu	EXAFS, TEM	Isolated Pd promotes H_2_ dissociation and H spillover to Cu. Cu provides selective hydrogenation	Enhanced conversion toward desired products	^244,249^
	PdAg	EXAFS	Isolated Pd provides H_2_ activation and Ag weakens H binding	Enhanced activity and selectivity.	^36^
	PtCu	EXAFS, DFT	Interfacial Pt remotely lowers the H_2_ dissociation barrier on Cu sites and weakens adsorbate binding	Interfacial Pt remotely modifies the electronic structure of Cu surface sites, improving selectivity. Dynamic behavior of Pt	^245,246^
	PtCu	TEM, DFT	H_2_ atmosphere induces Pt surface segregation and ordered Pt distributions on Cu facets/steps.	Dynamic Pt segregation under H_2_	^247^
	CrCu, MnCu	DFT	Midtransition-metal dopants tune C = O and C = C binding, favoring moderate C = O adsorption	Theoretical screening identified promising SAA catalysts that enhance C = O hydrogenation selectivity	^248^
	RuCo	AC-HAADF-STEM, XRD, XPS, XANES/EXAFS, CO-DRIFTS	Electron-rich Ru enhances C = O adsorption and H_2_ activation	Enhanced activity and stability. Kinetics showed Ru lowered the barrier of rate determining step	^251^
	RuNi	AC-HAADF-STEM, CO-DRIFTS, XANES/EXAFS	Ru–Ni interfacial sites control selective hydrogenation pathways by tuning substrate activation and intermediate hydrogenation	Enhanced activity, selectivity and stability	^252,257,258^
	IrNi	AC-STEM, CO-DRIFTS, XANES/EXAFS	Ir–Ni interfacial sites activate the N–O bond and suppress C = C hydrogenation	Theoretical screening. Enhanced selectivity	^253^
	PdNi	HAADF-STEM, XANES/EXAFS, CO-DRIFTS	Electron transfer from Ni to Pd forms electron-rich Pd, weakening ethylene adsorption and suppressing overhydrogenation	Enhanced activity, selectivity and stability with reduced apparent activation energy	^254^
	PtM (M= Co, Ni, Cu)	HAADF-STEM, XANES/EXAFS	Pt activates H_2_ while neighboring ensemble sites fix reactant	Enhanced activity and stability	^255^
	PtCo	AC-HAADF-STEM, XANES/EXAFS, XPS	Pt–Co interfacial sites facilitate C = O activation and lower the hydrogenation energy barrier	Enhanced activity and stability. Identified the slowest step	^256^
	PtPd	AC-HAADF-STEM, XANES/EXAFS, CO-DRIFTS, DFT	Electron deficient Pd enhances H_2_ activation and furan adsorption while resisting CO poisoning	Enhanced activity, selectivity and stability	^259^
**Dehydrogenation**	PtCu	STM, CO-DRIFTS, AC-HAADF-STEM, EXAFS depending on the study	Isolated Pt activates C–H bond, while Cu weakens product binding, suppresses deep dehydrogenation and coke formation	Enhanced catalytic performance	^38^,^68^,^69^,^272^,^276^,^277^
	PtFe	EXAFS, CO-DRIFTS, HAADF-STEM, XPS, PXRD	Pt single atoms provide efficient dehydrogenation sites, while the low-crystalline Fe overlayer gives decoking.	Enhanced catalytic performance	^277^
	RhCu	STM, CO-DRIFTS, EXAFS	Isolated Rh activates C–H bond, while Cu suppresses overdehydrogenation and coking	Enhanced catalytic performance. First-principles catalyst design	^56^
	NiCu	CO-IR, XANES/EXAFS, STM, DFT	Ni dopant lowers the dehydrogenation barrier and promotes α–C-H activation while Cu retains selectivity	Enhanced catalytic performance	^278^,^279^
	NiCu and RhCu	STM, CO-TPD, CO-RAIRS, CO-DRIFTS	Isolated Ni promotes selective dehydrogenation while isolated Rh can additionally cleave C–C bond	Both NiCu and RhCu increase conversion. Dopant identity controls selectivity	^91^
	PdCu	CO-infrared reflection absorption spectroscopy, STM, DFT	Isolated Pd promotes selective alcohol dehydrogenation, while Cu geometry stabilizes isolated Pd	100% selectivity and increase product yield	^226^
	NiAu	CO-RAIRS, CO-DRIFTS, TPD, DFT, XANES	NiAu lowers apparent activation energy and C–C scission barrier is increased	100% selectivity. Dynamic electronic structure and showed α-C–H and O–H bond cleavage is kinetically relevant	^58^
	PtCrAg	DFT, STM, TPD	Oxophilic Cr and hydrogenphilic Pt cooperate to break O–H bond. Cr stabilizes O-containing intermediates, while Pt stabilizes H.	Dual-site synergy in trimetallic PtCrAg: isolated Pt or Cr sites are inactive, but adjacent Pt–Cr dual sites enable ethanol dehydrogenation	^175^
	Ni, Pd, Pt, Rh doped Cu, Ag, Au	DFT	DFT-calculated formation energies, activation energies, reaction energies, BEP relationships for C–H activation, O–H activation, and C–O coupling	Computational kinetics and dopant identity controls whether C–H, O–H, or C–O steps are favored	^283^
	RuNi	HAADF-STEM, XANES/EXAFS, XPS, XRD	Ru sites anchored on MXene promote B–H/O–H bond cleavage, while MXene assists H adsorption/desorption and H_2_ formation	Enhanced catalytic performance. Kinetics indicated that H_2_O/O–H activation strongly affects the rate	^284^
**Methanol oxidation**	PtCu, RhCu	STM, CO-TPD	Pt doped Cu forms more defective oxides with shorter induction periods, while Rh doped Cu forms more ordered oxides with longer induction periods	Dilute Pt and Rh dopants tune Cu oxide order and defect density	^308^
**Ethylene epoxidation**	NiAg	STM, HERFD-XANES	Isolated Ni promotes O_2_ dissociation and stabilizes nucleophilic oxygen	Enhanced selectivity. Dynamic structure of Ni	^60^
**Direct synthesis of hydrogen peroxide**	PdAu	XANES/EXAFS, CO-FTIR	Isolated Pd in Au promotes H_2_ activation, while Au suppresses O–O bond cleavage	Enhanced selectivity	^302^,^314^
	PdSn	AC-HAADF-STEM, CO-DRIFTS, XANES/EXAFS	Pd single atoms on C_3_N_4_ and Pd–Sn sites on SnO_2_ promotes O_2_ hydrogenation while suppressing O–O bond cleavage	Enhanced productivity with selectivity	^317^
**Selective 2-propanol oxidation**	PdAu	TEM	Isolated Pd in Au promotes H_2_ activation, while Au activates O_2_ without O–O bond cleavage	PdAu replaced the Au/TiO_2_ interface for oxidant generation	^316^
**CO-PROX**	PtCu	AC-HAADF-STEM, CO-DRIFTS	Isolated Pt on Cux sites weaken CO adsorption, while Cu–CeO_2_ oxygen vacancies activate oxygen/lattice oxygen	Enhanced conversion and selectivity at low temperature	^318^
	RhCu, RhAg, NiCu	DFT	Isolated single atom sites activate CO, while host sites provide O/OH species and suppress H_2_ activation	High-throughput microkinetic screening of 672 SAAs	^319^
**CO-oxidation**	PdAu	DRIFTS, EXAFS, HAADF-STEM	Dynamic Pd migration creates isolated Pd surface sites, providing accessible CO binding sites	Enhanced catalytic activity. Dynamic structure of Pd	^320^
	BiPd	XANES/EXAFS, HAADF-STEM, CO-DRIFTS	Isolated Bi atoms tune CO/O_2_ and lower the barrier for intermediate formation	Achieved 100% conversion at low temperature and long-term stability	^323^
**Partial oxidation of methane**	MoNi	AC-HAADF-STEM, XANES/EXAFS	Oxophilic Mo sites tune O_2_/O adsorption and reduce O* poisoning on Ni, while preserving CH_4_ activation	Enhanced activity, selectivity and stability	^325^
**Liquid-phase aerobic oxidation of 5- Hydroxymethylfurfural**	PtCo	XANES/EXAFS, AC-HAADF-STEM, XRD, XPS	Pt–Co_3_ sites provide negatively charged Pt for key intermediate adsorption, while neighboring Co atoms promote O_2_ adsorption/activation	Enhanced product yield	^326^,^327^
**Oxidative coupling of unsaturated aldehydes and alcohols with methanol**	NiAu	CO-DRIFTS	Isolated Ni atoms in Au increase [Au–O_*x*_]^−^ active site formation and methanol activation while Au retains selectivity	Enhanced conversion with 100% selectivity	^334^
**Oxidative methanol coupling**	PdAu	STEM	Isolated Pd promotes O_2_ activation while Au maintains high selectivity	Enhanced conversion and selectivity	^336^
**Methyl iodide coupling**	NiAu, PdAu	STM, CO-RAIRS	Isolated Ni and Pd atoms on Au activate C–I cleavage and enable methyl coupling	Iodine acts as a site blocker, controlling the methyl-dopant exchange	^72^
**Oxidative methanol coupling and selective oxidation of linear alcohols**	PdAu	STEM	Isolated Pd sites promote O_2_ activation for oxidative methanol coupling, whereas longer-chain alcohols form strongly bound carboxylates that poison Pd sites	Alcohol-chain-length-dependent Pd promotion	^342^
**Water gas shift**	CuPdAu	MALDI-MS, HAADF-STEM	Isolated Pd atoms enhance CO adsorption/activation, whereas positively charged Cu facilitates the H_2_O/OH mediated reoxidation of reduced ZnO/oxygen vacancy sites	Enhanced catalytic performance. SMSI partial encapsulation	^347^
**Fischer–Tropsch**	RuCo	AC-HAADF-STEM, EXAFS, PXRD	Isolated Ru atoms with adjacent Co atoms promote both C–O bond cleavage and C–C chain growth	Enhanced catalytic performance and long-term stability.	^349^
	RuCo	AC-STEM, EXAFS, CO-DRIFTS	Ru dopants promote Co reduction via H spillover and help preserve the reduced Co active phase	High temperature Co restructuring. Active-phase stabilization	^66^
**Dry reforming of methane**	Pt-NiCe	AC-HAADF-STEM, EXAFS	Isolated Pt atoms enhance Ni reducibility via Pt–Ni interactions/H_2_ spillover and suppress carbon formation. Yolk-shell confinement promotes facile CO desorption	Enhanced catalytic stability and coke resistance	^353^
	PtNi	DFT	Ni to Pt charge transfer weakens CH_*x*_/C/O adsorption and increases the barrier for CH_4_-derived carbon formation	Mechanistic origin of coke resistance	^354^
**Methane cracking**	ReNi, IrNi	HAADF-STEM, XANES/EXAFS, time-of-flight secondary ion mass spectroscopy (TOF-SIMS)	Re and Ir single atoms lower C–H cleavage barrier, while ball-milling induced collision/friction removes deposited carbon from the catalyst surface	Enhanced H_2_ production and long-term stability. Coke removal	^62^

## 4 Electrocatalysis

### 4.1 Introduction

Research on SACs in electrocatalysis has been dominated largely by work on isolated atoms either anchored within graphitic materials or metal–organic frameworks (MOFs), or supported on conductive metal oxides.^33,74,359,360^ These systems are outside the scope of the present review. Work on SAAs has been more limited, although a growing number of examples have been published in recent years. We briefly highlight unique aspects of the electrochemical environment as it pertains to reaction mechanisms and material stability, then move to discuss the application of SAAs in different reactions. Few universal design strategies exist, so subsequent sections are organized by specific reactions and their closely related variants.

#### 4.1.1 Roles of Isolated Sites in Electrochemistry

Mechanism-informed design remains one of the primary goals and challenges in SAA electrocatalysis. As with thermal catalysis, common themes for electrochemical SAAs include the unique reactivity of the isolated atom itself, host-dopant interaction through either electronic modifications or neighbor-site cooperative pathways, and also spillover, where the guest atom may facilitate addition of a moiety that encounters coreactants upon diffusing to the host. Electrochemistry can further promote concerted ion-electron transfers from solution, such as proton-coupled electron transfer (PCET), which may in some cases diminish the need for spillover. The guest site may then be more predisposed to acting alone. SAAs can still break traditional adsorption-energy scaling relationships as was discussed in Section 2.4, though new interactions such as by adsorption of electrolyte ions must also be taken into account.

#### 4.1.2 Stability in the Electrochemical Environment

Electrochemical SAA systems also face notable challenges in stability due to the prevalence of corrosion phenomena.^361,362^ Electrochemical reactions are often operated near ambient temperature, which can in principle aid stability by suppressing solid diffusion and conventional particle ripening. On the other hand, extremely powerful driving forces are present for leaching by metal species redox chemistry as well as promoting phenomena such as galvanic ripening.^363^ This is exceptionally critical for preserving dilute sites, as there is not a large reservoir of material to replenish from. Susceptibility to corrosion can be understood in simple terms by considering standard redox tables: cathodic (reducing) potentials favor holding the metal in a zerovalent state, while anodic (oxidizing) potentials lead to formation of soluble cations or, at higher pH in water, metal-oxide species that may or may not be soluble.^361^ Cathodic corrosion phenomena are also possible but tend to occur at relatively more extreme potentials.^362^ Therefore, work on SAA electrocatalysts has been primarily shaped by reduction reactions. Oxidation reactions that are relatively facile and occur at small anodic potentials have also been studied.

While redox tables and more encompassing Pourbaix diagrams^364^ are readily available for elemental metals, these are only a starting point for understanding how SAAs may behave when polarized. The host metal may stabilize or destabilize a given species relative to its elemental form, and more complex relationships will exist in the presence of reactive substrates. The following reviewed literature all claim to have formed stable SAA structures, though *in situ* characterization has been deployed sparingly for electrochemical systems while *ex situ* techniques can mask changes that occur upon introduction or removal from reaction conditions. Stability can also be screened computationally, although the conventional DFT approach has its own limitations and poses difficulty when introducing solvent models and other complex reaction conditions. A handful of recent studies that have specifically focused on SAA surfaces restructuring in electrochemistry^365–367^ will be discussed in the context of specific reactions below.^366,368^

### 4.2 Reductive Electrocatalysis

Most reported studies on SAAs have focused on the oxygen reduction reaction (ORR), CO_2_ reduction reaction (CO_2_RR), and nitrogen or nitrate reduction reaction (N_2_RR/NO_3_RR). More complex reduction chemistries involving larger molecules, such as biomass derivatives, have been limited in scope but pose an area of opportunity for selectively modifying multifunctional molecules.^369^

#### 4.2.1 Oxygen Reduction Reaction

The ORR, shown below, is a central electrochemical transformation for sustainable energy conversion, serving as the cathodic reaction in a wide range of energy technologies. Full reduction to water is a four-electron pathway proceeding as follows:

$$\mathrm{Alkaline}:O_{2}+2H_{2}O+4e^{-}\to 4OH^{-}$$ 3

$$\mathrm{Acidic}:O_{2}+4H^{+}+4e^{-}⇋2H_{2}O$$ 4

The ORR is a key reaction for the operation of proton exchange membrane (PEM) fuel cells and alkaline fuel cells (AFC) running on hydrogen, formic acid, methanol, higher alcohol, or other fuels, as well as metal-air batteries.^370–372^ On most catalytic materials, the four-electron reduction to water is challenging due to the need to activate the O_2_ bond while maintaining moderate binding energies of various subsequent intermediates.^313,370,373^ Pt is well-established to be active for ORR, although the kinetics are still sluggish, and lower cost alternatives are desirable, with dispersion-optimized mass activities peaking for pure Pt around 0.25 A/mg_Pt_ at 0.9 V_RHE_.^374,375^ There is also interest in two-electron reduction to hydrogen peroxide, and/or from peroxide to water, for applications such as water treatment and hydrogen peroxide fuel cells.^376–378^ Advances using SAAs in these areas can help to mitigate the use of expensive transition metals such as Pt and Pd.^379^

Darby et al. explored SAAs of Pt-group metals hosted in Ag or Au, including a screening of tolerance to CO poisoning (often present in H_2_ gas feed for fuel cells) with DFT calculations. They suggested that these materials should be more selective to the four-electron ORR pathway than bulk Pt(111) in the presence of contamination due to weakened CO binding on the isolated site.^313^ Despite a lower density of active sites in a PtAu SAA, the CO-tolerance and efficiency toward the four-electron pathway were still estimated to compete with rates on bulk Pt(111). PtAu and PdAu were both predicted to have low theoretical overpotentials, highlighted in Figure 39. PdAu was found to be more tolerant than PtAu and was also found to be 10 orders of magnitude more tolerant to CO poisoning than Pt(111), suggesting that if CO is present Pd may be a more efficient active site.

**Figure 39:**
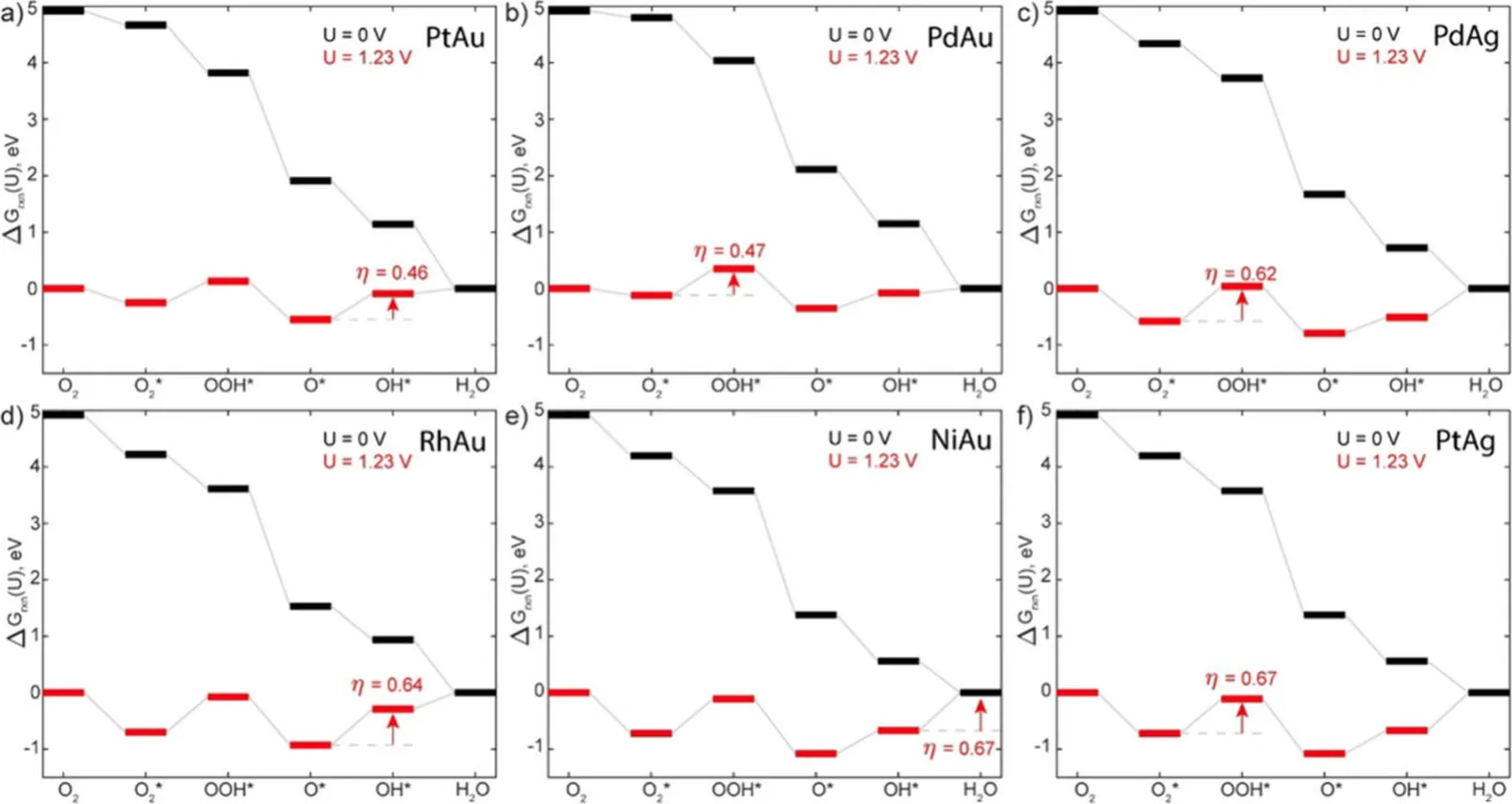
Gibbs free energy diagram for the four-electron ORR pathway on a) PtAu, b) PdAu, c) PdAg, d) RhAu, e) NiAu, and f) PtAg. Reproduced from ref.^313^ Copyright 2021 The Authors. Licensed under CC BY 4.0.

Cheng et al. combined experiment and theory to conclude that PtCo SAAs could achieve high selectivity toward the four-electron pathway.^380^ They attributed this to PtCo SAAs having delocalized charge, due to charge transfer between the Co surface and Pt atoms, that impacts the binding strength of intermediates. Atomic dispersion is commonly confirmed using synchrotron-based techniques such as XAS combined with high resolution microscopy. However, depending on the element of interest, there are laboratory methods, such as cyclic voltammetry and probe-molecule stripping experiments, that can indicate dispersion when combined with a method to determine loading such as inductively coupled plasma mass spectrometry (ICP-MS). In this work, the authors used a combination of HAADF-STEM and *in situ* XAS, which indicated that Pt atoms were atomically dispersed on the Co. When running CVs, Pt/C showed HER at reductive potentials where this reactivity was suppressed on the PtCo alloy, supporting isolated Pt sites. Moreover, pure Co supported on carbon nanotubes showed little ORR, but with the addition of Pt, clear ORR activity appeared. DFT calculations revealed that the OOH* intermediate binds optimally on PtCo SAAs through simultaneous binding on the Pt and Co sites. This binding motif was proposed to allow efficient O–OH cleavage and promote water formation. Two-electron selectivity to peroxide was measured as less than 5% in acid media by the rotating ring-disk electrode (RRDE) method, which assays peroxide formed at the working (disk) electrode using a second electrode (ring) over which the products flow. The mass activity was also reported to reach 2.57 A/mg_Pt_ at 0.85 V.

Utilizing single atom dopants to modify nearby active host metal sites, Luo et al. explored Au dopants in Pd and Pt, although only the former was evaluated for ORR activity.^381^ The catalysts were synthesized from PdBi and PtBi, where the Au dopants were added and then the Bi was removed using electrochemical dealloying. This removed Bi from the surface, but isolated Au atoms remained embedded within the tensile-strained Pd or Pt shells, as confirmed through HAADF-STEM, EELS, XPS, and synchrotron XAS measurements. The resulting AuPd SAA exhibited a mass activity of 5.37 A/mg_Pt+Au_ at 0.9 V RHE. Moreover, AuPd showed increased resistance toward deactivation by methanol. DFT calculations attributed this enhanced performance to electronic modification of the Pd surface by isolated Au atoms, changing the RDS from OH formation to H_2_O formation. The calculations assumed that the bridge site between Au and Pd SA is the active site. While new behavior was seen for the SAA, it was not addressed whether the changes in activity could be due to strain in the particle or vacancies caused by Bi dissolution.

Further exploring new types of active sites under realistic conditions for the four-electron ORR through the four-electron pathway, Pu et al. investigated Ag-based SAA clusters (M_1_Ag_54_) using DFT and MD simulations (with a DFT-parametrized potential), including calculated Pourbaix diagrams.^365^ Co, Ni, Fe, Ru, Rh, Pd, Pt, Au, Cu, Zn, and In were studied as dopant elements. The nature of the active center was identified as an M_1_O_*x*_(OH) complex formed under reaction conditions instead of the metallic dopant, emphasizing the importance of dynamic surface oxidation states during ORR. Among the compositions studied, Ag_54_CoO_2_OH emerged as the most active site, despite poor predicted activity for metallic Co SAAs. This was due to a need for striking a balance between O_2_ activation, OOH intermediate stabilization and OH desorption. In contrast, Pt was found to bind OH too strongly despite appearing optimal in its metallic state. These findings support experimental work by others, where Cu, Au and Pd alloys hosted in Ag appear to outperform Pt(111) in alkaline ORR.^329,365,382–385^ Given the importance of the active oxidation state, these materials should ultimately be examined by *in situ* characterization, as simpler *ex-situ* methods could lead to an incomplete or even incorrect understanding of the operating catalyst.

Fe has also shown promise as an ORR catalyst although usually in SA – not SAA – configurations, often coordinated within nitrogen-doped carbon frameworks. These materials nonetheless frequently have challenges with metal aggregation and conductivity.^386–388^ Niu et al. therefore attempted alloying dilute Fe into Cu particles, supported on top of nitrogen-doped carbon sheets, attempting to mitigate these issues.^387^ Here they showed that the FeCu SAA, with structure characterized by STEM and XAS, gave an increase in activity compared to commercial catalysts, with current density of 12.8 mA/cm^2^_geo_ at 0.9 V RHE. This was proposed to be due to charge asymmetry, which was claimed to accelerate the adsorption of oxygen and limit poisoning by intermediates; however, the exact mechanism was not discussed. This material showed minimal deactivation of Fe active centers while the Cu was suggested to enhance conductivity when compared to Fe–N_4_ SACs. While XAS showed changes in coordination environment and charge transfer between the Fe and Cu, there was not more specific mechanistic insight.

Tuning selectivities of ORR to the two-electron reduction path for H_2_O_2_ synthesis has also been attempted with SAAs but is less explored. Dong et al. demonstrated this concept using a NiCo SAA stabilized by embedding in a graphene aerogel.^389^ In this system, DFT was used to suggest that Co served as the primary active site for O_2_ adsorption and activation, while Ni was proposed to facilitate reduction to H_2_O_2_ and subsequent OH radical formation for a subsequent reaction. This synergistic division of roles enabled sufficient *in situ* H_2_O_2_ generation to drive the “electro-Fenton” degradation of organic contaminants, highlighting the potential utility of SAAs in selective ORR chemistry beyond energy conversion applications.

In summary, across SAA electrocatalysts for ORR, the isolated atom has been implicated to function as the active site or only as an electronic modifier in different cases. In Pt-group/Au and Pt-group/Ag SAAs, O_2_ adsorption has been predicted to occur at sites bridging both the guest and host, while isolation of the Pt-group atom is suggested to weaken CO adsorption and improve poisoning tolerance. Similarly, PtCo has been argued to promote the four-electron pathway through an OOH* intermediate bridging Pt and Co. In contrast, isolated Au within Pd has been suggested to primarily modify the intrinsic activity of the neighboring Pd site. Thus, available studies do not support a universal guest- or host-centered ORR mechanism. Instead, the pathway depends on relative oxygen affinities and can involve binding to the host, guest, a guest–host interfacial site, or even an oxidized cluster formed under reaction conditions. Trends for the two-electron H_2_O_2_ pathway remain unclear because substantially fewer SAA systems have been examined.

#### 4.2.2 Carbon Dioxide Reduction

The electrochemical reduction of carbon dioxide is of growing environmental and industrial significance.^390–394^ Converting CO_2_ into value-added fuels and chemicals offers the possibility of carbon-neutral or even carbon negative chemical production. However, most catalysts suffer from intrinsic trade-offs between CO_2_ activation, CO binding, and competition with HER.^391,395,396^ C–C bond formation is also a particular challenge. Very strong binding of adsorbates can poison the surface and suppress further reduction. Moderate binding (for example on Cu) can lead to high rates toward a range of products, while weak binding (for example on Au) leads to high selectivity toward CO.^397^ SAAs can provide unique means to tune reactivity through combined electronic and geometric effects, in turn modulating the binding strength of CO or other intermediates to promote C–C coupling or suppress HER, for example.

Xie et al. suggested that introducing Bi as a single atom dopant into Pd significantly weakens H adsorption, thereby suppressing the hydrogen evolution reaction (HER) and improving CO_2_RR selectivity toward CO.^398^ They attributed this to electronic effects from Bi donating electron density to Pd. It was also argued that Bi incorporation simultaneously lowers the reaction barrier for *COOH formation, enabling faster and more efficient CO production with Faradaic efficiency (FE) of 91.8% at −0.31 V vs RHE. While DFT was able to provide some support for these effects, complementary *in situ* spectroscopic techniques might further clarify the relative binding modes and affinities of reaction intermediates on Bi modified Pd sites.

In their investigation of CO versus HCOOH selectivity, Li et al. demonstrated that the relative adsorption energies of *COOH and *HCOO intermediates alone are insufficient to rationalize product selectivity.^399^ They first compared *COOH and *HCOO across experimentally reported Cu-based SAAs, including PdCu, BiCu, PbCu, InCu, AgCu, SnCu, and SbCu and predicted that *HCOO is thermodynamically preferable on each despite different experimental trends in product selectivity. They further investigated potential-dependent activation barriers and microkinetic models for PbCu and SbCu, which predominantly produce HCOOH and CO respectively. Their analysis revealed that PbCu preferentially forms HCOOH via the *COOH pathway rather than the conventional *HCOO route, in agreement with experiments.^52^ They attributed this to *COOH having an activation barrier of 0.08 eV, compared to 1.44 eV required for *HCOO. Likewise, the predicted high CO selectivity on SbCu also agreed with experiment,^400^ with conversion from *COOH to CO having a barrier of 0.17 eV compared to 0.47 eV for the *HCOOH pathway. These results emphasize that product selectivity cannot be inferred from intermediate adsorption energies alone but requires consideration of potential-dependent activation barriers within the complete reaction network.

Wang and colleagues thoroughly investigated CO_2_RR with dilute Pd in Au at various Pd loadings and showed that highly dispersed Pd atoms can activate CO_2_ effectively while resisting CO poisoning and mitigating HER at potentials above −0.5 V vs RHE, as seen in Figure 40.^390^ This study excelled at showing how ensemble size and weight loading can modify the reaction pathway and active sites, which is often overlooked when attempting to synthesize SAAs. Atomic dispersion was determined from XAS and STEM. While XAS signals were too weak to confirm atomic dispersion at the lowest Pd loading (2 wt %), the lack of nanoparticle features was taken as indirect evidence of single-atom dispersion. With Pd content below 5%, there was no appreciable Faradaic efficiency (FE; the amount of electrons used toward products as a fraction of the total applied) to formate. The FE toward CO exhibits a nonlinear behavior with Pd content, reaching a maximum of ∼ 80% at – 0.5 V for Pd_5_Au_95_. In contrast, the FE toward formate increases monotonically with Pd content, rising from 9.8% for Pd_5_Au_95_ to 56% for pure Pd at – 0.3 V. DFT corroborated these results: CO binding strength decreased with atomic dispersion of Pd, while *COOH intermediate stability increased, consistent with the lower overpotential for CO_2_ activation.

**Figure 40:**
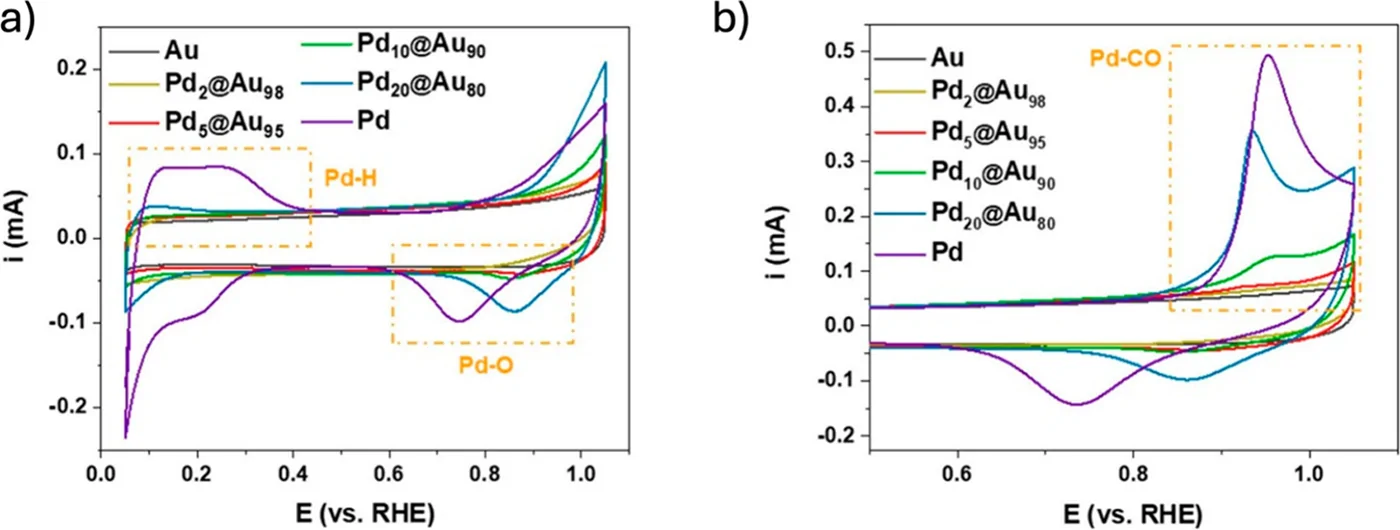
Cyclic voltammograms of various PdAu alloys in 0.1 M HClO_4_ a) with no reactant and b) CO stripping activity. Adapted with permission from ref.^390^ Copyright 2019 American Chemical Society.

Looking to enhance reduction to formate, Xue et al. explored doping Co into Sn.^401^ Sn is intrinsically selective toward formate due to strong binding of the O in *OCHO. Consequently, the next reduction step is energetically demanding. The authors suggested that Co–Sn orbital coupling electronically activates neighboring Sn sites, stabilizing the O-bound *OCHO intermediate and lowering the energy required for formate production. They found that Co_1_Sn maintained >92% formate FE from −100 to −1000 mA/cm^2^ and reached 99% FE above −1 A/cm^2^ from −0.83 to −1.33 V. This is yet another example where the SA may modify the adjacent host electronically to tune reactivity without being an active site itself.

Beyond CO and formate, a growing number of studies have targeted the generation of multicarbon (C_2_^+^) products using Cu-based SAAs, since Cu has well-documented abilities to produce a range of products.^329,394,402^ He et al. computationally examined how isolated dopants steer C–C coupling on Cu hosts by screening 29 Cu-based SAAs.^403^ Early transition-metal dopants created neighboring Cu sites with asymmetric *CO-binding strengths, thereby promoting *CO-*CO coupling toward C_2_+ products. Importantly, the authors distinguished between the reactivity of first-, second-, and more distant Cu neighbors, rather than treating all Cu sites surrounding the dopant as electronically equivalent. This work highlights how a single dopant can generate spatially distinct adsorption environments across the host surface. However, experimental validation remains challenging, and the analysis was limited to Cu(111)—less-coordinated facets, steps, and defects may exhibit different binding trends and reactivity.

Trying to balance CO_2_ activation capability and moderate CO binding strength (to enhance the C–C coupling while not overbinding), Wang et al. explored AgCu SAAs, that showed 65.1% FE at −1.22 V toward C_2_ products through a proposed dual-site mechanism.^394^ *Operando* attenuated total reflectance–surface-enhanced infrared absorption spectroscopy (ATR-SEIRAS) showed *CHO and *COCHO intermediates on AgCu, and these species were not seen for pure Cu. The authors attributed an increase in the *COCHO peak with cathodic potential to increased production by asymmetric CO–CHO coupling. To supplement this, they used DFT to conclude the initial activation of CO_2_ was on Ag, but then once converted to CO, surface diffusion to the Cu sites was promoted and expedited C–C coupling. They further showed improved reactivity compared to non-SAA AgCu nanoparticles, which showed an increase in ethanol selectivity from 0.41 ethanol: ethylene to 4.26, further supporting that the isolated Ag is an active site and critical to be dispersed.

Also exploring AgCu SAAs for CO_2_RR, Du et al. were able to achieve an FE of 78% toward C_2_ products at −0.65 V in a 1 M KOH flow cell with a AgCu SAA, compared to 56% with Cu nanoparticles.^329^ They screened a range of Ag loadings in Cu to gain insights on SAA, Ag nanoparticle, and combined single atom and nanoparticle compositions. It was found that the AgCu SAA and nanoparticle mixture outperformed the SAA alone in FE to C_2_ products with an FE of 94% at −0.65 V. However, too large of nanoparticles without single atoms caused a decrease due to the lack of adjacent Cu sites. The authors attributed high activity to the Ag nanoparticles converting CO_2_ to CO, which then cascaded to the Ag single site for further C–C coupling on the single atom and Cu sites. This was supported with CO reduction tests where a AgCu SAA had a higher FE than Ag single atom sites as well as nanoparticles in the Cu host. The authors performed DFT to further support experimental results and found that Ag-promoted C–C coupling at the Ag/Cu interface further enhanced C_2_^+^ formation, but Ag was also the active site for reduction of CO_2_ to CO before cascading to Cu.

Wang et al. examined the inverse scenario with a Cu_1_Ag_99_ SAA catalyst and reported enhanced CO selectivity and effective HER suppression through atomic Cu incorporation.^366^ Extensive structural characterization using STEM, EDS, and XPS confirmed the initial atomic dispersion; however, under applied potential in the presence of CO_2_, Cu migration to the surface was observed, albeit with sustained activity and selectivity. Once the Cu migrated to the surface, it enabled more Ag–Cu interfaces that were the preferred reaction site for CO_2_. This study was exemplary in showing structure rearrangement and tracking the cause for change in selectivity instead of solely relying on prereaction characterization. These findings raise important questions regarding the dynamic nature of SAAs during electrochemical operation and emphasize the necessity of rigorous *operando* and postreaction characterization when claiming true SAA behavior.

Chhetri et al. showed that PdCu SAAs can significantly improve the FE of CO_2_RR toward carbon-containing products; the FE to C_2_H_4_ increased from 16% to 33% compared to Cu nanoparticles. The goal in isolating Pd was to mitigate the competitive HER. They also synthesized NP alloys for the PdCu and showed no improvement in CH_4_ and C_2_H_4_ production. The alloys worsened HER due to appearance of Pd–Pd ensembles, emphasizing the beneficial role that SA Pd plays. DFT calculations rationalized these results in terms of suppressing HER and promoting C–C coupling in the presence of preadsorbed CO on the Pd site.^404^ This work also contains notably transparent discussion of the limitations of the testing, such as changes in NP size and facet changes, nonetheless building a strong case that PGM isolated atoms can improve CO_2_RR in Cu.

Exploring Cu in the limits of both dopant and host, Du et al. systematically explored Cu_1_In_99_ and Cu_99_In_1_ systems to disentangle the role of nominally inert In as a dopant on Cu reactivity.^405^ For the Cu_99_In_1_ configuration, atomically dispersed In sites significantly modified the CO binding strength on neighboring Cu atoms, resulting in an FE of 91% toward CO at −0.7 V. This was attributed to suppressing HER and lowered CO binding energy which was concluded from a combination of DFT and ATR-SEIRAS. However, increasing In content to 5% and 8% did not improve FE, which was attributed to the atomic configuration allowing less CO on the surface, with FE decreasing to around 80%. In contrast, isolated Cu atoms embedded within an In host featured negligible linear-bound CO and showed HCOO intermediates, thus promoting formate formation with a similarly high FE of 91% at −1.2 V. Pure Cu catalysts without atomic In incorporation exhibited poor suppression of the HER and produced a broad distribution of C_1_ and C_2_ products, illustrating how host-dopant interactions can selectively steer CO_2_RR pathways.

AlCu SAAs have been explored by both John et al. and Wang et al. through computational efforts.^406,407^ Both studies establish that the positively charged, oxophilic Al stabilizes oxygenated intermediates on Cu. However, they did not come to the same conclusion on whether this promotes C_1_ or C_2_ product formation. John et al. predicted that Al stabilizes *CHO but pushes *CO away from Al sites, thus suppressing C–C coupling. Conversely, Wang et al. suggest that Al promotes *CO retention and therefore encourages C–C coupling and stabilizes C_2_ intermediates when forming ethanol. The disagreement likely arises from their different choices of adsorption configurations, treatments of solvation, and which elementary steps were emphasized. This highlights the sensitivity and difficulty that persist when it comes to predicting candidate SAAs and mechanisms for electrochemistry and how DFT alone cannot provide definitive evidence for reactions.^176^

Xie et al. experimentally tested an Al_1_Zn_1_Cu alloy.^176^ The Al, similar to the computational work above, was suggested to promote CO_2_ activation and encourage CO binding on Cu. The Zn was included to aid in water dissociation to provide hydrogen. The authors achieved a C_2_ FE of 91.4% with a partial current density of 1.5 A/cm^2^ with the catalyst. These results support a synergistic mechanism, in which Al would likely supply carbon-containing intermediates and Zn would supply reactive hydrogen to promote asymmetric *CHO-*CO coupling. The C_2_ product formation aligns more closely with Wang et al.’s DFT results above, although asymmetric *CHO-*CO coupling is somewhat different than either computational analysis proposed. However, evidence for isolated Al was primarily limited to STEM and was not corroborated by *operando*, *in situ*, or postreaction XAS. Consequently, the coordination and stability of Al under reaction conditions, and its specific contribution to the active site, remain uncertain.

Methane is another CO_2_ reduction target due to its high energy density and widespread use as a chemical feedstock. DFT studies have identified M@Cu(211) bimetallic SAA interfaces as promising platforms for enhancing CH_4_ selectivity by modulating the relative binding of CO and H, with the stepped facets showing more activity than extended surfaces.^408^ Similarly, Li et al. experimentally and computationally demonstrated that alloying single Co atoms into Cu significantly shifts CO_2_RR selectivity toward CH_4_.^409^ They proposed that the introduction of isolated Co atoms alters the preferred *CO adsorption geometry from linear to bridge-bound configurations on Co–Cu sites, which suppresses C–C coupling pathways while facilitating *CO hydrogenation as seen in Figure 41a. Kinetic isotope studies were interpreted as indicating that isolated Co atoms facilitate water activation near bridge-bound CO, enabling a direct, water-mediated proton-coupled electron transfer to the carbon intermediate. The authors contrasted this proposed pathway with a surface-H pathway on pure Cu, in which H generated by water dissociation must migrate across the Cu surface before hydrogenating CO. However, the specific sites responsible for H generation and migration on pure Cu were not identified. IR further showed that CoCu featured bridge-bound CO as opposed to just linearly bound CO on Cu, which was also suggested by DFT. The calculations indicated that bridge-bound CO can undergo deep reduction to CH_4_, whereas linear-bound CO prefers to desorb instead of further reduce. The CoCu catalyst achieved an FE of 62.3% toward CH_4_ at current densities up to 600 mA cm^–2^ (Figure 41b), markedly outperforming pure Cu catalysts, which reached a maximum of 39.9% CH_4_ selectivity at significantly lower current densities (∼226 mA cm^–2^).

**Figure 41:**
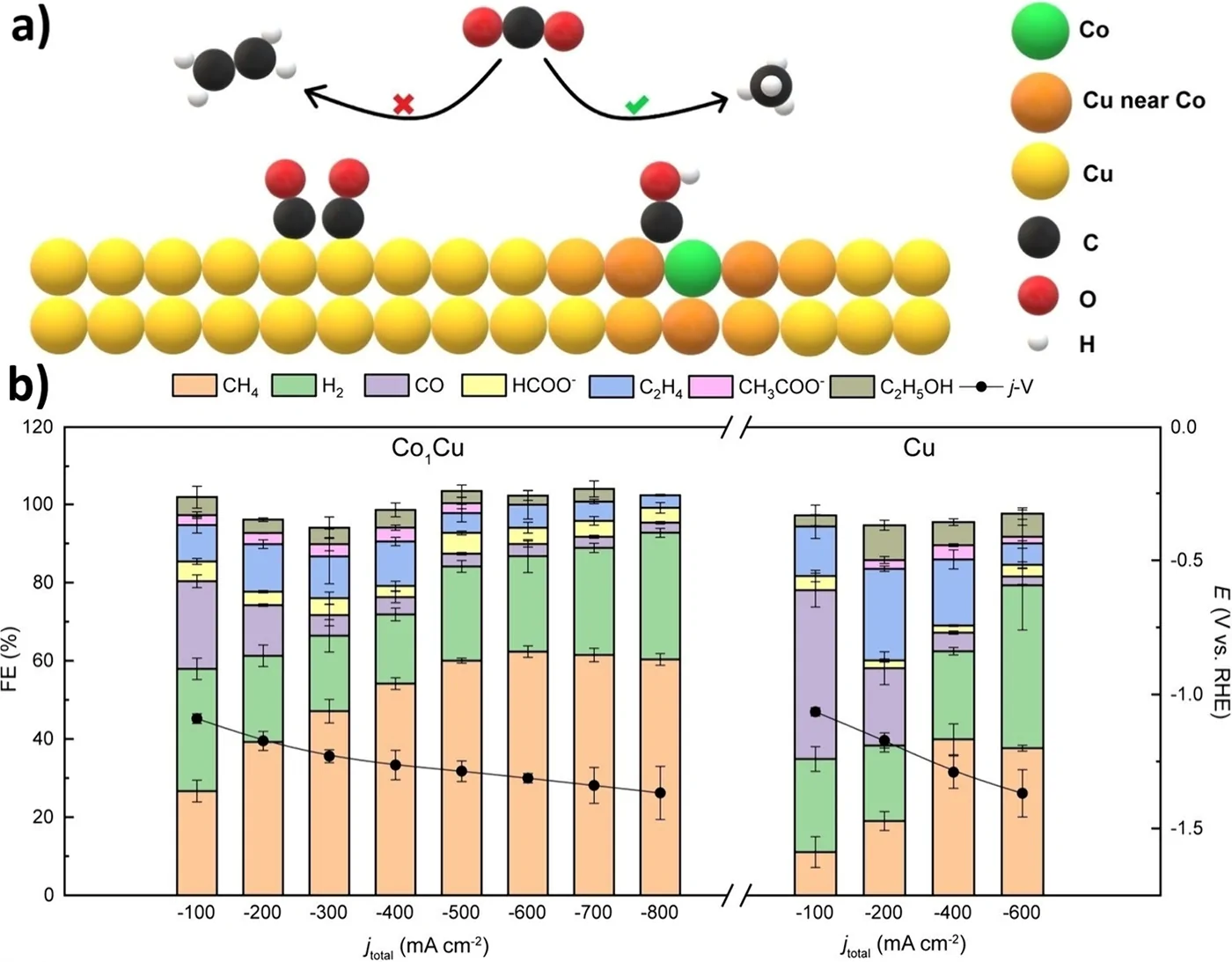
a) Proposed CO_2_RR reaction mechanism on Cu and Co_1_Cu. b) FEs to products and corresponding potentials at different current densities for Co_1_Cu and Cu. Adapted with permission from ref.^409^ Copyright 2025 Wiley-VCH GmbH.

Mixed product selectivity has additionally been explored in the context of process intensification with tandem reactors. Wang et al. explored CO_2_RR on Sn_1_Cu to generate the proper ratio of ethylene and CO that could be fed into a thermal reactor for subsequent hydroformylation to propanal.^410^ They hypothesized that Sn could increase the coverage of CO on Cu to enable C–C coupling. Experimentally, pure Cu gave ∼ 25% FE toward ethylene at −1.8 V and ∼ 30% toward CO at −0.8 V. With the single atom Sn, there was distinct selectivity at differing potentials: ethylene FE was maximized at 47.8% at −1.8 V RHE while 87.5% FE toward CO was seen at −0.7 V. The material was robust to switching potentials during electrolysis to make the ideal feed stream for hydroformylation. The authors provided a multitude of experimental and computational evidence to attribute the changes to SAA structure. With DFT it was found that the Sn electronically modifies the neighboring Cu. This was backed up with ATR-SEIRAS where Sn_1_Cu showed three adsorption motifs on the surface compared to pure Cu which has only one. Additionally, the bridge bound CO showed two desorption regimes, giving evidence for the stronger binding of CO that was predicted in DFT. Shifts in redox states of Sn from IV to 0, shown in Sn Mössbauer spectroscopy, also supported the electronic influence of Cu on Sn. Though not completely conclusive, there is good evidence that the Sn-adjacent sites could be the active ones.

Beyond pure CO_2_ reduction, schemes have emerged for coupling of multiple substrates, such as simultaneous C–C and C–N bond formation for amide synthesis. Wang et al. investigated Cu-based SAAs for the coreduction of CO_2_ and N_2_ to acetamide.^411^ A detailed computational screening was performed with 25 transition metals, sequentially evaluating thermal stability (based on formation energy), binding affinities for reactants, reduction of individual reactants and selectivity for HER. They identified WCu(111) as the most promising catalyst. Single atom W modifies the electronic structure of neighboring Cu to favor linear *CO binding and subsequent C–C coupling, forming a key *CCO intermediate. While N_2_ binds only weakly to pristine Cu, this study indicated that W incorporation can enhance N_2_ adsorption and enable its protonation, ultimately facilitating coupling between *NH and *CCO species to form adsorbed *CONH_2_ intermediates. While this computational study by Wang et al. is a useful starting point, the specific material has not been experimentally explored, and more detailed stability studies could also be useful (see Section 2.1).

To conclude the survey of SAAs for CO_2_RR, it appears generally that the host element retains much of its characteristic product-forming behavior, which is not surprising given that a majority of elements are CO_2_RR-active individually. The isolated guest can then modify the propensity toward CO_2_ activation, water dissociation, hydrogenation, or relative intermediate stability to steer selectivity. Such trends are particularly evident for Cu-rich hosts, where electronically modified Cu sites neighboring the isolated dopant are often proposed to perform hydrogenation or C–C coupling, while the isolated guests tend to be implicated in altering the generation, coverage, or adsorption configuration of CO-derived intermediates. For PGM dopants such as Pd, the promotion of CO_2_ activation and the formation or retention of CO-derived intermediates has been attributed to the isolated site, which also limits HER associated with larger PGM ensembles. The host can then influence product selectivity, with Au-rich hosts favoring CO and Cu hosts enabling further hydrogenation or C–C coupling. More broadly, selectivity cannot be predicted from simplistic metrics such as CO-binding strength alone, as it depends collectively on adsorption geometry, hydrogen availability, and the identity and proximity of neighboring sites.

#### 4.2.3 Nitrogen and Nitrate Reduction

Electrochemical reductions of either dinitrogen (N_2_RR) or nitrates (NO_3_RR) represent a potentially transformative opportunity to supplement the energy and infrastructure intensive Haber-Bosch process with distributed scale production of ammonia.^412^ Dinitrogen reduction is notoriously challenging due to the strength of the N–N triple bond, 945 kJ/mol,^413–415^ and the competing HER, while nitrates are comparatively easier to activate. SAAs provide an avenue in both cases to modulate adsorption energies of N_2_, NO_3_^–^ and H_2_ through electronic shifts or bifunctional active sites.

Han et al. reported PdCu dimers isolated in N-doped carbon (NC) for N_2_RR. While not precisely an alloy, the isolated sites have commonalities with SAAs due to electronic interaction between the two constituents. These interactions are also easier to directly identify. Here, Pd was suggested to act as the initial active site while Cu electronically modifies the Pd to facilitate electron transfer to adsorbed nitrogen.^416^ Similar to CO_2_RR, HER production was suppressed by isolation of Pd. The Cu was chosen to further help the desorption of hydrogen and also modulate the reactivity of Pd. XAS and HAADF-STEM confirmed uniform atomic dispersion and shifts in both the Pd 3d and Cu 2p XPS peaks relative to the single metal catalysts indicated that formation of the Pd–Cu sites altered the electronic structures of both metals. DFT also revealed that alloying shifts the Pd d-band center toward the Fermi level, enhancing d–2π* coupling with N_2_ and thereby lowering the activation barrier toward hydrogenation to NNH. The calculations suggested that the N–N hydrogenation barrier required a lower overpotential than HER in the SAA, which showed improved selectivity to NH_3_ compared to Pd with an FE of 24.8% at −0.45 V RHE.

Chen et al. observed similar effects with a PdCu SAA (conventional dopant in host) configuration, for NO reduction, where the weaker N–O bond facilitated nitrogen activation. This was tracked using FTIR as shown in Figure 42.^417^ Here they achieved 85% FE to NH_3_ at a current density of 210 mA/cm^2^. They attributed the facile activation of NO on PdCu to charge asymmetry when binding on the bimetallic hollow site as opposed to the hollow site of pure Cu. Differential electrochemical mass spectrometry (DEMS) was used to track reaction products and it was inferred that PdCu and Cu form a prominent intermediate of NH_2_OH, suggesting initial formation of *NHO in the pathway. Using DFT, they calculated the rate-determining step (RDS) to be NO reduction to NHO, with PdCu requiring 0.52 eV compared to 0.84 eV for pure Cu.

**Figure 42:**
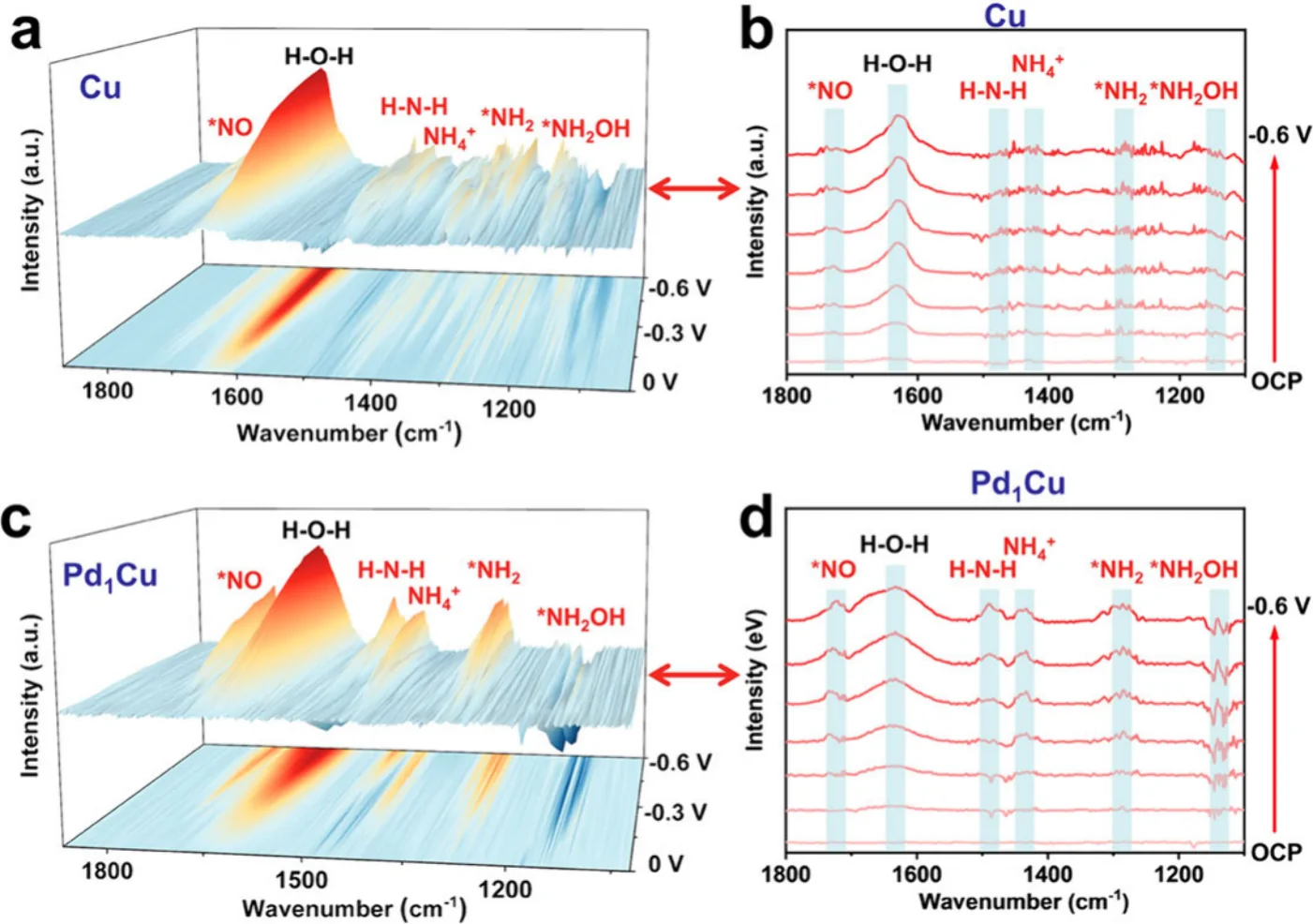
FTIR spectra for a,b) pure Cu and c,d) PdCu SAA *in situ* from 0 to −0.6 V showing bound species during NO reduction reaction in 0.5 M Na_2_SO_4_ saturated with NO/Ar. Adapted with permission from ref.^417^ Copyright 2024, American Chemical Society.

PdCu SAAs have also been explored for NO_3_RR. Li et al. investigated dilute PdCu alloys, suggesting a dual-site reaction pathway enabled by atomic Pd dispersion.^418^ They used XPS and HRTEM to provide evidence for electronic interaction between Pd and Cu and show the absence of Pd clustering. NO_3_^–^ removal rates were measured on PdCu, Pd, and Cu with and without sodium sulfite, used to selectively poison Pd, to understand the role of different sites. When sodium sulfite was present and the Pd was thus inactive, there was an accumulation in solution of NO_2_^–^ and NH_3_–N intermediates, suggesting that nitrate is initially reduced to nitrite on Cu sites, followed by conversion of nitrite to NH_3_ on Pd single atom sites through H* donation. Importantly, this catalyst also showed strong sulfate poisoning resistance, which is a necessary property for wastewater treatment. In another example, Du et al. achieved 94.9% FE at −0.6 V to ammonia in neutral media using a PdCu SAA.^419^ To avoid HER, they isolated the Pd sites, and to further evaluate the effect of Pd loading, they synthesized PdCu alloy nanoparticles and pure Pd nanoparticles on Cu. While NH_3_ yield increased with the concentration of Pd, the FE decreased. DFT calculations suggested that Pd was responsible for adsorption of NO_3_^–^ and further reduction. When compared with Pd nanoparticles supported on Cu, the atomically dispersed alloy exhibited higher ammonia yield and selectivity, confirming that atomic dispersion was critical for improving catalytic performance.

Similar to the PdCu SAAs mentioned above, CoZn SAAs have been proposed to permit a bifunctional effect in NO_3_RR.^420^ Chen et al. synthesized isolated Co in Zn nanosheets and achieved current densities of 2.4 A/cm^2^ with a FE of 98.7% toward NH_3_ at −0.8 V. They combined *in situ* FTIR and hydrogen adsorption experiments with DFT to support that Co sites dissociated water and would undergo H spillover to the neighboring Zn sites, thus facilitating hydrogenation. Each of these papers supports that in tuning an alloyed SA to prefer *H spillover before HER, it can facilitate hydrogenation to NH_3_.

Using a NiCu SAA, Cai et al. reported nearly 100% FE for NO_3_RR to ammonia under ambient conditions at a production rate of 326.7 μmol h^–1^cm^–2^ at – 0.55 V vs RHE.^421^ DFT calculations indicated that the Ni dopant may selectively stabilize and bind an NOOH* intermediate, suppressing side pathways and desorption of NO_2_^–^. In a complementary study, Yi et al. developed a unified descriptor linking H* adsorption and *NO_*x*_ hydrogenation kinetics to the d-band center, enabling the identification of NiCu as an optimal Cu-based single-atom alloy. Experimentally, NiCu delivered ∼ 95% FE at – 0.6 V vs RHE, attributed to Ni-induced electronic effects that enhance H* availability and lower hydrogenation barriers. These properties in turn suppress HER due to rapid consumption in *NO_*x*_ reduction steps^422^ —the authors showed that while HER is still possible due to the presence of *H, it was consumed 4x faster on NiCu as compared to Cu. The two aforementioned papers show a progression in mechanistic understanding, shifting from implication of the purely electronic effect of increasing *NOOH binding strength to additionally including a descriptor for coupled control of *H generation and *NOx hydrogenation. This highlights the broader theme that numerous complementary studies going beyond simple synthesis and testing are often necessary to fundamentally understand reaction pathways on SAAs.

Ye et al. explored a spillover-driven reaction mechanism with multiple SA dopants, using Mo and Fe atoms embedded within a Pd bulk lattice for the electrochemical NO_3_RR.^180^ In this dual SAA system, Mo and Fe atoms were spatially isolated from each other but suggested to benefit from electronic coupling between sites. STEM and XAS confirmed the lack of Mo or Fe ensembles and showed even dispersion throughout the Pd. Mechanistically, the authors proposed that nitrate and early NO_*x*_ intermediates preferentially adsorb and are activated on the oxophilic Mo and Fe sites, while subsequent hydrogenation steps occur on neighboring Pd regions through an intermediate spillover process. A mechanism was proposed using insight from ATR-IR and analysis of partial current densities on control samples of Fe_1_Pd, Mo_1_Pd, and Pd. The partial current densities to NO_2_^–^ were found to be highest on Mo_1_Pd and decrease in the order Mo_1_Pd > Pd > Fe_1_Pd > Mo_1_Fe_1_Pd. NH_3_ however followed the trend Mo_1_Fe_1_Pd > FePd > MoPd > Pd, which suggests that Mo promotes NO_2_^–^ formation while Fe conversely promotes NH_3_. ATR-IR and DFT studies with only one of the dopants showed intermediate binding shift and changes in reaction free energy depending on the dopant, indicating independent roles in the Mo_1_Fe_1_Pd alloy. The cooperative pathway shown in Figure 43 was supported by DFT calculations, which showed that Mo and Fe lower the energy barriers for NO_3_^–^ activation and early protonation steps, while Pd provides favorable energetics for later hydrogenation toward NH_3_. The spillover of reaction intermediates between dopant and host sites was also argued to suppress competing HER for high ammonia selectivity and production rates with FE up to 94% at −0.7 V.

**Figure 43:**
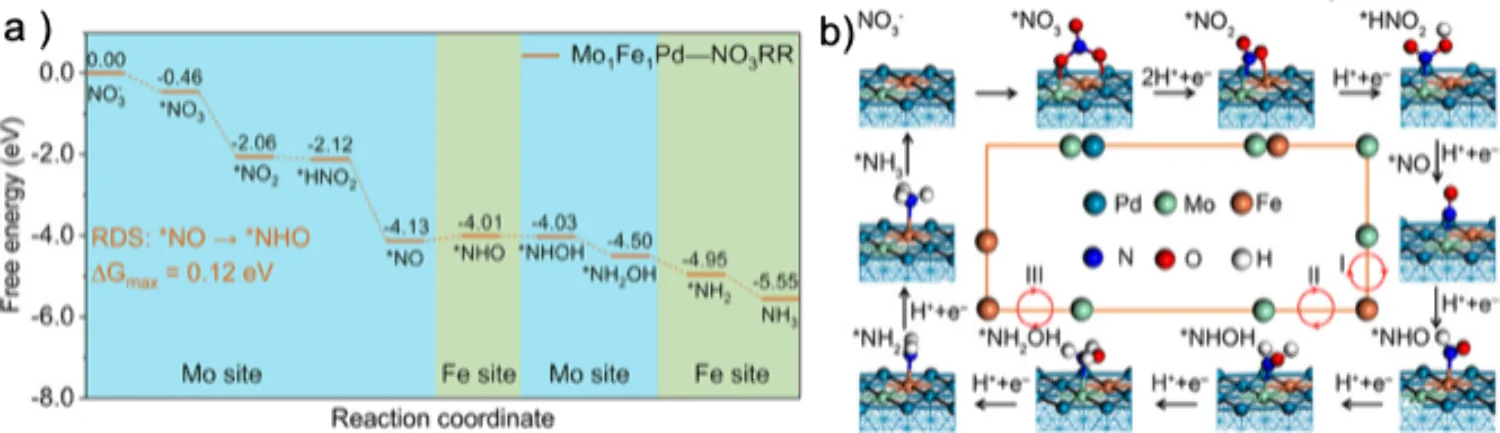
a) Gibbs free energy diagram for NO_3_RR over the Mo_1_Fe_1_Pd catalyst. b) Structural model of ideal spillover pathway from Mo and Fe sites to Pd. Adapted with permission from ref.^180^ Copyright 2025 Wiley-VCH GmbH.

Zhang et al. explored AuCu SAAs for NO_3_RR, also studying the effect of Cu vacancies generated by acid leaching.^423^ They hypothesized from XPS and XRD results that the neighboring Cu may have electronic changes due to charge redistribution caused by vacancies in the Cu lattice and also from the Au single atoms; this was supported by charge density analysis using DFT. The authors attributed some of the change in catalytic activity to Au increasing the strength of H* binding on Cu to avoid HER. The catalyst achieved an FE of 98.7% toward NH_3_ at −0.2 V RHE.

Gao and co-workers proposed a similar ligand-modification mechanism between Au and Cu but used an ordered intermetallic shell to obtain site-isolated structure.^424^ They synthesized both a dilute, nonintermetallic shell and a higher concentration intermetallic shell (tetragonal *P*4/*mmm* phase) which were well characterized with XAS and HAADF-STEM. In the dilute material, individual Au atoms were randomly dispersed within an fcc Cu lattice. In the ordered CuAu intermetallic structure, the Au atoms were periodically arranged but remained completely isolated from one another by Cu. The ordered SAA reached an NH_3_ FE of 85.5% at – 0.5 V and a yield rate of 8.47 mol NH3/h·g at – 0.6 V, while retaining its FE over 20 electrolysis cycles and showing no apparent current loss during 12 h of continuous operation. DFT supported that the neighboring gold sites inhibited HER, while also lowering *N binding to promote hydrogenation.

Yin et al. and Yu et al. also independently showed that AuCu SAA catalysts are effective for NO_3_RR to ammonia.^425,426^ In both studies, atomically dispersed Au within a Cu host dramatically enhanced activity relative to pure Cu, with Yin et al. reporting an NH_3_ FE of ∼ 99.7% at – 0.80 V vs RHE and Yu et al. achieving >90% FE across a wide potential window with long-term stability at industrially relevant current densities of 1 A/cm^2^. Both hypothesized that isolated Au atoms act as the primary sites for nitrate adsorption and early reduction steps, while the Cu host facilitates subsequent hydrogenation as seen in Figure 44. DFT calculations in both works showed that Au incorporation may lower the adsorption energy of NO_3_^–^-derived intermediates and accelerate the reduction of *NO_2_ and *NO relative to pure Cu, while suppressing N–N coupling pathways that would lead to N_2_ or N_2_O formation. Yin et al. focused on validation of this mechanism using *operando* DEMS to detect NO_2_ and NO intermediates during catalysis, whereas Yu et al. studied macroscopic activity trends, wide-potential operability, and durability. There remains a broader need for further studies to more confidently assign a mechanism and to clarify means to suppress HER while still rapidly consuming NO_*x*_ intermediates.

**Figure 44:**
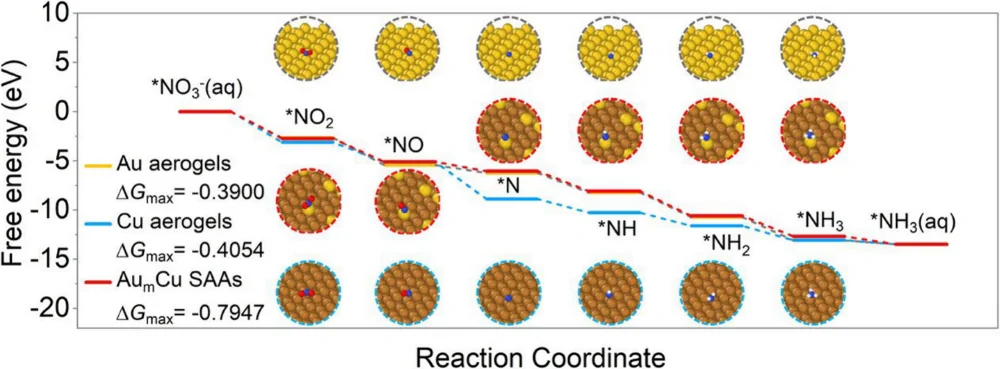
Reaction pathway of NO_3_ on Au, Cu, and AuCu aerogels at −0.8 V vs. RHE. Adapted with permission from ref.^426^ Copyright 2024 Wiley-VCH GmbH.

RuCu SAA nanowires have also shown notable performance for NO_3_RR, even sustaining ammonia FE of 93% at industrially relevant current densities of 1 A/cm^2^ and >99% nitrate conversion.^427^ This performance was attributed to an ability of Ru to activate nitrate, coupled with suppression of HER through both isolation of Ru sites and low activity of the Cu host. Detailed characterization using X-ray techniques suggested that the Ru atoms were indeed isolated in the Cu host, and in a metallic state. DFT indicated that the Ru sites are selective toward ammonia, with nitrate activation outcompeting HER. Interestingly, through the synthesis process the RuCu nanowires became highly porous while growing from 50 to 100 nm, which led to a 5-fold increase in the ECSA. This paper is a good example of combined kinetic and characterization studies that illustrate the impact of SAA structure. XAS, PDF, STEM, and atom probe tomography were all used to diagnose single atom Ru while acknowledging the limitations of each technique alone. This in combination with the DFT results gave strong evidence that the isolated Ru atoms are the key active site in this reaction. Further clarification of where the hydrogen involved in the formation of NH_3_ is sourced/formed on the surface could enhance the mechanistic insights already gained.

Computational screening studies have also shown an increasing importance in SAA electrocatalysis for N_2_RR and NO_3_RR. DFT screenings, such as work on TM@Ru(0001) materials by Kour et al., have expanded general understanding by identifying favorable adsorption energies, low hydrogenation barriers, and inherent HER suppression across different transition-metal dopants.^428^ Rivera et al. performed a detailed study with DFT to systematically examine 88 SAA configurations, spanning host metals (Y, Ti, Ni, Pd, Pt, Cu, Ag, and Au) and single atom dopants (Mo, W, Fe, and In).^429^ From this analysis, the authors concluded that the relative preference of reaction intermediates to bind to either the single atom site or the host metal governs the free-energy landscape of electrochemical nitrate reduction. This site preference was largely interpretable through electronic structure descriptors, such as the *d-*band center and Fermi level. The study further claimed that early transition metals, when used as host surfaces, tend to bind nitrogen-containing intermediates too strongly and readily promote the competing HER. However, when these early transition metals are introduced instead as single atom dopants within more inert noble metals, their strong binding character can be moderated, enabling favorable stabilization of key NO_3_RR intermediates while suppressing HER.

Across SAAs for N_2_RR and NO_3_RR, common mechanistic themes emerge, but trends are substantially clearer for NO_3_RR than for N_2_RR, for which relatively few SAA systems have been reported. For NO_3_RR, Cu has been the most commonly investigated host and isolated guest atoms are generally proposed to introduce complementary sites for nitrate activation or subsequent hydrogenation. However, the attributed division of these roles is not universal. Isolated Pd or Ni have been proposed to primarily promote H* formation and hydrogenation of NO_*x*_-derived intermediates, whereas isolated Au or Ru have been argued to facilitate nitrate adsorption and activation before downstream conversion on Cu. A more specific trend has been proposed for PGM SAAs. Isolating Pd or Ru within Cu retains their strong reactivity toward nitrogen containing intermediates while limiting contiguous PGM sites that would favor HER. The Cu host additionally moderates NO_x_* intermediate and H* binding, although it may participate directly in nitrate-to-nitrite reduction or later hydrogenation depending on the guest. Thus, the recurring design principle is not assignment of nitrate activation to one element, but spatial separation of NO_*x*_ activation and hydrogenation across isolated guest and host sites. This separation must balance sufficient H* availability for ammonia formation against excessive H_2_ evolution.

### 4.3 Oxidative Electrocatalysis

Oxidative electrocatalytic reactions present unique challenges compared to reductive processes, primarily due to corrosion and other degradation pathways for catalysts under high anodic potentials or voltage cycling. Catalysts may undergo dissolution as well as physical degradation through deep oxidation–reduction cycles. Even noble elements that are nominally quite stable can be susceptible to such loss mechanisms. SAAs may leach above or below individual metals’ dissolution potentials due to the host altering the dopant properties, though even very slow corrosion may have drastic impact due to the low total quantity of dopant elements. Stability issues with supports also arise more often in anodic conditions, including with supports such as carbon that degrades at high potentials. Common practical reaction targets therefore include low-potential oxidations such as the hydrogen oxidation reaction (HOR) and oxidation reactions of small organic molecules including formic acid (FAOR), methanol (MOR), and ethanol (EOR), all of which are useful as fuels for fuel cells. Organic oxidations are further complicated by sluggish kinetics, multistep reaction pathways, and the accumulation of terminally bound species such as *CO. Key elementary steps in organic electrooxidation generally involve substrate adsorption, selective O–H or C–H activation, and the generation of oxygen-containing species such as *OH from H_2_O or OH^–^.^430,431^ The binding energies of these oxygenated species often scale together, creating trade-offs between substrate oxidation, removal of poisoning intermediates, and surface blockage. SAAs may help separate these functions by using an isolated dopant for selective bond activation while the host supplies *OH, although site isolation may inhibit reactions requiring adjacent atoms for C–C cleavage. MOR has been the most extensively studied electro-oxidation reaction in the context of SAAs.^367,372,431,432^

Poerwoprajitno et al. investigated Pt single atoms dispersed on Ru nanoparticles for MOR. They confirmed atomic uniformity through TEM, XRD, and XAS.^367^ The PtRu SAA exhibited capacitance-corrected geometric current densities 3.1x higher than Pt islands on Ru and 13x higher than commercial PtRu alloy nanoparticles, without compromising stability. The total FE was 87%, compared to 63% on PtRu bulk alloy nanoparticles. The authors used DFT to improve understanding of the mechanism, showing that the adsorption of CH_3_OH is stronger on PtRu SAAs than conventional alloys, enhancing activation and thus activity. They also calculated that CO is bound strongly to PtRu alloys and further oxidation is uphill, whereas the PtRu SAA had weaker CO binding and subsequently downhill steps to CO_2_. Similarly, Ahmad et al. developed a PtRu SAA that achieved mass specific current density of 1.33 A/mg, based on total Pt and Ru, at 0.84 V RHE while maintaining excellent CO tolerance.^372^ In this system, the authors proposed that Ru donates OH species to facilitate CO oxidation while simultaneously weakening CO binding on Pt through electronic modifications. These claims were supported by DFT and XAS. Notably, the catalyst operated stably under acidic conditions, where OH-availability is limited and corrosion risks are elevated, highlighting the robustness of this single atom configuration.

Chen et al. investigated Ru single atoms doped into Pt nanoparticles for both MOR and HOR.^433^ Structural characterization using HAADF-STEM and XAS confirmed isolated Ru atoms within the Pt lattice. A slight negative shift in the Pt L_3_-edge and Pt 4f XPS peaks was attributed to electron transfer from Ru to Pt. These catalysts were reported to have specific activity 4.8 times higher than Pt/C nanoparticles for MOR. An earlier onset potential for CO oxidation and higher sustained current densities were attributed to lowered CO adsorption energy, as well as a lower barrier for CO–OH coupling. DFT calculations for MOR identified the CO–OH coupling step as rate-determining for the Pt, RuPt nanoparticle and RuPt SAA, as shown in Figure 45 **;** however, the barrier was significantly decreased once Ru was atomically dispersed. Under HOR conditions, the Ru-doped catalysts showed a sharper increase in current density with potential compared to Pt/C. Exchange current densities and mass activities were 13.3 and 15.7 times higher than Pt/C, respectively at 0.05 V. Shifts in hydrogen underpotential deposition peaks toward more negative potentials suggested weakened hydrogen binding.

**Figure 45:**
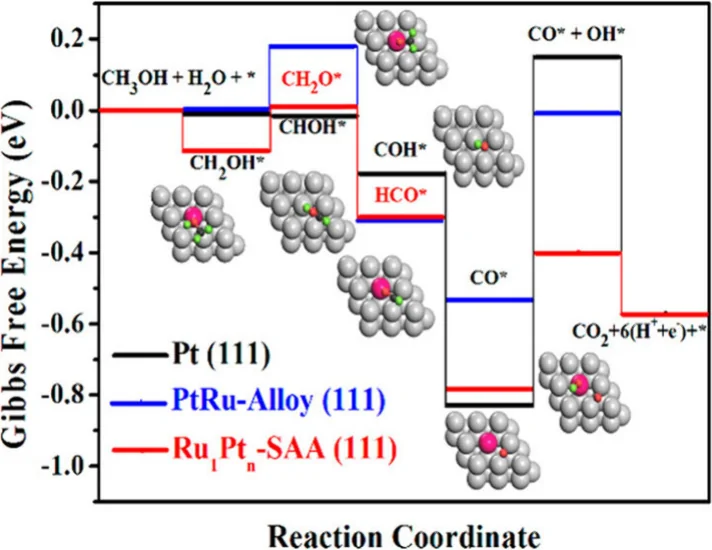
Calculated reaction free energies for MOR on Pt(111), PtRu-Alloy, and RuPt SAA. Adapted with permission from ref.^433^ Copyright 2022, American Chemical Society.

Moving beyond dual-metal systems, Liu et al. explored high-entropy alloy single atom catalysts (HEASAs) for methanol oxidation.^432^ HEAs are broadly characterized by containing a large number (at least 5) elements, which promotes alloying even when various binary subsystems would not be miscible.^434^ In the referenced work, Pt serves as the primary active site but was embedded within a multimetallic environment of Ni, Co, Mg, Bi, and Sn. These HEASA-Pt catalysts exhibited high mass activity and superior catalytic stability relative to Pt nanoparticles supported on the same alloy compositions. This was attributed to suppression of CO poisoning. When run in a DMFC, HEASA-Pt had a peak power of 22.8 mW/cm^2^, compared to 17.2 mW/cm^2^ for Pt/C, with only 20% of the Pt weight/cm^2^. DFT calculations revealed a downward shift in the d-band center of Pt in the HEASA-Pt surface compared to Pt(111), accompanied by increased d-orbital filling. XPS further supported electron redistribution, indicating that Pt acts as an electron acceptor in this configuration. While high entropy alloys can inherently convolute understanding of the active site(s), this work was notable in trying to distinguish the role of Pt by altering the loading of the alloys. A high entropy alloy containing Pt without single atom dispersion remained susceptible to CO poisoning, whereas the single atom Pt configuration was suggested to follow a CO-free oxidation pathway. The CO-hindered pathway can also still occur on the HEASA-Pt; however, the adsorption energy of CO was weakened by 0.7 eV on the HEASA-Pt compared to Pt(111), which was a greater effect than any Pt-bimetallic. Further work could address whether certain coordination environments around the Pt SA help promote the activity most.

Current examples of SAAs for EOR have only used single atoms hosted within Pt or Pd to facilitate C–C cleavage and/or help mitigate CO poisoning.^435–439^ Chen et al. explored Pt within Pd, using a tensile-strained PtPd SAA for EOR. The tensile strain was created by using Pd_5_Bi_3_ intermetallic nanocrystals, where Pt was added to the shell and Bi was electrochemically dealloyed to create a strained Pt_1_Pd shell.^440^ The performed DFT calculations indicated that tensile strain strengthened ethanol adsorption, and that isolated Pt accelerated oxidation along the C_2_ pathway while modestly lowering the *CH_2_CO C–C cleavage barrier from 0.76 to 0.66 eV. In situ FTIR detected acetaldehyde and acetate beginning at 0.2 V versus RHE, along with weakly bound CO, as compared with Pt/C. At 0.8 V, acetate remained dominant, with an FE of 83.2%, whereas only 14.5% was attributed to the C1 pathway to CO_2_/carbonate. While Pt was utilized efficiently, the main benefits were improved resistance to CO poisoning and rapid partial ethanol oxidation rather than predominantly complete oxidation to CO_2_.

Wang et al. studied the case of aerogels composed of Bi atomically dispersed on Pd nanowires, functionalized with ionic liquids.^441^ XPS revealed a lower Pd 3d binding energy, attributed to electron transfer from Bi to Pd. DFT calculations suggested that BiPd favors the C_2_ oxidation pathway with a lower required overpotential compared to Pd. Experimentally, the catalyst exhibited higher current densities, improved stability, and an earlier onset potential compared to Pd alone. During electrolysis, higher conversion of ethanol to acetate was observed on the SAAs with a conversion of 0.2 acetate: ethanol for Pd and 0.97 for BiPd, suggesting an improvement in the C_2_ four-electron pathway. The authors explored increasing the ratio of Bi to Pd; however, increased Bi caused activity to decrease due to Bi blocking active Pd sites, emphasizing the value of the SAA structure thus used Bi_1_Pd_50_ instead of Bi_1_Pd_5_. Additionally, it did not appear that Bi could promote C–C cleavage to oxidize to CO_2_. While the improvements to EOR were clear, it was not distinguished how much the IL affected the Bi and Pd respectively, which would be necessary to extrapolate and predict trends for various catalyst.

Finally, FAOR has been explored for fuel cells to minimize losses from indirect (CO-forming) oxidation pathways. Li et al. reported that Pt-decorated Pd nanocrystals exhibit earlier onset potentials and higher FAOR current densities than Pd/C catalysts.^442^ They spanned a composition series and saw activity increase when single atoms were evenly dispersed, but then a sharp decrease occurred once Pt islands started to form. Structural characterization using HAADF-STEM and EXAFS confirmed the presence of dispersed Pt species within the Pd matrix, while XPS showed minimal changes, suggesting that electronic effects may be limited in this system. Mechanistic interpretations based on DFT calculations attributed the improved performance to local electronic modification of Pd by neighboring Pt atoms. The PtPd SAA gave a current density of 5.2A/mg_metal_ at ∼ 0.85 V, and power densities up to 3x higher than those of commercial Pd/C fuel cells. While CO poisoning was decreased, it was not fully suppressed due to sites distant from Pt having little change from bulk Pd. The same group also investigated Au-doped Pd SAAs for use in direct formic acid fuel cells, aiming to improve on conventional Pt and Pd-based catalysts that suffer from CO poisoning through side pathways.^443^ Structural characterization confirmed the presence of Au single atoms within the Pd lattice, and XPS of higher loading samples revealed increasing Pd 3d binding energies with increasing Au content, attributed to charge transfer from Pd to Au and a corresponding downward shift in the Pd d-band center. Electrochemical measurements showed sustained current densities at higher potentials for AuPd SAAs, whereas Pd nanoparticles exhibited rapid deactivation above 0.9 V. These results suggest that Au promotes the direct oxidation pathway and mitigates CO poisoning through electronic effects.

Throughout electro-oxidation reactions, surface reconstruction is an especially critical issue to monitor, given the propensity of various metals to themselves oxidize under anodic polarization. Li and co-workers illustrated this issue well using an Ir_1_Ru single-atom alloy embedded in TiO_2_ nanowires while studying acidic water splitting—a reaction requiring conditions far too oxidizing for the vast majority of materials.^368^ The catalyst achieved a specific activity of 3.13 mA mF^–1^ at 1.53 V, outperforming Ru/TiO_2_, while the isolated Ir atoms also improved stability by suppressing excessive Ru oxidation and dissolution. *In-situ* XAS showed that the surface reconstructed into a thin, self-limiting IrRuO layer containing contracted Ru–O–Ir motifs. DFT supported that these motifs stabilized an HOO* intermediate, and inhibited deeper oxidation and lattice-oxygen participation. Notably, the active phase was not the initially synthesized metallic SAA structure, but a dynamically formed oxide surface whose structure and stability were regulated by the underlying Ir–Ru alloy and TiO_2_ support.

Despite limited work under anodic conditions, several trends arise for SAAs used in electro-oxidation. Isolated guest atoms are commonly found to provide a complementary function to that of the host rather than acting independently as the active site. This behavior has been invoked particularly for SAA PtRu catalysts, where organic adsorption and activation are generally attributed to Pt, while Ru or other oxophilic neighboring atoms are proposed to promote OH* formation and lower the barrier for oxidation of adsorbed CO* intermediates. Improved poisoning resistance has further been attributed to atomic isolation weakening the binding of CO* to Pt or maintaining proximity between organic-activating and oxygenate-supplying sites. A related but distinct trend appears for Pd-hosted organic oxidation catalysts, where isolated guests primarily have been suggested to modify pathway selectivity. Depending on the guest identity, these electronic and geometric effects may promote deeper oxidation, redirect the reaction toward less poisoning-prone pathways, or suppress the formation of strongly adsorbed CO*. Collectively, these studies suggest a mechanistic theme in which a PGM, whether as host or guest, will activate the organic reactant, while neighboring, more oxophilic sites may supply oxygenated intermediates or indirectly destabilize poisoning species. Maintaining atomic dispersion may be important for preserving this local division of catalytic functions, although guest-ensemble effects have not been systematically evaluated across oxidative SAA reactions.

### 4.4 Conclusions and Outlook in Electrocatalysis

Stability challenges, both structural and catalytic, remain some of the most significant barriers to widespread application of SAA electrocatalysts. Catalytic stability, the ability to stay reactive throughout the course of a reaction, is often limited by strongly bound intermediates such as CO on Pt. There have been many computational studies showing electrochemical screening and stability with SACs,^77,444–446^ but full throughput studies with catalytic ability and stability are scarce on true SAA catalysts.^447^ Characterization of structure is also a hurdle for electrochemical systems, but critical to ensure correct identification and utilization of single atoms. The need for electrolyte and electrical connections makes traditional methods used in thermal catalysis for *in situ* and *operando* studies, such as XAS or STEM, more difficult. Current techniques such as in situ XAS can be beneficial when confirming single atoms during a reaction; however, XAS primarily provides information about the local environment of the predominant species in a sample while being “blind” to subpopulations.^228^ Thus, the identification of clusters in a sample that is primarily single atom sites is limited using techniques like XAS.

While significant progress has been achieved for SAAs in reductive electrocatalytic reactions, advances in oxidative electrolysis remain comparatively limited. Reducing conditions are fundamentally more conducive to structural stability and therefore have comprised a larger proportion of the literature. Oxidative environments introduce more prominent structural stability challenges, including corrosion and reconstruction, which not only challenge application spaces but also mechanistic work attempting to characterize active sites under reaction conditions. Still, SAAs have been tailored to improve poisoning tolerance and stability during oxidations, with performance gains often attributed to ensemble disruption and electronic effects. The dominant factors frequently vary across systems, and systematic understanding remains incomplete for both oxidative and reductive reactions.

Establishing the reaction mechanism is further critical for design in SAA electrocatalysis, but is challenging to achieve. Studies often include quantum calculations to suggest the method of modification of the reaction mechanism, but these have seldom been supported with experimental kinetic data. This disconnect is exacerbated by the inherent difficulties in modeling electrochemical systems, as solvent and electrochemical potential can be very challenging to accurately capture and are frequently neglected. Calculations have been corroborated with IR in certain cases, but there are limitations when it comes to differentiating spectator species and intermediates without sufficient control experiments. In cases of kinetic studies to support the computational thrust, kinetic data for parameters, such as transfer coefficient and reaction order, is taken over too limited of a range to fully diagnose. Overall, computational studies provide a strong tool for predicting and rationalizing, and qualitative trends can be meaningful, yet significant approximations are nearly always used that must be considered when comparing to experiment.

Future progress in this field will benefit from continued development of *operando* techniques capable of identifying true active sites and tracking their evolution under realistic electrochemical conditions. Close collaboration between computational and experimental researchers will be crucial to integrate quantum chemical screening with synthetic feasibility and *in situ* validation. Studies that convincingly combine *in situ* spectroscopy, kinetics, and theory remain sparse, and distinguishing bifunctional effects from electronic modulation remains one of the central challenges in understanding oxidation chemistry on SAAs. Expanding SAA research beyond small-molecule reactions toward more complex transformations, including electrochemical biomass upgrading and other multielectron organic conversions, represents an exciting frontier. With improved understanding of stability, ensemble effects, and reaction mechanisms, single-atom alloys could provide a method for atom-efficient, durable, and tunable electrocatalysts. Table 6 summarizes representative SAA systems reported in this section, together with the experimental and characterization techniques used to verify atomic isolation, the corresponding reaction type or elementary step, key catalytic performance metrics, the proposed reaction mechanism, and the relevant references.

**Table 6:** Summary of Representative SAA Catalytic Systems, The Techniques Used to Verify Atomic Isolation, The Associated Reaction Type or Elementary Step, Key Catalytic Performance Metrics, Proposed Reaction Mechanisms, and Corresponding References

**SAA system**	**Technique used to show evidence for atomic isolation**	**Reaction type**	**Key catalytic metric(s)**	**Stability test/observation**	**Catalytic performance**	**Proposed mechanism**	**Ref. #**
**Pt-group/Ag or Au**	N/A - computational DFT screening	ORR	4e^–^ pathway selectivity	N/A	ORR overpotentials of 0.46 V (PtAu) and 0.47 V (PdAu), vs 0.48 V for Pt(111). PdAu was predicted to begin CO poisoning at pCO ∼ 10^–9^ atm, ∼ 10 orders of magnitude above Pt(111).	Pt electronically modifies Au, stronger O_2_ binding in hollow/bridge site between host and dopant. Weakened CO binding on isolated Pt-group sites	^313^
**PtCo**	HAADF-STEM, XAS	ORR	4e^–^ pathway selectivity	30,000 potential-scanning cycles with minimal activity change and no morphological changes	Mass activity: 2.57 A/mg_Pt_ at 0.85 V, HO_2_^–^ selectivity <5% from 0.2–0.8 V	Charge transfer/delocalized charge tunes binding; OOH* bridges Pt and Co, promoting O–OH cleavage to water	^380^
**AuPd**	HAADF-STEM, EELS, XPS, XAS	ORR	Mass activity and power density	Minimal half wave potential loss after 120,000 cycles, negligible activity loss when methanol present	Mass activity: 5.37 A/mg_metal_ at 0.90 V vs RHE	Isolated Au electronically modifies Pd, shifting RDS from OH formation to H_2_O formation; adjacent Pd assumed active site.	^381^
**M1Ag54 clusters (Co, Ni, Fe, Ru, Rh, Pd, Pt, Au, Cu, Zn, In in Ag)**	N/A - DFT, MD, calculated Pourbaix diagrams	ORR	4e^–^ pathway selectivity	N/A	N/A	Operating active center proposed to be oxidized M_1_O_*x*_(OH), balancing O_2_ activation, OOH stabilization, and OH desorption	^365^
**FeCu**	STEM and XAS	ORR	Area-specific rate (ECSA) and selectivity to 4e^–^ pathway	Minimal deactivation of Fe active centers after 5000 cycles	Geometric current density: 12.8 mA/cm^2^ at 0.90 V vs RHE.	Charge asymmetry accelerates O_2_ adsorption and limits intermediate poisoning; Cu improves conductivity vs Fe–N_4_ SACs	^387^
**NiCo**	HAADF-STEM, XAS	2e^–^ ORR/electro-Fenton	H_2_O_2_ selectivity	Retained current after 1000 CV cycles, 70% activity after 5 reuse cycles.	H_2_O_2_ selectivity was >70% with an electron-transfer number of approximately 2.0 between 0.22 and 0.40 V vs Ag/AgCl.	Ni becomes more electron rich making Co the preferred O_2_ active site.	^389^
**BiPd**	HAADF-STEM, EDS, XPS, XAS	CO_2_RR	FE/selectivity to CO	20 h stability in flow Cell	CO FE: 91.8% at −0.31 V vs RHE.	Bi donates electron density to Pd, weakens H adsorption, suppressing HER	^398^
**PdAu**	XAS and STEM	CO_2_RR	FE to CO	24 h electrolysis with less than 15% loss	Maximum CO FE ∼ 80% at −0.50 V vs RHE; formate FE 9.8%.	Isolated Pd weakens CO binding and stabilizes *COOH, lowering CO_2_ activation overpotential	^390^
**CoSn**	HAADF-STEM, XRD, XPS, XAS	CO_2_RR	FE to formate	High FE to formate for 130h electrolysis	Formate FE: 99% at −0.89 V. FE to formate maintained above 92% from −0.83 to −1.33 V.	Co electronically activates adjacent Sn, stabilizing *OCHO.	^401^
**AgCu**	HAADF-STEM, EDX, XRD, XPS, XAS	CO_2_RR	Selectivity to C2 products	Flow electrolysis in 1 M KOH, retained selectivity	C_2_-product FE 65.1% at – 1.22 V; ethanol/ethylene ratio 4.26.* C_2_-product FE 78% at −0.65 V.**	Ag dissociates water which facilitates CO hydrogenation and asymmetric CO–CHO coupling*. Ag forms CO that diffuses to the Cu to promote C–C coupling**	^394^,^329^
**Cu_1_Ag_99_**	STEM, EDS, XPS	CO_2_RR	CO selectivity and HER suppression	Cu migration to surface under applied potential despite sustained activity/selectivity	FE increase from 48%-97.5% during 3 h electrolysis at −0.91 V. Partial current density to CO 0.72 μA/cm^2^ (ECSA normalized)	CO_2_ preferentially adsorbs to the rearranged Ag–Cu interfaces, charge transfer between Cu–Ag weakens CO binding	^366^
**PdCu**	HAADF-STEM, EDS, XAS	CO_2_RR	Suppression of HER, selectivity to CH_4_ and C_2_H_4_	FE over 50% for 300 min	FE to C_2_H_4_-33% and to CH_4_-25% at −1.1 V	Isolated Pd suppresses HER and binds CO, which promotes C–C coupling with CHO on Cu	^404^
**Cu**_**99**_ **In**_**1**_ **and Cu**_**1**_ **In**_**99**_	HAADF-STEM, EDS,XAS, XPS	CO_2_RR	Cu_99_In_1_ FE to CO; Cu_1_In_99_:FE to formate	12 hour continuous electrolysis retained high FEs	Cu_99_In_1_: CO FE 91% at −0.70 V. Cu_1_In_99_: formate FE 91% at −1.20 V	In dopants lower CO binding energy on Cu, In reduces surface H_2_O coverage, suppressing HER; Cu dopants in In favor *HCOO intermediates and binds *CO weakly, promoting formate	^405^
**AlCu**	N/A- computational	CO_2_RR	Predicted CO_2_RR energy requirements vs Cu; Selectivity to C_1_* and C_2_** products	Thermodynamic preference for isolated surface Al. * structural stability based on charge transfer and similar effective Al/Cu atomic radii.**	0.53 V for *CO → *CHO. Decreased activation barrier from 0.94 to 0.85 eV. * Predicted barriers of 0.42 eV for CO_2_ dissociation, 0.86 eV for CO dimerization, and 0.39 eV for the ethanol-forming hydrogenation step.**	Al stabilizes *CHO but repels *CO and weakens *H adsorption, favoring C_1_ hydrogenation while suppressing C–C coupling and HER.* Oxophilic Al activates CO_2_, retains CO and assists CO–CO coupling.**	^406^,^407^
**AlZnCu**	HAADF-STEM, EDX, XPS, XAS	CO_2_RR	C_2_+ FE at ampere-level current density; C_2_+/C_1_ ratio and energy efficiency	Retained activity and C_2+_ selectivity for >100 h at −1.5 V vs RHE.	Maximum C_2_+ FE = 91.4% at −1.66 A/cm^2^ with a C_2_+ partial current density of – 1.5 A/cm^2^.	AlCu sites activate CO_2_ and increase/stabilize *CO coverage, while ZnCu sites dissociate water and supply transferable *H. Lowers the *CO→ *CHO barrier and promotes asymmetric *CO-*CHO coupling on adjacent Cu sites.	^176^
**CoCu**	STEM,EDS,XPS,XAS	CO_2_RR	FE and current densities to CH_4_	10 h pulsed electrolysis retained high selectivity	CH_4_ FE: 62.3% at a current density of 600 mA/cm^2^(ECSA)	Co changes *CO from linear to bridge-bound, suppressing C–C coupling; Co enhances water dissociation and direct methanation	^409^
**SnCu**	HAADF-STEM, EDX,XAS, XPS, XRD.	CO_2_RR	Potential-switchable CO/C_2_H_4_ selectivity	Stable CO current and FE for 17 h at −0.70 V vs RHE. C_2_H_4_/CO/H_2_ FE ratio remained stable during 245 h in an MEA.	CO FE: 87.5% at −0.70 V vs RHE. C_2_H_4_FE: 48.3% at −1.90 V vs RHE, with 257 mA/cm^2^	Sn_1_–O–Cu sites lower the CO_2_→ *COOH barrier and favors CO. At more negative potentials, metallic Sn_1_Cu sites modify *CO adsorption and lower the *CO–*CO coupling barrier , promoting C_2_H_4_.	^410^
**WCu(111)**	N/A - computational screening of 25 transition metals	CO_2_RR/N_2_RR	Suppressing HER while favoring C–C and C–N coupling	N/A: Showing strong thermodynamic stability	Predicted barrier of 0.85 eV for *CO → *COH.	W modifies Cu to favor linear *CO and *CCO formation while also enhancing N_2_ adsorption/protonation	^411^
**PdCu**	HAADF-STEM, EDS, XAS	N_2_RR	Selectivity to NH_3_	Maintained current and selectivity during 30 h electrolysis	NH_3_ FE: 24.8% at −0.45 V vs RHE.	Cu electronically modifies isolated Pd, lowering *NNH hydrogenation barrier below HER	^416^
**PdCu**	AC-STEM, XAS	NORR	Selectivity to NH_3_	Industrial current densities at 200 h electrolysis	NH_3_ FE: 85%; current density: 210 mA/cm^2^(ECSA)	Electronic coupling between Pd–Cu lowers *NO hydrogenation barrier	^417^
**PdCu**	HAADF-STEM, XRD, XPS	NO_3_^–^RR	Nitrate removal/poisoning resistance	Strong sulfate poisoning resistance in flow cell	97.8% NO_3_–N removal after 180 min at an absolute cell current of 0.10 A (not normalized to geometric area or ECSA).	Cu reduces nitrate to nitrite, and Pd provides H* for hydrogenation	^418^
**PdCu**	HAADF-STEM, XAS, EDS	NO_3_^–^RR	Selectivity to NH_3_	Maintained high FE for 10 h	NH_3_ FE: 94.9% at –0.60 V	Dual-site pathway: Cu reduces nitrate to nitrite; Pd single atoms convert nitrite to NH_3_ via H* donation	^419^
**CoZn**	HAADF-STEM, XAS, XRD	NO_3_^–^RR	FE to NH_3_	Maintained NH_3_ FE and yield over 10 cycles. Stable for 24h at Amp current densities.	FE to NH_3_:98.7% at −0.8 V with current density of 2.4 A/cm^2^(geometric)	Isolated Co atoms dissociate water at lower barriers allowing *H to spillover to *NO_3_ on Zn for hydrogenation.	^420^
**NiCu**	HRTEM, XRD, HAADF-STEM**, XAS**	NO_3_^–^RR	FE/selectivity to NH_3_	Repeated electrolysis, high FE.* 30 h flow electrolysis with stable activity and morphology.**	Nearly 100% NH_3_ FE and 326 μmol/h*cm^2^ (geometric) at −0.55 V vs RHE.* ∼ 95% NH_3_ FE at −0.60 V vs RHE.**	Ni electronically modifies Cu to stabilize NOOH* and suppresses side pathways.* Ni promotes *H formation, lowering barrier for NOx hydrogenation**	^421^,^422^
**AuCu**	HAADF-STEM, XAS,	NO_3_^–^RR	FE/selectivity to NH_3_	High FE to NH_3_ over repeated cycles	NH_3_ FE: 98.7% at −0.20 V vs RHE.	Au and Cu vacancies electronically modify Cu; Au strengthens H* binding, favoring NO_3_^–^RR over HER.	^423^
**AuCu**	HAADF-STEM, XAS	NO_3_^–^RR	FE/selectivity to NH_3_	Minimal FE loss over 20 cycles, no loss in current density at −0.5 V for 12 h.	FE of 85.5% at −0.5 V with production rate of 8.47 mol/h*g_metal_ (normalized to total Cu + Au metal)	Au strengthens *NO_3_ binding on Cu through electronic effects. Weakens *N and promotes hydrogenation	^424^
**AuCu**	HAADF-STEM, XAS, XPS, EDS	NO_3_^–^RR	FE/selectivity to NH_3_	High FE over prolonged electrolysis	NH_3_ FE ∼ 99.7% at −0.80 V vs RHE.* > 90% NH_3_ FE across a broad potential window and stable operation at 1 A/cm^2^(ECSA).**	Au sites adsorb/activate nitrate, Cu host facilitates hydrogenation.* Au lowers NO_2_ hydrogenation barrier, making consumption of H faster than HER.**	^425^,^426^
**RuCu**	HAADF-STEM, XAS, XPS, Atomic Probe Tomography	NO_3_^–^RR	FE/selectivity to NH_3_	High FE over 100 hour flow electrolysis	NH_3_FE: 93% at 1 A/cm^2^(ECSA); nitrate conversion >99%	Isolated Ru activates nitrate and favors ammonia due to Cu suppressing HER	^427^
**PtRu**	HAADF-STEM, EDX, XAS	MOR	Current density	Maintained current densities and structure. Resist CO poisoning.	Total FE 87% at 0.6 V, 1.58 A/mg_Pt_ at 0.70 V vs RHE.* Mass-specific current density 1.33 A/mg_Pt+Ru_ at 0.84 V vs RHE.**	Pt strongly binds methanol, Ru donates OH. Isolated Pt lowers CO binding strength.	^367^,^372^
**RuPt**	HAADF-STEM, XAS, XPS	MOR/HOR	Current density	Stable over 1000 cycles.	MOR specific activity 2.805 mA/μg_PtRu,_	OH binding energy on Ru increased, lowering the CO–OH coupling step barrier.	^433^
**HEASA-Pt**	HAADF-STEM, XAS, XPS,XRD	MOR/DMFC	Mass activity	Retained current densities and structure over repeated CA’s	DMFC peak power density: 22.8 mW/cm^2^(geometric)	Isolated Pt weakens CO binding; Stabilizes COOH and OCHO due to oxophilic neighbors. OH donation from neighbors.	^432^
**PtPd**	HAADF- STEM, EDX, XAS	EOR	Current density and selectivity to acetate, low CO poisoning	Retained initial current density after 12 h at 0.93 V. Maintained high mass activity after 5000 CV’s	34.73 A/mg_Pt+Pd_ at 0.93 V. Acetate FE = 83.2%, acetate selectivity = 83.3%, and C_1_-product FE ∼ 14.5% at 0.8 V	Pt electronically modifies the strained Pd surface and strengthens ethanol adsorption at the Pd–Pt interface, lowering the *CH_2_CO, C–C cleavage barrier.	^440^
**BiPd**	HAADF-STEM, XAS	EOR	Current density	Maintained high mass activity over 1 h CA	Acetate/ethanol conversion ratio: 0.97 for BiPd	Bi electronically modifies Pd to favor C_2_ oxidation/acetate formation.	^441^
PtPd*/AuPd**	HAADF-STEM, XAS, XPS	FAOR	Current density	Current peak remained after cycling. Reduced but not fully suppressed CO poisoning	5.2 A/mg_metal_ at ∼ 0.85 V.*	Pt*/Au** locally modifies Pd electronically, increasing direct FAOR pathway and reducing CO poisoning	^442^,^443^
**IrRu**	HAADF-STEM, EDS, XAS	OER	Current density	Small potential loss after 50,000 LSV cycles at 300 mA/cm^2^	At 1.53 V vs RHE: 1.97 A/mg_Ir+Ru_	Ir stabilizes Ru–O without deep oxidation which stabilizes HOO*.	^368^

## 5 Plasmonic Catalysis

### 5.1 Fundamentals and Evolution of Plasmonic Catalysis

Plasmonic catalysis utilizes the intense light-harvesting capability of coinage metal hosts (Ag, Cu, Au) and Al to capture visible light and bypass traditional ground-state thermal scaling relationships.^53,448–453^ In weakly interacting systems, these plasmons primarily decay via nonradiative Landau damping, generating energetic, nonequilibrium electron–hole pairs within the metal lattice on an ultrafast (<10 fs) time scale.^454–457^ However, at the strongly hybridized interfaces characteristic of dilute alloys, chemical interface damping (CID) can dominate. This interfacial pathway bypasses bulk transport losses by driving direct, momentum-conserving hot-carrier injection directly into the molecular orbitals of chemisorbed species. The subsequent time scale of hot-carrier relaxation (100 fs- several ps) overlaps with that of bond activation at adsorbate–metal interfaces, enabling plasmon-driven chemical transformations.^457^ The optical absorption efficiency is governed by the material’s underlying dielectric properties, which is optimized when dissipative losses are minimized in the visible spectrum.^458^

Foundational ultrahigh vacuum (UHV) surface science on extended single crystals^459–464^ established that these nonthermal carriers can inject into adsorbate molecular orbitals to form a transient negative ion (TNI). This drives chemical transformations through Desorption Induced by Electronic Transitions (DIET) or,^465^ at high photon fluxes via multibody vibrational “ladder-climbing,”^463^ Desorption Induced by Multiple Electronic Transitions (DIMET). State-resolved and pulse-correlation studies (e.g., NO/Pt(111),^466^ CO oxidation/Ru(0001),^467^ CO/Au(111)^468^) provided further evidence that these electron-mediated reaction pathways operate independently of bulk phonon-driven lattice heating. More detailed discussion of photochemical dynamics at extended surfaces can be found elsewhere.^469^

Translating these nonthermal frameworks to nanostructured monometallic particles demonstrated that localized surface plasmon resonance (LSPR) channels can drastically enhance reactivity and manipulate selectivity.^470,471^ Early nanoscale milestones suggest that plasmonic hot carriers lower the rate-limiting activation barriers for diatomic bonds, enabling low-temperature O–O dissociation for ethylene epoxidation over Ag^470,471^ and room-temperature H–H activation via Feshbach resonances on Au.^472^ Furthermore, light intensity can act as a direct kinetic knob to change reaction pathways; dynamic hot-carrier injection has been shown to tune selectivity by partially reducing self-poisoning surface oxide shells on Cu to increase propylene oxide selectivity,^473^ or by creating high local densities of transient intermediates (such as CO_2_^•‑^) on Au to force a shift from CH_4_ generation to multielectron C–C coupling.^474^ Despite these light-driven advantages, unalloyed plasmonic coinage hosts remain intrinsically less reactive toward many critical industrial feedstocks. This fundamental limitation highlights the need to transition to dilute plasmonic alloys (DPAs) and SAAs, which pair these efficient light-harvesting hosts with highly dispersed transition-metal catalytic engines (including isolated atoms and small ensmebles).

Given the nascent state of plasmonic catalysis in dilute bimetallic systems and the inherent challenges in their structural characterization, this section divides dilute alloys into three classes: (i) alloys with reactive metal ensembles, (ii) alloys containing both single atom and ensemble sites, and (iii) alloys dominated by single atom sites. The first two are referred to as dilute alloys (DAs), while the third will be explicitly defined as SAAs as done previously throughout this review. As in the rest of the review, we will use the notation XY where X is the minority and Y is the majority component. Note that in some figures this notation is reversed because plasmonic alloys are often referred to as “antenna-reactors” in which the majority metal is the “antenna” and the minority is the “reactor”. This section will focus exclusively on bimetallic systems, excluding SACs in which isolated metal atoms are directly supported on semiconductors or oxides.^475,476^

### 5.2 The Concept of Antenna-Reactor and Advantages of Plasmon- or Light-Assisted Processes

The incorporation of reactive transition metals (such as Pd, Pt, Rh, Ru) into light-harvesting antennas allows for synergy between photon absorption and chemical catalysis. This *antenna-reactor* concept was first demonstrated in Pd–Al heterostructures,^449^ where Halas and co-workers utilized LSPR to drive selective acetylene hydrogenation (Figure 46). By inducing hot-carrier-mediated H_2_ dissociation-the rate-limiting step-light illumination steered selectivity toward ethylene by modulating surface hydrogen coverage. This sets the stage for the alloy antenna-reactors discussed below, including SAAs.

**Figure 46:**
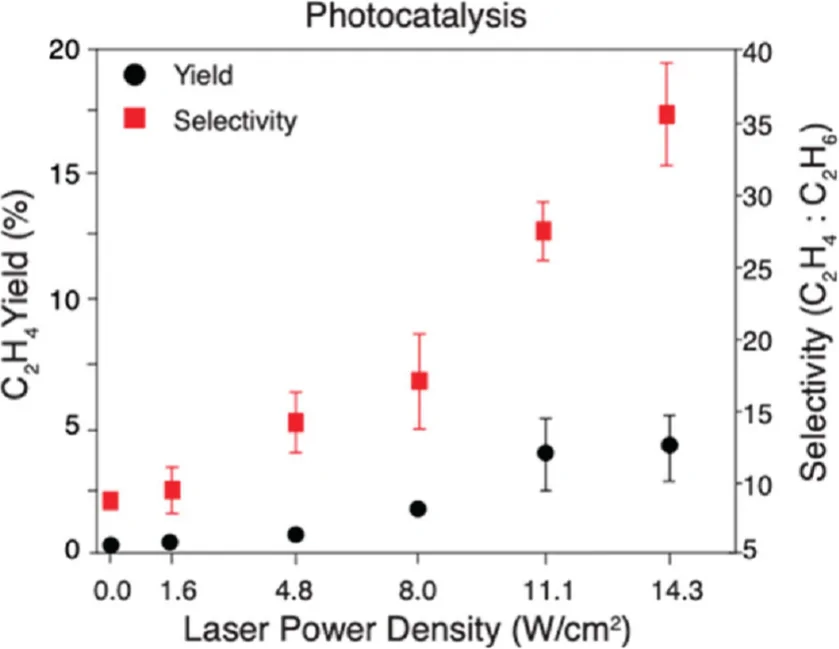
Model catalysts validating the concept of the antenna-reactor: Ethylene (C_2_H_4_) yield from acetylene semihydrogenation over Pd–Al as a function of light intensity. Adapted with permission from ref.^477^ Copyright 2021 American Chemical Society.

#### 5.2.1 Energy Flow and Near Field Localization

The efficiency of these bimetallic systems depends on the precision of energy flow from the plasmonic core to the reactive shell. Linic and co-workers elucidated this by comparing Ag and Ag–Pt core–shell structures, finding that even a thin Pt layer significantly shifts the plasmon decay pathway from scattering to absorption.^450^ This concentrates electromagnetic energy at the reactive Pt surface, as evidenced by light-enhanced CO oxidation rates that mirror the Ag–Pt extinction spectra. Multiple key parameters are required to control this energy transfer. For example, smaller nanoparticle cores generally maximize near-field intensity at the interface, although performance must be balanced against increased surface damping at the sub-10 nm scale.^478–480^ A high ratio of the imaginary dielectric functions of the two components (ε_2, shell_/ε_2, core_) ensures preferential energy dissipation into the reactive component.^481^ Finally, the geometry influences performance, as utilizing nanorods or super crystals can be used to tune optical coupling and minimize Ohmic losses.^482–484^

#### 5.2.2 Fundamentals and Advantages of Plasmon-Assisted Process

In this section, we discuss the unique features of plasmon-assisted bond reshaping and outline design principles for localizing charge at isolated atoms or small clusters embedded within a plasmonic host, as this is a critical process for DAs and SAAs.

##### 5.2.2.1 Vibrational Excited States and Transient Negative Ion

While IR lasers can directly populate vibrationally excited states,^468,485^ plasmonic catalysis achieves this indirectly *via* electronic excitation. Specifically, transient hot-electron transfer from the metal into an adsorbate’s antibonding orbital forms a transient negative ion (TNI).^486^ Upon the electron’s return to the metal, the molecule is left in a vibrationally excited state on the electronic ground-state potential energy surface. For simplicity, only electronically excited states are presented in this figure (Figure 47).

**Figure 47:**
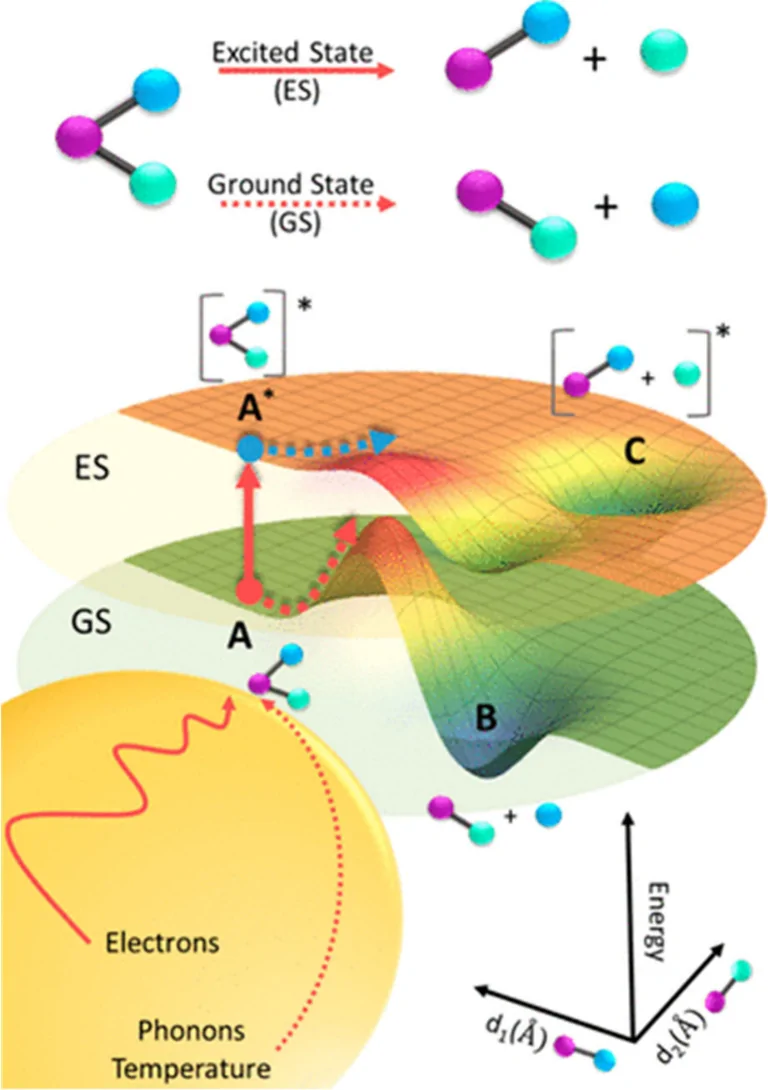
Schematic illustration of excited states: the potential energy surface for electronic ground- and excited-states in dissociation of triatomic molecules, with the latter having a vastly different energy landscape. Reproduced with permission from ref.^487^ Copyright 2019 American Chemical Society.

This TNI-mediated vibrational pumping has been experimentally validated through KIE measurements. For instance, in O–O dissociation on Ag and H_2_O-assisted methanol reforming over ZnCu alloys, researchers observed KIE values were higher than those predicted by thermal models.^470,488^ Taking O–O dissociation as an example, the 10 fs electronic lifetime of the TNI is insufficient for direct dissociation; however, the residual vibrational energy deposited upon relaxation accelerates ^16^O more effectively than ^18^O. This energy gap between isotopologues is further magnified under DIMET conditions, where high photon fluxes drive the system higher up the vibrational ladder before relaxation can occur, providing a distinct diagnostic signature of nonthermal pathways.^471^

##### 5.2.2.2 Leveraging Light-Induced Desorption and Surface Diffusion to Mitigate Surface Poisoning and Improve Stability

Another advantage of bimetallic plasmonic catalysis is the ability to regulate surface species through light-induced desorption, first demonstrated on Pd–Al model catalysts for the selective semihydrogenation of acetylene.^449^ Using DRM over RuCu as an example,^75^ Periodic DFT+D3 and embedded multiconfigurational calculations suggest that light illumination promotes C–H activation, with a larger effect on RuCu than Cu. Furthermore, H–H recombination and desorption are favored under illumination, which lowers the probability of H–O recombination that would otherwise form H_2_O and decrease the selectivity (Figure 48a). C, H and O exhibit low diffusion barriers on Cu and can therefore diffuse freely across Cu surfaces. In contrast, a ∼ 2 eV diffusion barrier is present at Ru sites, which act as anchoring points that inhibit C migration and subsequent C–C coupling, thereby suppressing surface carbon accumulation and coking (Figure 48 **b,c**). These results highlight the significance of integrating experimental and theoretical insights to obtain atomic-scale understanding of surface processes and to guide the design of future plasmonic catalysts.

**Figure 48:**
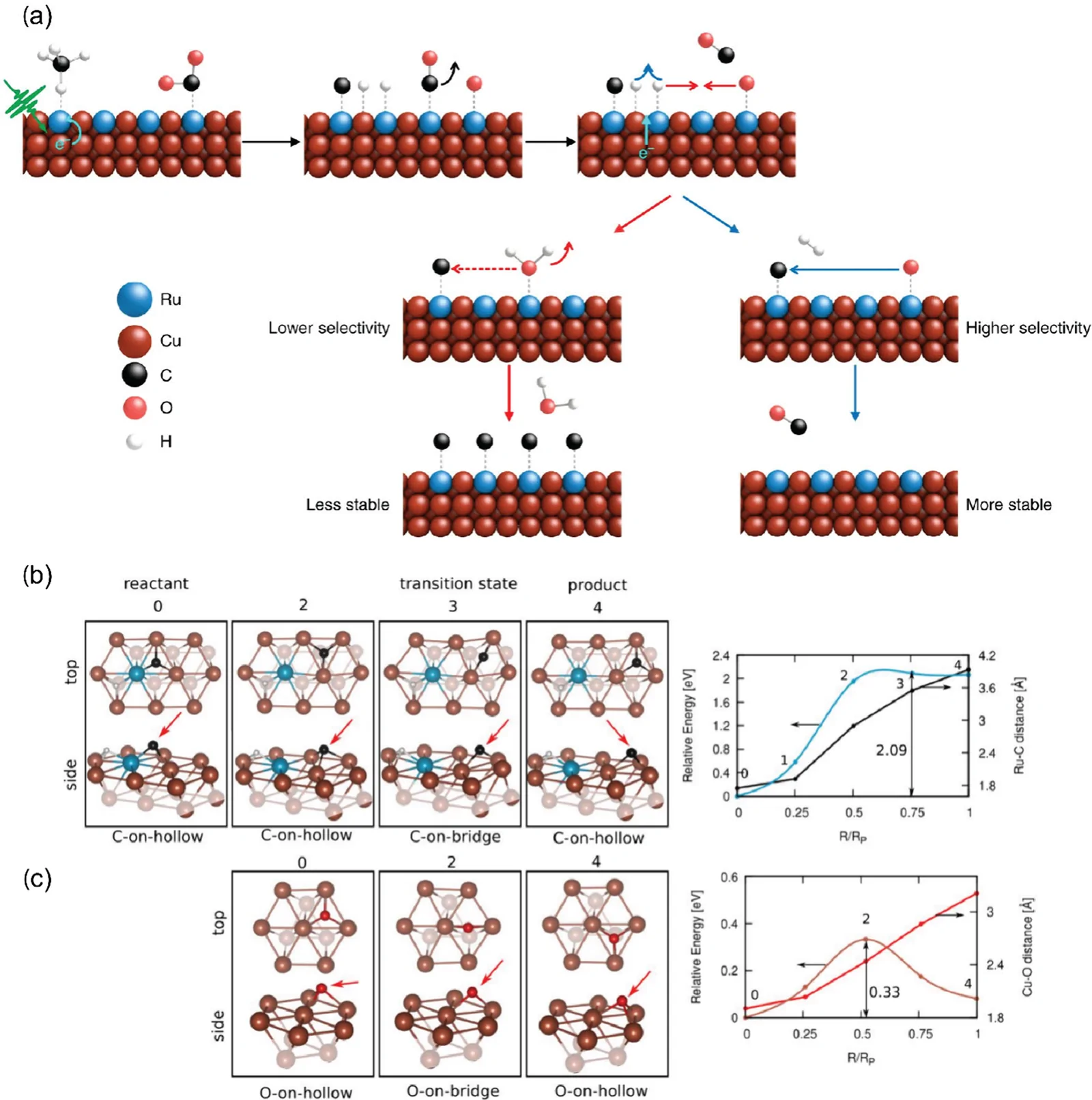
a) Demonstrating the DIET mechanism in enhancing the selectivity and stability of RuCu for methane dry reforming. b) C diffusion pathway and diffusion barriers from Ru sites on RuCu(111). c) O diffusion pathway and diffusion barriers from Cu sites on Cu(111). Adapted from ref.^75^ Copyright 2020 Springer Nature.

The photochemical processes can be further investigated and contrasted with *ex-situ* or *in situ* vibrational spectroscopy such as Raman^489,490^ and infrared spectroscopy.^491–493^ Recently, Halas and co-workers reported a light-assisted approach for regenerating thermally deactivated catalysts during the steam methane reforming reaction over RhCu dilute alloys. Combined XPS and Raman spectroscopy indicated the retention of partially oxidized Rh sites and a significant reduction in surface carbon coverage, providing indirect evidence that DIET facilitates the removal of surface coke and thereby maintains the stability of these dilute alloys in carbon-related chemical transformations. This approach was further applied to study N_2_O decomposition,^490^ where oxide accumulation is a key deactivation factor. *Ex-situ* Raman mapping suggests that light enhances O–O recombination and desorption, contributing to long-term photocatalytic performance that is absent under purely thermal conditions (Figure 49).

**Figure 49:**
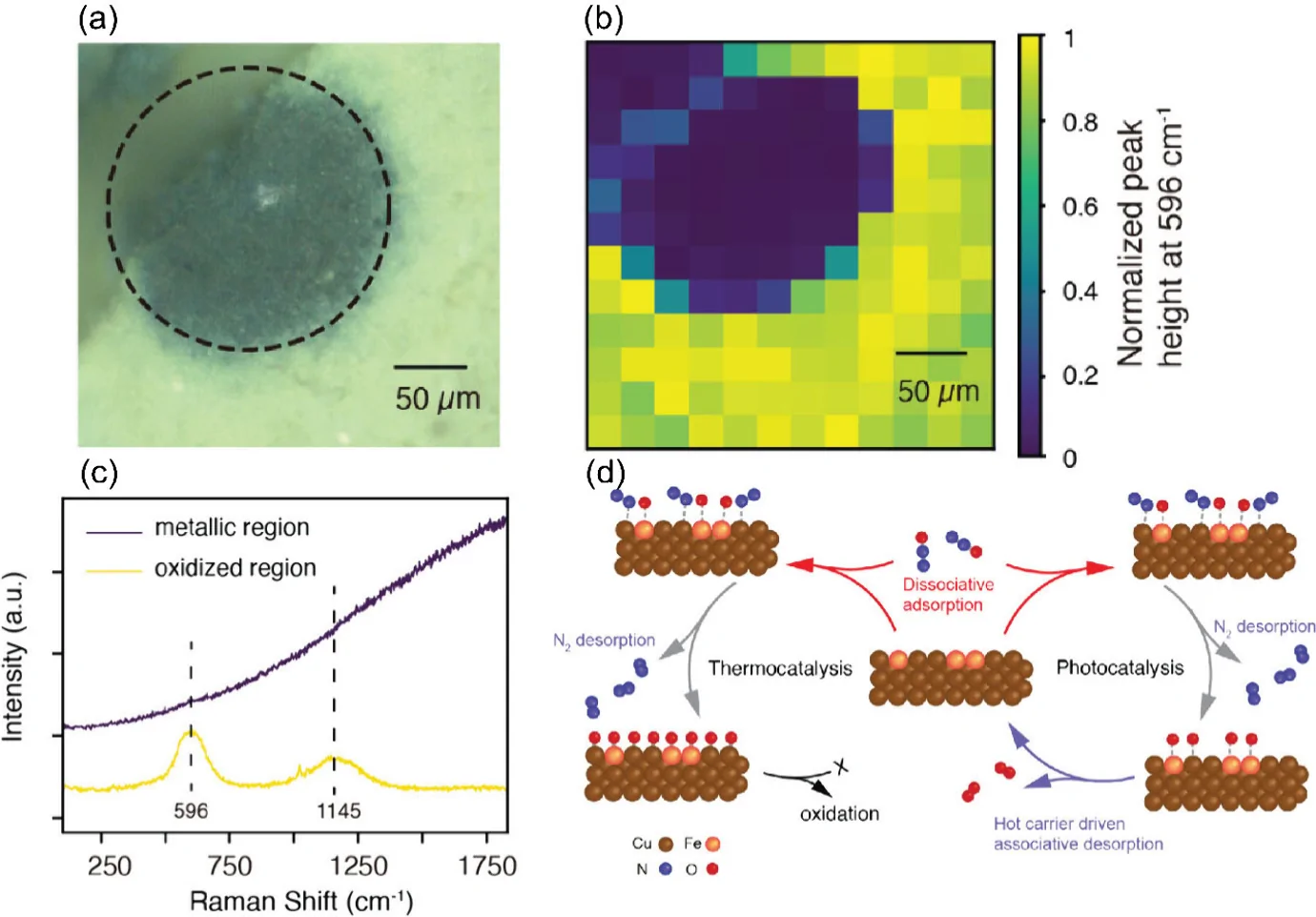
Further contrasting chemical transformations under light vs dark conditions using vibrational spectroscopy, such as Raman spectroscopy. a) Spent FeCu catalysts after the N_2_O decomposition reaction. b) Raman mapping monitoring oxide formation, with the characteristic peak at 596 cm^–1^. c) Typical Raman spectra of the metallic (circled region in part a) and oxide regions (exterior region in part a), as shown in (a). d) Proposed photo- and thermal-induced mechanisms for N_2_O decomposition over FeCu. Adapted with permission from ref.^490^ Copyright 2025 American Chemical Society.

##### 5.2.2.3 Decoding Site-Specific Desorption using Monometallic Catalysts as Models

To understand how these macroscopic effects arise at specific atomic motifs, it is essential to first examine elementary steps on monometallic surfaces and then extend these insights to more complex bimetallic surfaces. Christopher and co-workers provided a definitive contrast in light-induced CO desorption between alumina supported Pt and Pd.^491^ Using DRIFTS to quantify desorption rates, they demonstrated that Pt exhibits a significantly higher photoresponse than Pd (Figure 50). This disparity is explained by the PDOS: the narrower unoccupied states in Pt-CO lead to longer excited-state lifetimes and more pronounced C–O bond elongation compared to the rapidly quenched excitations on Pd. Furthermore, energy dissipation is governed by the adsorbate configuration. On Pd, frustrated rotational modes coupled with lateral CO–CO interactions dissipate excited-state energy before desorption can occur. In contrast, the predominantly linear CO binding on Pt favors perpendicular vibrational motion upon excitation, which more effectively drives the molecule over the desorption barrier.

**Figure 50:**
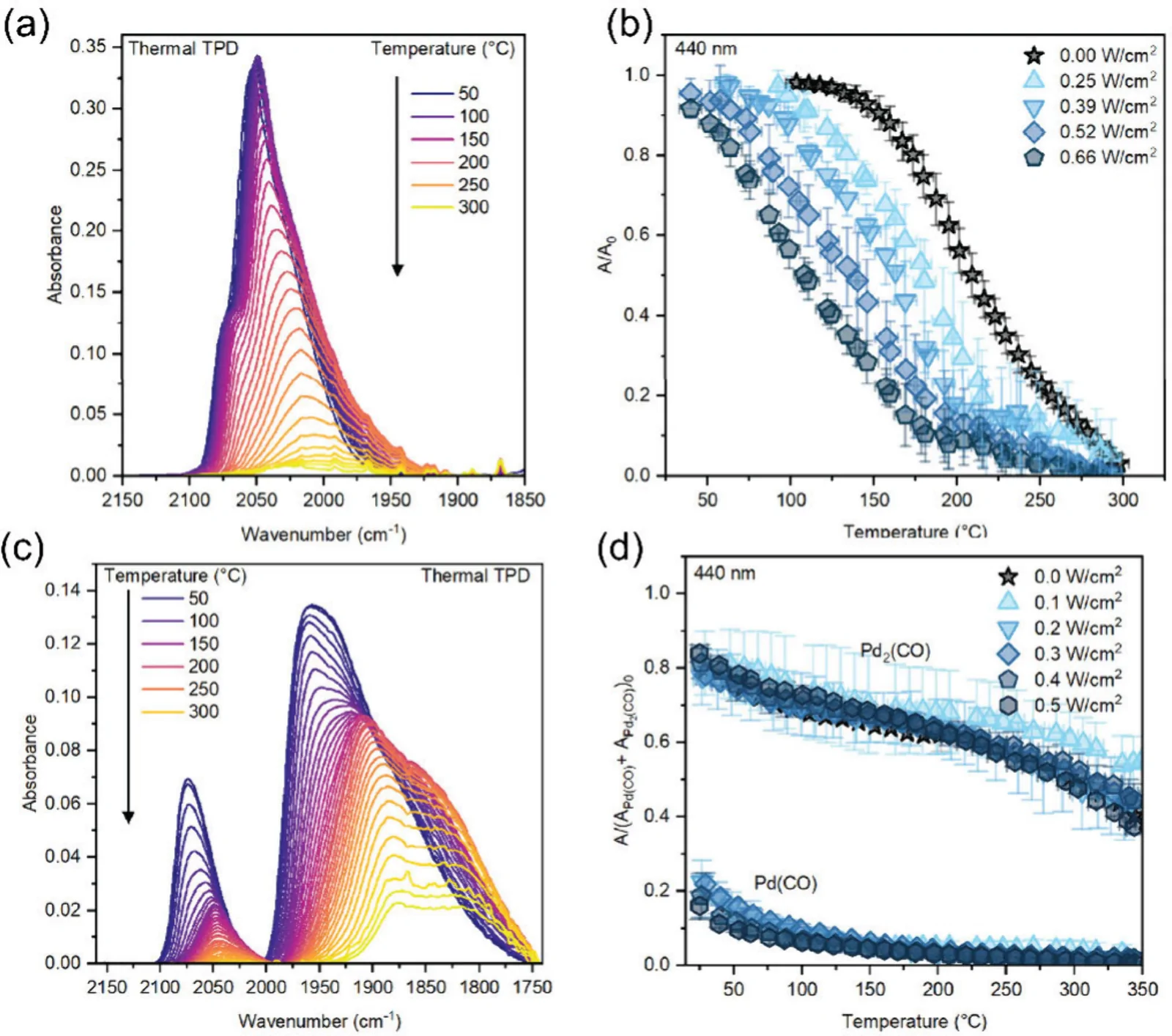
Direct comparison of light-induced CO desorption behavior on 1 wt % Pt/Al_2_O_3_ and Pd/Al_2_O_3_ catalysts. a) DRIFTS monitoring of C–O stretching modes on Pt/Al_2_O_3_ as a function of temperature. b) Quantified CO peak area on Pt as a function of temperature under different light power intensities at a fixed wavelength. c) C–O stretching modes on Pd/Al_2_O_3_ as a function of temperature. d) Quantified CO peak area on Pd as a function of temperature under different light power intensities at a fixed wavelength. Adapted with permission from ref.^491^ Copyright 2024 American Chemical Society.

##### 5.2.2.4 Toward a Temperature-Dependent Quantum Efficiency Model

The precision of these measurements has recently enabled the extraction of site-specific desorption rates, distinguishing between well-coordinated and undercoordinated surface atoms on Pt/Al_2_O_3_ model catalysts.^132^ While well-coordinated Pt sites show a strong correlation with light-induced desorption, highly undercoordinated sites do not. Conventional light-perturbed thermal models fail to capture these trends; instead, the data necessitate a model incorporating temperature-dependent apparent quantum efficiency (AQE).

In this framework, the AQE scales with temperature as the population of vibrationally excited ground states increases, effectively lowering the barrier for subsequent electronic excitation. This model provides a quantitative foundation for predicting how elementary steps-adsorption, diffusion, and desorption-behave under the synergistic thermal and optical environments characteristic of plasmonic reactors. In short, fundamental aspects-such as scrutinizing DIET through theoretical studies or *ex-situ* and *in situ* spectroscopy, and probing elementary steps including photoinduced CO adsorption and desorption, with potential extension to more complex molecules such as light alkanes using *operando* spectroscopy can provide critical insight into photodriven or photoassisted processes and offer guidelines for the future design of plasmonic catalysts.

##### 5.2.2.5 Probing Local Heating *via* Nanothermometry and Time-Resolved Measurements

Disentangling photothermal and photochemical contributions in plasmonic catalytic processes remains challenging.^494,495^ While infrared thermography and multithermocouple reactor designs are commonly used to estimate catalyst temperatures, these methods primarily capture macroscopic, ensemble-averaged temperatures. Moreover, infrared thermography relies on accurate emissivity values, which can vary under reaction conditions and complicate temperature determination. Consequently, they lack the spatial and temporal resolution required to resolve molecular-level plasmon generation and the subsequent nonequilibrium heat dissipation.^496^ The vastly different heat capacities and relaxation times of electrons and nuclei under illumination often preclude the definition of a single, uniform system temperature. In this context, *operando* nanothermometry has emerged as a critical tool for probing localized energy gradients and distinguishing nonthermal contributions from bulk heating.^497,498^

While Raman thermometry has long been established in the spectroscopy community,^499,500^ Linic and co-workers^497,501^ leveraged the Stokes to anti-Stokes ratio specifically to probe nonequilibrium vibrational temperatures in plasmonic catalytic systems. They utilized this approach to demonstrate that under illumination, adsorbates can reach vibrational populations far exceeding those predicted by the macroscopic lattice temperature, thereby validating the presence of nonthermal pathways. The anti-Stokes background intensity serves as a sensitive reporter of electronic relaxation; as photoexcited electrons in the metal relax toward the Fermi level, they populate vibrational manifolds that emit anti-Stokes photons. Enhanced anti-Stokes signals under 785 nm illumination (Figure 51) highlight the presence of hot spots in close-contact nanoparticles and suggest direct, momentum-conserving charge-transfer pathways at the molecule–surface interface.

**Figure 51:**
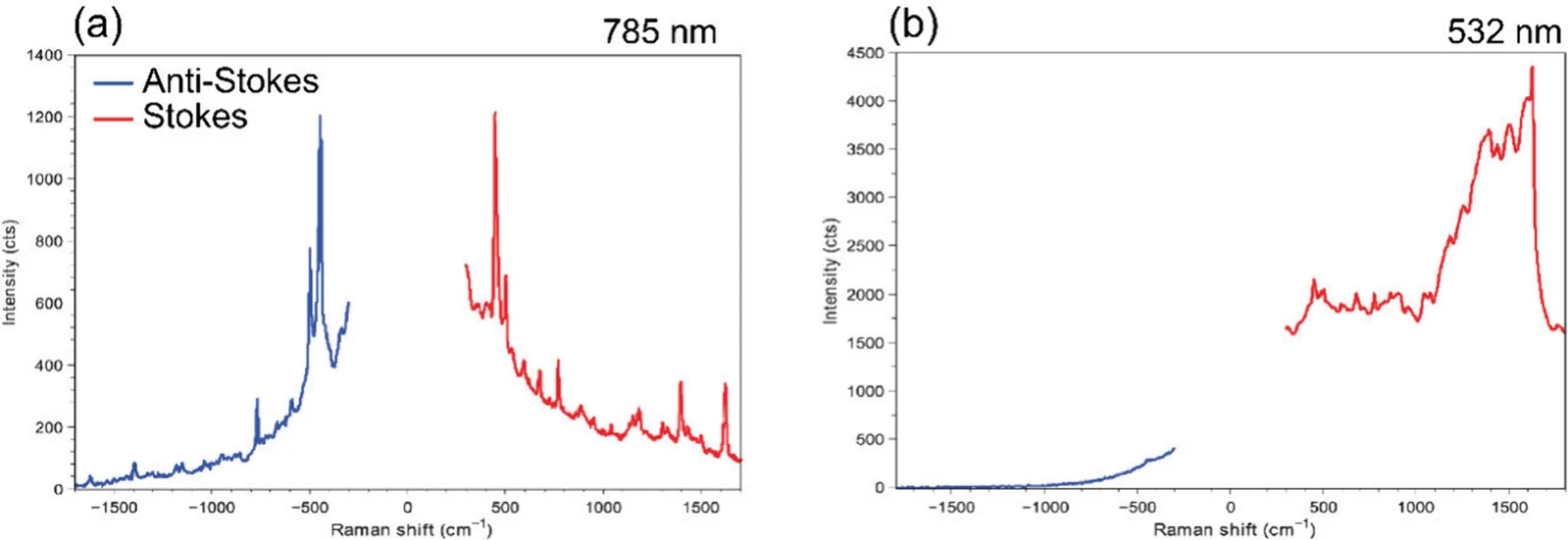
Raw Raman spectra showing the Stokes and anti-Stokes signals of Ag–methylene blue systems after excitation with a) 785 nm and b) 532 nm lasers, with the 785 nm laser producing a more pronounced anti-Stokes feature. Adapted with permission from ref.^501^ Copyright 2016 American Chemical Society.

While Raman thermometry offers high *in situ* sensitivity to vibrational energy, its application is often limited by signal-to-noise constraints in the gas phase. As a complementary alternative, X-ray absorption nanothermometry exploits the temperature-dependent disorder of local coordination environments. By quantifying the Debye–Waller factor (lattice disorder) *via* EXAFS, researchers can differentiate temperature fields between metal nanoparticles and their supports. This technique has been successfully applied to probe Ni nanoparticles during the RWGS and dry methane reforming reactions.^498^ However, this approach faces limitations as the surface-to-volume ratio approaches unity, such as in small clusters and single-atom catalysts, where nearly all atoms are exposed to the surface and can undergo dynamic structural reconstructions that complicate the separation of thermal and structural disorder. In addition, the reduced signal-to-noise ratio at elevated temperatures further challenges EXAFS analysis, particularly for highly dilute dopants approaching the single-atom limit. Specific to bimetallic plasmonic catalytic systems, the electron–phonon coupling during the plasmon decay process for bimetallic systems can be further captured by coupling X-ray techniques with pump–probe spectroscopy.^502^

Pump–probe spectroscopies can be further applied to investigate the efficiency of electron–phonon coupling, thereby providing insight into energy localization in heterogeneous systems.^139^ For example, in core–satellite Au–Pd structures, the decay time is significantly shortened within the Pd clusters, leading to a local temperature gradient exceeding ∼ 150 K between Pd and Au, with energy preferentially localized at the Pd sites.

Nanothermometry approaches leveraging anti-Stokes Raman spectroscopy, X-ray techniques, and ultrafast spectroscopies provide ideal platforms for probing vibrational temperatures in molecule-metal complexes, heat transport between dissimilar metals, and the local temperatures of active species. Combining scanning probe microscopy with atomically sharp tips and ultrafast spectroscopy represents a promising future direction for enabling real-time, spatially resolved monitoring of plasmonic processes.^496^ Details of the instrumentation used to characterize plasmon-assisted processes are described elsewhere.^503^

##### 5.2.2.6 Probing Surface Dynamics under Light Illumination

Active sites confined to the single atom limit may undergo continuous structural evolution under realistic reaction conditions, posing additional challenges for both spatial resolution and signal-to-noise ratio. Although underexplored in the plasmonic community, isolated dopant metal sites have been shown to significantly enhance the sintering resistance of coinage-metal host matrices in thermal catalysis. This stabilization behavior—initially observed during selective ethanol dehydrogenation over NiCu SAAs^95^ under thermal conditions—was subsequently confirmed by Christopher and co-workers for PtCu architectures.^98^ The introduction of pulsed laser illumination adds layers of complexity to these processes, driving transient local heating, nonequilibrium hot-carrier injection, and rapid, site-specific adsorbate desorption.^504^

To probe these variations under reaction conditions, Swearer and co-workers utilized a coupled GC and operando CO-DRIFTS methodology.^505^ By systematically altering the propane/Ar and propane/CO/Ar feed compositions at 200–500 °C, the retention of a flat linear CO-Pt stretching mode and the complete absence of bridge-bound CO confirmed that the isolated Pt sites are likely to remain atomically dispersed and unperturbed during active propane dehydrogenation. This thermal resilience is corroborated by EXAFS analysis by Cargnello and co-workers.^507^ Elevated temperatures can drive dopant incorporation into the host lattice, increasing dopant–host coordination, and promoting the formation of highly dispersed substitutional alloy structures. Such restructuring is governed by the interplay of local alloy energetics, electronic interactions between dopant and host atoms, and, at sufficiently high temperatures, by configurational entropy.

Illumination can actively drive these local coordinates; during CO_2_ hydrogenation over Ru_0.1_Cu/MgAl_2_O_4_ DAs, Ye and co-workers performed wavelet transform analysis of EXAFS spectra to monitor the Ru–Ru/Cu scattering pathways. Under light exposure, they observed a shortening of the local scattering distances compared to bulk Ru foils, pointing to the *in situ* structural reconstruction of the RuCu alloy phase. In a separate study, Swearer and co-workers evaluated the structural stability of RhCu SAAs via STEM-based particle size distributions, tracking a minor shift from 1.3 ± 0.3 nm (as-synthesized) to 1.7 ± 0.4 nm postillumination, compared to 1.3 ± 0.4 nm under dark thermal conditions. This negligible variation implies that no severe structural sintering occurs within the investigated temperature regime, identifying carbonaceous coking rather than atom migration as the primary deactivation pathway for light-enhanced propane dehydrogenation. Moving forward, systematically isolating these light-induced dynamics will require moving beyond conventional steady-state measurements toward advanced *operando* toolsets. Specifically, careful EXAFS analysis under illumination must account for catalyst thermalization, including Debye–Waller effects and thermal broadening, to distinguish light-induced structural changes from temperature-dependent effects. Wavelet-transform analysis can further aid in visualizing different scattering contributions in multicomponent alloys..^228,498^ Furthermore, operando TEM coupled with synchronized pulsed-laser illumination could track light-induced atomic and surface restructuring and its evolution toward a light-induced steady state.. By combining these complementary, time-resolved structural probes, the community can begin to establish real-time nonequilibrium phase diagrams that describe the structural evolution of operating plasmonic catalysts under illumination.

### 5.3 Transitioning to Dilute Plasmonic Alloys (DPAs)

The ability to resolve local energy gradients and vibrational pumping at the nanoscale provides the theoretical justification for the antenna-reactor design strategy. While monometallic surfaces allow for the scrutiny of elementary steps, bimetallic architectures-specifically those that decouple light harvesting from bond activation–offer a pathway to maximize catalytic efficiency. By concentrating the electromagnetic near-field at a reactive minority site, these systems aim to exploit the nonthermal hot-carrier pathways elucidated by nanothermometry, all while preserving the optical properties of the plasmonic host.

Building on the energy-flow principles observed in core–shell and heterometallic systems, the antenna-reactor concept naturally extends to DPAs. In these systems, a reactive transition metal is incorporated into a plasmonic host at low concentrations, typically below a 10% atomic ratio.^506^ The nature of active sites can be tuned *via* dopant loading and careful active site engineering. Considering the dynamic nature of metal dopants under reaction conditions^507,508^ and the relatively early stage of efforts to confine dopants to the single atom regime in plasmonic catalysis, we follow the definitions and classifications established in Section 5.1 throughout the following discussion on DPAs.

Before discussing chemical transformations, we briefly highlight recent advances in simulating chemical reactions at plasmonic interfaces. Because photoexcited states are present, ground-state quantum mechanical models, such as traditional DFT, do not generally provide accurate descriptions. However, more advanced methods such as embedded correlated wave function (ECW),^453,509^ real-time time-dependent density functional theory (RT-TDDFT),^510^ and real-time time-dependent density functional tight binding (RT-TDDFTB)^511^ represent three major approaches. ECW can give the excited-state potential energy surfaces for an elementary step, and is well suited for modeling excited-state reaction barriers. RT-TDDFT employs explicit time propagation of Kohn–Sham eigenstates to obtain the nonadiabatic electronic density at each step, enabling simulations in nonlinear regimes such as strong-field conditions.^512^ Thus, RT-TDDFT can capture bond activation dynamics under applied electric fields. RT-TDDFTB offers the computational efficiency needed to study larger clusters relevant to nanoparticle systems. These approaches are generally complementary, with different strengths and weaknesses and providing somewhat different types of insights.

To optimize hot-carrier deployment in plasmonic catalysis, nonradiative decay channels and the resulting carrier energy profiles must be precisely mapped.^513^ Quantum mechanical modeling across monometallic hosts (Au, Ag, Cu and Al) demonstrates that while direct interband transitions persist at all scales, geometry-assisted intraband pathways become competitive only below ∼ 40 nm. The energy distributions of these carriers are fundamentally constrained by the host electronic structure: the close-lying d-bands in Cu and Au facilitate strong interband (d → sp) visible transitions that yield highly asymmetric distributions (where hot holes carry the highest excess energies), whereas the deeper d-bands in Ag isolate visible excitations to symmetric, intraband distributions concentrated near the plasmon energy.^514,515^ Extending these bulk decay channels to the metal–adsorbate interface, RT-TDDFT simulations of CO on coinage hosts demonstrate that a discrete fraction (≈ 0.5–3%) of photoexcited electrons directly populates unoccupied molecular states, extending transient electron density up to ∼ 5 Å into the adsorbate prior to formal chemical bonding.^516^ For comprehensive theoretical derivations of these nonradiative bulk decay pathways and explicit real-time interfacial charge-transfer dynamics, readers are directed to the landmark studies by Atwater and co-workers and Erhart and co-workers.^514–516^

Erhart and co-workers extended these interfacial models to dilute PtAg plasmonic alloys,^517^ demonstrating that increasing Pt content enhances hot-carrier localization at the surface but reduces overall carrier generation, with an optimal balance achieved at 17% Pt. At this fixed concentration, comparing different alloying elements (Re, Ru, Rh, Pt, Pd, Cu, and Au) revealed that while hot electron distributions remain largely uniform, hot hole profiles are highly element-specific (Figure 52a,b). Under 3.3 eV excitation, Pt and Pd generate “hotter” holes (−2 to 0 eV range) that localize at the surface compared to Ru and Rh which generate them in the −1 to 0 eV range), establishing a clear guideline for bimetallic hot-hole engineering. Rossi et al. extended this concept to single-atom dopants embedded in Cu, Ag, and Au hosts, confirming that photoexcited hot holes strongly localize directly at the isolated single-atom sites, whereas the corresponding hot electrons remain delocalized across the plasmonic matrix.^518^

**Figure 52:**
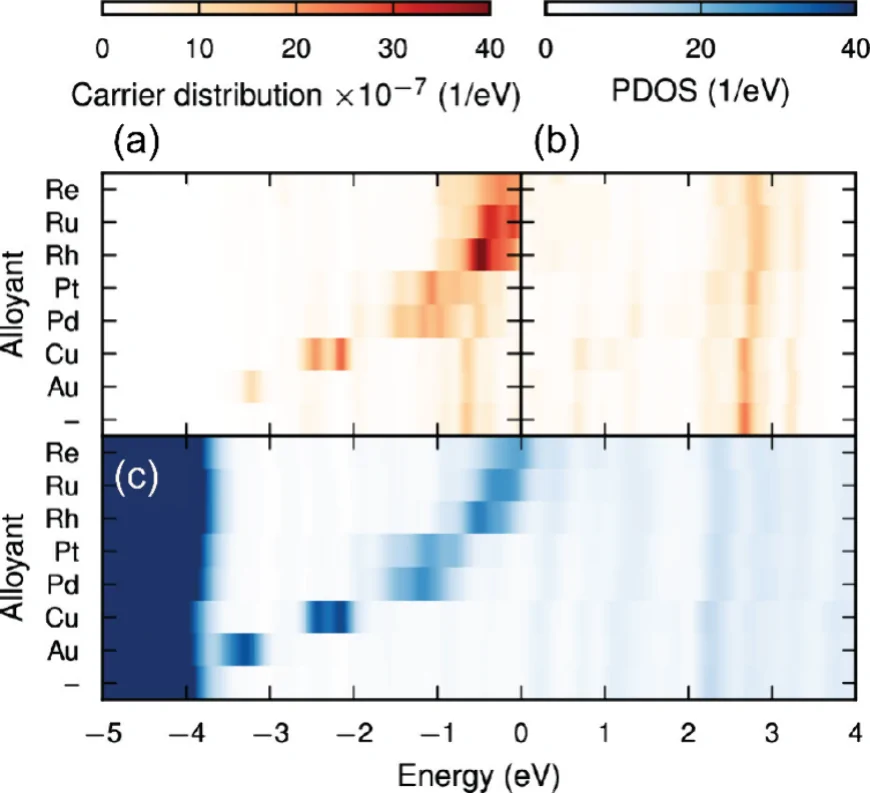
Hot-carrier distributions following 3.3 eV laser excitation on Ag-based DPAs with a dopant loading of 17%. (a) Hot-hole distribution, (b) hot-electron distribution, and (c) surface PDOS of the top layer. Reproduced from ref.^517^ Copyright 2024 The Authors. Licensed under CC BY 4.0.

Following the mapping of plasmon decay channels, evaluating nonequilibrium bond activation trends is critical for establishing predictive design principles for future dilute plasmonic alloys (DPAs). Carter and co-workers examined Au-based alloys (M = Mo, Fe, Co, Ni) using an ECW approach,^519^ demonstrating that alloy formation feasibility scales in the order NiAu > CoAu > FeAu > MoAu. While the presence of adsorbed O_2_ stabilizes the Ni, Co, and Fe variants, the high oxygen affinity of Mo hinders its reduction. Based on calculated N_2_ adsorption energies and excited-state configurations, FeAu and MoAu were identified as promising candidates for plasmon-driven ammonia synthesis via nonthermal energy-transfer pathways that lower the N–N dissociation barrier.^520,521^

To expand these descriptors to alternative feedstocks, Montemore and co-workers modeled the N_2_, H_2_O, CH_4_, and CO_2_ activation over Cu-based dilute alloys (TiCu, AgCu, MoCu, RuCu, and ReCu) subject to an oscillating electric field (Figure 53).^522^ They established that localized charge-transfer oscillations and adsorbate-dopant orbital overlap serve as effective, computationally accessible descriptors for predicting light-induced bond elongation. They found that both are effective in describing the observed trends, with orbital overlap being more computationally accessible. A notable inversion in this trend occurs during CO_2_ activation on MoCu, ReCu, and RuCu; here, strong ground-state chemical bonding alters the linear electronic structure of the molecule, which suppresses subsequent field-driven charge transfer and limits photoassisted activation.

**Figure 53:**
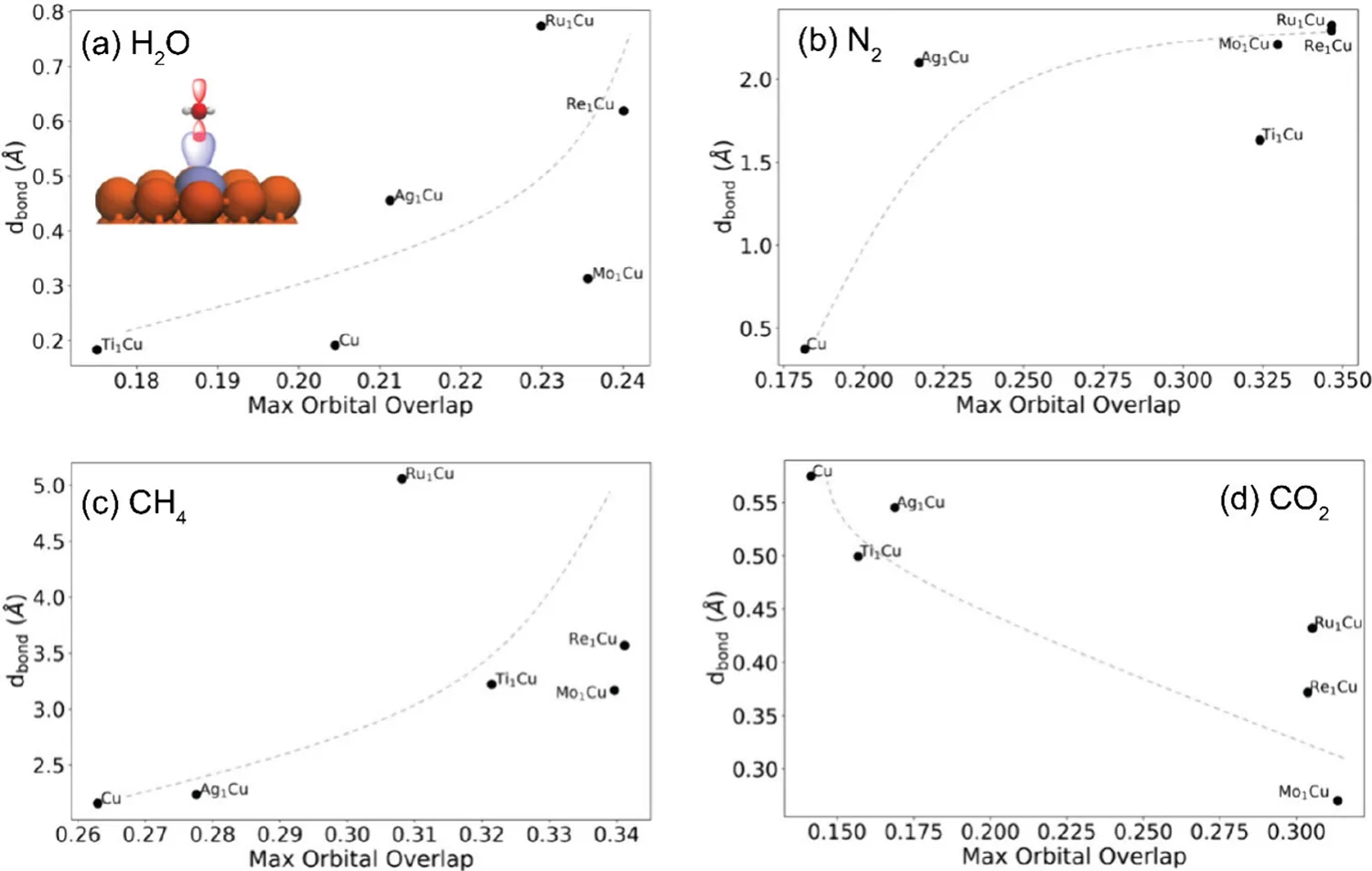
Correlating the orbital overlap between adsorbates and dopants with bond elongation under an oscillating electric field for (a) H_2_O dissociation, (b) N_2_ dissociation, (c) CH_4_ activation, and (d) CO_2_ dissociation, with an inset showing a schematic illustration of the orbital overlap between a water molecule and dopant sites. Reproduced from ref.^522^ Copyright 2025 The Authors. Licensed under CC BY 4.0.

Other computational studies have also shown that different dopants can lead to quite different predicted photoactivation. For example, simulations of N_2_ photoactivation on Ag nanowires with various single-atom dopants found significant differences between dopants, with Au showing the strongest activation and no clear correspondence between N_2_ binding energy and excited-state dynamics.^523^ Similarly, a study of methane activation on Ag nanoclusters with single-atom dopants found that dopants can facilitate photoactivation, with Ga appearing particularly promising.^524^

Correlating reactivity trends with simple, accessible descriptors has yielded promising results in thermal SAA catalysis.^56,60^ However, systematic investigations of reactivity trends in dilute and single atom plasmonic alloys remain scarce. These processes can be further complicated by synthesis conditions and thermal- or plasmon-induced active-site dynamics that are not accurately captured between experiment and modeling efforts. Therefore, developing a theoretical understanding of a wide range of dopants in plasmonic hosts—whether in terms of hot-carrier distribution, bond activation, or bond formation^504,525^ —will provide invaluable insights for the rational design of next-generation plasmonic catalysts and may even extend to earth-abundant elements that are typically less active in thermal catalysis due to unfavorable binding strengths or reaction landscapes.

### 5.4 Reactions on Dilute and Single Atom Plasmonic Alloys

In this section, we discuss plasmon-enhanced hydrogenation, dehydrogenation, oxidation, and other reactions. Due to the early stage of plasmonic SAA research, we have chosen to highlight both DPAs and SAAs, but specify when rigorous characterization of isolated dopants was reported and achieved.

#### 5.4.1 Hydrogenation

##### 5.4.1.1 Semi-Hydrogenation of Unsaturated Hydrocarbons

Hydrogenation reactions are among the oldest catalytic transformations, converting unsaturated hydrocarbons into value-added products. For example, acetylene, formed as a side product of naphtha cracking, can deactivate downstream ethylene polymerization catalysts, necessitating its removal at the ppm level.^526,527^ In most cases, the rate-limiting step is the dissociation of H_2_. Early work from Halas and co-workers demonstrated the potential of leveraging hot-carrier chemistry to promote H_2_ dissociation, even on thermodynamically unfavorable surfaces such as Au nanoparticles.^472,528,529^ This concept was further exemplified by Pd–Al systems, which served as a proof-of-concept antenna–reactor model for the selective semihydrogenation of acetylene over pure Pd, as discussed in the previous section.^449^

More recently, this reaction has been employed as a model system to demonstrate the potential automation of photocatalytic testing on PdAu for Arrhenius analysis, rate-order determination, and power- and wavelength-dependent studies.^530^ However, this system exhibited a slightly increased activation barrier under light irradiation and negligible changes in rate order, suggesting that a photothermal process could dominate and even lead to photoinhibition of the semihydrogenation reaction. Ringe and co-workers reported a Pd-decorated Mg nanoparticle system (Pd_0.03_Mg). However, the nature of the Pd sites-whether isolated single atoms or clusters-was not clearly established. The catalyst exhibited a reduced activation barrier and wavelength-dependent behavior, with a more pronounced decrease observed under 785 nm illumination.^531^ This study also highlights the potential of expanding plasmonic hosts to other earth-abundant elements such as Mg.

Overall, plasmon-assisted catalysis offers a promising route for acetylene semihydrogenation, although the mechanism remains unclear-specifically, whether light promotes H_2_ desorption and how it varies across different bimetallic systems (e.g., Pd–Al vs Pd decorated Mg nanoparticles). Incorporating Mg as a plasmonic host with a dopant provides an exciting opportunity to expand the catalyst library, but further studies are needed to establish the stability of these bimetallic combinations.

##### 5.4.1.2 CO_2_ Hydrogenation

CO_2_ valorization, whether through RWGS, CO_2_ methanation, or the more challenging conversion of CO_2_ to C_2_ hydrocarbons and oxygenates, promotes efficient carbon utilization, and light-assisted CO_2_ hydrogenation has emerged as a promising approach in this regard. Xiong and co-workers fabricated an Au metamaterial,^532^ with a Au–SiO_2_–Au (ASA) sandwich structure,^533^ where the top patterned Au layer suppresses the reflection of electromagnetic waves, the SiO_2_ spacer damps the electromagnetic field, and the Au mirror restrains light transmission. Cu_1_Ag_8_ dilute alloys were subsequently integrated into this structure for light-assisted CO_2_ hydrogenation. The ASA–Cu_1_ Ag_8_ system exhibited enhanced CO and CH_4_ formation compared to its Au, Ag, and Cu counterparts and could operate under broadband light, thereby maximizing solar utilization. Subsequent DFT calculations revealed that CO adsorbs in an atop configuration on the Cu site for both Cu_1_Ag_8_(111) and Cu_1_Ag_8_(220). While the weaker CO binding on (111) favors CO formation, the stronger CO binding on (220) stabilizes CO and promotes further hydrogenation to CH_4_.

RuCu alloys have also been used for plasmonic catalysis. Ye and co-workers combined XAS, XPS and UV–vis spectroscopy to show that Ru–Cu interactions evolve under photoassisted CO_2_ hydrogenation, consistent with the *in situ* formation of a RuCu alloyed structure.^534^ The Ru_0.1_Cu/MgAl_2_O_4_ catalyst exhibited ∼ 99% selectivity toward methanol formation, along with an enhanced methanol yield, while the RWGS reaction was suppressed under light irradiation (Figure 54 **b,c**). In contrast, the Ru_0.1_Cu/Al_2_O_3_ reference catalyst favored the RWGS and methanation pathways, highlighting the crucial role of the oxide support in promoting methanol formation even in the presence of plasmonic excitation and active site engineering. Nevertheless, the mechanistic basis of this support effect remained unclear. *In-situ* DRIFTS measurements during light-assisted CO_2_ hydrogenation further revealed the accumulation of peaks at 2961, 2922, and 2851 cm^–1^, corresponding to adsorbed HCOO* species and the asymmetric and symmetric C–H stretching modes of CH_3_O* (Figure 54d). These observations suggest that methanol formation likely proceeds *via* the formate-methoxy pathway.

**Figure 54:**
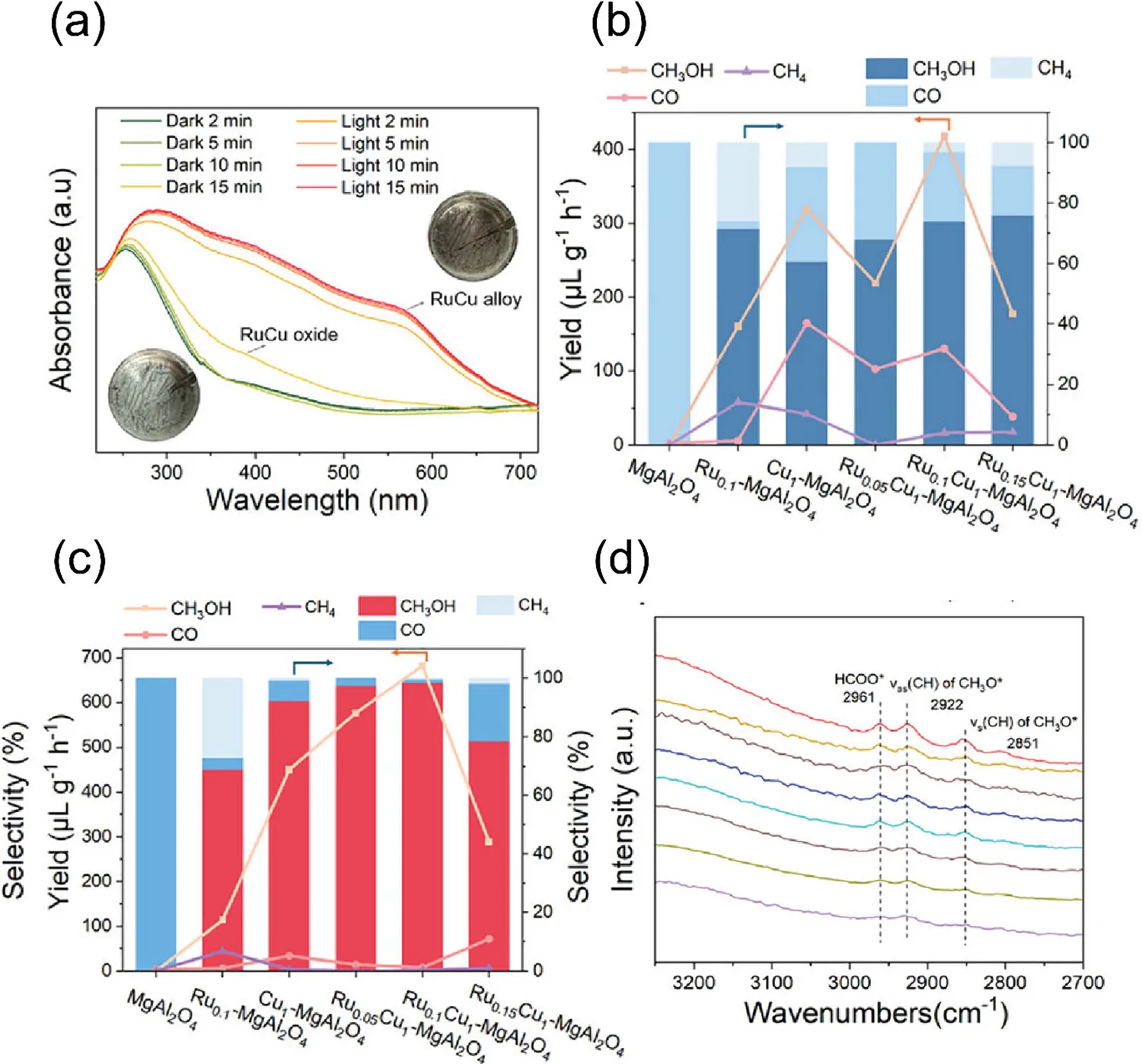
(a) *In-situ* UV–vis spectra monitoring the LSPR and oxidation state of Cu in the RuCu bimetallic system. (b) Thermal and (c) photocatalytic performance of Cu_*x*_Ru_*y*_-MgAl_2_O_4_. (d) *In-situ* IR spectra under light illumination over Ru_0.1_Cu-MgAl_2_O_4_, monitoring surface species during CO_2_ hydrogenation. Adapted with permission from ref.^534^ Copyright 2025 Wiley-VCH GmbH.

Tanksale and co-workers further demonstrated CO_2_ hydrogenation at ambient temperature in aqueous solution by varying Pt loadings in PtAg nanoprisms.^535^ The formation of CH_3_COOH, a C–C coupling product, increased with Pt loading, suggesting that Pt facilitates C–C coupling. Wavelength-dependent studies revealed that the in-plane dipolar resonance is the primary contributor to the observed reactivity enhancement. By varying the intermediates (HCO_3_^–^, HCOOH, HCHO, and CH_3_OH) in solution, they observed a significant increase in CH_3_COOH formation in the presence of CH_3_OH, indicating that CH_3_* is the key intermediate for CH_3_COOH production. In the absence of XAS or DRIFTS measurements, however, the nature of the Pt sites within Ag cannot be definitively identified beyond an alloyed phase. Chen and co-workers investigated light-assisted CO_2_ hydrogenation over a series of Fe-based catalysts (M = Mn, Co, Cu, Zn).^536^ For the FeCu dilute alloy catalyst, a ∼ 30% increase in CO and CH_4_ formation was observed under light illumination, while the production of C_2_^+^ species remained unchanged. The lack of enhancement in C_2_^+^ products could be attributed to light-induced CO desorption. However, detailed discussion of chain propagation and Fe carbide formation—crucial factors for C_2_^+^ product formation—was not provided in this study.

Light-assisted CO_2_ valorization represents a promising emerging approach toward a carbon-neutral energy scheme. The RWGS reaction provides an alternative route for CO production, circumventing energy-intensive methane steam or dry reforming processes. However, the catalytic performance of current plasmonic and photocatalytic systems remains insufficient to replace conventional thermal catalysts. Converting CO_2_ into methanol presents another appealing direction, yet the underlying light-assisted mechanisms remain poorly understood and warrant further investigation through transient kinetic analysis^537^ and phase-modulation spectroscopic techniques.^538^ Calculations suggest there is a trade-off between chemical preactivation of CO_2_ and subsequent photochemical activation of CO_2_, such that dopants that promote photoactivation of other molecules inhibit photoactivation of CO_2_.^522^ This may make design of DPAs for CO_2_ conversion particularly challenging. In addition, the oxide support plays an important role in dictating product selectivity, as recently demonstrated: oxide-supported catalysts favor heterolytic H_2_ dissociation, producing charged H^+^ species, whereas plasmon-driven H_2_ dissociation proceeds homolytically, yielding neutral H* species. The former pathway promotes the formation of C_2_ products.^539^

#### 5.4.2 Dehydrogenation

##### 5.4.2.1 Methane Reforming

Direct methane valorization is promising, but separating C_2_H_4_ and C_2_H_6_ products can be challenging.^526,527^ DRM and SRM, which convert methane into syngas—the feedstock for well-established FTS—represent another important route for methane utilization. Ye and co-workers^540^ investigated AuPd by varying the Pd loading in bimetallic systems and found that Pd_90_Au_10_ retained more reactivity after a 3 h photocatalytic test compared to pure Pd. *In-situ* electron spin resonance spectroscopy measurements suggested that plasmonic Pd_*y*_Au_*x*_ facilitates CO_2_ activation to produce O radicals, which promote CH_*x*_ removal and enhance reactivity under light illumination. The same group also compared PtAu and Pt catalysts,^541^ where incorporating Au enhanced light absorption around 520 nm due to the spectral overlap with the LSPR of Au. Wavelength-dependent studies showed that the rate enhancement mirrors the UV–vis absorption profile of PtAu, with the maximum enhancement coinciding with the Au LSPR. DRIFTS analysis during the DRM process revealed a pronounced decrease in CO* and COOH* species under illumination, suggesting that light promotes their removal, freeing active sites for subsequent bond activation. However, fine-tuning and characterization of the active sites are lacking in these studies.

Halas and co-workers^75^ further investigated DRM over RuCu by systematically varying the Ru loading (from single atom to ensemble-dominant sites). Ru_0.1_Cu_19.9_ and Ru_0.2_Cu_19.8_ exhibited high H_2_/CO selectivity (the main side reaction of DRM is RWGS, which can be monitored by the H_2_/CO ratio) and remained stable for ∼ 20 h under intense light illumination (19.2 W cm^–2^, Figure 55a). In contrast, Cu and Ru_0.5_Cu_19.5_, with Ru ensembles dominant, showed a gradual decay in H_2_/CO selectivity under illumination. Active sites in these RuCu dilute alloys were further probed *via* CO-DRIFTS (Figure 55b). This behavior can be rationalized by the higher C mobility and closer proximity of C* species in the latter, both of which favor C–C coupling of surface carbon leading to graphite formation. In comparison, isolated Ru sites help anchor C species and more readily accommodate O* species, enabling a highly selective DRM process. In addition, light enhances the diffusion of surface species and promotes H_2_ recombination and DIET, as evidenced by ECW calculations. However, *in situ* DRIFTS or Raman evidence is needed to further support this claim.

**Figure 55:**
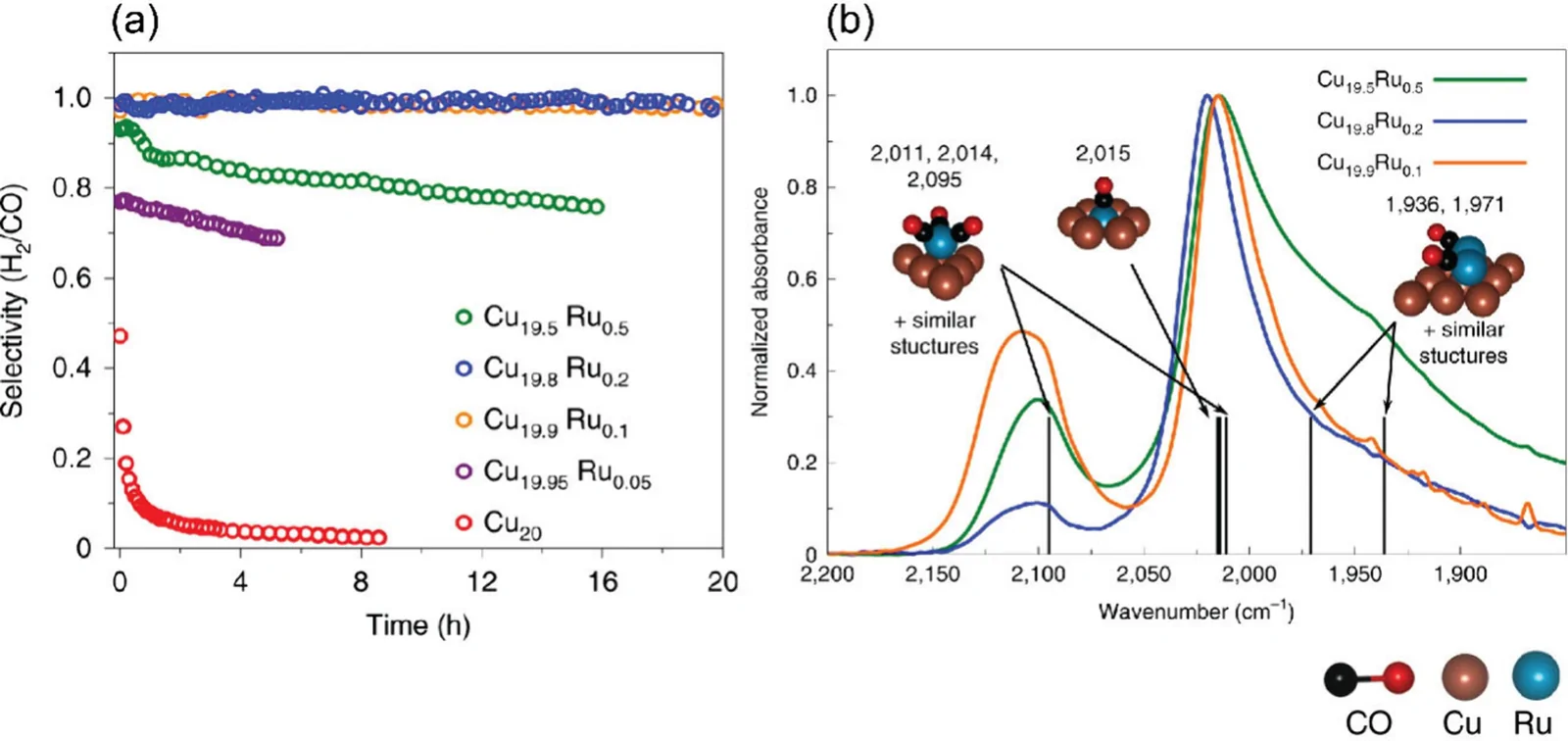
a) H_2_/CO selectivity of Cu and RuCu catalysts with various Ru loadings from methane dry reforming. b) CO DRIFTS spectra probing Cu and Ru sites in RuCu dilute alloys. Adapted from ref.^75^ Copyright 2020 Springer Nature.

The same group more recently investigated SRM over light-assisted RhCu dilute alloys (Rh_0.1_Cu_19.9_–Rh_2_Cu_18_) and found that light rejuvenates thermally deactivated catalyst surfaces, as evidenced by MS, XPS, and Raman analyses.^489^ Under light–thermal–light switching conditions, a significant decrease in H_2_ TOF and an increase in the CO_2_/H_2_ ratio were observed when switching to thermal conditions, indicating that surface oxidation and insufficient O* removal contribute to catalyst deactivation. XPS revealed an increase in oxidized Cu species under thermal conditions, accompanied by the emergence of D (∼1300 cm^–1^) and G (∼1600 cm^–1^) bands in Raman spectra, characteristic of surface carbon deposition. Returning to light conditions restored the H_2_ TOF and decreased the CO_2_/H_2_ ratio, concurrent with the recovery of metallic Cu and the disappearance of D and G band features, confirming light-induced catalyst regeneration. The light-enabled catalyst regeneration could be attributed to light-facilitated associative desorption of C* and O*, which is ineffective under phonon heating; however, further evidence, such as light-coupled chemisorption or *in situ* DRIFTS, is needed to elucidate the underlying mechanism.

##### 5.4.2.2 Alcohol Dehydrogenation and Reforming

Converting oxygen-containing species, such as ethanol, to acetaldehyde *via* dehydrogenation is another promising route for small-molecule valorization. SAAs have been extensively studied under thermal conditions, with NiCu SAAs emerging as particularly promising candidates.^95^ Ye and co-workers^542^ further demonstrated that incorporating light enables acetaldehyde production under ambient conditions, lowering the activation barrier for acetaldehyde formation from 92.4 kJ mol^–1^ and 67.4 kJ mol^–1^ on Cu and NiCu, respectively, to 61.2 kJ mol^–1^ and 41.5 kJ mol^–1^. Power- and wavelength-dependent studies showed a linear relationship consistent with the UV–vis spectrum, confirming a photochemical contribution. Moreover, the decrease in O–H stretching intensity of adsorbed ethanol further supports light-assisted ethanol dehydrogenation.

An alternative route for alcohol dehydrogenation involves cofeeding water for on-site H_2_ generation, as demonstrated by Ye and co-workers in both ZnCu^488^ and CoCu^543^ systems. For the ZnCu DPA system, a significant increase in H_2_ production rate was observed under light illumination, accompanied by a marked decrease in the activation barrier from 102.7 kJ mol^–1^ (thermal conditions) to 53.6 kJ mol^–1^ (light conditions), indicating the participation of hot carriers. *In-situ* DRIFTS measurements at 180 °C revealed a steady increase in CO_2_ peak intensity, with a distinct slope change upon light exposure, further confirming the positive effect of light. KIE analysis showed a slightly lower KIE value ( $KIE=\frac{{rate}_{H2O}}{{rate}_{D2O}}$ ) on ZnCu (KIE = 1.36) than on Cu (KIE = 1.47) under dark conditions, suggesting a more favorable reaction complex that facilitates O–H and O–D bond cleavage. Upon illumination, the KIE value of ZnCu over water dissociation rate increased from 1.36 to 1.54, implying the involvement of vibrationally excited states and further supporting hot-carrier participation.^471^ Similar light-induced enhancement was also observed on CoCu SAA,^543^ demonstrating the general applicability of incorporating earth-abundant elements as dopants in DPAs for this reaction.

##### 5.4.2.3 Alkane Dehydrogenation

Direct alkane dehydrogenation presents an intriguing alternative to naphtha cracking for olefin production. However, this reaction requires substantial energy input to activate the inert C–H bond. Conventional Pt and Ni catalysts suffer from severe coking, as they also promote C–C bond cleavage, leading to surface carbon accumulation.^544,545^ Dispersing reactive dopants into a metallic host can tune the electronic structure, modulate adsorption strength, and alter decomposition pathways from energetically downhill to uphill, thereby suppressing deep dehydrogenation.^38,56^ Among such systems, PtCu SAAs stand out as promising candidates for propane dehydrogenation under thermal conditions.^68^

Swearer and co-workers advanced this concept by incorporating light, directly comparing Pt single atom and ensemble sites in funneling photon energy to facilitate C–H activation.^505^ A more pronounced rate enhancement under light illumination was observed on the PtCu (1:246) SAA, whereas a moderate enhancement was observed on the PtCu (1:70) DA (Figure 56 **a,b**). This was further supported by Arrhenius analysis, which revealed an approximately 31 kJ mol^–1^ reduction in activation barrier for the PtCu SAA within the 400–520 °C range, suggesting the participation of hot carriers in the bond activation process. The claim was further corroborated by KIE studies comparing olefin formation rates using CH_3_CH_2_CH_3_ and CH_3_CD_2_CH_3_: an increase in KIE value was observed on the PtCu (1:246) SAA under light illumination, while negligible change occurred on the PtCu (1:70) DPA (Figure 56c). The higher KIE value under illumination indicates the involvement of vibrationally excited states generated through plasmon decay, which impact C–H­(D) scission on the central carbon, particularly for PtCu (1:246) SAA. Swearer and co-workers also compared light-enhanced propane dehydrogenation over RhCu and PtCu SAAs and found that RhCu favors a plasmon-mediated photothermal mechanism, whereas PtCu exhibits a distinct photochemical response. This comparison highlights the critical role of dopant identity in governing the pathways of light-enhanced propane dehydrogenation.^546^

**Figure 56:**
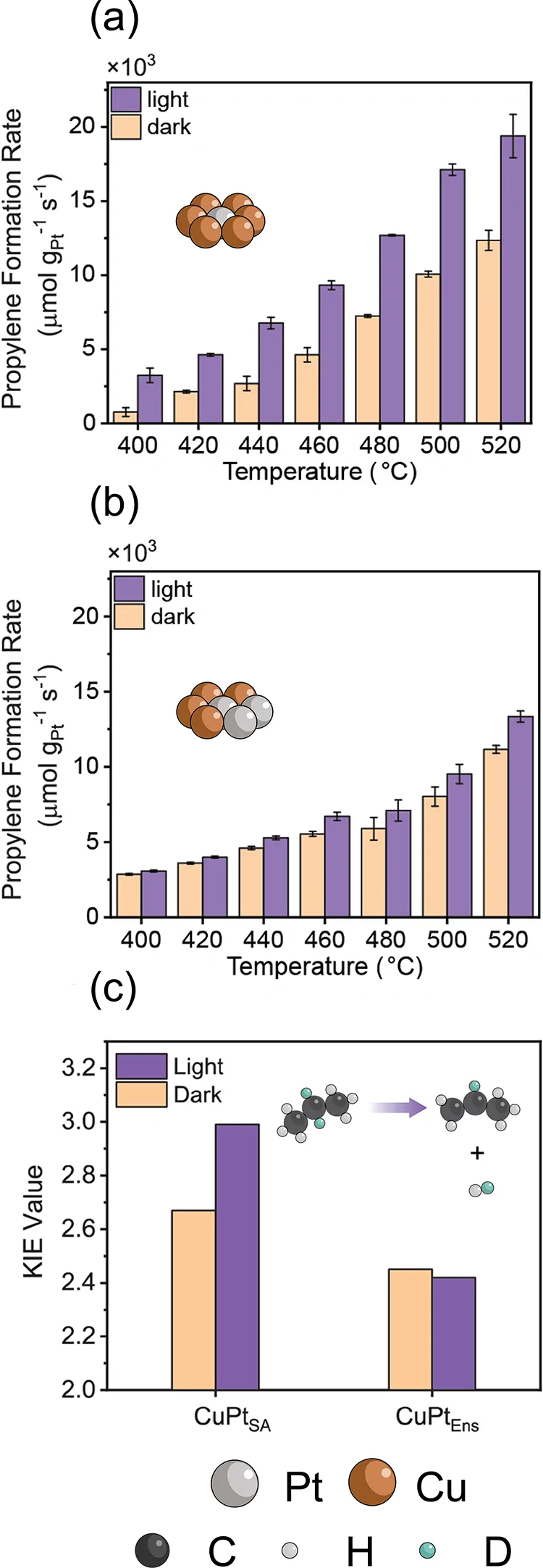
Propylene formation rate over (a) PtCu (1:246) SAA and (b) PtCu (1:70) DA. (c) KIE measurement comparing propylene formation rates between CH_3_CH_2_CH_3_ and CH_3_CD_2_CH_3_. Inset shows the schematic illustration of the KIE experiment. Adapted with permission from ref.^505^ Copyright 2025 American Chemical Society.

#### 5.4.3 Selective Oxidation

##### 5.4.3.1 Alcohol Oxidation

Selective oxidation is an important process in chemical production, and monometallic plasmonic catalysts have shown promise for olefin epoxidation.^470,473^ Introducing isolated dopant sites can further enhance the catalytic performance of plasmonic metal hosts. Lee and co-workers adapted a reported colloidal synthesis method^547^ to systematically evaluate the optical and electronic properties of PdAu alloys with varying Pd loading (Figure 57a).^548^ They found that Pd_10_Au_90_, dominated by Pd single atom sites with a less pronounced presence of Pd clusters, lies at the top of the observed volcano plot for the selective oxidation of BOH to benzaldehyde, showing a ∼ 186% activity photoenhancement with nearly 100% selectivity (Figure 57b). The negligible temperature increase supports a nonthermal pathway, attributed to plasmon-enhanced near-field effects and efficient hot-carrier transfer from Au to isolated Pd sites. The colloidal approach, with well-controlled Pd loading and surface composition, provides an effective strategy to investigate how dopant identity influences charge transfer and electronic structure, and how these factors correlate with the observed light-enhanced reaction rates.

**Figure 57:**
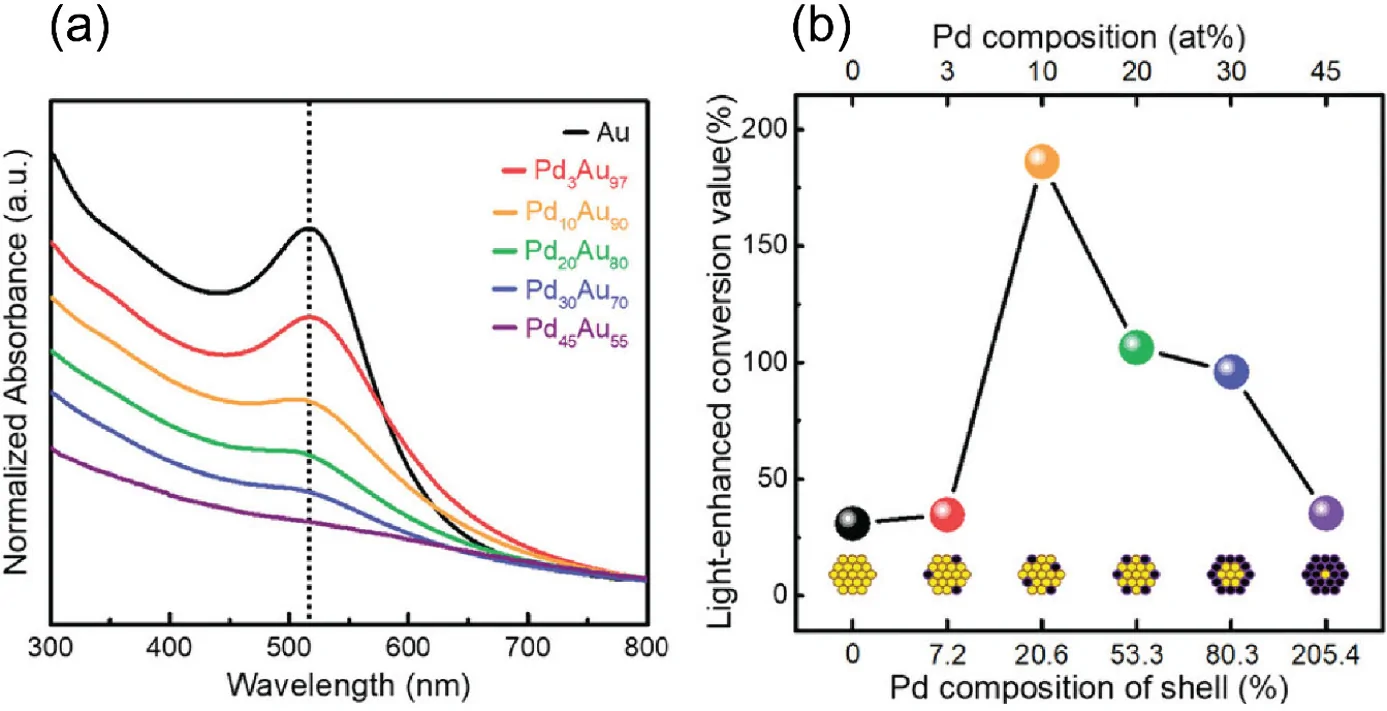
a) Normalized UV–vis spectra of PdAu nanoparticles with varying Pd loadings. b) Benzyl alcohol oxidation activity as a function of Pd loading. Adapted from ref.^548^ Copyright 2024 The Authors. Licensed under CC BY 4.0.

##### 5.4.3.2 Oxidative Methane Coupling

Methane coupling is challenging due to the high temperature typically required to activate the inert C–H bond (C–H bond dissociation energy 439 kJ mol^–1^) and has been extensively studied in thermal catalysis, although interest in the field has declined in recent decades.^549^ Photocatalytic approaches have therefore emerged as an alternative. Pioneered by Long and co-workers, initial strategies utilized an oxide–metal synergy to drive methane coupling in the presence of water over Au/ZnO catalysts.^550^ In this architecture, oxygen vacancies within the ZnO plane serve as active sites to accommodate and activate CH_4_, while the Au cocatalyst acts as an electron sink, rendering the ZnO support electron-deficient to further accelerate C–H cleavage. Ye and co-workers subsequently advanced this paradigm into a continuous, steady-state oxidative methane coupling process over a hybrid Au/ZnO/TiO_2_ system.^551^ Through a combination of O_2_-TPR, *in situ* EPR, H_2_^18^O isotopic exchange, and DFT calculations, they decoupled the multicomponent cooperative mechanics: introducing TiO_2_ facilitates controlled O_2_ adsorption and activation, while ZnO manages CH_4_ chemisorption. Crucially, *in situ* DRIFTS and DFT calculations benchmarking against a combustion-heavy Pt/ZnO/TiO_2_ reference revealed that Pt aggressively promotes the coupling of surface CH_3_* and O* into a highly stable surface methoxy (OCH_3_*) intermediate-the key kinetic precursor for deep oxidation to CO and CO_2_. In stark contrast, Au exhibits a moderate binding affinity for CH_3_*, effectively suppressing methoxy formation and facilitating the rapid desorption of free methyl radicals (CH_3_^•^) into the gas phase for selective C–C coupling to C_2_ and C_3_ products. Beyond interfacial site selection, the macro-environmental CH_4_/O_2_ feed ratio must be precisely tuned to regulate surface coverage; optimizing this ratio prevents the close structural proximity of adjacent CH_3_* and O* species, choking off the pathway to unwanted methoxy formation. Building upon these foundational principles of intermediate management, Xiong and co-workers further explored an isolated PdAu/ZnO SAA system.^552^ This architecture leverages the established advantage of ZnO for CH_4_ accommodation and Au for charge separation, but utilizes well-dispersed, isolated Pd sites to intentionally drive downstream C–H bond cleavage, shifting the ultimate selectivity from ethane to ethylene. This hypothesis is further supported by TPR results, which show the formation of CO and H_2_—decomposition products of methoxy species—and by catalytic testing, where introducing ethane to PdAu/ZnO, compared to Au/ZnO, resulted in ∼ 89.4% ethylene selectivity.

Building on this work, Tang and co-workers prepared TiO_2_-supported Pd-containing alloys and tested oxidative methane coupling under steady-state conditions.^553^ They found that PdCu alloys exhibited the highest C_2_ formation rate among the candidates (PdFe, PdNi and PdCo) (Figure 58a) and that the photocatalysts remained stable for ∼ 112 h with a C_2_ formation rate of ∼ 48 μmol·h^-1^ at a gas hourly space velocity (GHSV) of 342,000 mL gcat^-1^ h^-1^ and a CH_4_:O_2_ ratio of 373:1 (Figure 58b). They further investigated charge transfer between Cu, Pd, and the TiO2 support via in situ electron paramagnetic resonance (EPR) spectroscopy, XPS, and XAS. In the EPR spectra (Figure 58c), TiO_2_ under light illumination yielded two sets of g-values: (2.003, 2.014, 2.017) and (2.003, 2.017, 2.027), corresponding to bridge and terminal oxygen radicals, respectively. Incorporating Pd lowered both sets of g-values, suggesting photogenerated hole transfer from TiO_2_ to Pd sites. This signal further decreased when the gas environment was switched from Ar to CH_4_, with CH_4_ acting as a photogenerated hole scavenger. Charge transfer was also observed in XPS, where Pd 3d_5/2_ and Cu 2p_3/2_ exhibited positive shifts while Ti 2p_3/2_ exhibited a negative shift. XANES measurements showed a decrease in the Ti L-edge DOS under light, while the Cu L-edge increased (Figure 58 **d,e**). Together, these characterizations suggest that PdCu acts as a photogenerated hole acceptor, softening the C–H bond in methane to promote activation and enabling H spillover to TiO_2_ sites to recombine with O radicals, thus completing the reaction loop. It is worth noting that this study supports the theoretical prediction that dopant sites can localize hot holes^517^ and could facilitate site-specific bond activation.

**Figure 58:**
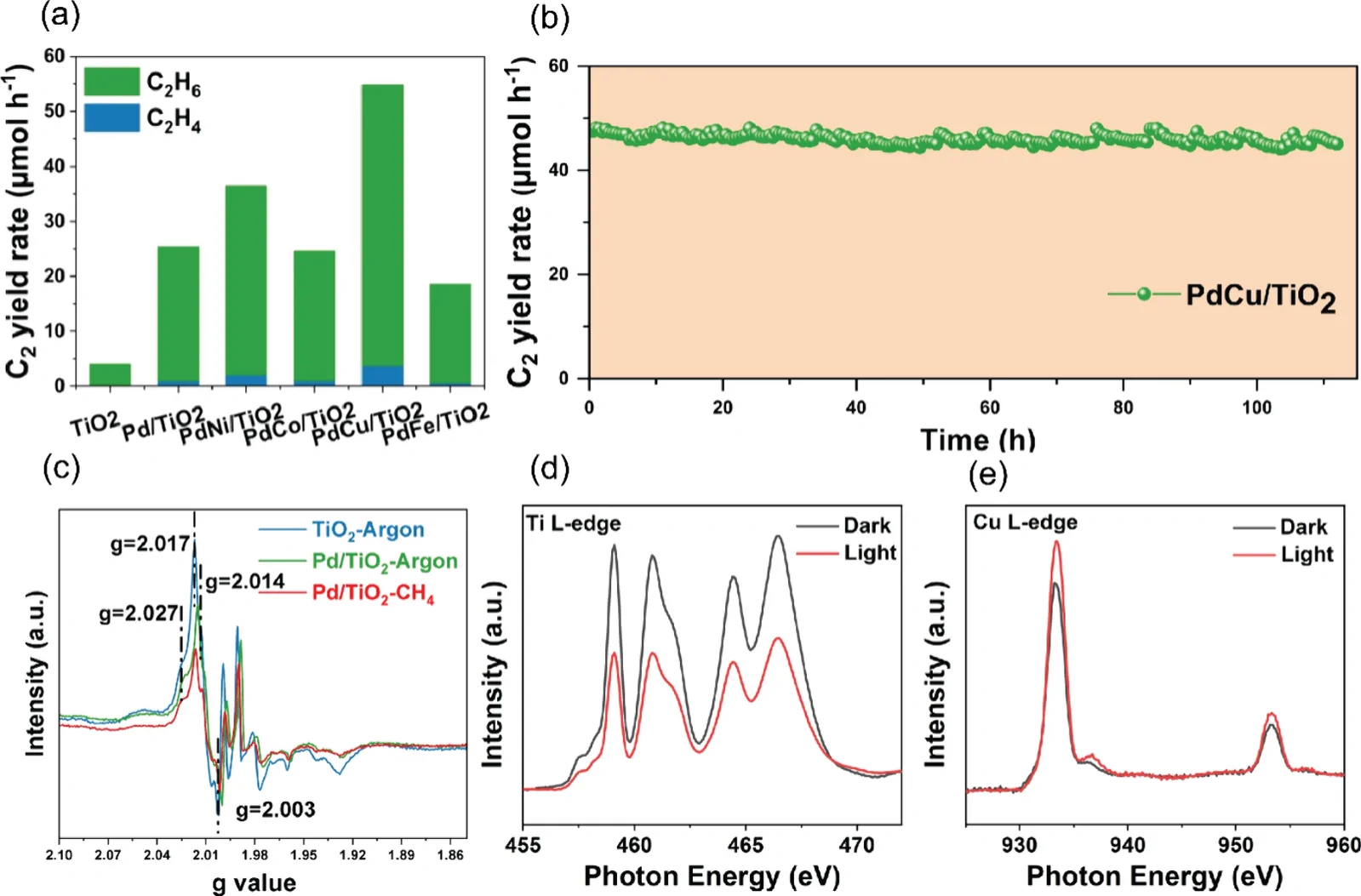
a) Catalytic performance of TiO_2_-supported Pd-containing alloys for photoassisted oxidative methane coupling. b) Stability test of PdCu/TiO_2_. c) *In-situ* EPR spectrum of TiO_2_ under Ar, Pd/TiO_2_ under Ar, and Pd/TiO_2_ in a CH_4_ environment. d) XANES spectrum of the Ti L-edge of PdCu/TiO_2_. e) Cu L-edge of PdCu/TiO_2_ under dark and light irradiation. Adapted from ref.^553^ Copyright 2023 The Authors. Licensed under CC BY 4.0.

Overall, selective oxidation-particularly for methane activation-represents one of the most formidable yet promising frontiers in catalysis, where photochemically driven approaches offer unique thermodynamic advantages for mild-condition methane valorization. Bypassing traditional scaling relationships requires delicate interfacial engineering that balances cooperative oxide-metal synergies, oxygen-assisted C–H activation, hot-carrier separation, and intermediate spillover, while strictly regulating surface coverages. As demonstrated across these benchmark systems, advanced *in situ* spectroscopies (including DRIFTS, EPR, and TPR) are indispensable for uncovering transient intermediates, mapping reaction pathways, tracking lattice oxygen participation, and quantifying the surface residence times of reactive oxygen species. Looking forward, the field must continue leveraging these *operando* techniques to gain molecular-level insights, while simultaneously extracting generalizable electronic descriptors. Correlating fundamental properties-such as projected density of states (PDOS), Bader charges, and work functions-with the competitive kinetics of C–H activation versus CH_3_*-O* coupling will be vital. Ultimately, exploiting these descriptors to guide strategic dopant incorporation, such as single-atom alloys, offers a rational blueprint to unlock targeted downstream dehydrogenation pathways for direct methane-to-olefin valorization.

#### 5.4.4 N-Related Chemistry

##### 5.4.4.1 Ammonia Decomposition

Ammonia is a carbon-free hydrogen carrier with favorable volumetric and gravimetric energy densities for energy storage and transport, offering an alternative pathway to H_2_ storage and delivery.^554^ Identifying catalysts capable of efficient ammonia decomposition under ambient conditions could help enable a sustainable hydrogen economy. Ru-based materials are promising candidates for this purpose.^555^ *It is worth noting that these bimetallic systems operate in the dilute-alloy regime, containing a mixture of single atom sites and small clusters.*

Halas and co-workers incorporated Ru into a plasmonic Cu host and disentangled the thermal and nonthermal contributions under light illumination by varying the wavelength and light intensity (Figure 59a).^53^ Notably, illumination changes the reaction order from zeroth order in the dark to pseudo-first order under light, demonstrating that hot carriers can alter the reaction mechanism (Figure 59b). They proposed that light induces DIET, which facilitates the recombination and desorption of strongly bound N* species (Figure 59c). Carter and co-workers^453^ applied the ECW model to further resolve the mechanistic details by comparing the first N–H activation and N–N recombination—two sluggish steps in ammonia decomposition^556^—under ground-state and various excited-state conditions. They proposed that, during the first N–H activation, the lowest eight excited states S1–S8 can be accessed under illumination, somewhat lowering the effective barrier from 1.35 to 0.95 eV. During N–N recombination, higher excited states can be accessed, and the barrier is significantly lowered from 2.84 eV to 1.16 or 0.65 eV depending on the specific excitation. Thus, accessing these excited states shifts the rate-limiting step from N association to N–H activation, consistent with experimental observations (Figure 59 **d,e**).

**Figure 59:**
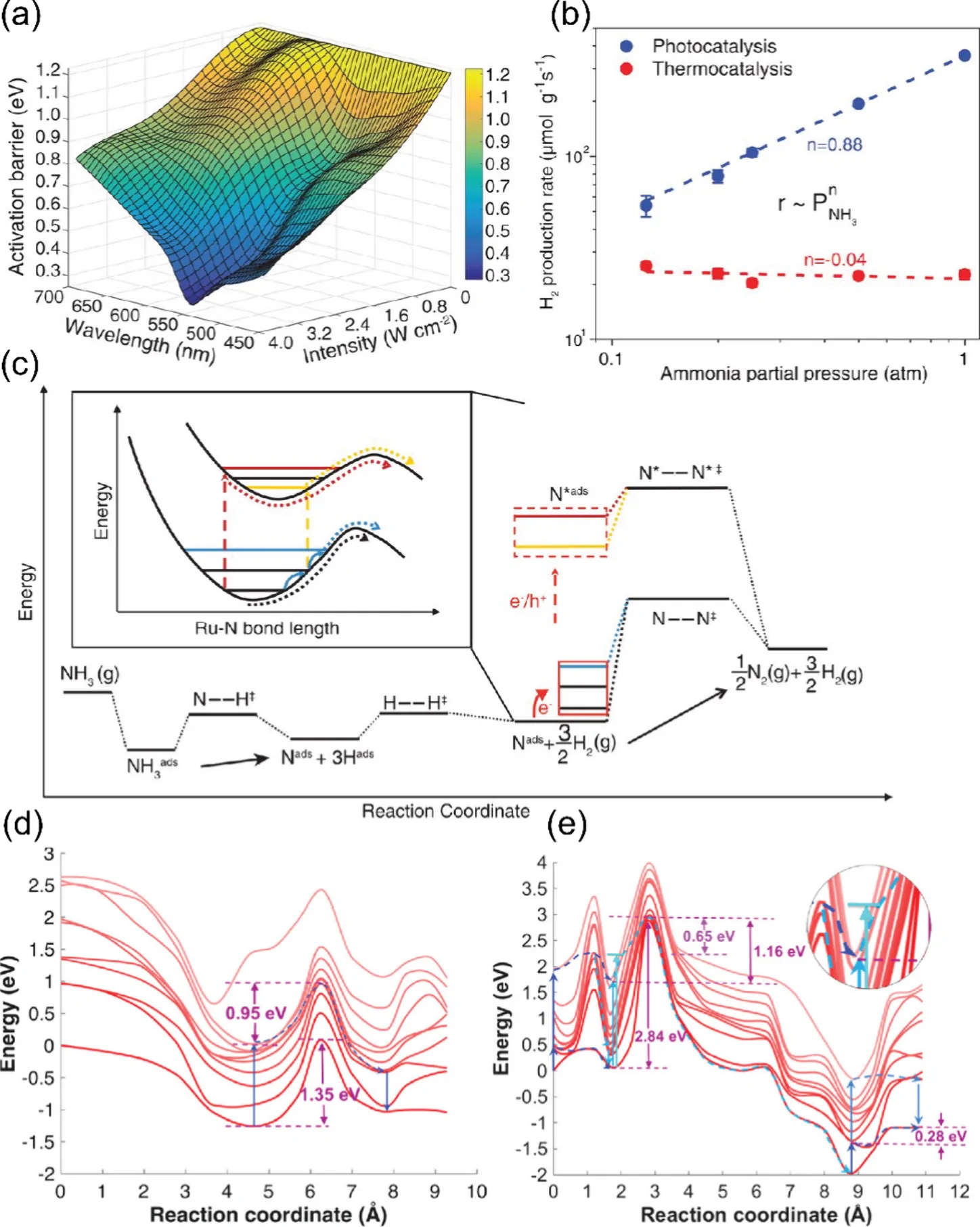
a) Identifying the optimal wavelength and light-intensity conditions by examining the apparent activation barriers as a function of these parameters. b) Rate order as a function of NH_3_ partial pressure at 425 °C under 6.4 W cm^–2^ white light. c) Energy landscape for light-assisted ammonia decomposition over RuCu. Adapted with permission from ref.^53^ Copyright 2018 AAAS. d) Ground-state and S1–S9 excited-state pathways for the first N–H bond activation and e) N–N recombination. Adapted with permission from ref.^453^ Copyright 2019 American Chemical Society. Arrows denote the potential excited pathway for bond activation and dissociation processes.

Halas and co-workers explored earth-abundant elements as reactive dopants for this reaction.^452^ They introduced Fe, which is typically an inferior candidate for ammonia decomposition under thermal conditions due to its strong binding of N* species. However, under light illumination without external heating, the FeCu exhibits ammonia decomposition activity (∼ 0.435 mmol g^–1^ s^–1^) comparable to state-of-the-art Ru-based catalysts operated at 623 K.^557^ ECW calculations suggest that illumination destabilizes the initial NH_3_* and N* states, thereby lowering the activation barriers for N–H activation (from 1.20 eV involving a quintet-to-triplet spin-state crossing, down to 0.53 eV) and N–N association (from 2.67 eV, with spin-state crossing, down to 0.50 eV). They further evaluated FeCu under more practical conditions and found that gram-scale (g/day) H_2_ production can be achieved when 3.7 g of FeCu is used under constant 140 W (4.1 W cm^–2^), 470 nm light illumination (LED) in the absence of external heating.

Overall, these studies on DPAs, though not in the single atom regime, offer new opportunities to decouple photothermal and photochemical processes, highlighting the potential of using light to alter the rate-limiting step and thereby change the reaction mechanism. In addition, accessing excited states can modulate binding characteristics that typically hinder earth-abundant metals like Fe from being effective candidates, thereby lowering activation barriers and potentially expanding the catalyst library from noble to non-noble metals.

##### 5.4.4.2 Ammonia Synthesis

Ammonia synthesis is one of the most challenging catalytic reactions because the N–N triple bond in dinitrogen is extremely inert (bond dissociation energy 945 kJ/mol^413–415^ ). As a result, high temperatures and pressures are required to simultaneously overcome both thermodynamic and kinetic limitations in the commercial Haber–Bosch process. Developing more sustainable pathways that operate under ambient conditions is therefore essential for a carbon-neutral economy.^558^

Xiong and co-workers synthesized RuAu DPA for light-assisted ammonia synthesis from N_2_ and H_2_O, achieving this reaction under ambient conditions in a batch reactor.^559^ They identified Ru_0.31_Au as the optimal Au/Ru ratio, as further increasing the Ru loading diminished the material’s light-harvesting capability. Evidence from ^15^N NMR and *in situ* IR confirmed ammonia formation under illumination. In addition, the presence of N* species observed by *in situ* XPS suggests that the reaction likely proceeds through a dissociative mechanism.

Dionne and co-workers have evaluated the RuAu system synthesized *via* a colloidal approach at elevated temperatures under continuous-flow conditions.^493^ They found that ammonia synthesis under illumination at milder temperatures (50–100 °C) outperformed the dark reaction rates measured at 150–360 °C, with Ru_0.2_Au identified as the optimal Ru loading. Wavelength-dependent studies suggest that the rate enhancement is most pronounced between 500–550 nm. Performing semiclassical electromagnetic simulations^560^ to calculate the hot carrier generation rate indicates that surface-assisted Landau damping, rather than interband transitions, is the driving force for light-assisted ammonia synthesis. *In-situ* DRIFTS, contrasting surface species under dark vs light + heat conditions, indicates that light decreases the coverage of NH_x_* while facilitating NH_2_* hydrogenation, thereby contributing to the observed rate enhancement in the presence of light.

Although incorporating Ru in plasmonic metals shows promising behavior for light-assisted ammonia synthesis under ambient conditions, the overall conversion remains low. Significant improvements in both reactor design and catalyst optimization are still required before this approach can become commercially viable.

### 5.5 Conclusions and Outlook in Plasmonic Catalysis

Addition of external fields such as light offers an exciting pathway to enhance the reactivity of SAAs by leveraging the strong light–matter interactions exhibited by coinage-metal hosts (Cu, Ag, and Au). Accessing photoexcited states can bypass high activation barriers imposed by thermal potential energy surfaces. Light can further regulate the binding configurations of surface species through excited-state pathways and modulate surface coverage, ultimately enabling tunable selectivity. Introducing dopants as isolated atoms or small clusters enables selective binding of surface intermediates, providing an additional handle for controlling reaction pathways. Dopants can also alter plasmon decay channels, potentially localizing hot carriers at specific sites and enabling site-specific bond reconstruction as well as light-induced desorption. Collectively, these attributes could expand the catalyst design space toward earth-abundant metals, which often bind reactants too strongly under purely thermal conditions, rendering key catalytic steps inefficient. Thus, more work is needed to understand how plasmons decay throughout SAAs and DPAs and how this may drive chemistry.

Lowering reaction barriers through light-assisted pathways can significantly reduce operating temperatures, which not only decreases overall emissions but also mitigates catalyst deactivation caused by coking and sintering. Recent studies have shown that active-site engineering and light can work synergistically to anchor individual carbon atoms, suppress C–C coupling at host metal sites, and promote selective H–H or O–O recombination, thereby preventing the accumulation of undesirable surface species.^75,489^ These effects can prolong catalyst lifetimes or even rejuvenate thermally deactivated catalysts.

Despite these advances, fundamental understanding of how light impacts catalyst stability, sintering, and selectivity remains limited.^504,525^ Most existing studies present isolated examples across diverse structural motifs—including alloys, core–shell, core–satellite, and metamaterial systems—making direct comparisons and systematic reactivity trend analyses challenging. Some studies indicate that SAAs can be particularly effective in driving photoactivation,^505^ but the generality of this conclusion is unclear. Future research should emphasize rigorous structural characterization, continuous monitoring of active-site evolution, and direct comparisons between dark and illuminated conditions. Experimental and computational approaches must continue to evolve to provide spatially and temporally resolved descriptions of plasmonic catalysis, encompassing charge transfer, plasmon decay, and energy localization processes. Completing the energy-transfer picture during plasmon decay will require probing elementary reaction steps using molecules such as CO and NO, cross-comparing different metals,^491^ and a variety of advanced characterization techniques. For example, combining time-resolved techniques such as pump–probe techniques with microscopy to offer spatial and temporal information,^561^ along with anti-Stokes Raman spectroscopy,^497,500,501^ X-ray absorption^498^ and X-ray diffraction^502,562^ could bring significant new insights. Scanning probe microscopies,^496^ such as atomic force microscopy and STM, can be further integrated to achieve nanoscale spatial resolution of plasmonic processes. Additionally, rationalizing trends across different materials and reactions is only in its infancy compared to thermal SAA catalysis. SAAs can contribute to these efforts to understand plasmonic catalysis due to their relatively well-defined active sites, along with their strong performance. Together, these advances will yield molecular-level insights into plasmonic catalysis and ultimately enable rational catalyst design.

Table 7 summarizes representative SAA systems reported in this section, together with the experimental and characterization techniques used to verify atomic isolation, the corresponding reaction type or elementary step, key catalytic performance metrics, the proposed reaction mechanism, and the relevant references.

**Table 7:** Summary of the Bimetallic Plasmonic Alloy Systems for Different Reactions^a^

**SAA system**	**Technique used to show evidence for atomic isolation**	**Reaction type**	**Key catalytic metric(s)**	**Stability test/observation**	**Proposed mechanism**	**Ref. #**
**CuAg SAA**	EXAFS	Hydrogenation (CO_2_ hydrogenation)	Near-field electromagnetic-field-driven CO_2_ hydrogenation under broadband illumination	∼88% activity retention after 72 h continuous run	Interfacial hot carrier injection from Au metasurface to Ag clusters subsequently localizes at the Cu sites, where embedded Cu atoms on Ag(111)/Ag(220) facets anchor CO*/CHO* intermediates to direct CO and CH_4_ pathways, respectively.	^533^
**NiCu SAA**	CO DRIFTS	Dehydrogenation (ethanol dehydrogenation)	Achieves a 1.7–3.3-fold enhancement in H_2_ production compared with thermal conditions across the examined temperature range	Stable performance for up to 5 h	The generation of hot electrons in Cu and their subsequent migration to Ni sites facilitates hydrogen production while suppressing charge-carrier recombination	^542^
**CoCu SAA**	CO DRIFTS	Dehydrogenation (methanol reforming)	Achieves 3.3–5.1 times higher reactivity in comparison to dark conditions across the examined temperature range (180- 220 °C)	Stable performance for up to 30 h	Co sites facilitate both methoxy C–H activation and water dissociation; however, the role of light and the underlying charge-transfer mechanism remain elusive	^543^
**PtCu SAA**	CO DRIFTS	Dehydrogenation (propane dehydrogenation with H_2_ coflow)	PtCu SAAs exhibit more than a 50% rate enhancement (400- 520 °C), outperforming PtCu catalysts dominated by Pt ensembles.	N/A	Isolated Pt sites are more effective than Pt ensembles at localizing plasmonic charge, as evidenced by increased KIE value.	^505^
**RhCu SAA**	CO DRIFTS	Dehydrogenation (propane dehydrogenation with H_2_ coflow)	Photothermal effects dominate in RhCu SAAs, resulting in only modest rate enhancement and changes in the apparent activation barrier	N/A	Pt uniquely directs energy into adsorbates, whereas Rh preferentially dissipates energy through thermal pathways	^546^
**Dilute alloy system**						
**PdAu dilute alloy**	N/A	Hydrogenation (acetylene semihydrogenation)	A slight activation barrier increase was observed	N/A	Photothermal effects and surface dynamics complicate mechanistic interpretation	^530^
**PdMg dilute alloy**	N/A	Hydrogenation (acetylene semihydrogenation)	exhibits ∼ 50% selectivity toward ethylene, alongside a 40-fold rate enhancement relative to thermal conditions at 60 °C and a nearly 2-fold decrease in the activation barrier	N/A	Wavelength-dependent rate enhancements suggest a light-driven pathway; however, the precise mechanism remains ambiguous	^531^
**NiCu dilute alloy**	CO DRIFTS	Hydrogenation (CO_2_ hydrogenation)	Achieves ∼ 4.7 times higher CO production than Cu while maintaining ∼ 95.4% selectivity	Stable performance for up to 5 h	Ni lowers the activation barriers for CO_2_ and H_2_ activation, modulates the oxidation state of Cu, and, under illumination, facilitates CO desorption to mitigate CO poisoning	^563^
**RuCu dilute alloy**	CO DRIFTS and EXAFS	Hydrogenation (CO_2_ hydrogenation)	High methanol selectivity (∼ 99%) was enabled by metal–support synergy and photothermal effects.	Maintains robust methanol formation over 60 h on stream	Interfacial MgAl_2_O_4_–RuCu synergy: Ru sites promote thermal hydroxyl (OH) generation and stabilize HCOO* and CH_3_O* intermediates; exact nonthermal plasmonic contribution vs local photothermal effects under illumination remain unverified.	^564^
	CO DRIFTS and DFT	Dehydrogenation (dry methane reforming)	RT CO_2_ assisted C–H activation of methane	Enhanced Coke resistance	Light facilitates C* and O* spillover and desorption (desorption induced by electronic transition – DIET mechanism)	^75^
**RhCu dilute alloy**	N/A - lacking	Dehydrogenation (steam methane reforming)	Enables steam reforming without external heating, and light can regenerate thermally deactivated catalysts.	Enhanced coke resistance and the ability to regenerate thermally deactivated catalysts	Light shifts the rate-limiting step from associative CO desorption to dissociative CH_4_ adsorption, clearing the crowded surface and regenerating active Rh sites	^489^
**ZnCu dilute alloy**	N/A - lacking	Dehydrogenation (methanol reforming)	Delivers 3 times higher reactivity than Cu under identical illuminated surface temperature conditions.	N/A	Zn sites promote water dissociation and enable more efficient charge transfer through favorable Zn–H orbital interactions	^488^
**PdAu dilute alloy**	N/A - lacking	Partial oxidation (selective oxidation of benzyl alcohol)	Pd_10_Au_90_ with predominantly isolated Pd sites, shows a ∼ 186% increase in activity under illumination and nearly 100% selectivity	N/A	Minimal photothermal heating suggests a nonthermal mechanism, in which near-field enhancement and hot-carrier migration from Au to isolated Pd atoms play key roles	^548^
	CO DRIFTS and EXAFS	Partial oxidation (oxidative methane coupling)	Ambient condition methane conversion to C2 products	N/A – batch reactor	Interfacial oxide-PdAu synergy: oxide generates methyl/methoxy radicals; single-atom Pd sites drive methoxy/methyl dehydrogenation and subsequent coupling for final C2 production	^552^,^565^
	EXAFS	Partial oxidation (oxidative methane coupling with N_2_O)	Under optimized conditions, white-light illumination enables the formation of C_2_–C_3_ hydrocarbons with ∼ 80% selectivity, while the corresponding thermal reaction (equivalent measured surface temperature) yields only CO and CO_2_	N/A	Plasmon-generated carriers at the metal–oxide interface alter OH coverage, thereby shifting the reaction pathway from overoxidation to selective oxidative coupling	^566^
**PdCu dilute alloy**	EXAFS	Partial oxidation (oxidative methane coupling)	Steady-State Flow Conversion of Methane to C_2_ Products under Ambient Conditions	Enhanced Coke resistance due to optimized binding strength	Methane adsorption on Pd sites softens the C–H bond, while photogenerated holes transferred from TiO_2_ to PdCu promote its subsequent cleavage, facilitating C–H activation.	^553^
**FeCu dilute alloy**	N/A - lacking	N related chemistry (ammonia decomposition)	The earth-abundant FeCu catalyst exhibits reactivity comparable to RuCu under light illumination	Achieves gram-scale H_2_ production with no apparent deactivation for ∼ 67 h	The excited initial states weaken NH_3_* and N* binding, resulting in a lower overall activation barrier	^452^
**RuAu dilute alloy**	EXAFS	N related chemistry (ammonia synthesis)	Ru_0.1_Au and Ru_0.2_Au exhibit ∼ 2-fold higher reactivity under illumination than under thermal conditions, despite operating at lower measured surface temperatures	N/A	Light modulates the binding configuration of surface intermediates, facilitating NH_2_ hydrogenation, as evidenced by in situ DRIFTS and theoretical calculations.	^567^

a Both SAAs and dilute alloys (with mixed single atom sites and ensembles) are presented given the emergence of this class of materials in plasmonic catalysis and the rather underexplored nature of quantifying the dopant identities.

## 6 Overall Outlook

In each of the sections on thermal, electrochemical, and plasmonic catalysis, we include conclusions and outlook specific to each topic. Here, we provide thoughts on the SAA field in general and emphasize the potential for accelerated growth enabled by theory-guided studies.

### 6.1 Computation-Guided Future Directions

Computation has long been used to rationalize the behavior of catalysts, and SAAs in particular have received extensive computational study due to their relatively well-defined active sites. Building on this, computation is increasingly used for a priori design of catalysts,^568–573^ including several examples of new SAAs that would not have been predicted from conventional catalytic wisdom. The well-defined nature of SAAs can also be leveraged to improve mechanistic understanding, which may in some cases extend to deeper understanding of these reactions on more traditional catalysts, particularly by comparing different SAAs to pure metals. More broadly, the breaking of scaling relations, spillover, and well-defined active sites that have enabled SAAs to catalyze a wide range of highly selective chemical reactions could be translated to other classes of materials, including compounds like oxides and chalcogenides.

Several computational studies have pointed toward early transition-metal dopants (e.g., Y, Ti, Hf) as potentially promising SAA candidates for a number of reactions, but these have received relatively little experimental attention (see Figure 60). In some cases, nontransition metals also appear to be promising. Experimentally realizing catalysts with these elements, many of which are very reactive, may require advances in synthesis and strategies to prevent overoxidation, as indicated by recent studies on TiCu dilute alloys.^574,575^ However, large scale computational screening, including the ML-based approaches critically discussed in Section 2.6.1, could be particularly useful for searching the large materials space for trimetallic extensions to SAAs, such as alloy hosts with a third metal dopant, DAAs, and trimetallic SAAs. These structures offer more tunability and hence greater potential for high performance, addressing one limitation of traditional SAAs: their design space is smaller than multimetallic alloys, such that there are fewer levers for fine-tuning properties. Although more complex materials may be harder to characterize and to prepare with well-defined active sites, their potential higher tunability motivates improved screening, synthetic and characterization methods.

**Figure 60:**
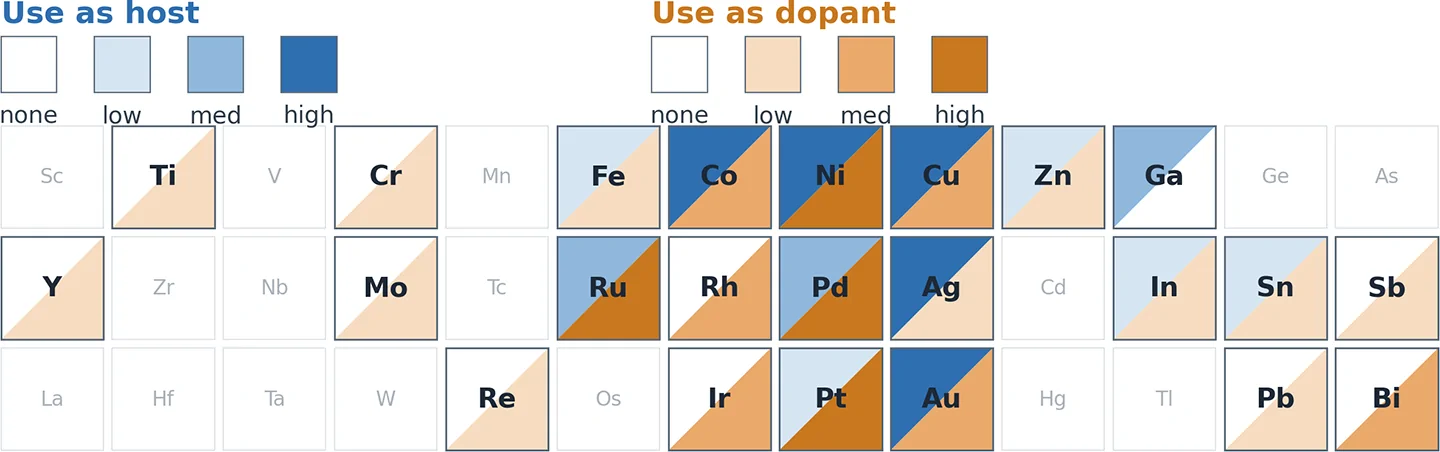
Periodic table showing use of different metals as hosts and dopants in bimetallic SAAs based on the number of experimental references in this Review as a simple, qualitative measure of research activity. The lower bin thresholds for none/low/med/high are 0, 1, 3, 9.

Closing the aforementioned computation-experimental gaps should prove very valuable for the understanding and development of all types of SAAs. For example, improved capture of adsorbate effects on structure and reactivity, consideration of multiple possible surface facets including stepped surfaces, and more detailed description of the multiple possible reaction sites (dopant and host), and diffusion between them, should all improve both design and understanding of SAAs. Electrocatalytic and plasmonic systems provide even more complexity, such as solvent effects, electrochemical potential, and electronic excitation by light. Improved characterization of these experimental systems will also help guide theoretical models. Some gap between the computational model and the actual working catalyst is likely to remain, especially for nanoparticle catalysts in a reactor. Simpler screening strategies combined with strong fundamental understanding, as well as more detailed, higher-fidelity modeling, are both useful approaches to meet this challenge.

One area where the computation-experiment comparison deserves particular future attention is the role of supports. Many SAA studies have strong correspondence between nanoparticle systems, model single-crystal systems, and computational models. While this is a clear strength of SAAs, studies have increasingly shown that supports can also play an important role in SAA performance. The large change in propane-dehydrogenation activity when switching the support (SiC vs SiO_2_ or Al_2_O_3_) for PtGa SCALMS catalysts is one striking example (Section 3.3.2). Thus, while support effects can be challenging to probe and understand, they represent a clear opportunity for further improvement in performance.

### 6.2 Concluding Discussion

SAAs have emerged as one of the most conceptually powerful platforms in heterogeneous catalysis because they combine atomically defined active sites on a wide range of bimetallic combinations with the practicality, robustness, and scalability of metal nanoparticle catalysts. Fundamental studies of SAAs have shown that the isolation of reactive guest atoms within a host metal creates catalytic sites with unusual geometric and electronic structures that are not captured by the traditional mean-field picture of alloying. SAAs represent a paradigm shift from empirical catalyst discovery toward predictive design frameworks in which mixing enthalpy, segregation behavior, molecular adsorption energetics, and electronic structure are used to identify which SAAs are likely to be catalytically active, selective, and stable. A central advance of the field has been the realization that the unique geometry of SAAs enables linear scaling relationships between the transition state energy and the reaction step energy to be broken which leads to active and selective catalysts.

Studies spanning hydrogenation, dehydrogenation, oxidation, and coupling among a plethora of other chemistries have established that SAAs are not niche materials, but rather a versatile class of catalyst that can bridge the pressure and materials gaps between model single-crystal experiments and industrially important transformations on practical catalysts. Furthermore, the benefits of SAAs have been demonstrated in thermal catalysis, electrocatalysis, and plasmon-driven so-called “antenna reactors”. Most recently, several examples of theory-guided SAA design by identifying alloy combinations that outperform expectations from conventional catalytic wisdom have been demonstrated. In this work surface science has been crucial in providing atomic-scale images of the active sites and the mechanistic validation needed to connect prediction to catalytic performance.

Looking ahead, the greatest opportunities in the SAA field lie in expanding from isolated successes for specific reactions to truly general design methodologies, the “holy grail” of heterogeneous catalysis research. However, challenges remain in experimentally validating the rapidly growing number of theoretically predicted systems. This is because understanding catalyst performance, structure, stability and restructuring under realistic conditions with experiments is time-intensive. This is especially true for more complex environments such as liquid-phase electrocatalysis, photocatalysis, and multicomponent trimetallic systems. However, the recent surge in machine-learning methods for catalysis, including interatomic potentials that accelerate DFT calculations by orders of magnitude, has come at the right time in terms of navigating the enormous composition space of SAAs, modeling the aforementioned effects beyond the single-crystal models and pointing experimentalists to the most promising candidate materials. The long-term opportunity is therefore not merely to improve existing catalysts, but to use the SAA platform to systematically discover entirely new alloy chemistries for challenging reactions, enabling selective transformations that remain beyond the reach of current industrial catalysts.

## Abbreviations

AFC: alkaline fuel cells
AQE: apparent quantum efficiency
ASA: Au–SiO2–Au
ATR-SEIRAS: attenuated total reflectance surface-enhanced infrared absorption spectroscopy
BEP: Bro̷nsted–Evans–Polanyi
BOH: benzyl alcohol
CN(s): coordination number(s)
**CO**_**2**_ **RR**: carbon dioxide reduction reaction
CRR: combustion reforming reaction
DAA(s): dual-atom alloy(s)
DAs: dilute alloys
DEMS: differential electrochemical mass spectrometry
DFT: density functional theory
DIET: desorption induced by electronic transitions
DIMET: desorption induced by multiple electronic transitions
DOS: density of states
DPA(s): dilute plasmonic alloy(s)
DQPSS: dynamical quantum processes on solid surfaces
DRM: dry reforming of methane
ECFP: element-centered fingerprint
ECW: embedded correlated wave function
EOR: ethanol oxidation reaction
EPR: electron paramagnetic resonance
EXAFS: extended X-ray absorption fine structure
FAOR: formic acid oxidation/formic acid oxidation reaction
FDCA: 2,5-furandicarboxylic acid
FTIR: Fourier-transform infrared spectroscopy
FTS: Fischer–Tropsch synthesis
HAADF: high-angle annular dark-field
HEASAs: high-entropy alloy single atom catalysts
HER: hydrogen evolution reaction
HERFD: high-energy-resolution fluorescence detection
5-HMF: 5-hydroxymethylfurfural
HOR: hydrogen oxidation reaction
IMC(s): intermetallic compound(s)
IRRAS: infrared reflection–absorption spectroscopy
KIE: kinetic isotope effect
KMC: kinetic Monte Carlo
LOHCs: liquid organic hydrogen carriers
LSPR: localized surface plasmon resonance
MGR: Menzel–Gomer–Redhead
ML: machine learning
MOF(s): metal–organic framework(s)
MOR: methanol oxidation reaction
**N**_**2**_ **RR**: nitrogen reduction reaction
NO3RR: nitrate reduction reaction
NP(s): nanoparticle/nanoparticles
ORR: oxygen reduction reaction
PDF: pair distribution function
PEM: proton exchange membrane
PEMFCs: proton exchange membrane fuel cells
PGMs: platinum-group metals
POM: partial oxidation of methane
PROX: preferential oxidation
PSA: phase-separated alloy
RAIRS: reflection–absorption infrared spectroscopy
RCT: raspberry colloid templated
RT-TDDFT: real-time time-dependent density functional theory
RT-TDDFTB: real-time density functional tight binding
RWGS: reverse water–gas shift
SAA(s): single-atom alloy(s)
SAC(s): single-atom catalyst(s)
SCALMS: supported catalytically active liquid metal solutions
SMSI: strong metal–support interaction
SPP(s): surface plasmon polariton(s)
SRM: steam reforming of methane
STEM: scanning transmission electron microscopy
STM: scanning tunneling microscopy
TEM: transmission electron microscopy
THF: tetrahydrofuran
TMs: transition metals
TNI: transient negative ion
TPD: temperature-programmed desorption
UHV: ultrahigh vacuum
VA: vinyl acetate
WGS: water–gas shift
XANES: X-ray absorption near-edge structure
XAS: X-ray absorption spectroscopy
XPS: X-ray photoelectron spectroscopy
XRD: X-ray diffraction
